# Computational drug design of novel COVID-19 inhibitor

**DOI:** 10.1186/s42269-022-00892-z

**Published:** 2022-07-15

**Authors:** David Ebuka Arthur, Benjamin Osebi Elegbe, Augustina Oyibo Aroh, Mahmoud Soliman

**Affiliations:** 1 Department of Pure and Applied Chemistry, University of Maiduguri, Maiduguri, Nigeria; 2 Department of Chemistry, Baze University Abuja, Abuja, Nigeria; 3 Department of Chemistry, Ahmadu Bello University Zaria, Zaria, Nigeria; 4 Department of Pharmaceutical Sciences, University of KwaZulu Natal, Durban, South Africa

**Keywords:** Binding energy, Covid-19, HIV-1 inhibitors, Molecular docking, SBDD

## Abstract

**Background:**

In 2003, the first case of severe acute respiratory syndrome coronavirus (SARS-CoV) was recorded. Coronaviruses (CoVs) have caused a major outbreak of human fatal pneumonia. Currently, there is no specific drug or treatment for diseases caused by SARS CoV 2. Computational approach that adopts dynamic models is widely accepted as indispensable tool in drug design but yet to be exploited in covid-19 in Zaria, Nigeria. In this study, steps were taken to advance on the successful achievements in the field of covid-19 drug, with the aid of in silico drug design technique, to create novel inhibitor drug candidates with better activity. In this study, one thousand human immunodeficiency virus (HIV1) antiviral chemical compounds from www.bindingBD.org were docked on the SARS CoV 2 main protease protein data bank identification number 6XBH (PDB ID: 6XBH) and the molecular docking score were ranked in order to identify the compounds with the highest inhibitory effects, and easy selection for future studies.

**Results:**

The docking studies showed some interesting results. Inhibitors with Index numbers 331, 741, and 819 had the highest binding affinity. Similarly, inhibitors with Index number 441, 847, and 46 had the lowest hydrogen bond energy. Inhibitor with index number 331 was reported with the lowest value (− 48.38kCal/mol). Five new compounds were designed from the selected six (6) compounds with the best binding score giving a total of thirty (30) novel compounds. The low binding energy of inhibitor with index no. 847b is unique, as most of the interaction energies are of H-bond type with amino acids (Thr26, Gly143, Ser144, Cys145, Glu166, Gln189, Hie164, Met49, Thr26, Thr25, Thr190, Asn142, Met165) resulting in an overall negative value (−16.31 kCal/mol) making it the best of all the newly designed inhibitors.

**Conclusions:**

The novel inhibitor is 2-(2-(5-amino-2-((((3-aminobenzyl)oxy)carbonyl)amino)-5-oxopentanamido)-4-(2-(tert-butyl)-4-oxo-4-(pentan-3-ylamino) butanamido)-3-hydroxybutyl) benzoic acid. The improvement it has over the parent inhibitor is from the primary amine group attached to meta position of first benzene ring and the carboxyl group attached to the ortho position of the second benzene ring. The molecular dynamics studies also show that the novel inhibitor remains stable after the study. This result makes it a better drug candidate against SARS CoV 2 main protease when compared with the co-crystallized inhibitor or any of the 1000 docked inhibitors.

**Supplementary Information:**

The online version contains supplementary material available at 10.1186/s42269-022-00892-z.

## Background

Coronaviruses (CoVs) have caused a major outbreak of human fatal pneumonia since the beginning of the twenty-first century. COVID‐19 is an acute respiratory disease caused by the RNA virus SARS‐CoV‐2. In severe cases, the infection can cause pneumonia, severe acute respiratory syndrome, kidney failure, and even death (Du et al. 2020). There is currently no specific medicine or treatment for diseases caused by SARS-CoV-2 (Huang et al. 2020).

SARS-CoV-2 virus targets cells through the viral structural spike (S) protein that binds to the angiotensin-converting enzyme 2 (ACE2) receptor. Once inside the cell, viral polyproteins are synthesized that encode for the replicase-transcriptase complex. Structural proteins are synthesized leading to completion of assembly and release of viral particles (De Groot et al. 2013; Lau et al. 2005; Reusken et al. 2013).

Hosseini and Amanlou (2020) conducted a virtual screening procedure employing docking of 1615 FDA approved drugs to identify new potential small molecule inhibitors for protease protein of COVID-19 and their result indicates that among all FDA-approved drugs, simeprevir which is used for Hepatitis C virus (HCV) NS3/4A protease inhibitor, revealed strong interaction with protease binding pocket and placed well into the pocket even better than the lopinavir-ritonavir (Abd El-Aal et al. 2022; Al-Hossainy et al. 2021; El Azab et al. 2021). Since this compound is FDA-approved and has successfully passed various testing steps, they suggested that this drug could be a potential drug for treating the COVID-19 (Hosseini & Amanlou 2020).

Motiwale et al. (2020) and friends applied molecular docking approach in conjugation with molecular dynamics (MD) simulations to find out potential inhibitors against Mpro of SARS-CoV-2 from previously reported SARS-3CL protease inhibitors. They used a total of 61, previously known inhibitors, where 4-(Sacco et al. 2020) benzoic acid, and 4-(4-methoxyphenyl)-6-oxo-2-[(2-phenylethyl)sulfanyl]-1,6-dihydropyrimidine-5-carbonitrile were reported to have minimum and maximum binding energy, respectively (Motiwale et al. 2020).

To achieve a fast and reliable drug in this current crisis, we initiated a virtual screening procedure(Gagic et al. 2020), employing docking of 1000 HIV1 protease inhibitors compounds from www.bindingBD.org over binding pocket of SARS CoV 2 main protease using 1 pdb file PDB: (6XBH) downloaded from Research Collaboratory for Structural Bioinformatics (RCSB) which represent main protease of SARS CoV 2 to identify potent inhibitors against the virus and to design a novel drug whose molecular dynamics studies will be done to ascertain the effectiveness in inhibiting the SARS-CoV-2 Mpro (Sacco et al. 2020).

The molecular docking result would provide first-hand knowledge about the interactions between the ligands and the target receptors since most of these ligands work by profoundly inhibiting the specificity and efficiency of protein (target) action (Adeniji et al. 2020; Stockwell 2000). All these will undoubtedly offer important structural insight into the design of novel COVID-19 drugs (Abd El-Aal et al. 2022; Al-Hossainy et al. 2021; El Azab et al. 2021). Besides, these methods will help to: promote savings in the cost of drug design and development, reduce the requirement for lengthy and expensive animal tests and, promote green chemistry to increase efficiency and eliminate chemical waste (DiMasi et al. 2003). Overall, the purpose of the work is to carry out a computer-aided drug design (CADD) of SARS CoV 2 main protease inhibitor.

## Methods

### Software

The following is a list of software used in this research work: Molsoft.icm-pro.v3.8.3 software built Nov 30 2014 20:23. 4.7.5. © Copyright 1989–2022, MolSoft L.L.C. 11199 Sorrento Valley Road, S209 San Diego CA 92121 (Abagyan et al. 1994), Visual Molecular Dynamics (VMD 1.9, 2011-03-14 Platforms) Windows OpenGL, CUDA (Windows XP/Vista/7/8/10 (32-bit) with OpenGL and CUDA) (Humphrey et al. 1996) and Nanoscale Molecular Dynamics (NAMD) version 2.14 (2020-08-05) Platforms Win64 software (freeware license) which were developed by the Theoretical and Computational Biophysics Group in the Beckman Institute for Advanced Science and Technology at the University of Illinois at Urbana-Champaign." (Phillips et al. 2020), Discovery studio Client v21.1.0.2098, Copyright © 2020, Dessault Systemes Biovia Corp (Biovia 2017), Molecular Operating Environment (MOE), 2020.09 Chemical Computing Group ULC, 1010 Sherbooke St. West, Suite #910, Montreal, QC, Canada, H3A 2R7, 2022 (Environment 2014) and Spartan'14, version 1.1. 2 Wavefunction, Inc 18401 Von Karman Ave., Suite 435 Irvine, CA 92612 (Wavefunction 2013; Baig et al. 2020).

### Experimental dataset

In this study, a dataset of 1000 HIV 1 antiretroviral compounds presented in Additional file 1: Table S1 was used for molecular docking and molecular dynamics studies to generate a novel inhibitor for the SARS CoV 2 main protease. These compounds are derivatives of: diazepam-2-one, benzamide, butanamide, carbamate, thiadiazepane 1,1-dioxide, thiadiazepane 1,1-dione, hexanediamide, dihydro-2H-pyran-2-one, benzenesulfonate imidazole-2-sulfonate, sulfamate,

butanediamide, chromen-2-one, sulfonamide, thiazolidine-4-carboxamide, phenylpentanamide, piperidine-2-carboxamide, benzenesulfonamide, pyridine-2-sulfonamide, pyrimidinone, coumarin, pyran-2-on.

## Molecular docking study

### Ligand preparation

The 2D structure of each inhibitor was drawn using the ChemDraw v16.0 Windows 10 (32 bit and 64 bit), Copyright 1998–2016 PerkinElmer Informatics Inc and presented in table (Arthur et al. 2020). The structures were introduced into wavefunction 14 graphic user interphase (GUI) after which the 2D structures were converted into 3D structures by selecting the view dialog box present on Spartan 14 GUI. From the build option on Spartan 14, the structures were clean by checking to minimize using molecular mechanic force field (MM+) option to remove all strain from the molecular structure. In addition, this will ensure a well-defined conformer relationship among compounds of the study (Viswanadhan, Ghose, Revankar, & Robins, 1989). From the setup calculation option on Spartan 14, the calculation was set to equilibrium geometry at the ground state using a semi-empirical PM6 (Parameterization Method 6) (Bikadi and Hazai 2009).

### Preparation of receptor

The x-ray diffraction crystal structure SARS-CoV-2 (COVID-19) main protease with PDB ID: 6XBH (Sacco et al. 2020) with a resolution of 1.60 Å was used for the study. The complexed inhibitor, (R)-3-(((2R,5S)-5-(((S)-(benzyloxy)(hydroxy)methyl) amino)-1-hydroxy-4-oxo-6-phenyl hexan-2-yl) amino) -1,3-dihydro-2H-pyrrol-2-one was removed from the chain of 6XBH where it was covalently bonded with the DNA in the receptor.

The receptor structure was imported into the Molsoft.icm-pro.v3.8.3 GUI (Arthur and Uzairu 2019), and the PDB files were converted into an internal coordinate mechanics (ICM-object) (MolSoft, 2000) by deleting the additional water molecules confined in the X-ray structure collected from the PDB data bank. All the hydrogen atoms were optimized before the receptor was then subjected to the process of molecular docking treatment (Sastry et al. 2013).

There are five different interaction potentials that contribute to the overall free binding energy established between the receptor pocket and the docked ligand (Gallicchio et al. 2010). These potentials include van der Waals potential for a hydrogen atom probe, van der Waals potential for a heavy-atom probe (generic carbon of 1.7A radius), hydrophobic energy terms, optimized electrostatic energy term, and lone-pair-based potential, which reflects directional preferences in hydrogen bonding. These energy terms are based on the all-atom vacuum force field with added functions to account for solvation free energy, desolvation energy and entropic contribution. It was shown that after each random step, full local minimization greatly improves the efficiency of the procedure (Abd El-Aal et al. 2022; Al-Hossainy et al. 2021; Ibrahim et al. 2020; Mohamed et al. 2022; Zwawi et al. 2021). The ICM program relies on global optimization of the entire flexible ligand in the receptor field and combines large-scale random moves of several types with gradient local minimization and a search history mechanism (Arthur et al. 2018).

### Molecular dynamics simulation

Molecular dynamics (MD) simulations were carried out on the 3D crystal structure of SARS-CoV-2 (COVID-19) main protease with PDB ID: 6XBH (Sacco et al. 2020) in complex with the reference inhibitor (R)-3-(((2R,5S)-5-(((S)-(benzyloxy) (hydroxy) methyl)amino)-1-hydroxy-4-oxo-6-phenylhexan-2-yl)amino)-1,3-dihydro-2H-pyrrol-2-one. The 3D crystal structure of SARS-CoV-2 (COVID-19) main protease with PDB ID: 6XBH (Sacco et al. 2020) was extracted from the crystal structure complex with reference inhibitor. This was complexed with the best inhibitor for MD simulations studies as well. MD simulations were carried out using the AMBER version 11 package with the ff99SB force field (Hornak et al. 2006).

The protein structure was surrounded with a 15 Å layer of TIP4P BOX water molecules. The electrostatic charge was neutralized by adding counter ions using the LeaP program of AMBER ver.11. After minimization, heating and equilibration, the production MD phase was carried out at 300 K for 1 ns with a time step of 1 ps (picoseconds) using the constant volume and temperature (NVT) ensemble and the Particle Mesh Ewald algorithm for the calculation of electrostatic interactions (Haspel, Zheng, Aleman, Zanuy, & Nussinov, 2017). The initial velocity of atoms was generated at 100 K in heating phase with a Maxwellian distribution and maintained. The pressure was kept at 1 bar by Berendsen weak coupling approach during equilibration (Berendsen et al. 1984).

## Results

The numerical results of this study are presented in the tables presented below. This has become necessary because of the need to correlate some of the data. Other results, such as plots and pictorial representation of the interactions between the ligands and their receptor binding sites are presented as figures.

**Fig. 1:**
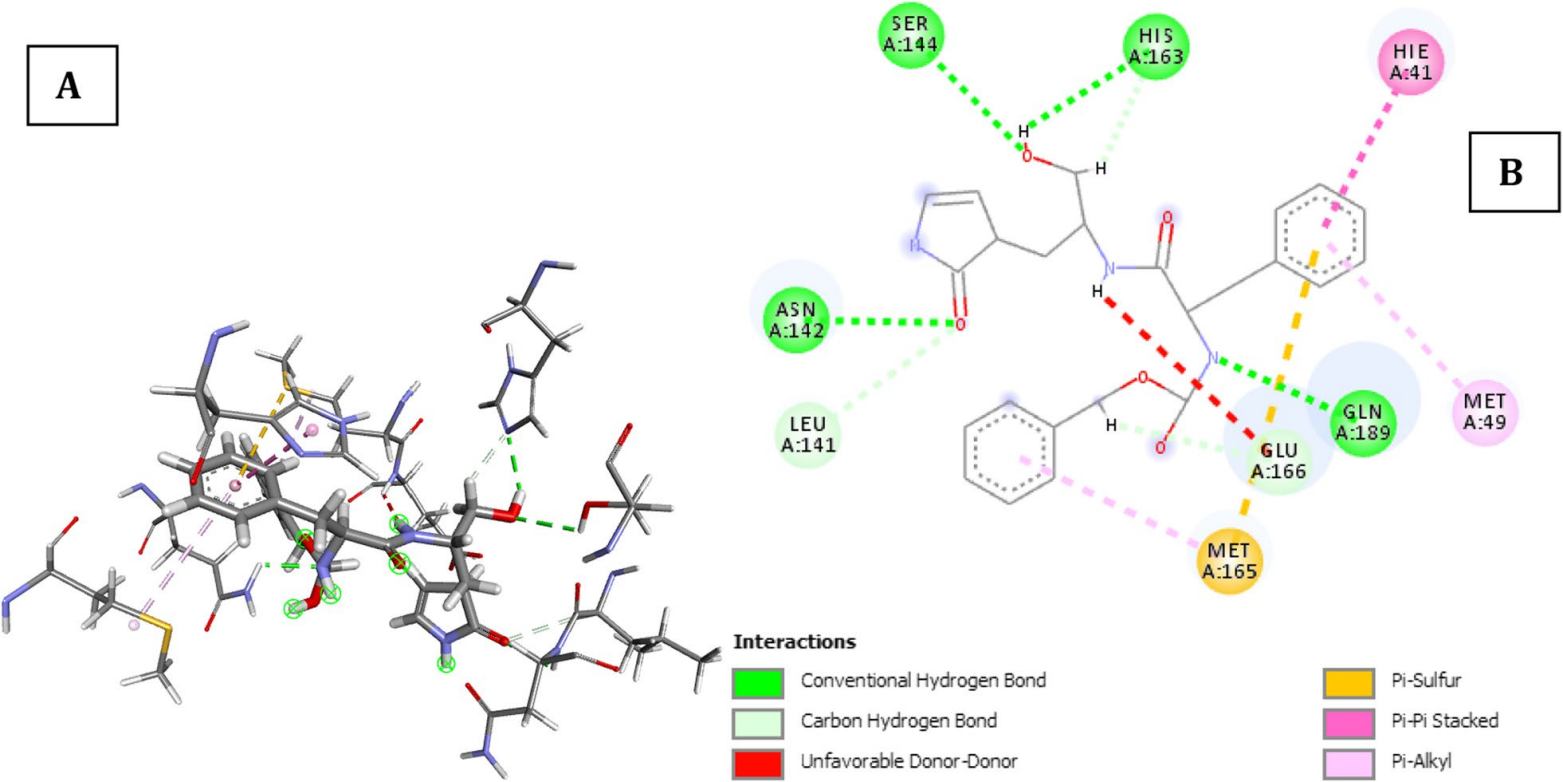
Docked poses of SARS COV-19 main protease (PDB ID: 6XBH) with REF-IN (stick figure) **a** 3D view of REF-IN with surrounding amino acids of 6xbh; **b** 2D view of interaction type of REF-IN with surrounding amino acids of 6xbh

Table S2 shows the molecular docking result of the reference inhibitor and 1000 HIV 1 antiviral compounds on SARS-CoV-2 main protease receptor (PDB ID: 6XBH). The following parameters are shown: number of rotatable torsions, hydrogen bond energy, hydrophobic energy in exposing a surface to water, the van der Waals interaction energy (sum of gc and gh van der Waals), internal conformational energy of the ligand, the desolvation of exposed H-bond donors and acceptors, the solvation electrostatics energy change upon binding and mean force score. According to the molecular docking results, it was found that the binding energy of co-crystallized ligand, (R)-3-(((2R,5S)-5-(((S)-(benzyloxy)(hydroxy)methyl)amino)-1-hydroxy-4-oxo-6-phenylhexan-2-yl) amino)-1,3-dihydro-2H-pyrrol-2-one was − 23.56kCal/mol while the binding energy of all the 1000 HIV 1 antiviral inhibitors lies between − 4.73 and − 48.38 kCal/mol. Figure 1 is the docked poses of SARS CoV-2 main protease (PDB ID: 6XBH) with REF-IN (stick figure) while Table 3 shows the interaction types with surrounding amino acids of SARS CoV 2 Main Protease (PDB ID: 6XBH) with REF-IN.

**REF-IN** is the reference inhibitor with IUPAC name (R)-3-(((2R,5S)-5-(((S)-(benzyloxy) (hydroxy) methyl) amino)-1-hydroxy-4-oxo-6-phenylhexan-2-yl) amino)-1,3-dihydro-2H-pyrrol-2-one.

REF-IN binds at the ligand binding site and forms seven (7) H-bonds with critical amino acids residue in the ligand binding domain site of 6XBH, four (4) of which are conventional H-bonds involving Asn142, Ser144, Gln189, His163 and three carbon-hydrogen bonds between the ligand and Leu141, Glu166 and His163 which are clearly shown in Fig. 1. Other interactions are Π-Pi stacked with Hie41, Π-sulfur interaction with Met165 and alkyl-type interaction with Met49 and Met165 shown in Table 1. The ICM score for the best possible interaction pose was given as − 23.56 kCal/mol.

**Table 1:** Interaction types with surrounding amino acids of SARS CoV 2 Main Protease (PDB ID: 6xbh) with REF-IN

Name	Distance (Å)	Category	Types	From	From Chemistry	To	To Chemistry	Angle DHA º	Angle HAY º
A:ASN142:HN -:RES1:O4	2.299	Hydrogen Bond	Conventional H-Bond	A:ASN142:HN	H-Donor	:RES1:O4	H-Acceptor	104.1	148.7
A:SER144:HG -:RES1:O5	2.624	Hydrogen Bond	Conventional H-Bond	A:SER144:HG	H-Donor	:RES1:O5	H-Acceptor	103.5	94.8
A:GLN189:HE21 -:RES1:N1	2.086	Hydrogen Bond	Conventional H-Bond	A:GLN189:HE21	H-Donor	:RES1:N1	H-Acceptor	113.6	95.6
:RES1:H04 -A:HIS163:NE2	2.291	Hydrogen Bond	Conventional H-Bond	:RES1:H04	H-Donor	A:HIS163:NE2	H-Acceptor	130.0	107.8
A:LEU141:HA -:RES1:O4	2.771	Hydrogen Bond	Carbon H-Bond	A:LEU141:HA	H-Donor	:RES1:O4	H-Acceptor	122.4	133.1
:RES1:H12 -A:GLU166:O	2.521	Hydrogen Bond	Carbon H-Bond	:RES1:H12	H-Donor	A:GLU166:O	H-Acceptor	118.2	146.4
:RES1:H242 -A:HIS163:NE2	2.323	Hydrogen Bond	Carbon H-Bond	:RES1:H242	H-Donor	A:HIS163:NE2	H-Acceptor	120.2	106.2
A:MET165:SD -:RES1	5.527	Other	Π-Sulfur	A:MET165:SD	Sulfur	:RES1	Π-Orbitals		
A:HIE41 -:RES1	4.089	Hydrophobic	Π-Pi Stacked	A:HIE41	Π-Orbitals	:RES1	Π-Orbitals		
:RES1 -A:MET49	5.235	Hydrophobic	Π-Alkyl	:RES1	Π-Orbitals	A:MET49	Alkyl		
:RES1 -A:MET165	4.468	Hydrophobic	Π-Alkyl	:RES1	Π-Orbitals	A:MET165	Alkyl		

The molecular docking score was ranked in order to identify the compounds with the highest inhibitory effects, and easy selection for future studies. The docking studies showed that inhibitors with Index numbers 331, 741, and 819 had the highest binding energies of all the compounds that were docked on SARS CoV 2 main protease (Additional file 1: Table S2). Similarly, inhibitors with Index number 441, 847, and 46 had the highest hydrogen bond energy. Inhibitor with index number 331 was reported with the highest value (− 48.38 kCal/mol) and inhibitor with index number 46 having the least value (− 15.69 kCal/mol) for all the best six (6) selected. The high correlation of H-bonds with the number of flexible bonds (nflex) reflects on the high binding energies of the compounds, with the exception of inhibitor with index number 331 which has six (6) flexible bonds. The low binding energy of inhibitor with index number 331 is unique, its amplified hydrogen bond energy was as a result of an inductive effect created by the presence of three Π-sulfur interactions observed with Cys145, Met165 and Cys145, an amide pi stacked interactions with Thr24 and Thr25, π-lone pair interaction with Thr24, π-pi stacked interaction with Hie41 and π-alkyl interaction involving Met49 and Cys145. Another noticeable point is the bond length of two of the conventional H-bonds involving Thr26 and Hie164 with bond length 1.68 and 1.94A, respectively, thus impacting positively on its activity.

Based on binding energy ranking, inhibitors with Index numbers 331, 741, and 819 were selected for design. Similarly, inhibitors with Index numbers 441, 847, and 46 were selected based on hydrogen bond energy for design. Five new inhibitors labeled a-e were designed for each of the selected inhibitors above. All the compounds in the dataset docked were found to inhibit the receptor by completely occupying the active sites in the target receptor. Most of the inhibitors were tangled in both hydrophobic and hydrogen bonding interactions with the receptor. Here, it was found that strong inhibitor binding is reflected by the frequency of hydrogen bonds. In addition, the molecular docking studies carried out show that all the compounds were found to inhibit the receptors by completely occupying the active sites in the target receptor. The mechanism for this reaction is the same in all cases, which includes the intercalation of the inhibitors between the covalently bonded SARS CoV 2 main protease complex. Additional file 1: Table S3 shows the structures and IUPAC name of designed novel inhibitors while Table 8 shows the molecular docking results. From the table of docking studies, inhibitors with Index numbers 741a, 847b and 741d had the highest binding energies of all the compounds that were docked on SARS CoV 2 main protease. Similarly, inhibitors with Index numbers 847b, and 46d had hydrogen bond energy of − 16.31 kCal/mol and 15.69 kCal/mol, respectively. Inhibitor with index number 741a was reported with the lowest binding energy value of − 45.33 kCal/mol and inhibitor with index number 46d have the least binding energy value of − 34.35 kCal/mol for all the best four (4) selected designed novel inhibitors. The high binding energy of inhibitor with index number 847b is unique, as most of the interaction energies are of H-bond type with amino acids (Thr26, Gly143, Ser144, Cys145, Glu166, Gln189, Hie164, Met49, Thr26, Thr25, Thr190, Asn142, Met165) resulting in an overall negative value. The result could be partly explained by the fact that the inhibitor has nineteen (19) hydrogen bond interaction with the amino acids of the binding pocket of the SARS CoV 2 main protease which is evidenced by the high hydrogen bond energy value of − 16.31 kCal/mol making it the highest of all the newly designed inhibitors. Other noticeable interactions with the receptor include π-alkyl interaction mediated through Cys145. The inhibitor benzyl (5-amino-1-((4-(2-(tert-butyl)-4-oxo-4-(pentan-3-ylamino) butanamido)-3-hydroxy-1-phenylbutan-2-yl)amino) -1,5-dioxopentan-2-yl) carbamate (Index number 847) from which it was designed has binding score energy of − 39.89 kCal/mol and H-bond energy of − 10.27 kCal/mol as against binding score energy and hydrogen bond energy of − 41.32 and − 16.31 kCal/mol, respectively, for the novel inhibitor. The molecular dynamics studies also show that all the hydrogen bonds formed by the compound with index number 847b remain stable after the study.

2-(2-(5-amino-2-((((3-aminobenzyl)oxy)carbonyl) amino)-5-oxopentanamido)-4-(2-(tert-butyl)-4-oxo-4-(pentan-3-ylamino) butanamido)-3-hydroxybutyl) benzoic acid differ significantly in activity from its parent chain because of the introduction of primary amine group attached to meta position of first benzene ring and the carboxyl group attached to the ortho position of the second benzene ring. These groups have the ability to increase the overall binding energy by increasing the number of hydrogen bonds interaction present in their complex. This effects makes 2-(2-(5-amino-2-((((3-aminobenzyl)oxy)carbonyl) amino)-5-oxopentanamido)-4-(2-(tert-butyl)-4-oxo-4-(pentan-3-ylamino)butanamido)-3-hydroxybutyl) benzoic acid a better drug candidate against SARS CoV 2 main protease with the binding energy of -41.32 kCal/mol.

**Fig. 2:**
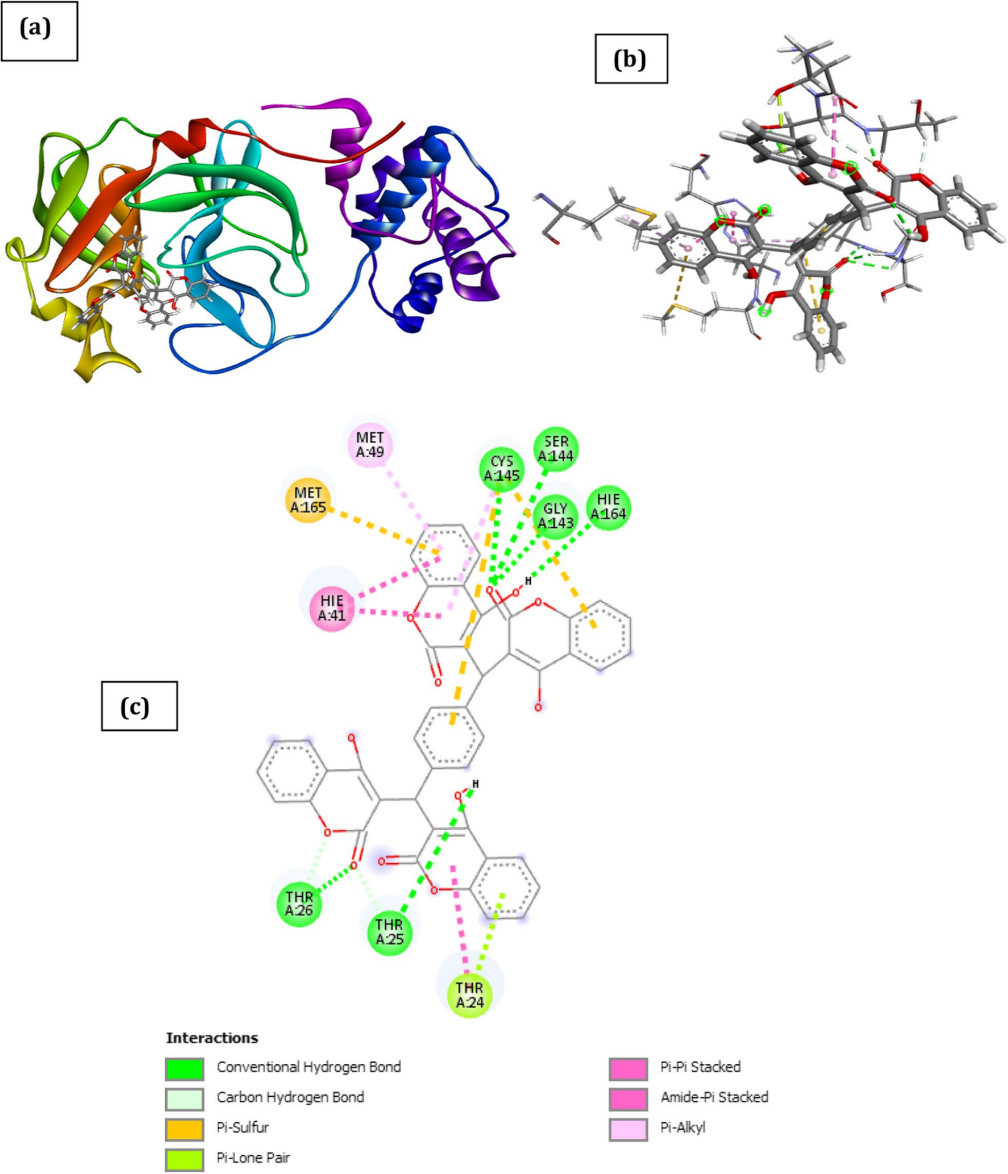
Docked poses of SARS CoV 2 main protease (PDB ID: 6XBH) with inhibitor with Index number 331 (stick figure) **a** 3D view of docked pose of SARS CoV 2 Main Protease (PDB ID: 6XBH) with inhibitor with Index number 331; **b** 3D view of inhibitor with Index number 331 with surrounding amino acids of 6XBH; **c** 2D view of interaction type of inhibitor with Index number 331 with surrounding amino acids of 6XBH

**Fig. 3:**
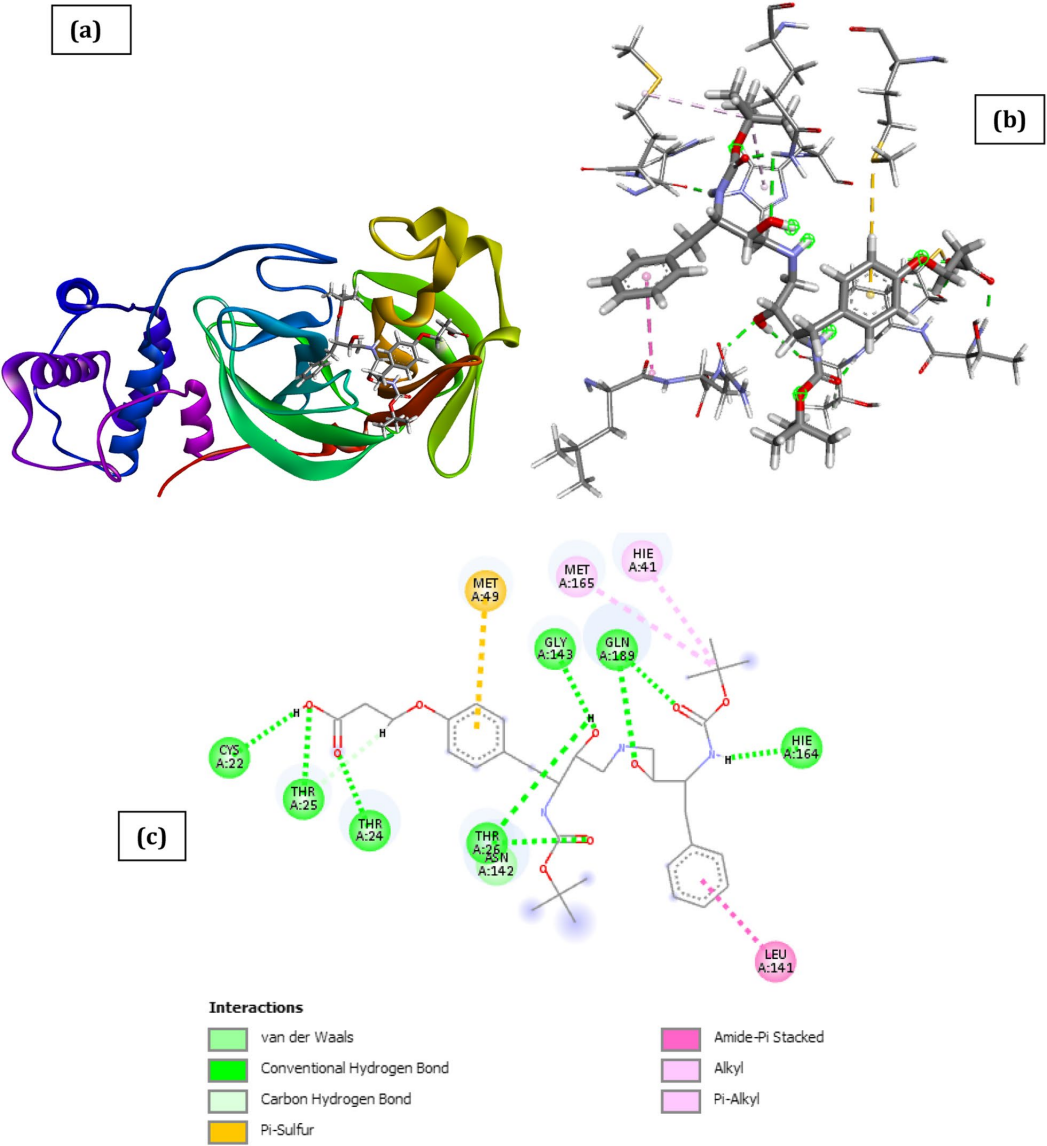
Docked poses of SARS CoV 2 main protease (PDB ID: 6XBH) with inhibitor with Index number 441 (stick figure) **a** 3D view of docked pose of SARS CoV 2 Main Protease (PDB ID: 6XBH) with inhibitor with Index number 441; **b** 3D view of inhibitor with Index number 441 with surrounding amino acids of 6XBH; **c** 2D view of interaction type of inhibitor with Index number 441 with surrounding amino acids of 6XBH

**Fig. 4:**
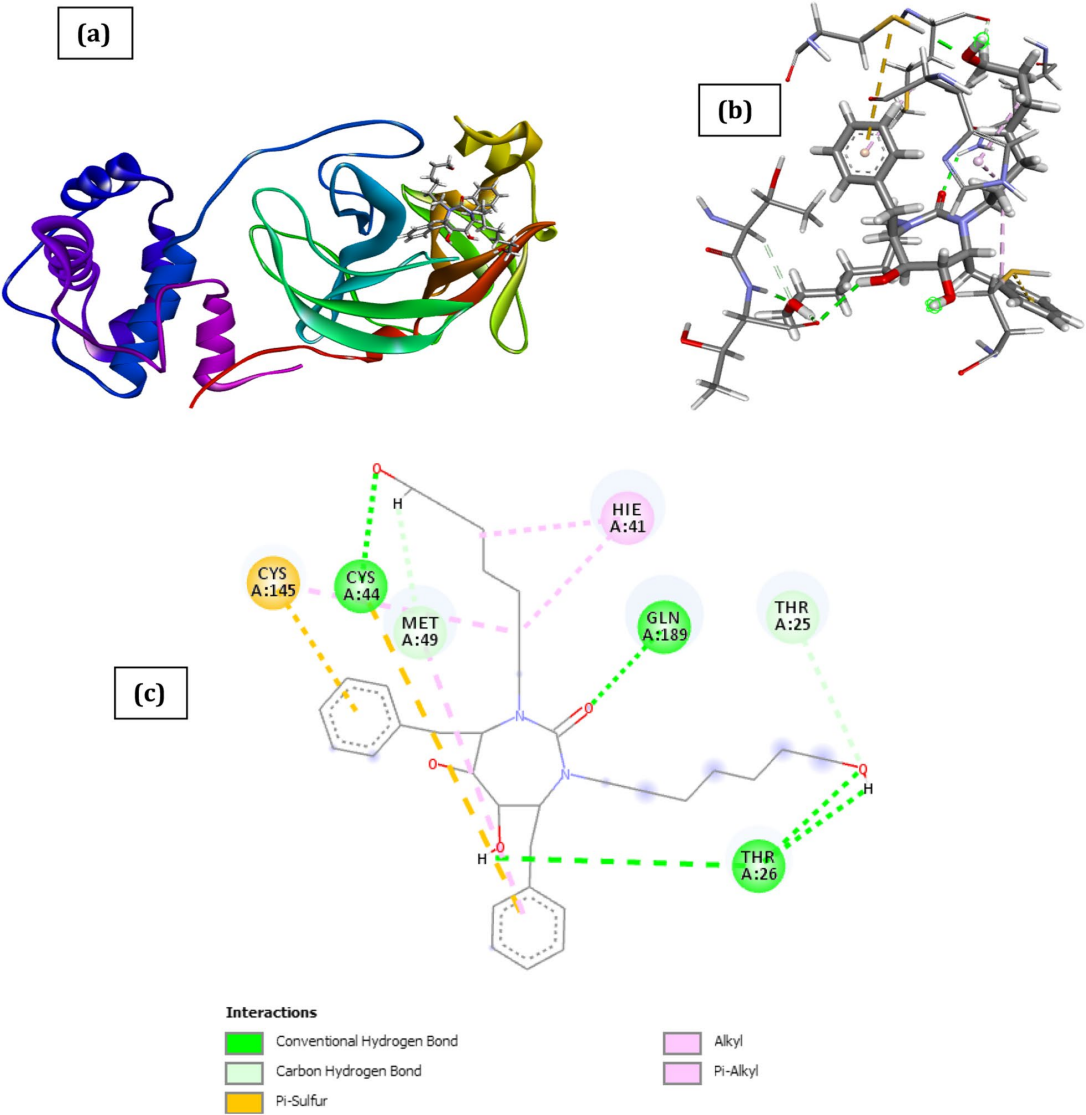
Docked poses of SARS CoV 2 main protease (PDB ID: 6XBH) with inhibitor with Index number 46 (stick figure) **a** 3D view of docked pose of SARS CoV 2 Main Protease (PDB ID: 6XBH) with inhibitor with Index no. 46; **b** 3D view of inhibitor with Index number 46 with surrounding amino acids of 6XBH; **c** 2D view of interaction type of inhibitor with Index number 46 with surrounding amino acids of 6XBH

**Fig. 5:**
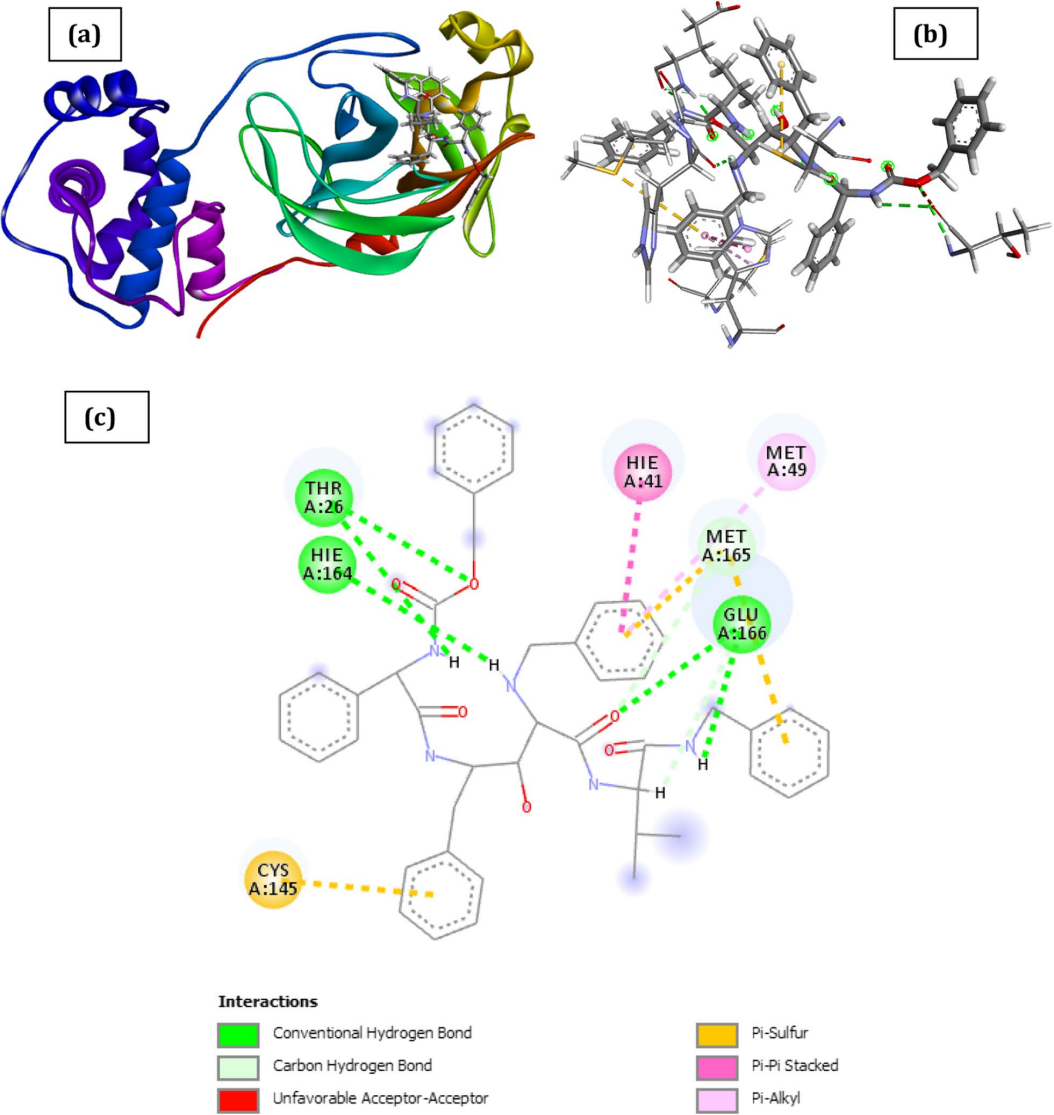
Docked poses of SARS CoV 2 main protease (PDB ID: 6XBH) with inhibitor with Index number 741 (stick figure) **a** 3D view of docked pose of SARS CoV 2 Main Protease (PDB ID: 6XBH) with inhibitor with Index number 741; **b** 3D view of inhibitor with Index number 741 with surrounding amino acids of 6XBH; **c** 2D view of interaction type of inhibitor with Index number 741 with surrounding amino acids of 6XBH

**Fig. 6:**
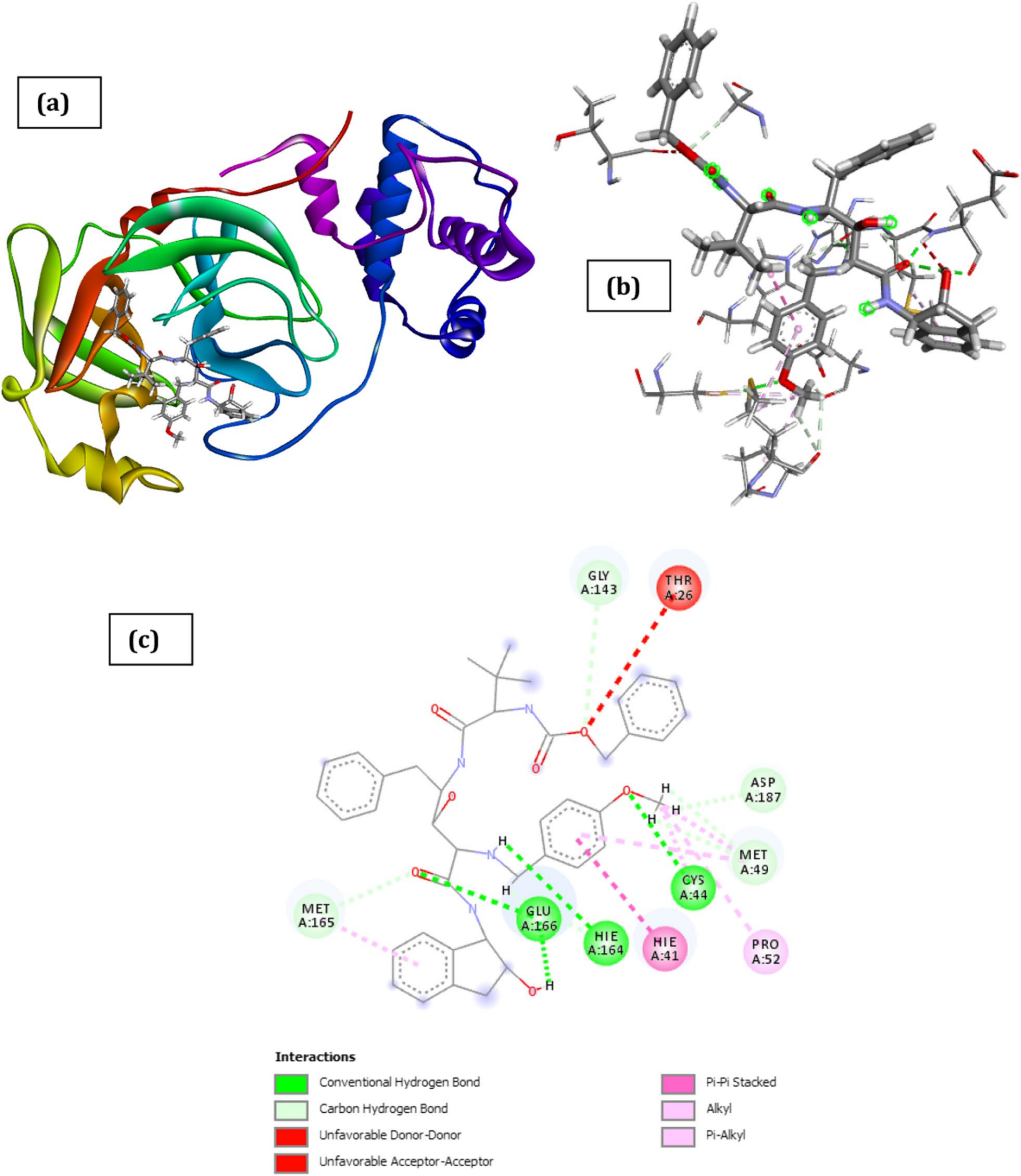
Docked poses of SARS CoV 2 main protease (PDB ID: 6XBH) with inhibitor with Index number 819 (stick figure) **a** 3D view of docked pose of SARS CoV 2 Main Protease (PDB ID: 6XBH) with inhibitor with Index number 819; **b** 3D view of inhibitor with Index number 819 with surrounding amino acids of 6XBH; **c** 2D view of interaction type of inhibitor with Index number 819 with surrounding amino acids of 6XBH

**Fig. 7:**
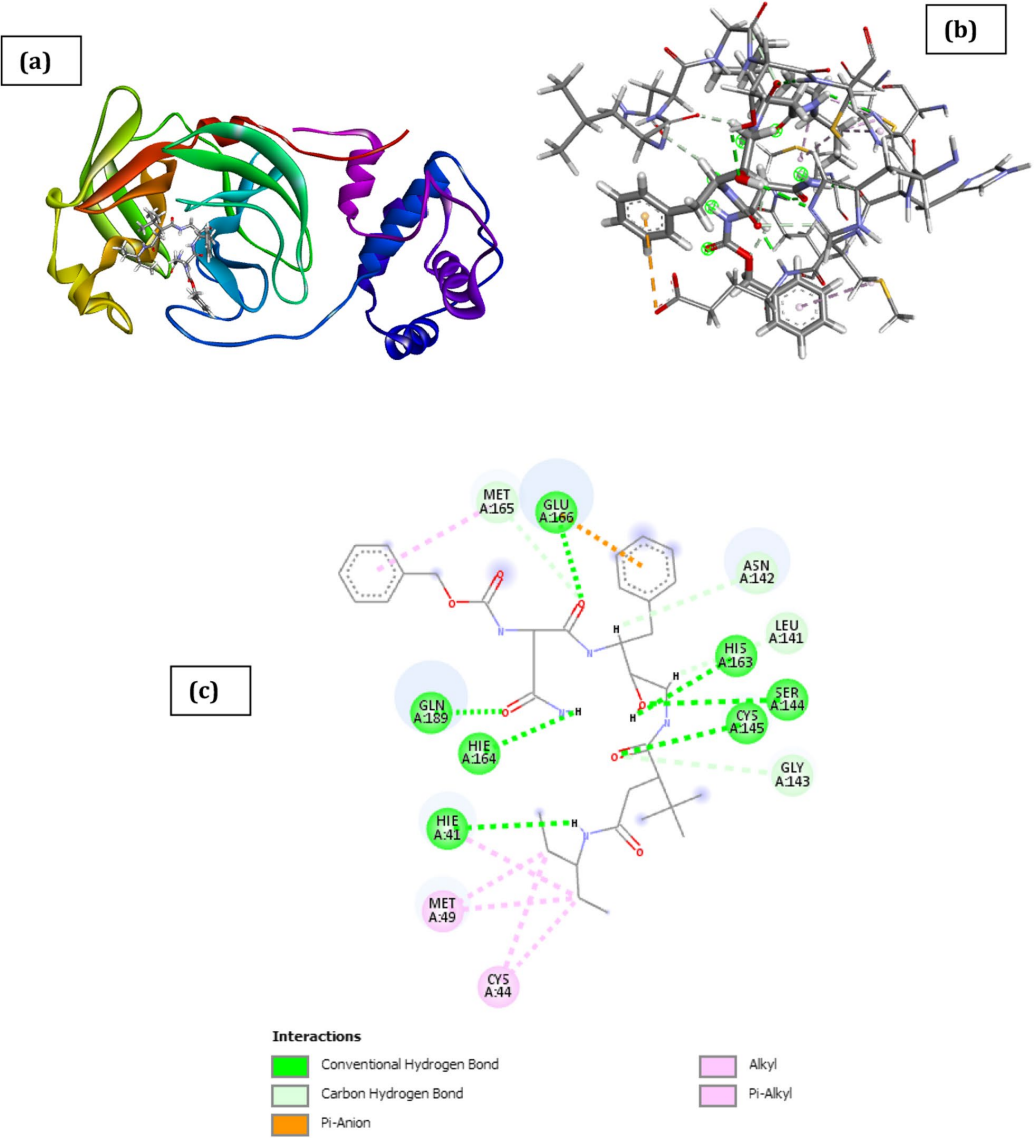
Docked poses of SARS CoV 2 main protease (PDB ID: 6XBH) with inhibitor with Index number 847 (stick figure) **a** 3D view of docked pose of SARS CoV 2 Main Protease (PDB ID: 6XBH) with inhibitor with Index number 847; **b** 3D view of inhibitor with Index number 847 with surrounding amino acids of 6XBH; **c** 2D view of interaction type of inhibitor with Index number 847 with surrounding amino acids of 6XBH

The docked poses of SARS CoV 2 main protease (PDB ID: 6XBH) and inhibitors with Index numbers 331, 441, 46, 741, 819, 847 are presented in Figs. 2, 3,4, 5, 6 and 7, while Tables 2, 3, 4, 5, 6 and 7 show the interaction types with surrounding amino acids of SARS CoV 2 Main Protease (PDB ID: 6XBH) with inhibitor Index numbers 331, 441, 46, 741, 819, 847.

**Fig. 8:**
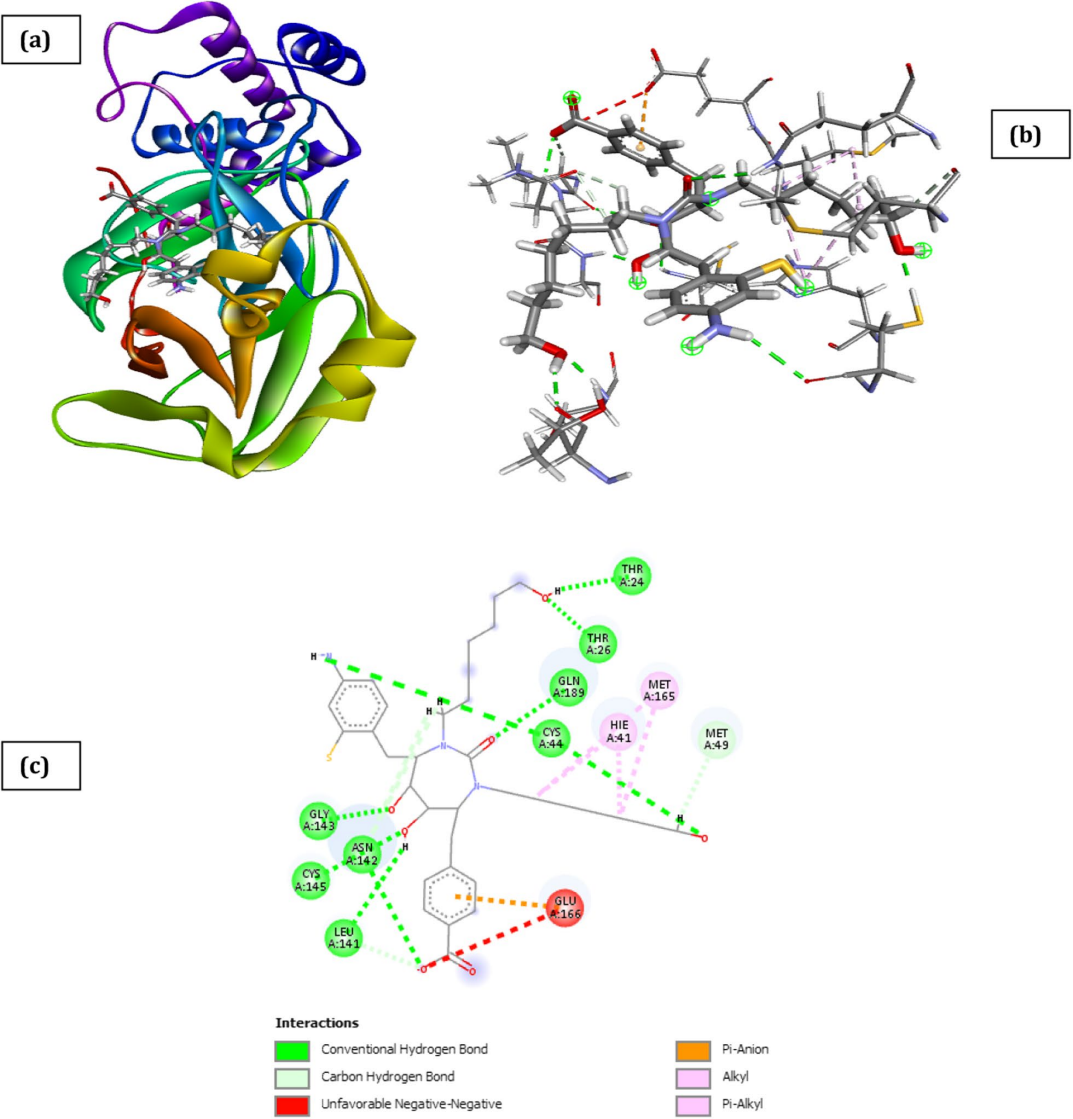
Docked poses of SARS CoV 2 main protease (PDB ID: 6XBH) with inhibitor with Index number 46d (stick figure) **a** 3D view of docked pose of SARS CoV 2 Main Protease (PDB ID: 6XBH) with inhibitor with Index number 46d **b** 3D view of inhibitor with Index number 46d with surrounding amino acids of 6XBH; **c** 2D view of interaction type of inhibitor with Index number 46d with surrounding amino acids of 6XBH

**Fig. 9:**
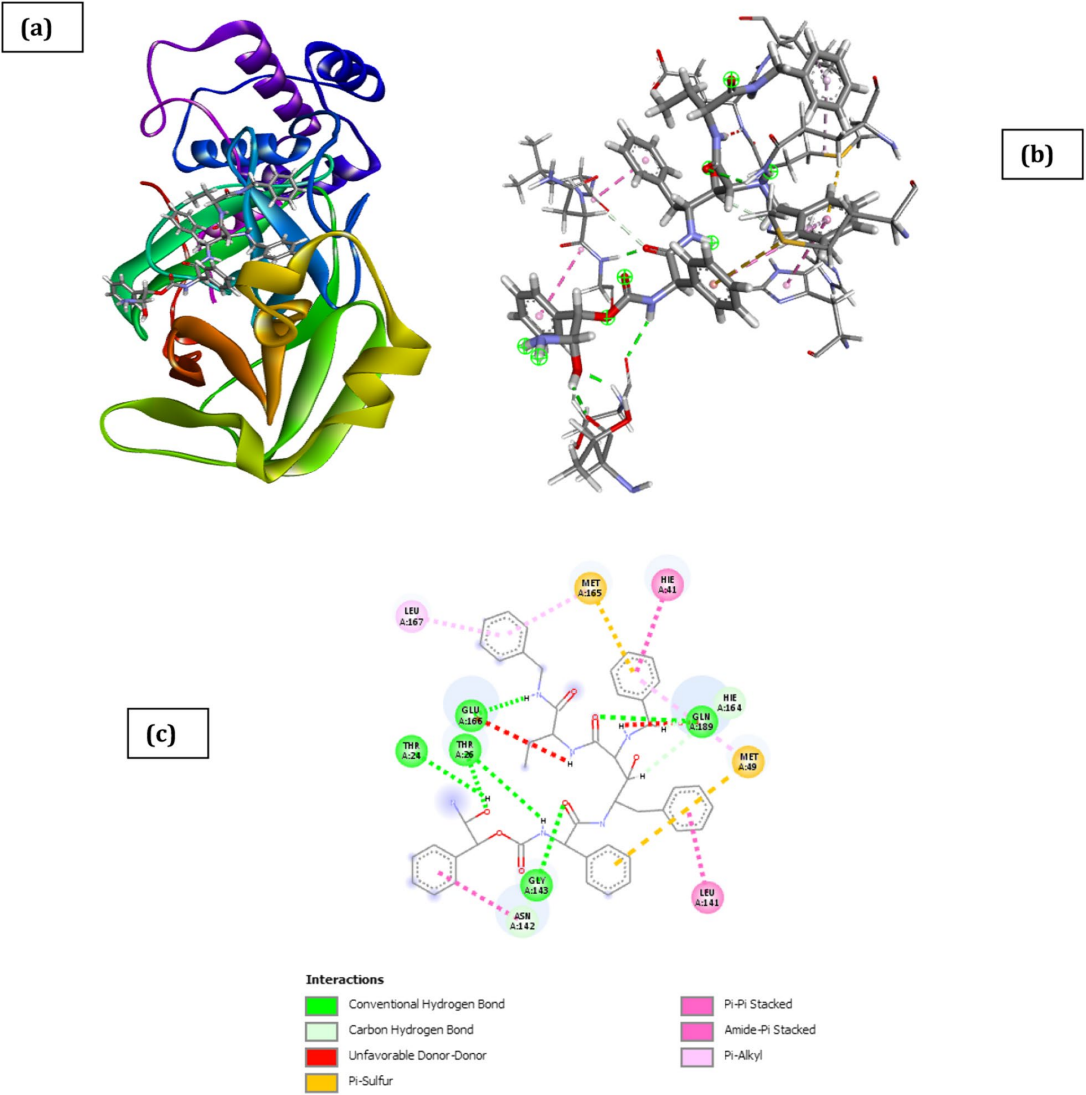
Docked poses of SARS CoV 2 main protease (PDB ID: 6XBH) with inhibitor with Index number 741a (stick figure) **a** 3D view of docked pose of SARS CoV 2 Main Protease (PDB ID: 6XBH) with inhibitor with Index number 741a; **b** 3D view of inhibitor with Index number 741a with surrounding amino acids of 6XBH; **c** 2D view of interaction type of inhibitor with Index number 741a with surrounding amino acids of 6XBH

**Fig. 10:**
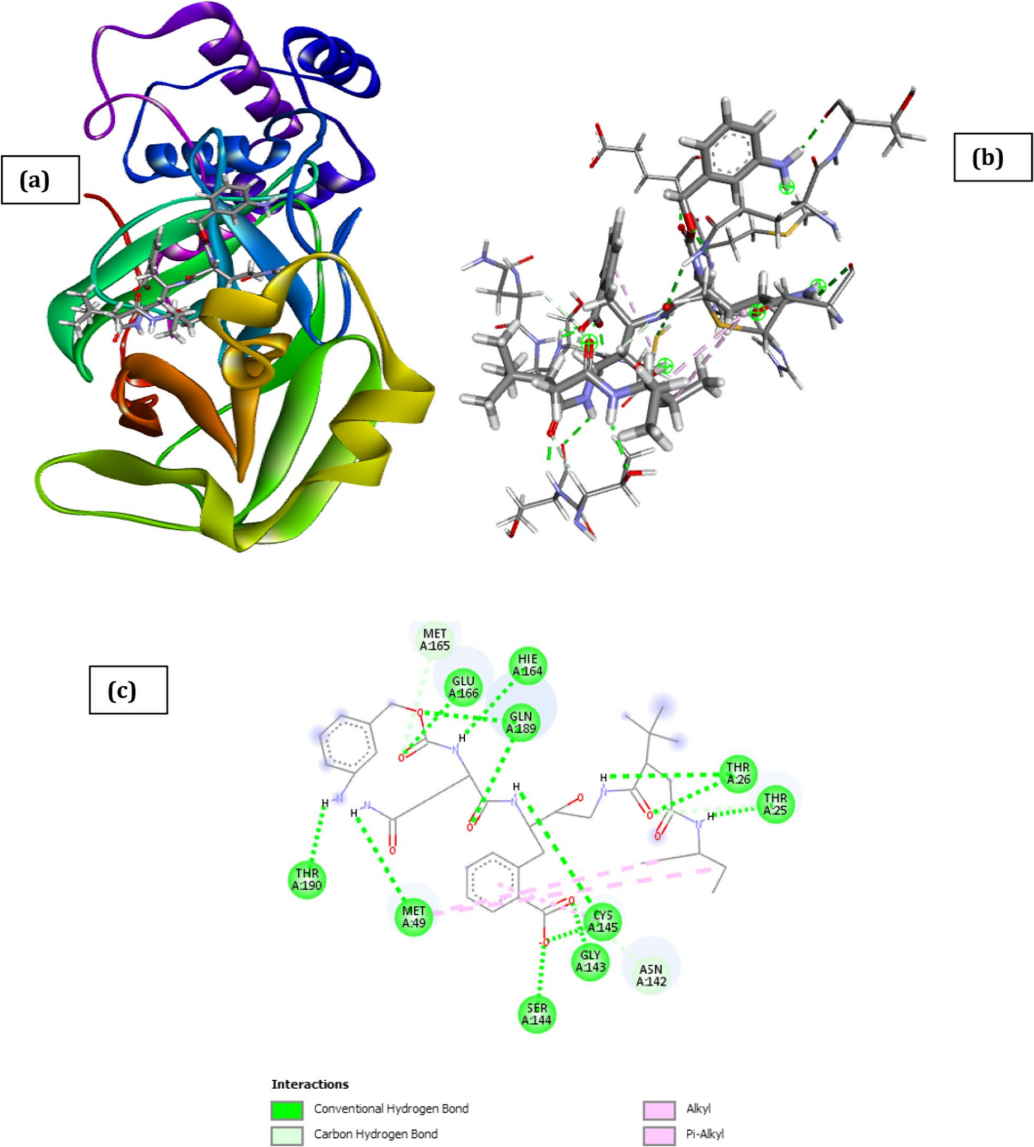
Docked poses of SARS CoV 2 main protease (PDB ID: 6XBH) with inhibitor with Index number 847b (stick figure) **a** 3D view of docked pose of SARS CoV 2 Main Protease (PDB ID: 6XBH) with inhibitor with Index number 847b; **b** 3D view of inhibitor with Index number 847b with surrounding amino acids of 6XBH; **c** 2D view of interaction type of inhibitor with Index number 847b with surrounding amino acids of 6XBH

Table S3 presents the structure and IUPAC name of designed novel inhibitors, while Table 8 presents the molecular docking result of designed novel inhibitors on SARS CoV 2 main protease receptor (PDB ID: 6XBH). The docked poses of SARS CoV 2 main protease (PDB ID: 6XBH) with designed novel inhibitors with Index numbers 46d, 741a, and 847b are presented in Figs. 8, 9 and 10, while Tables 9, 10 and 11 present interaction types with surrounding amino acids of SARS CoV 2 Main Protease (PDB ID: 6XBH) with designed novel inhibitor with Index numbers 46d, 741a and 847b.

## Discussion

The inhibitor with Index number 331 (IUPAC name 3,3'-((4-((4-hydroxy-2-oxo-2H-chromen-3-yl) (2-hydroxy-4-oxo-4H-chromen-3-yl)methyl)phenyl)methylene)bis(4-hydroxy-2H-chromen-2-one)), has the highest ICM score in magnitude, given as -48.38 kCal/mol. This is not a surprise as it has nine (9) hydrogen bonds, comprising of seven (7) conventional H-bonds involving Thr26, Gly143, Ser144, Cys145, Hie164, Thr25, and two (2) Carbon H-bonds involving Thr25 and Thr26. Further enquiry indicates the presence of an amide pi stacked interactions with Thr24 and Thr25, π-lone pair interaction with Thr24, π-pi stacked interaction with Hie41 and π-alkyl interaction involving Met49 and Cys145. Another noticeable point is the bond length of two of the conventional H-bonds involving Thr26 and Hie164 with bond length 1.68 and 1.94A, respectively, thus impacting positively on its activity. This is clearly shown in Fig. 2 and Table 2.

**Table 2:** Interaction types with surrounding amino acids of SARS CoV 2 Main Protease (PDB ID: 6XBH) with inhibitor Index number 331

Name	Distance(Å)	Interaction Types	From	From Chemistry	To	To Chemistry	Angle º DHA	Angle º HAY
A:THR26:HN -:RES1:O12	1.680	Conventional H-Bond	A:THR26:HN	H-Donor	:RES1:O12	H-Acceptor	176.5	128.4
A:GLY143:HN -:RES1:O6	2.071	Conventional H-Bond	A:GLY143:HN	H-Donor	:RES1:O6	H-Acceptor	135.8	125.7
A:SER144:HN -:RES1:O6	2.560	Conventional H-Bond	A:SER144:HN	H-Donor	:RES1:O6	H-Acceptor	116.1	122.9
A:CYS145:HN -:RES1:O6	2.303	Conventional H-Bond	A:CYS145:HN	H-Donor	:RES1:O6	H-Acceptor	155.2	124.3
:RES1:H11 -A:HIE164:O	1.944	Conventional H-Bond	:RES1:H11	H-Donor	A:HIE164:O	H-Acceptor	142.4	139.8
:RES1:H25 -:RES1:O11	2.401	Conventional H-Bond	:RES1:H25	H-Donor	:RES1:O11	H-Acceptor	141.3	127.1
:RES1:H26 -A:THR25:OG1	2.559	Conventional H-Bond	:RES1:H26	H-Donor	A:THR25:OG1	H-Acceptor	167.6	92.4
A:THR25:HA -:RES1:O12	2.695	Carbon H-Bond	A:THR25:HA	H-Donor	:RES1:O12	H-Acceptor	138.6	172.2
A:THR26:HB -:RES1:O7	2.402	Carbon H-Bond	A:THR26:HB	H-Donor	:RES1:O7	H-Acceptor	151.0	116.7
A:CYS145:SG -:RES1	5.972	Π-Sulfur	A:CYS145:SG	Sulfur	:RES1	Π-Orbitals		
A:CYS145:SG -:RES1	5.716	Π-Sulfur	A:CYS145:SG	Sulfur	:RES1	Π-Orbitals		
A:MET165:SD -:RES1	5.788	Π-Sulfur	A:MET165:SD	Sulfur	:RES1	Π-Orbitals		
A:THR24:OG1 -:RES1	2.866	Π-Lone Pair	A:THR24:OG1	Lone Pair	:RES1	Π-Orbitals		
A:HIE41 -:RES1	3.825	Π-Pi Stacked	A:HIE41	Π-Orbitals	:RES1	Π-Orbitals		
A:HIE41 -:RES1	3.524	Π-Pi Stacked	A:HIE41	Π-Orbitals	:RES1	Π-Orbitals		
A:THR24:C,O;THR25:N -:RES1	4.419	Amide-Pi Stacked	A:THR24:C,O;THR25:N	Amide	:RES1	Π-Orbitals		
:RES1 -A:MET49	5.261	Π-Alkyl	:RES1	Π-Orbitals	A:MET49	Alkyl		
:RES1 -A:CYS145	5.463	Π-Alkyl	:RES1	Π-Orbitals	A:CYS145	Alkyl		

The docked structure presented in Fig. 3 and Table 3 showing interaction type of is 3-(4-(2-((tert-butoxycarbonyl) amino)-4-((3-((tert-butoxycarbonyl) amino)-2-hydroxy-4-phenylbutyl) amino)-3-hydroxybutyl) phenoxy) propanoic acid (inhibitor with Index number 441) with SARS CoV 2 main protease shows a negative free energy of binding (-29.01 kCal/mol) implying that binding is feasible as most of the interaction energies are of H-bond type with amino acids (Thr24, Thr25, Thr26, Gly143, Gln189, Hie164, Cys22) resulting in an overall negative value.

**Table 3:** Interaction types with surrounding amino acids of SARS COV 19 Main Protease (PDB ID: 6XBH) with inhibitor Index no. 441

Name	Distance(Å)	Interaction Types	From	From Chemistry	To	To Chemistry	Angle º DHA	Angle º HAY
**A:THR24:HN -:RES1:O9**	1.983	Conventional H-Bond	A:THR24:HN	H-Donor	:RES1:O9	H-Acceptor	161.4	120.5
A:THR25:HG1 -:RES1:O8	2.249	Conventional H-Bond	A:THR25:HG1	H-Donor	:RES1:O8	H-Acceptor	93.5	114.4
A:THR26:HN -:RES1:O4	1.714	Conventional H-Bond	A:THR26:HN	H-Donor	:RES1:O4	H-Acceptor	163.1	131.8
A:GLY143:HN -:RES1:O2	2.137	Conventional H-Bond	A:GLY143:HN	H-Donor	:RES1:O2	H-Acceptor	133.8	107.0
A:GLN189:HE21 -:RES1:O1	2.831	Conventional H-Bond	A:GLN189:HE21	H-Donor	:RES1:O1	H-Acceptor	93.0	108.1
A:GLN189:HE21 -:RES1:O6	1.473	Conventional H-Bond	A:GLN189:HE21	H-Donor	:RES1:O6	H-Acceptor	165.1	151.0
:RES1:H19 -A:THR26:O	2.763	Conventional H-Bond	:RES1:H19	H-Donor	A:THR26:O	H-Acceptor	178.7	154.3
:RES1:H31 -A:HIE164:O	1.927	Conventional H-Bond	:RES1:H31	H-Donor	A:HIE164:O	H-Acceptor	157.2	151.2
:RES1:H49 -A:CYS22:O	2.235	Conventional H-Bond	:RES1:H49	H-Donor	A:CYS22:O	H-Acceptor	138.7	133.0
A:THR26:HB -:RES1:O4	2.859	Carbon H-Bond	A:THR26:HB	H-Donor	:RES1:O4	H-Acceptor	118.1	131.4
:RES1:H46 -A:THR25:OG1	2.342	Carbon H-Bond	:RES1:H46	H-Donor	A:THR25:OG1	H-Acceptor	119.2	109.5
A:MET49:SD -:RES1	5.685	Π-Sulfur	A:MET49:SD	Sulfur	:RES1	Π-Orbitals		
A:LEU141:C,O;ASN142:N -:RES1	4.176	Amide-Pi Stacked	A:LEU141:C,O;ASN142:N	Amide	:RES1	Π-Orbitals		
:RES1:C22 -A:MET165	4.705	Alkyl	:RES1:C22	Alkyl	A:MET165	Alkyl		
A:HIE41 -:RES1:C22	4.433	Π-Alkyl	A:HIE41	Π-Orbitals	:RES1:C22	Alkyl		

The result could be partly explained by the fact that the inhibitor has a strong hydrogen bond interaction with the amino acids of the binding pocket of the SARS CoV 2 main protease which is evidenced by the high hydrogen bond energy value of − 11.45 kCal/mol making it the highest of all the docked inhibitors. Other noticeable interactions with the receptor include carbon–hydrogen interaction with Thr26 and Thr25, π-sulfur interaction with Met49, amide-pi Stacked interaction with Leu141 and Asn142, π-alkyl interaction with Hie41. Zhijian Xu et al. in their work used both MM/GBSA and SIE methods and they voted for nelfinavir, with the binding free energy of − 24.69 ± 0.52 kCal/mol and − 9.42 ± 0.04 kCal/mol, respectively, to be a potential inhibitor against 2019- nCov Mpro (Xu et al. 2020). The inhibiting capacity of their proposed drug is not comparable to that obtained with the compounds with index numbers 441 and 741, making it a better drug candidate than nelfinavir.

The result for compound with index numbers 441 is shown in Fig. 4, the binding energy is reported in Additional file 1: Table S2 to be − 15.67 kCal/mol and the interaction type result is as shown in Table 4. The docked result shows that the inhibitor has seven hydrogen bond interactions with five amino acids (Thr26, Cys44, Gln189, Thr25, and Met49). The binding energy of inhibitor with Index number 741 is determined to be − 47.88 kCal/mol. This makes it the 2nd most active chemical agent with the ability to inhibit SARS CoV 2 main protease (Additional file 1: Table S2). The docked result owes its binding affinity to the presence of seven H-bond with the amino acids which include Thr26, Glu166, Hie164, and Met165 (Fig. 5). Other interactions such as Π-sulfur-type with Cys145, Met165, π-pi stacked with Hie41 and π-alkyl with Met49 are also observed (Table 5).

**Table 4:** Interaction types with surrounding amino acids of SARS CoV 2 Main Protease (PDB ID: 6XBH) with inhibitor Index number 46

Name	Distance(Å)	Interaction Types	From	From Chemistry	To	To Chemistry	Angle º DHA	Angle º HAY
A:THR26:HN -:RES1:O5	1.620	Conventional H-Bond	A:THR26:HN	H-Donor	:RES1:O5	H-Acceptor	168.2	106.2
A:CYS44:HG -:RES1:O4	1.923	Conventional H-Bond	A:CYS44:HG	H-Donor	:RES1:O4	H-Acceptor	156.7	92.5
A:GLN189:HE22 -:RES1:O1	2.273	Conventional H-Bond	A:GLN189:HE22	H-Donor	:RES1:O1	H-Acceptor	130.2	155.0
:RES1:H16 -A:THR26:O	2.131	Conventional H-Bond	:RES1:H16	H-Donor	A:THR26:O	H-Acceptor	122.6	126.1
:RES1:H9 -A:THR26:O	2.638	Conventional H-Bond	:RES1:H9	H-Donor	A:THR26:O	H-Acceptor	109.4	147.5
A:THR25:HA -:RES1:O5	2.926	Carbon H-Bond	A:THR25:HA	H-Donor	:RES1:O5	H-Acceptor	133.6	116.9
:RES1:H19 -A:MET49:O	2.452	Carbon H-Bond	:RES1:H19	H-Donor	A:MET49:O	H-Acceptor	157.3	118.8
A:CYS44:SG -:RES1	5.917	Π-Sulfur	A:CYS44:SG	Sulfur	:RES1	Π-Orbitals		
A:CYS145:SG -:RES1	5.193	Π-Sulfur	A:CYS145:SG	Sulfur	:RES1	Π-Orbitals		
A:CYS145 -:RES1	5.159	Alkyl	A:CYS145	Alkyl	:RES1	Alkyl		
A:HIE41 -:RES1	3.784	Π-Alkyl	A:HIE41	Π-Orbitals	:RES1	Alkyl		
A:HIE41 -:RES1	4.411	Π-Alkyl	A:HIE41	Π-Orbitals	:RES1	Alkyl		
:RES1 -A:MET49	4.817	Π-Alkyl	:RES1	Π-Orbitals	A:MET49	Alkyl

**Table 5:** Interaction types with surrounding amino acids of SARS CoV 2 Main Protease (PDB ID: 6XBH) with inhibitor Index number 741

Name	Distance(Å)	Interaction Types	From	From Chemistry	To	To Chemistry	Angle DHA º	Angle HAY º
A:THR26:HN -:RES1:O1	2.848	Conventional H-Bond	A:THR26:HN	H-Donor	:RES1:O1	H-Acceptor	148.7	95.7
A:GLU166:HN -:RES1:O5	1.687	Conventional H-Bond	A:GLU166:HN	H-Donor	:RES1:O5	H-Acceptor	169.4	159.2
:RES1:H11 -A:GLU166:O	2.089	Conventional H-Bond	:RES1:H11	H-Donor	A:GLU166:O	H-Acceptor	142.2	145.3
:RES1:H22 -A:HIE164:O	2.505	Conventional H-Bond	:RES1:H22	H-Donor	A:HIE164:O	H-Acceptor	146.1	133.0
:RES1:H3 -A:THR26:O	2.852	Conventional H-Bond	:RES1:H3	H-Donor	A:THR26:O	H-Acceptor	134.8	136.5
A:MET165:HA -:RES1:O5	2.750	Carbon H-Bond	A:MET165:HA	H-Donor	:RES1:O5	H-Acceptor	113.7	123.2
:RES1:H10 -A:GLU166:O	2.674	Carbon H-Bond	:RES1:H10	H-Donor	A:GLU166:O	H-Acceptor	146.3	121.9
A:CYS145:SG -:RES1	5.282	Π-Sulfur	A:CYS145:SG	Sulfur	:RES1	Π-Orbitals		
A:MET165:SD -:RES1	4.377	Π-Sulfur	A:MET165:SD	Sulfur	:RES1	Π-Orbitals		
A:MET165:SD -:RES1	5.480	Π-Sulfur	A:MET165:SD	Sulfur	:RES1	Π-Orbitals		
A:HIE41 -:RES1	4.171	Π- Π Stacked	A:HIE41	Π-Orbitals	:RES1	Π-Orbitals		
:RES1 -A:MET49	5.242	Π-Alkyl	:RES1	Π-Orbitals	A:MET49	Alkyl		

Based on hydrogen bond energy ranking, it occupies the third position, and it has hydrogen bond energy of 9.57 kCal/mol. Other important interactions such as π-alkyl, π-sulfur interactions are also reported. This is far better than all the compounds obtained by Motiwale and colleagues (Motiwale et al. 2020). They applied molecular docking approach in conjugation with molecular dynamics (MD) simulations to find out potential inhibitors against Mpro of SARS CoV-2 from previously reported SARS-3CL protease inhibitors.

They used a total of 61, previously known inhibitors. According to the molecular docking results, it was found that the binding energy of co-crystallized ligand, JFM (*N*-(2-phenylethyl)- methanesulfonamide**)** was found to be − 5.1 kCal/mol while the binding energy of all the 61 inhibitors lies between − 6.2 and − 8.8 kCal/mol. Where, 4-{[(4*Z*)-1-(3-chlorophenyl)-5-oxo-3-phenyl-4,5-dihydro-1*H*-pyrazol-4-ylidene]-methyl}benzoic acid, and 4-(4-methoxyphenyl)-6-oxo-2-[(2-phenylethyl)sulfanyl]-1,6-dihydropyrimidine-5-carbonitrile were reported to have minimum and maximum binding energy, respectively. Compounds having a binding energy of − 8.5 kCal/mol or less were considered better agents for the M^pro^. Using this criteria, six molecules namely 4-{[(4*Z*)-1-(3-chlorophenyl)-5-oxo-3-phenyl-4,5-dihydro-1*H*-pyrazol-4-ylidene]methyl}benzoic acid, 5-amino-1-[2-(1-benzothiophen-2-yl)-2-oxoethyl] -2,3-dihydro-1*H*-indole-2,3-dione, *N*-(4-{[(4*Z*)-5-oxo-1,3-diphenyl-4,5-dihydro-1*H*-pyrazol-4-ylidene] methyl} phenyl) acetamide, (4*Z*)-4-{[4-(dimethylamino) phenyl]methylidene}-1,3-diphenyl-4,5-dihydro-1*H*-pyrazol-5-one, 4-{[(4*Z*)-5-oxo-1,3-diphenyl-4,5-dihydro-1*H*-pyrazol-4-ylidene]methyl}benzoic acid, and 4-{[(4*Z*)-1-(4-chlorophenyl)-5-oxo-3-phenyl-4,5-dihydro-1*H*-pyrazol-4-ylidene]methyl}benzoic acid were selected as potential drug candidate (Motiwale et al. 2020).

### SARS CoV 2 main protease (PDB ID: 6XBH) with inhibitor Index number 819

Benzyl (1-((3-hydroxy-5-((2-hydroxy-2,3,3a,7a-tetrahydro-1H-inden-1-yl) amino)-4-((4-methoxybenzyl) amino) -5-oxo-1-phenylpentan-2-yl) amino)-3,3-dimethyl-1-oxobutan-2-yl) carbamate (inhibitor Index number 819) binds at the SARS CoV 2 Main Protease (PDB ID: 6XBH) binding site and forms eleven (11) H-bonds with a critical amino acids residue in the binding domain of 6XBH, five (5) of which are Conventional H-Bonds involving Cys44, Glu166, Hie164, Gly143, Glu166 and six (6) carbon-hydrogen bonds involving Met165, Hie164, Met49, Asp187 and Met49 which are clearly shown in Fig. 6 and in Table 6. The ICM score for the best possible interaction pose was given as − 47.52 kCal/mol, and that makes it the third most active compound reported. However, there are two notable unfavorable interactions reported. These are unfavorable donor–donor and unfavorable acceptor–acceptor interactions. Other interactions include Π-alkyl interaction with Met49, Met165, Π-Pi stacked interaction with Hie41 and alkyl-type interaction with Cys44, Met49, Pro52.

**Table 6:** Interaction types with surrounding amino acids of SARS CoV 2 Main Protease (PDB ID: 6xbh) with inhibitor Index number 819

Name	Distance(Å)	Interaction Types	From	From Chemistry	To	To Chemistry	Angle º DHA	Angle º HAY
A:CYS44:HG -:RES1:O4	2.247	Conventional H-Bond	A:CYS44:HG	H-Donor	:RES1:O4	H-Acceptor	170.3	117.1
A:GLU166:HN -:RES1:O2	1.609	Conventional H-Bond	A:GLU166:HN	H-Donor	:RES1:O2	H-Acceptor	168.2	155.2
:RES1:H15 -A:HIE164:O	2.332	Conventional H-Bond	:RES1:H15	H-Donor	A:HIE164:O	H-Acceptor	143.5	127.7
:RES1:H34 -A:GLU166:O	1.977	Conventional H-Bond	:RES1:H34	H-Donor	A:GLU166:O	H-Acceptor	140.5	126.8
:RES1:H34 -:RES1:O2	2.008	Conventional H-Bond	:RES1:H34	H-Donor	:RES1:O2	H-Acceptor	124.0	110.0
A:GLY143:HA1 -:RES1:O6	2.696	Carbon H-Bond	A:GLY143:HA1	H-Donor	:RES1:O6	H-Acceptor	123.8	103.6
A:MET165:HA -:RES1:O2	3.007	Carbon H-Bond	A:MET165:HA	H-Donor	:RES1:O2	H-Acceptor	107.9	104.5
:RES1:H17 -A:HIE164:O	2.803	Carbon H-Bond	:RES1:H17	H-Donor	A:HIE164:O	H-Acceptor	118.6	171.6
:RES1:H24 -A:MET49:O	2.877	Carbon H-Bond	:RES1:H24	H-Donor	A:MET49:O	H-Acceptor	99.1	121.4
:RES1:H25 -A:ASP187:O	2.167	Carbon H-Bond	:RES1:H25	H-Donor	A:ASP187:O	H-Acceptor	126.0	96.0
:RES1:H26 -A:MET49:O	2.754	Carbon H-Bond	:RES1:H26	H-Donor	A:MET49:O	H-Acceptor	106.3	141.9
A:HIE41 -:RES1	3.677	Π- Π Stacked	A:HIE41	Π-Orbitals	:RES1	Π-Orbitals		
:RES1:C23 -A:CYS44	4.818	Alkyl	:RES1:C23	Alkyl	A:CYS44	Alkyl		
:RES1:C23 -A:MET49	5.133	Alkyl	:RES1:C23	Alkyl	A:MET49	Alkyl		
:RES1:C23 -A:PRO52	4.138	Alkyl	:RES1:C23	Alkyl	A:PRO52	Alkyl		
:RES1 -A:MET49	4.926	Π-Alkyl	:RES1	Π-Orbitals	A:MET49	Alkyl		
:RES1 -A:MET165	4.782	Π-Alkyl	:RES1	Π-Orbitals	A:MET165	Alkyl		

### SARS CoV 2 main protease (PDB ID: 6XBH) with inhibitor index number 847

As presented in Additional file 1: Table S2, the binding energy of this interaction is reported to be − 39.89 kCal/mol. This is the inhibitor with the highest number of hydrogen bond making it one of the best chemical agents with the ability to inhibit SARS CoV 2 main protease. The docked result owes its binding affinity to the presence of thirteen (13) H-bond with the amino acids Ser144, Cys145, Glu166, Gln189, His163, Hie41, Hie164, Gly143, Met165, Asn142, Leu141 as shown in Fig. 7 and confirmed in detail in Table 7. The inter-atomic distances for the H-bond are 2.514, 2.57, 1.866, 1.574, 2.402, 2.649, 2.2, 3.069, 2.676, 2.722, and 2.247 Å, respectively.

**Table 7:** Interaction types with surrounding amino acids of SARS CoV 2 Main Protease (PDB ID: 6XBH) with inhibitor Index number 847

Name	Distance(Å)	Interaction Types	From	From Chemistry	To	To Chemistry	Angle º DHA	Angle HAY º
A:SER144:HG -:RES1:O4	2.514	Conventional H-Bond	A:SER144:HG	H-Donor	:RES1:O4	H-Acceptor	108.8	93.5
A:CYS145:HN -:RES1:O5	2.570	Conventional H-Bond	A:CYS145:HN	H-Donor	:RES1:O5	H-Acceptor	161.8	137.2
A:GLU166:HN -:RES1:O3	1.866	Conventional H-Bond	A:GLU166:HN	H-Donor	:RES1:O3	H-Acceptor	127.0	137.9
A:GLN189:HE21 -:RES1:O7	1.574	Conventional H-Bond	A:GLN189:HE21	H-Donor	:RES1:O7	H-Acceptor	164.8	118.5
:RES1:H24 -A:HIS163:NE2	2.402	Conventional H-Bond	:RES1:H24	H-Donor	A:HIS163:NE2	H-Acceptor	172.3	100.4
:RES1:H29 -A:HIE41:ND1	2.649	Conventional H-Bond	:RES1:H29	H-Donor	A:HIE41:ND1	H-Acceptor	123.3	92.9
:RES1:H50 -A:HIE164:O	2.200	Conventional H-Bond	:RES1:H50	H-Donor	A:HIE164:O	H-Acceptor	121.3	151.2
A:GLY143:HA1 -:RES1:O5	3.069	Carbon H-Bond	A:GLY143:HA1	H-Donor	:RES1:O5	H-Acceptor	99.2	122.1
A:MET165:HA -:RES1:O3	2.676	Carbon H-Bond	A:MET165:HA	H-Donor	:RES1:O3	H-Acceptor	127.9	125.4
:RES1:H11 -A:ASN142:OD1	2.722	Carbon H-Bond	:RES1:H11	H-Donor	A:ASN142:OD1	H-Acceptor	144.8	133.3
:RES1:H12 -:RES1:O3	2.347	Carbon H-Bond	:RES1:H12	H-Donor	:RES1:O3	H-Acceptor	119.0	90.2
:RES1:H13 -A:LEU141:O	2.247	Carbon H-Bond	:RES1:H13	H-Donor	A:LEU141:O	H-Acceptor	145.8	113.8
:RES1:H9 -:RES1:O1	2.098	Carbon H-Bond	:RES1:H9	H-Donor	:RES1:O1	H-Acceptor	107.3	91.2
A:GLU166:OE1 -:RES1	4.471	Π-Anion	A:GLU166:OE1	Negative	:RES1	Π-Orbitals		
:RES1:C31 -A:CYS44	4.628	Alkyl	:RES1:C31	Alkyl	A:CYS44	Alkyl		
:RES1:C31 -A:MET49	4.519	Alkyl	:RES1:C31	Alkyl	A:MET49	Alkyl		
:RES1:C32 -A:CYS44	4.050	Alkyl	:RES1:C32	Alkyl	A:CYS44	Alkyl		
:RES1:C32 -A:MET49	4.152	Alkyl	:RES1:C32	Alkyl	A:MET49	Alkyl		
A:HIE41 -:RES1:C32	4.043	Π-Alkyl	A:HIE41	Π-Orbitals	:RES1:C32	Alkyl		
:RES1 -A:MET165	4.626	Π-Alkyl	:RES1	Π-Orbitals	A:MET165	Alkyl		

Other interactions such as π-anion interaction with Glu166 and π-alkyl interaction with Hie41, Met165 also contributed to the high affinity of the inhibitor in the binding site by stabilizing its structure to conform to the surface of the polar amino acids. From the virtual screening results by Khan and colleague, two drug molecules were selected for each drug target protein [Paritaprevir (ΔG = − 9.8 kCal/mol) &Raltegravir (ΔG = − 7.8 kCal/mol) for 3CLpro and Dolutegravir (ΔG = − 9.4 kCal/mol) and Bictegravir (ΔG = − 8.4 kCal/mol) for 2'-OMTase]. From their extensive computational analysis, they proposed Raltegravir, Paritaprevir, Bictegravir and Dolutegravir as excellent lead candidates for these crucial proteins and they could become potential therapeutic drugs against 2019-nCoV (Khan et al. 2020). This result cannot be compared with our proposed drug that has a binding free energy of − 39.89 kCal/mol.

### SARS CoV 2 main protease (PDB ID: 6XBH) with inhibitor index number 741a

2-amino-2-hydroxy-1-phenylethyl (2-((4-(benzylamino)-5-((1-(benzylamino)-1-oxobutan-2-yl) amino) -3-hydroxy-5-oxo-1-phenylpentan-2-yl)amino)-2-oxo-1-phenylethyl) carbamate binds firmly at the target site of 6XBH with seven Conventional H-Bonds (Thr26, Gly143, Gln189, Glu166, Thr24) and four C-H interaction with Thr26, Asn142, Hie164. The ICM score for the best interaction pose is reported in Table 8 as − 45.33 kcal/mol.

**Table 8:** Molecular docking result of designed inhibitors on SARS CoV 2 main protease receptor (PDB ID: 6XBH)

Index	Binding Energy	Nflex	H-bond	Hphob	VwInt	Eintl	Dsolv	SolEl	mfScore
46a	− 18.5366	24	− 11.104	− 8.08547	− 36.1333	32.1132	31.2896	20.0605	− 79.3598
46b	− 23.6537	25	− 8.29326	− 8.19559	− 41.113	21.5838	28.8813	11.0171	− 74.6023
46c	− 32.2717	24	− 12.8084	− 7.5728	− 38.9914	24.9066	29.7891	11.2983	− 31.6793
46d	− 34.3518	24	− 15.6902	− 7.06253	− 37.7932	30.4018	34.4839	11.525	− 55.5554
46e	− 21.4314	23	− 12.0182	− 7.54758	− 39.3951	21.8896	34.46	20.8811	− 83.4363
331a	− 37.6649	6	− 7.6348	− 7.60136	− 50.2086	22.022	32.5754	13.5167	− 107.326
331b	− 29.1238	7	− 4.39542	− 8.6488	− 53.1397	24.7549	33.746	17.3054	− 122.159
331c	− 36.8913	7	− 4.76964	− 7.82109	− 53.4324	17.8368	30.1628	10.6459	− 121.819
331d	− 35.5207	8	− 6.1511	− 8.27298	− 55.1179	24.8203	33.2654	16.596	− 135.635
331e	− 35.9797	7	− 8.03762	− 6.85719	− 51.4947	24.2922	38.5594	11.0983	− 118.761
441a	− 26.9181	22	− .41735	− 8.07898	− 41.5193	21.2575	27.7305	11.079	− 77.2422
441b	− 15.799	23	− 8.65041	− 8.30234	− 39.2935	23.0035	33.1055	18.6568	− 33.448
441c	− 32.5423	22	− 9.13326	− 7.79642	− 39.328	20.9304	27.5653	1.88249	− 61.9512
441d	− 20.9648	21	− -6.59808	− 7.30528	− 35.1109	16.4746	22.6386	9.32569	− 29.6152
441e	− 12.513	22	− 5.68332	− 7.05838	− 43.2093	29.4196	30.6283	20.656	− 82.4897
741a	− 45.3297	24	− 6.29661	− 10.4884	− 65.8965	32.0133	23.1536	17.8169	− 88.4696
741b	− 27.896	25	− 3.24516	− 10.2221	− 59.3859	26.9172	29.3925	14.4008	− 65.8882
741c	− 26.3067	24	− 5.62273	− 9.48531	− 58.4721	23.4983	31.9674	21.7735	− 105.67
741d	− 40.1221	20	− 4.72178	− 7.82943	− 56.6013	23.0709	24.99	4.43712	− 70.7136
741e	− 39.96	20	− 3.21161	− 10.5506	− 64.6257	29.932	25.3152	13.4448	− 128.321
819a	− 31.0462	19	− 8.32726	− 8.35527	− 54.9642	35.6823	30.142	25.1174	− 95.9107
819b	− 27.1278	20	− 4.92637	− 9.22775	− 52.7082	37.9844	27.9969	17.0136	− 116.119
819c	− 25.965	19	− 7.21995	− 8.16025	− 50.8068	18.7796	24.638	28.329	− 102.438
819d	− 32.6312	20	− 5.29234	− 10.0566	− 50.9015	38.1114	19.7349	17.5708	− 75.6605
819e	− 27.3056	20	− 4.84095	− 9.80879	− 49.769	35.7886	23.566	17.5885	− 67.5418
847a	− 27.5722	22	− 7.106	− 7.66575	− 50.6212	32.3605	26.9864	19.278	− 88.7127
847b	− 41.3159	23	− 16.3125	− 7.4723	− 45.5976	35.595	33.3591	17.787	− 73.3745
847c	− 35.3557	22	− 9.5154	− 7.95671	− 50.7538	24.0559	27.9374	16.1365	− 123.373
847d	− 39.6517	22	− 13.3167	− 7.19533	− 46.3986	13.4494	30.7941	13.5811	− 90.9053
847e	− 28.9414	23	− 11.2521	− 8.22348	− 47.9086	24.1596	33.8423	20.8528	− 79.5009

Nflex:- Number of rotatable torsions

H-bond:- hydrogen bond energy

Hphob:- hydrophobic energy in exposing a surface to water

Vwint:- The van der Waals interaction energy (sum of gc and gh van der Waals)

Eintl:- Internal conformational energy of the ligand

Dsolv:- The desolvation of exposed H-bond donors and acceptors

SolEl:- The solvation electrostatics energy change upon binding

This reported result for the interaction of 2-amino-2-hydroxy-1-phenylethyl (2-((4-(benzylamino)-5-((1-(benzylamino)-1-oxobutan-2-yl)amino)-3-hydroxy-5-oxo-1-phenylpentan-2-yl)amino)-2-oxo-1-phenylethyl) carbamate in the binding site of 6XBH in Table 9 is attributed to the large number of π-interactions such as π-pi interaction with Hie41, π-alkyl interaction with Met49, Met165, and Leu167, amide-pi stacked interaction with Leu145, Asn142, Gly143 and finally π-sulfur interaction with (Met49 and Met165). However, there is also an unfavorable donor–donor interaction with 6XBH which was the reason further studies was not carried out on it (Fig. 8).

**Table 9:** Interaction types of surrounding amino acids of SARS CoV 2 Main Protease (PDB ID: 6XBH) with inhibitor with Index number 46d

Name	Distance(Å)	Interaction types	From	From Chemistry	To	To Chemistry	Angle º DHA	Angle º HAY
A:THR26:HN -:RES1:O3	1.816	Conventional H-Bond	A:THR26:HN	H-Donor	:RES1:O3	H-Acceptor	158.9	97.3
A:CYS44:HG -:RES1:O2	1.928	Conventional H-Bond	A:CYS44:HG	H-Donor	:RES1:O2	H-Acceptor	158.3	99.7
A:ASN142:HN -:RES1:O7	2.155	Conventional H-Bond	A:ASN142:HN	H-Donor	:RES1:O7	H-Acceptor	131.1	129.8
A:GLY143:HN -:RES1:O4	1.609	Conventional H-Bond	A:GLY143:HN	H-Donor	:RES1:O4	H-Acceptor	152.0	116.6
A:CYS145:HN -:RES1:O5	2.855	Conventional H-Bond	A:CYS145:HN	H-Donor	:RES1:O5	H-Acceptor	133.5	96.0
A:GLN189:HE22 -:RES1:O1	2.918	Conventional H-Bond	A:GLN189:HE22	H-Donor	:RES1:O1	H-Acceptor	113.0	121.4
:RES1:H02 -A:THR24:O	2.033	Conventional H-Bond	:RES1:H02	H-Donor	A:THR24:O	H-Acceptor	161.0	138.5
:RES1:H03 -A:LEU141:O	2.636	Conventional H-Bond	:RES1:H03	H-Donor	A:LEU141:O	H-Acceptor	158.9	120.1
:RES1:H04 -A:CYS44:O	2.465	Conventional H-Bond	:RES1:H04	H-Donor	A:CYS44:O	H-Acceptor	145.6	118.0
A:LEU141:HA -:RES1:O7	2.677	Carbon H-Bond	A:LEU141:HA	H-Donor	:RES1:O7	H-Acceptor	128.4	126.0
:RES1:H191 -A:MET49:O	2.433	Carbon H-Bond	:RES1:H191	H-Donor	A:MET49:O	H-Acceptor	164.9	122.0
:RES1:H201 -A:ASN142:OD1	2.588	Carbon H-Bond	:RES1:H201	H-Donor	A:ASN142:OD1	H-Acceptor	112.9	117.5
:RES1:H202 -A:ASN142:OD1	2.753	Carbon H-Bond	:RES1:H202	H-Donor	A:ASN142:OD1	H-Acceptor	102.7	93.9
A:GLU166:OE2 -:RES1	4.177	Π-Anion	A:GLU166:OE2	Negative	:RES1	Π-Orbitals		
:RES1 -A:MET165	5.239	Alkyl	:RES1	Alkyl	A:MET165	Alkyl		
:RES1 -A:MET165	5.095	Alkyl	:RES1	Alkyl	A:MET165	Alkyl		
A:HIE41 -:RES1	4.603	Π-Alkyl	A:HIE41	Π-Orbitals	:RES1	Alkyl		
A:HIE41 -:RES1	4.287	Π-Alkyl	A:HIE41	Π-Orbitals	:RES1	Alkyl		

The docked structure presented in Fig. 9 and Table 10 showing interaction type of SARS CoV 2 Main Protease (PDB ID: 6XBH) with 2-(2-(5-amino-2-((((3-aminobenzyl) oxy) carbonyl) amino)-5-oxopentanamido)-4-(2-(tert-butyl)-4-oxo-4-(pentan-3-ylamino) butanamido)-3-hydroxybutyl) benzoic acid (Index number 847b) shows a binding energy of − 41.32 kCal/mol implying that binding is feasible as most of the interaction energies are of H-bond type with amino acids (Thr26, Gly143, Ser144, Cys145, Glu166, Gln189, Hie164, Met49, Thr26, Thr25, Thr190, Asn142, Met165) resulting in an overall negative value. The result could be partly explained by the fact that the inhibitor has nineteen (19) hydrogen bond interaction with the amino acids of the binding pocket of the SARS CoV 2 main protease which is evidenced by the high hydrogen bond energy value of − 16.31 kCal/mol making it the highest of all the newly designed inhibitors.

Other noticeable interactions with the receptor include π-alkyl interaction mediated through Cys145. The inhibitor benzyl (5-amino-1-((4-(2-(tert-butyl)-4-oxo-4-(pentan-3-ylamino) butanamido)-3-hydroxy-1-phenylbutan-2-yl)amino)-1,5-dioxopentan-2-yl)carbamate (Index no. 847) from which it was designed has binding score energy of − 39.89 kCal/mol and H-bond energy of − 10.27 kCal/mol as against binding score energy and H-bond energy of − 41.32 and − 16.31 kCal/mol, respectively, for the novel inhibitor (Table 11). Komatsu et al. in their work, show the binding pose of main protease system of SARS CoV 2 with darunavir, the ligand interacted with Ser46, Met49, Glu166, Val186, Gln189, and Thr190, ritonavir interacted with Cys44, Cys145, Met165, Asp187, Arg188, and Gln189, indinavir interacted with His41, Gly143, Glu166, and Gln189 (Komatsu et al. 2020).

**Table 10:** Interaction types of surrounding amino acids of SARS CoV 2 Main Protease (PDB ID: 6xbh) with inhibitor with Index number 741a

Name	Distance(Å)	Interaction types	From	From Chemistry	To	To Chemistry	Angle º DHA	Angle º HAY
A:THR26:HN -:RES1:O7	1.808	Conventional H-Bond	A:THR26:HN	H-Donor	:RES1:O7	H-Acceptor	149.5	100.6
A:GLY143:HN -:RES1:O3	1.703	Conventional H-Bond	A:GLY143:HN	H-Donor	:RES1:O3	H-Acceptor	169.5	149.3
A:GLN189:HE22 -:RES1:O5	2.567	Conventional H-Bond	A:GLN189:HE22	H-Donor	:RES1:O5	H-Acceptor	94.4	116.5
:RES1:H01 -A:THR26:O	2.326	Conventional H-Bond	:RES1:H01	H-Donor	A:THR26:O	H-Acceptor	146.8	142.8
:RES1:H04 -:RES1:O4	1.932	Conventional H-Bond	:RES1:H04	H-Donor	:RES1:O4	H-Acceptor	118.2	107.5
:RES1:H05 -A:GLU166:O	2.333	Conventional H-Bond	:RES1:H05	H-Donor	A:GLU166:O	H-Acceptor	141.1	154.7
:RES1:H07 -A:THR24:O	2.132	Conventional H-Bond	:RES1:H07	H-Donor	A:THR24:O	H-Acceptor	149.8	147.1
A:THR26:HB -:RES1:O7	2.973	Carbon H-Bond	A:THR26:HB	H-Donor	:RES1:O7	H-Acceptor	113.3	108.8
A:ASN142:HA -:RES1:O3	2.653	Carbon H-Bond	A:ASN142:HA	H-Donor	:RES1:O3	H-Acceptor	130.9	149.7
:RES1:H251 -A:HIE164:O	2.490	Carbon H-Bond	:RES1:H251	H-Donor	A:HIE164:O	H-Acceptor	168.4	123.9
:RES1:H272 -A:HIE164:O	2.314	Carbon H-Bond	:RES1:H272	H-Donor	A:HIE164:O	H-Acceptor	126.5	131.2
A:MET49:SD -:RES1	4.769	Π-Sulfur	A:MET49:SD	Sulfur	:RES1	Π-Orbitals		
A:MET165:SD -:RES1	5.630	Π-Sulfur	A:MET165:SD	Sulfur	:RES1	Π-Orbitals		
A:HIE41 -:RES1	4.402	Π-Pi Stacked	A:HIE41	Π-Orbitals	:RES1	Π-Orbitals		
:RES1 -:RES1	5.794	Π-Pi T-shaped	:RES1	Π-Orbitals	:RES1	Π-Orbitals		
A:LEU141:C,O;ASN142:N -:RES1	3.411	Amide- Π Stacked	A:LEU141:C,O;ASN142:N	Amide	:RES1	Π-Orbitals		
A:ASN142:C,O;GLY143:N -:RES1	4.052	Amide- Π Stacked	A:ASN142:C,O;GLY143:N	Amide	:RES1	Π-Orbitals		
:RES1 -A:MET49	5.048	Π-Alkyl	:RES1	Π-Orbitals	A:MET49	Alkyl		
:RES1 -A:MET165	4.45607	Π-Alkyl	:RES1	Π-Orbitals	A:MET165	Alkyl		
:RES1 -A:LEU167	5.41087	Π-Alkyl	:RES1	Π-Orbitals	A:LEU167	Alkyl

**Table 11:** Interaction types of surrounding amino acids of SARS CoV 2 Main Protease (PDB ID: 6XBH) with inhibitor with Index number 847b

Name	Distance(Å)	Interaction types	From	From Chemistry	To	To Chemistry	Angle DHA º	Angle º HAY
A:THR26:HN -:RES1:O6	1.768	Conventional H-Bond	A:THR26:HN	H-Donor	:RES1:O6	H-Acceptor	144.1	157.2
A:GLY143:HN -:RES1:O8	1.322	Conventional H-Bond	A:GLY143:HN	H-Donor	:RES1:O8	H-Acceptor	157.1	125.6
A:SER144:HN -:RES1:O9	2.080	Conventional H-Bond	A:SER144:HN	H-Donor	:RES1:O9	H-Acceptor	110.0	96.7
A:CYS145:HN -:RES1:O9	1.636	Conventional H-Bond	A:CYS145:HN	H-Donor	:RES1:O9	H-Acceptor	123.0	130.8
A:GLU166:HN -:RES1:O3	1.644	Conventional H-Bond	A:GLU166:HN	H-Donor	:RES1:O3	H-Acceptor	153.0	163.3
A:GLN189:HE21 -:RES1:O1	2.252	Conventional H-Bond	A:GLN189:HE21	H-Donor	:RES1:O1	H-Acceptor	123.8	122.6
A:GLN189:HE22 -:RES1:O4	2.585	Conventional H-Bond	A:GLN189:HE22	H-Donor	:RES1:O4	H-Acceptor	109.6	119.6
:RES1:H01 -A:HIE164:O	2.240	Conventional H-Bond	:RES1:H01	H-Donor	A:HIE164:O	H-Acceptor	162.6	131.3
:RES1:H02 -A:MET49:O	2.749	Conventional H-Bond	:RES1:H02	H-Donor	A:MET49:O	H-Acceptor	149.6	130.8
:RES1:H03 -A:THR26:O	2.362	Conventional H-Bond	:RES1:H03	H-Donor	A:THR26:O	H-Acceptor	125.8	127.0
:RES1:H04 -A:THR25:OG1	2.169	Conventional H-Bond	:RES1:H04	H-Donor	A:THR25:OG1	H-Acceptor	150.5	108.0
:RES1:H31 -A:CYS145:SG	2.944	Conventional H-Bond	:RES1:H31	H-Donor	A:CYS145:SG	H-Acceptor	129.1	116.6
:RES1:H62 -A:THR190:O	2.083	Conventional H-Bond	:RES1:H62	H-Donor	A:THR190:O	H-Acceptor	148.1	156.3
A:THR25:HA -:RES1:O6	2.296	Carbon H-Bond	A:THR25:HA	H-Donor	:RES1:O6	H-Acceptor	138.9	119.2
A:ASN142:HA -:RES1:O8	2.560	Carbon H-Bond	A:ASN142:HA	H-Donor	:RES1:O8	H-Acceptor	123.2	97.2
A:MET165:HA -:RES1:O3	2.286	Carbon H-Bond	A:MET165:HA	H-Donor	:RES1:O3	H-Acceptor	114.8	105.4
:RES1:H161 -:RES1:O4	2.728	Carbon H-Bond	:RES1:H161	H-Donor	:RES1:O4	H-Acceptor	103.8	101.5
:RES1:H171 -:RES1:O8	2.367	Carbon H-Bond	:RES1:H171	H-Donor	:RES1:O8	H-Acceptor	100.5	154.0
:RES1:H172 -:RES1:O8	2.389	Carbon H-Bond	:RES1:H172	H-Donor	:RES1:O8	H-Acceptor	99.07	123.8
:RES1:C29 -A:MET49	4.324	Alkyl	:RES1:C29	Alkyl	A:MET49	Alkyl		
:RES1:C30 -A:MET49	5.212	Alkyl	:RES1:C30	Alkyl	A:MET49	Alkyl		
:RES1 -A:CYS145	5.305	Π-Alkyl	:RES1	Π-Orbitals	A:CYS145	Alkyl		

None of these proposed drugs have as much interactions as 2-(2-(5-amino-2-((((3-aminobenzyl)oxy) carbonyl)amino)-5-oxopentanamido)-4-(2-(tert-butyl)-4-oxo-4-(pentan-3-ylamino) butanamido)-3-hydroxybutyl) benzoic acid (the novel inhibitor). This improvement resulted from the primary amine group attached to meta position of first benzene ring and the carboxyl group attached to the ortho position of the second benzene ring (Fig. 10). This result makes 2-(2-(5-amino-2-((((3-aminobenzyl)oxy) carbonyl) amino)-5-oxopentanamido)-4-(2-(tert-butyl)-4-oxo-4-(pentan-3-ylamino) butanamido)-3-hydroxybutyl) benzoic acid a better drug candidate against SARS CoV-2 main protease in comparison with the co-crystallized inhibitor or any of the 1000 inhibitors.
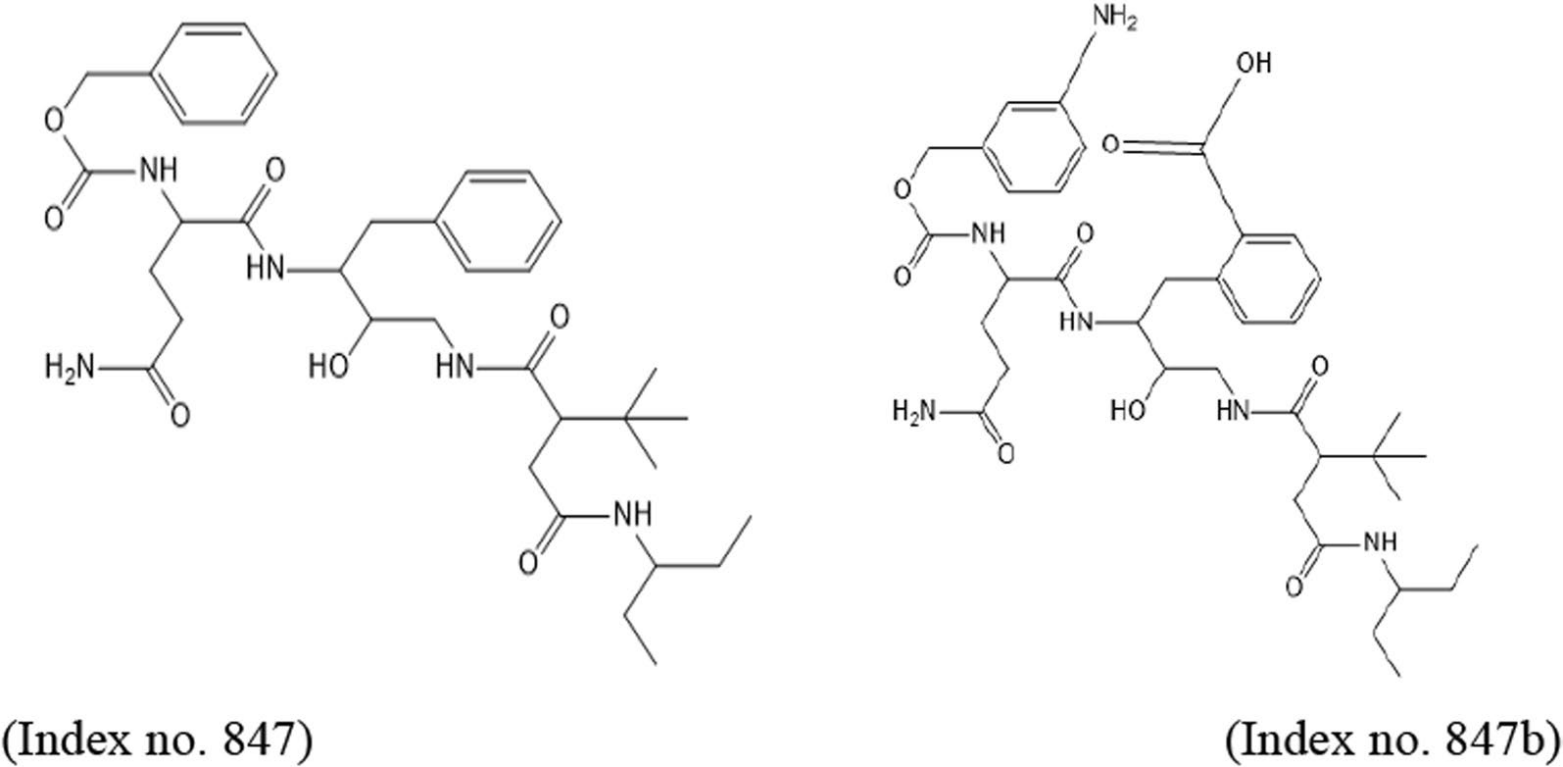


### Molecular dynamics

**Fig. 11:**
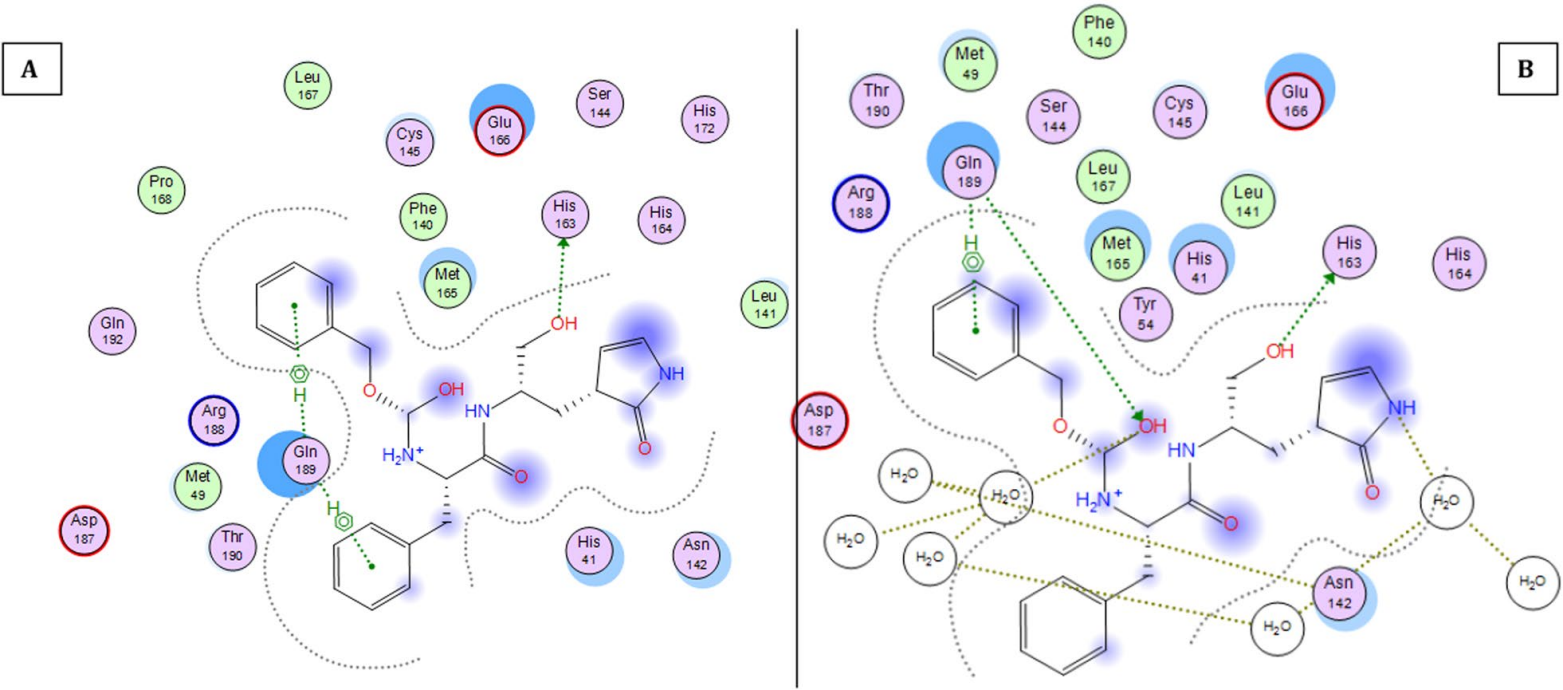
2D interaction of REF-IN **A** Before Molecular dynamics study and **B** After Molecular dynamics analysis

**Fig. 12:**
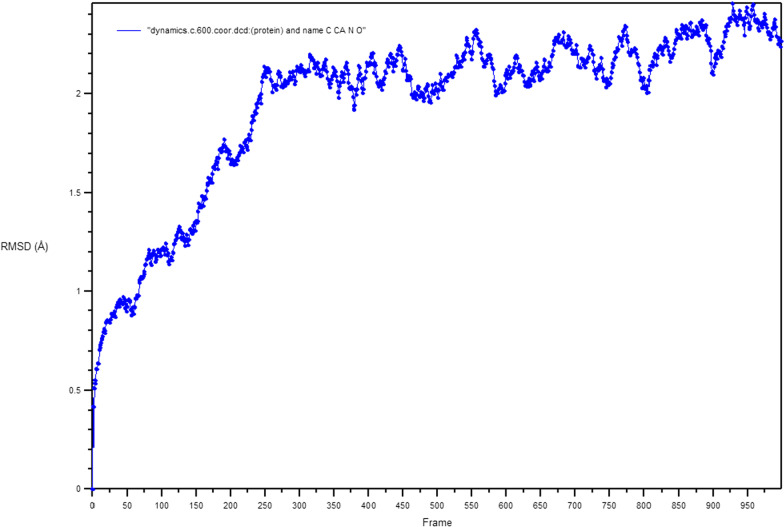
RMSD plot of REF-IN

**Fig. 13:**
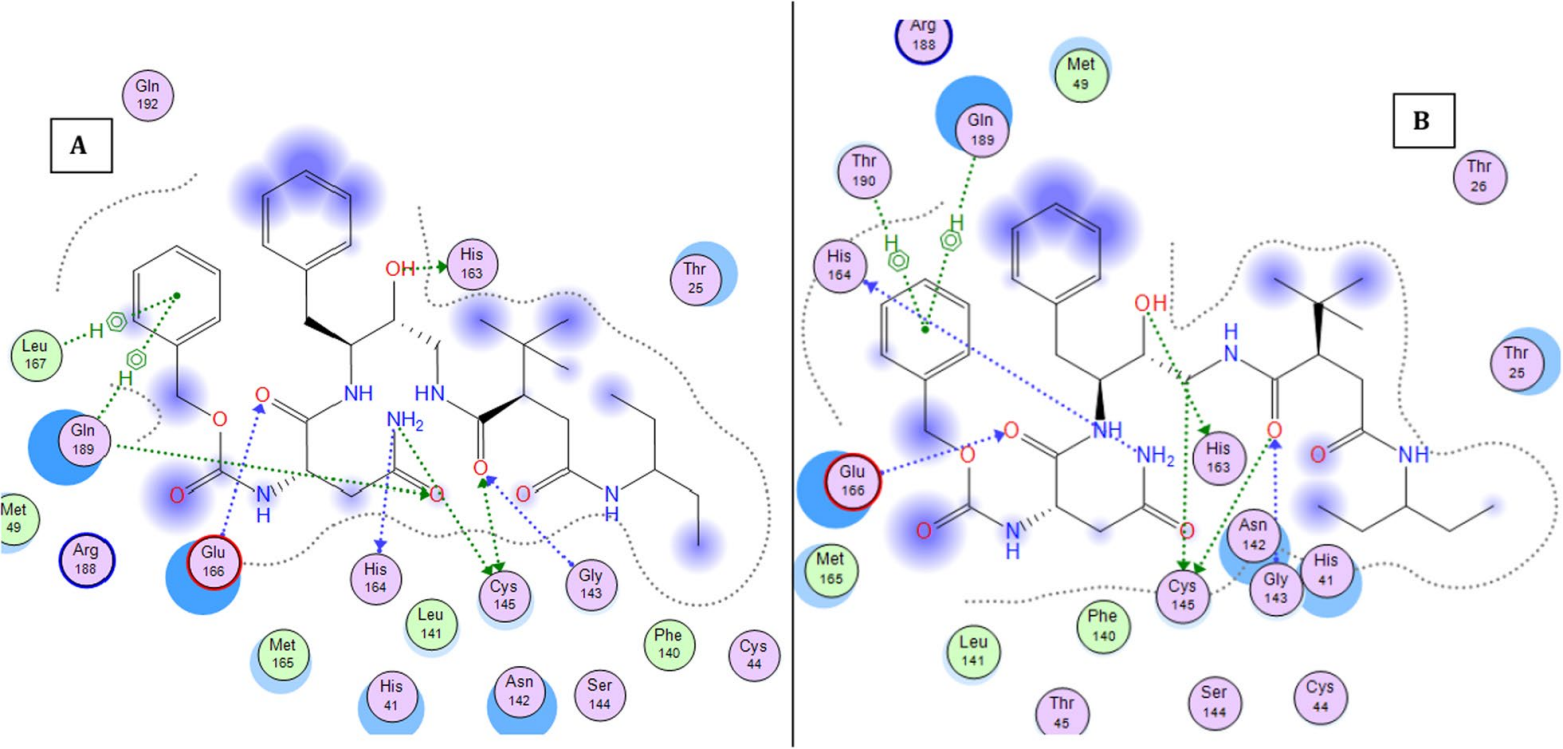
2d interaction of inhibitor with Index number 847 **A** Before Molecular dynamics study and **B** After Molecular dynamics analysis

**Fig. 14:**
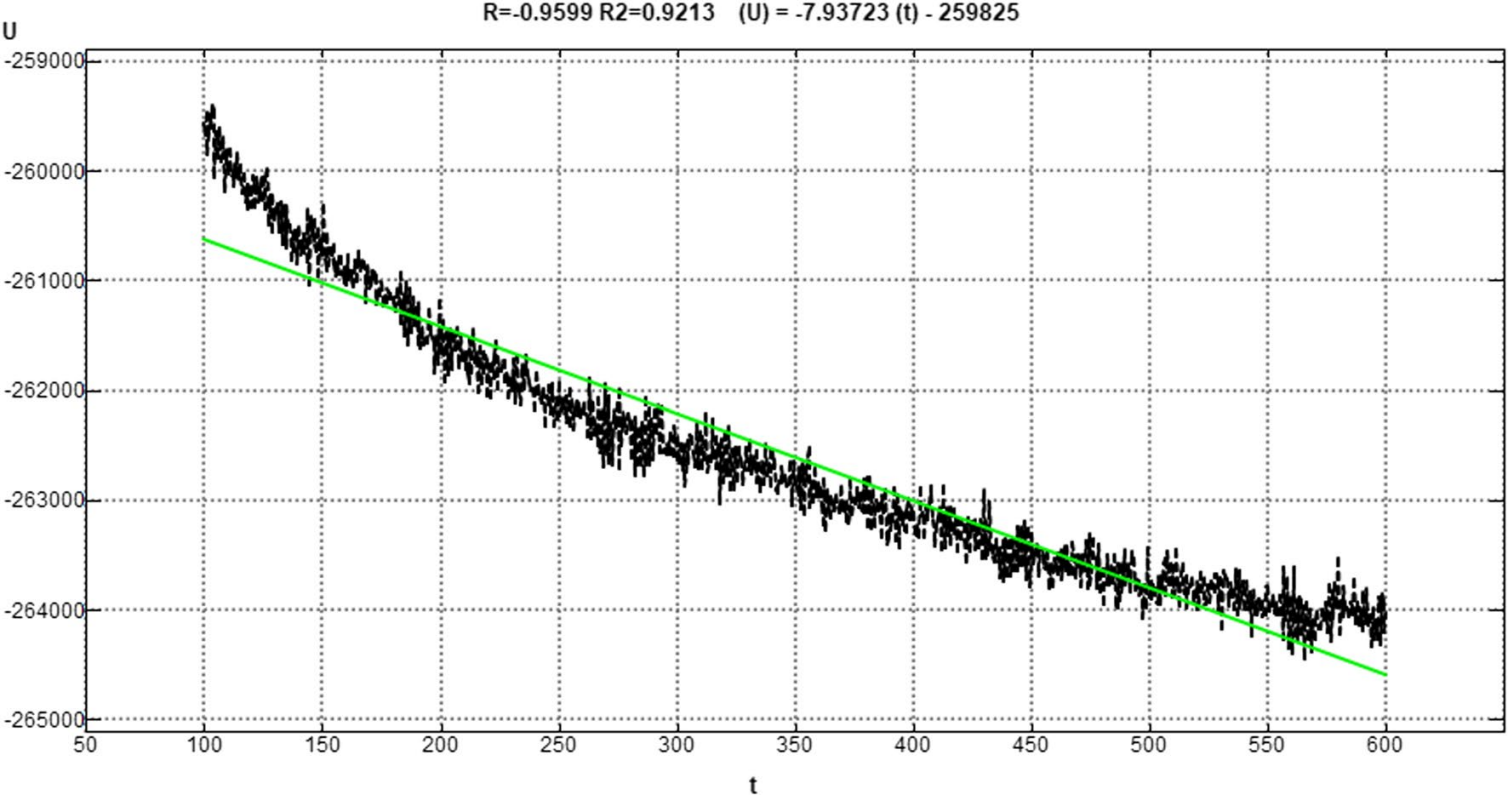
Plot of Internal Energy (*U*) versus time (*t*) for molecular dynamics study of inhibitor with Index number 847

**Fig. 15:**
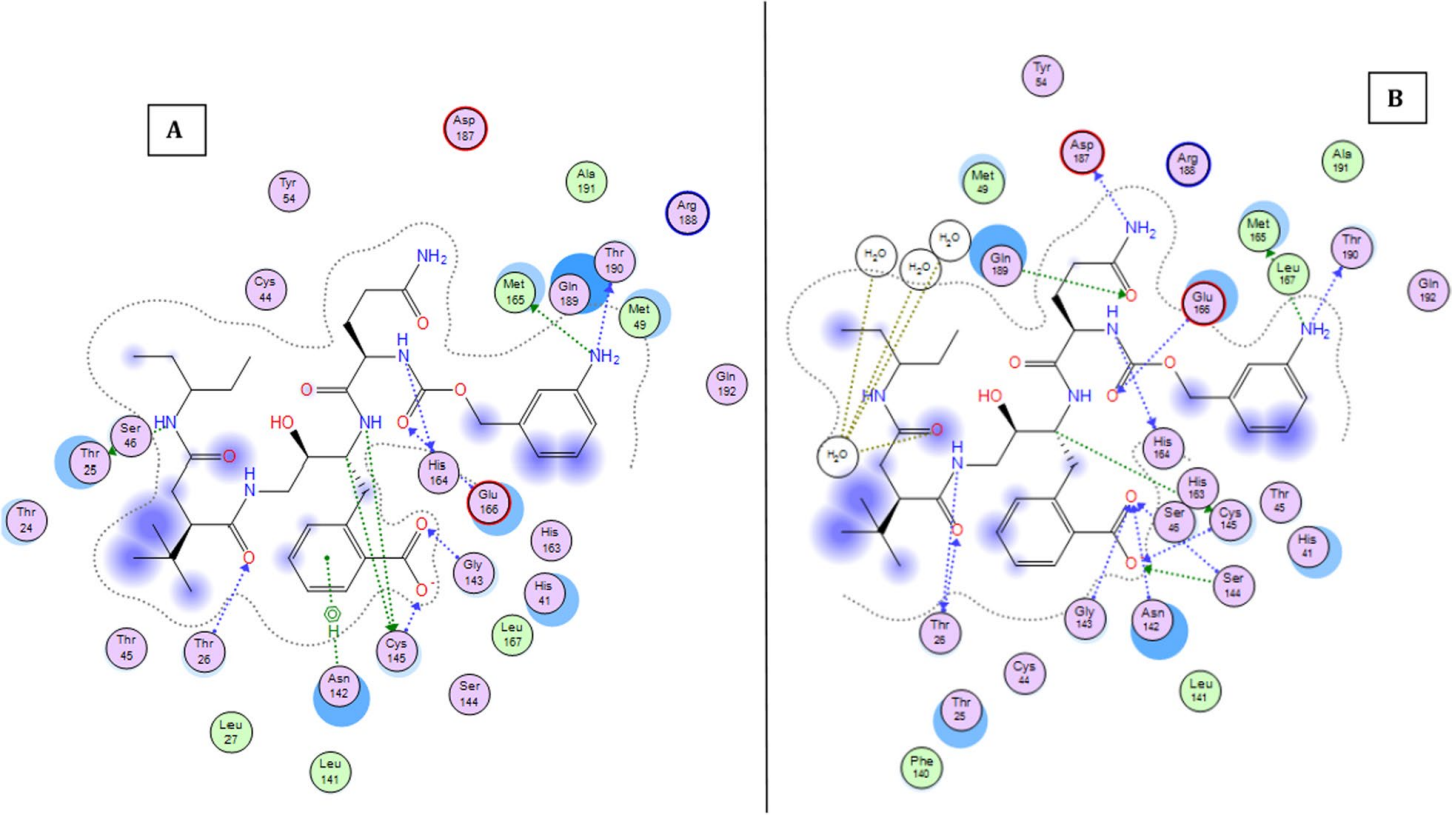
2d interaction of inhibitor with Index number 847b **A** Before Molecular dynamics study and **B** After Molecular dynamics analysis

**Fig. 16:**
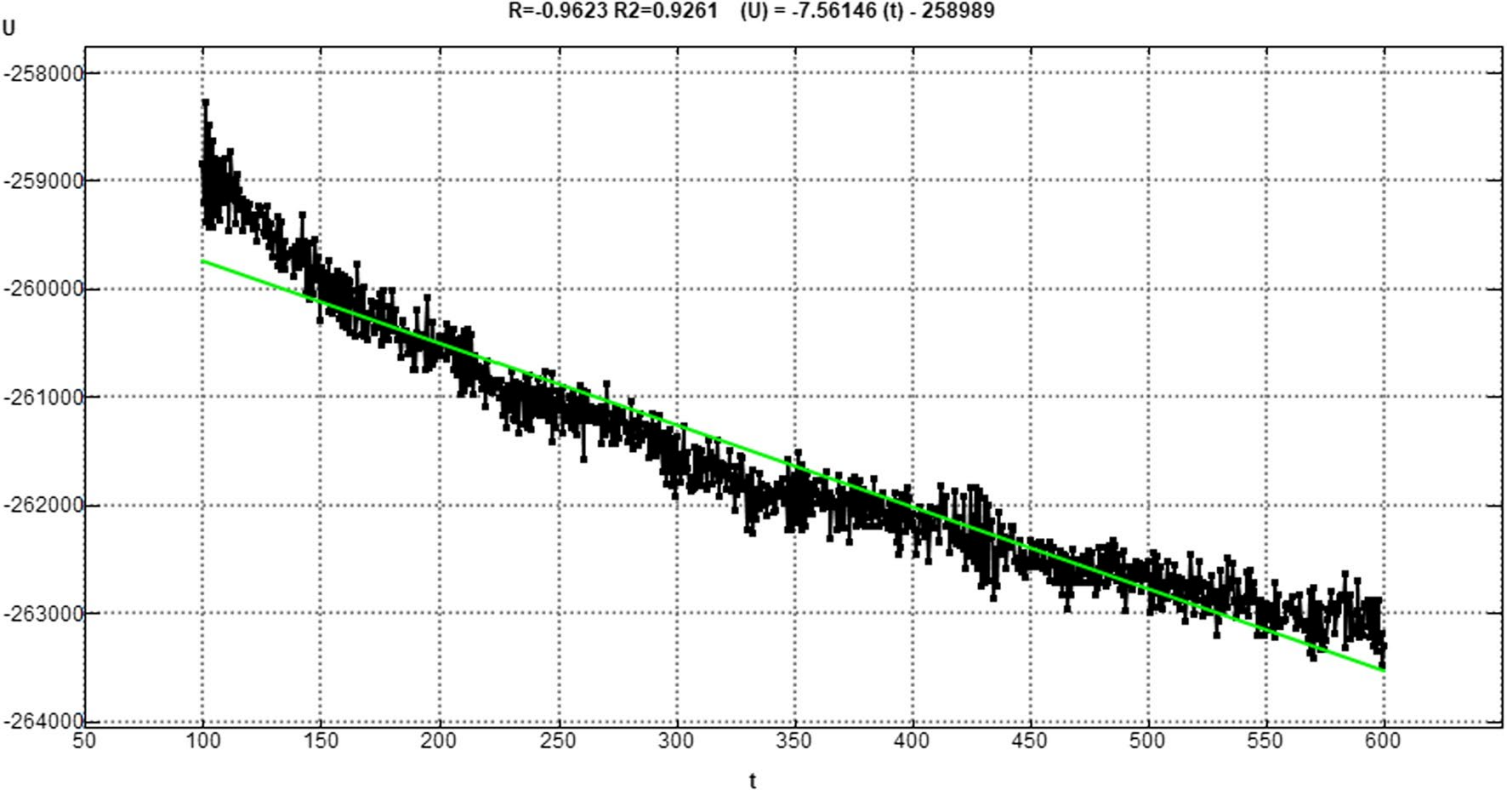
Plot of Internal Energy (*U*) versus time (*t*) for molecular dynamics study of inhibitor with Index number 847b

Figure 11 shows the 2D interaction of REF-IN with the main protease of SARS CoV2 before and after molecular dynamics study, while Figs. 12 shows the RMSD plot. Figures 13 and 14 shows the shows the 2D interaction of inhibitor with Index number 847 with the main protease of SARS CoV 2 before and after molecular dynamics study, a plot of internal energy with time, a plot of potential energy with time, and a plot of enthalpy change with time, respectively. Figures 15 and 16 show the 2D interaction of inhibitor 847b with the main protease of SARS CoV 2 before and after molecular dynamics study, and a plot of internal energy against time.

### Reference inhibitor

#### Interaction of benzyl (5-amino-1-((4-(2-(tert-butyl)-4-oxo-4-(pentan-3-ylamino) butanamido)-3-hydroxy-1-phenylbutan-2-yl) amino)-1,5-dioxopentan-2-yl) carbamate (inhibitor Index number 847) before and after molecular dynamics study

Figure 13 is the 2d interaction of SARS CoV 2 main protease with benzyl (5-amino-1-((4-(2-(tert-butyl)-4-oxo-4-(pentan-3-ylamino) butanamido)-3-hydroxy-1-phenylbutan-2-yl)amino)-1,5-dioxopentan-2-yl)carbamate (inhibitor Index number 847) before and after molecular dynamics study placed side by side. It can be seen that all the interactions remained intact after the dynamics study. This is further proven in Fig. 14 by the constancy of potential energy over time. The internal energy decreased consistently until 600 picoseconds when it stabilizes at − 264,500 kCal/mol.

Interaction of 2-(2-(5-amino-2-((((3-aminobenzyl)oxy) carbonyl) amino)-5-oxopentanamido)-4-(2-(tert-butyl)-4-oxo-4-(pentan-3-ylamino) butanamido)-3-hydroxybutyl) benzoic acid (index number 847b) before and after molecular dynamics study.

Figures 15 and 16 show the 2d interaction of SARS CoV 2 main protease with 2-(2-(5-amino-2-((((3-aminobenzyl)oxy) carbonyl)amino)-5-oxopentanamido)-4-(2-(tert-butyl)-4-oxo-4-(pentan-3-ylamino) butanamido)-3-hydroxybutyl)benzoic acid (the novel inhibitor) before and after molecular dynamics study and the plot of internal energy versus time. As shown in Fig. 16, the internal energy decreases consistently until it stabilizes between 575 and 600 picoseconds. It also shows that the interaction is a spontaneous one in which energy in form of heat is lost, the enthalpy change reduces gradually over time to achieve stability at 600 picoseconds.

## Conclusions

The molecular docking results shown in the figures confirm that the hydrophobic and hydrogen bonding interactions with these targets have pivotal contributions to the binding structures and binding free energies, even though the van der Waals and π-interactions contributed to the stabilization of the binding structures.

The molecular docking result also shows that, inhibitors with Index numbers 331, 741, 819, 441, 847, and 46 with ICM score of − 48.38 kCal/mol, − 47.88 kCal/mol, − 47.52 kCal/mol, 29.01 kCal/mol, 39.89 kCal/mol, and − 15.67 kCal/mol, respectively, best inhibit SARS CoV 2 main protease of the compounds within our data set. These compounds were further utilized in designing new potent inhibitor compounds by attaching potent fragments to the compounds. Most of the newly designed compounds were reported to be more active than the parent structure. This includes compounds with index number 741a, 847b, and 741d with a binding affinity of − 45.33 kCal/mol, − 41.32 kCal/mol and − 40.12 kCal/mol, respectively. However, compounds with index numbers 741a and 741b and 46d were not considered to be our potential drug candidate because of the presence of unfavorable interactions they formed with SARS CoV2 main protease. The fragments responsible for their affinities were primarily carboxylic group and primary amine group. At the end of the study, we were able to computationally design a potent novel compounds that can be used to inhibit SARS CoV 2 main protease. The novel drug is 2-(2-(5-amino-2-((((3-aminobenzyl)oxy) carbonyl) amino)-5-oxopentanamido)-4-(2-(tert-butyl)-4-oxo-4-(pentan-3-ylamino) butanamido)-3-hydroxybutyl) benzoic acid with binding score energy and H-bond energy of − 41.32 and − 16.31 kCal/mol, respectively.

## Supplementary Information

**Additional file 1**. **Supplementary table 1:** Name, target source, article doi, Authors and Zinc ID of the complete dataset. **Supplementary table 2.** Molecular docking result of reference inhibitor and complete dataset on COVID 19 main protease receptor (PDB ID: 6XBH). **Supplementary table 3:** Structure and IUPAC Name of Designed Novel Inhibitors.

## Data Availability

All data generated or analyzed during this study are included in this published article (and its supplementary information files).

## Abbreviations

AMBER: Assisted model building with energy refinement
Å: Armstrong
BFGS: Broyden–Fletcher–Goldfarb–Shanno (BFGS) algorithm
CADD: Computer-aided drug design
CHARMM: Chemistry at HARvard macromolecular mechanics
CG: Conjugate gradient
DNA: Deoxyribonucleic acid
GROMOS: GROningen MOlecular Simulation
GUI: Graphic user interphase
HCV: Hepatitis C virus
HIV: Human immunodeficiency virus
ICM: Internal coordinate mechanics
kCal/mol: Kilo Calorie per mole
LBDD: Ligand-based drug design
L-BFGS: Limited memory Broyden–Fletcher–Goldfarb–Shanno (BFGS) algorithm
LGA: Lamarckian genetic algorithm
MD: Molecular dynamics
MM-GBSA: Molecular mechanics/generalized born surface area
MM: Molecular mechanics
Mpro: Main protease
MT-DTI: Molecule transformer–drug target interaction
MTTK: Martyna–Tuckerman–TobiasKlein
NAMD: NAnoscale molecular dynamics
nCoV: Novel corona virus
NMR: Nuclear magnetic resonance
NVE: Microcanonical ensemble (a system is isolated from changes in moles (N), volume (V), and energy (E))
NVT: Canonical ensemble (amount of substance (N), volume (V) and temperature (T) are conserved)
NS3: Non-structural protein 3
PBC: Periodic boundary conditions
PDB: Protein data bank
PM6: Parameterization method 6
PME: Particle mesh Ewald
QSAR: Quantitative structure–activity relationship
RdRp: RNA-dependent RNA polymerase
RMSD: Root mean square deviation
RNA: Ribonucleic acid
SARS CoV 2: Severe acute respiratory syndrome coronavirus 2
SBDD: Structure-based drug design
SD: Steepest descent
VMD: Visual molecular dynamics
0D, 1D, 2D, 3D: Zero- , one- , two- and three-dimensional descriptor, respectively
2’-O-MTase: 2’-O-ribose methyltransferase
3 CL pro: 3 C-like proteinase
∆G: Free energy of binding
