# Supplementary material for: Computational drug design of novel COVID-19 inhibitor
Source: Bull Natl Res Cent. 2022 Jul 15;46(1):210. doi: 10.1186/s42269-022-00892-z (PMC9284480; doi:10.1186/s42269-022-00892-z)
Supplement: Supplementary file 1 — Additional file 1. Supplementary table 1: Name, target source, article doi, Authors and Zinc ID of the complete dataset. Supplementary table 2. Molecular docking result of reference inhibitor and complete dataset on COVID 19 main protease receptor (PDB ID: 6XBH). Supplementary table 3: Structure and IUPAC Name of Designed Novel Inhibitors. [file 42269_2022_892_MOESM1_ESM.docx]

Supplementary table 1: Name, target source, article doi, Authors and Zinc ID of the complete dataset.

| Index | **Binding bd ligand** | **target source** | **Article doi** | **Authors** | **Zinc ID** |  |
| --- | --- | --- | --- | --- | --- | --- |
| 1 | (4R,5S,6S,7R)-4,7-dibenzyl-5,6-dihydroxy-1,3-bis[(2E)-3-(1H-pyrazol-4-yl)prop-2-en-1-yl]-1,3-diazepan-2-one | HIV 1 | 10.1021/jm9602571 | Lam et al | ZINC03948988 | |
| 2 | (4R,5S,6S,7R)-4,7-dibenzyl-1-(cyclopropylmethyl)-5,6-dihydroxy-3-[(2E)-3-(1H-pyrazol-4-yl)prop-2-en-1-yl]-1,3-diazepan-2-one | HIV 1 | 10.1021/jm9602571 | Lam et al | ZINC03826079 | |
| 3 | (4R,5S,6S,7R)-4,7-dibenzyl-1-(cyclopropylmethyl)-5,6-dihydroxy-3-(6-hydroxyhexyl)-1,3-diazepan-2-one | HIV 1 | 10.1021/jm9602571 | Lam et al | ZINC14907367 | |
| 4 | (4R,5S,6S,7R)-4,7-dibenzyl-1-(cyclopropylmethyl)-5,6-dihydroxy-3-(5-hydroxypentyl)-1,3-diazepan-2-one | HIV 1 | 10.1021/jm9602571 | Lam et al | ZINC14907369 | |
| 5 | (4R,5S,6S,7R)-4,7-dibenzyl-1-butyl-3-(cyclopropylmethyl)-5,6-dihydroxy-1,3-diazepan-2-one | HIV 1 | 10.1021/jm9602571 | Lam et al | ZINC14907371 | |
| 6 | (4R,5S,6S,7R)-4,7-dibenzyl-1,3-bis(cyclobutylmethyl)-5,6-dihydroxy-1,3-diazepan-2-one | HIV 1 | 10.1021/jm9602571 | Lam et al | ZINC13604438 | |
| 7 | (4R,5S,6S,7R)-4,7-dibenzyl-5,6-dihydroxy-1,3-bis(5-hydroxypentyl)-1,3-diazepan-2-one | HIV 1 | 10.1021/jm9602571 | Lam et al | ZINC14907373 | |
| 8 | (4R,5S,6S,7R)-4,7-dibenzyl-1,3-dibutyl-5,6-dihydroxy-1,3-diazepan-2-one | HIV 1 | 10.1021/jm9602571 | Lam et al | ZINC13604416 | |
| 9 | (4R,5S,6S,7R)-4,7-dibenzyl-5,6-dihydroxy-1,3-bis(3-methylbut-2-en-1-yl)-1,3-diazepan-2-one | HIV 1 | 10.1021/jm9602571 | Lam et al | ZINC13604434 | |
| 10 | (4R,5S,6S,7R)-4,7-dibenzyl-1-butyl-5,6-dihydroxy-3-(3-methylbut-2-en-1-yl)-1,3-diazepan-2-one | HIV 1 | 10.1021/jm9602571 | Lam et al | ZINC14907375 | |
| 11 | (4R,5S,6S,7R)-4,7-dibenzyl-1,3-bis(cyclopropylmethyl)-5,6-dihydroxy-1,3-diazepan-2-one | HIV 1 | 10.1021/jm9602571 | Lam et al | ZINC03810793 | |
| 12 | (4R,5S,6S,7R)-4,7-dibenzyl-5,6-dihydroxy-1,3-bis(6-hydroxyhexyl)-1,3-diazepan-2-one | HIV 1 | 10.1021/jm9602571 | Lam et al | ZINC14907377 | |
| 13 | (4R,5S,6S,7R)-4,7-dibenzyl-1-(cyclopropylmethyl)-5,6-dihydroxy-3-(3-methylbut-2-en-1-yl)-1,3-diazepan-2-one | HIV 1 | 10.1021/jm9602571 | Lam et al | ZINC14907379 | |
| 14 | (4R,5S,6S,7R)-4,7-dibenzyl-1,3-bis(6-bromo-6-hydroxyhexyl)-5,6-dihydroxy-1,3-diazepan-2-one | HIV 1 | 10.1021/jm9602571 | Lam et al | ZINC96126610 | |
| 15 | (4R,5S,6S,7R)-4,7-dibenzyl-5,6-dihydroxy-3-(5-hydroxypentyl)-1-(5-methanesulfonylpentyl)-1,3-diazepan-2-one | HIV 1 | 10.1021/jm9602571 | Lam et al | ZINC14907381 | |
| 16 | (4R,5S,6S,7R)-4,7-dibenzyl-5,6-dihydroxy-1-(6-hydroxyhexyl)-3-(5-hydroxypentyl)-1,3-diazepan-2-one | HIV 1 | 10.1021/jm9602571 | Lam et al | ZINC14907383 | |
| 17 | (4R,5S,6S,7R)-4,7-dibenzyl-5,6-dihydroxy-3-(3-methylbut-2-en-1-yl)-1-(prop-2-en-1-yl)-1,3-diazepan-2-one | HIV 1 | 10.1021/jm9602571 | Lam et al | ZINC14907384 | |
| 18 | (4R,5S,6S,7R)-4,7-dibenzyl-5,6-dihydroxy-1,3-bis(5-hydroxyhexyl)-1,3-diazepan-2-one | HIV 1 | 10.1021/jm9602571 | Lam et al | ZINC14907392 | |
| 19 | (4R,5S,6S,7R)-4,7-dibenzyl-1-(cyclopentylmethyl)-5,6-dihydroxy-3-(prop-2-en-1-yl)-1,3-diazepan-2-one | HIV 1 | 10.1021/jm9602571 | Lam et al | ZINC14907393 | |
| 20 | (4R,5S,6S,7R)-4,7-dibenzyl-5,6-dihydroxy-3-(5-hydroxypentyl)-1-(5-methanesulfinylpentyl)-1,3-diazepan-2-one | HIV 1 | 10.1021/jm9602571 | Lam et al | ZINC14907397 | |
| 21 | (4R,5S,6S,7R)-4,7-dibenzyl-1-(cyclopropylmethyl)-5,6-dihydroxy-3-(prop-2-en-1-yl)-1,3-diazepan-2-one | HIV 1 | 10.1021/jm9602571 | Lam et al | ZINC14907398 | |
| 22 | (4R,5S,6S,7R)-4,7-dibenzyl-1,3-bis(5,6-dihydroxyhexyl)-5,6-dihydroxy-1,3-diazepan-2-one | HIV 1 | 10.1021/jm9602571 | Lam et al | ZINC14907405 | |
| 23 | (4R,5S,6S,7R)-4,7-dibenzyl-1,3-bis(cyclopentylmethyl)-5,6-dihydroxy-1,3-diazepan-2-one | HIV 1 | 10.1021/jm9602571 | Lam et al | ZINC13604439 | |
| 24 | (4R,5S,6S,7R)-4,7-dibenzyl-1-butyl-5,6-dihydroxy-3-(prop-2-en-1-yl)-1,3-diazepan-2-one | HIV 1 | 10.1021/jm9602571 | Lam et al | ZINC14907407 | |
| 25 | (4R,5S,6S,7R)-4,7-dibenzyl-5,6-dihydroxy-1,3-dipentyl-1,3-diazepan-2-one | HIV 1 | 10.1021/jm9602571 | Lam et al | ZINC13604418 | |
| 26 | (4R,5S,6S,7R)-4,7-dibenzyl-1-(cyclopentylmethyl)-3-(cyclopropylmethyl)-5,6-dihydroxy-1,3-diazepan-2-one | HIV 1 | 10.1021/jm9602571 | Lam et al | ZINC14907409 | |
| 27 | (4R,5S,6S,7R)-4,7-dibenzyl-1-butyl-3-(cyclopentylmethyl)-5,6-dihydroxy-1,3-diazepan-2-one | HIV 1 | 10.1021/jm9602571 | Lam et al | ZINC14907413 | |
| 28 | (4R,5S,6S,7R)-4,7-dibenzyl-5,6-dihydroxy-1,3-bis(prop-2-en-1-yl)-1,3-diazepan-2-one | HIV 1 | 10.1021/jm9602571 | Lam et al | ZINC03786324 | |
| 29 | (4R,5S,6S,7R)-4,7-dibenzyl-1,3-dihexyl-5,6-dihydroxy-1,3-diazepan-2-one | HIV 1 | 10.1021/jm9602571 | Lam et al | ZINC13604420 | |
| 30 | (4R,5S,6S,7R)-4,7-dibenzyl-1-(cyclopropylmethyl)-5,6-dihydroxy-3-[(2E)-3-phenylprop-2-en-1-yl]-1,3-diazepan-2-one | HIV 1 | 10.1021/jm9602571 | Lam et al | ZINC14907414 | |
| 31 | (4R,5S,6S,7R)-4,7-dibenzyl-5,6-dihydroxy-1,3-bis[(2E)-3-phenylprop-2-en-1-yl]-1,3-diazepan-2-one | HIV 1 | 10.1021/jm9602571 | Lam et al | ZINC14907416 | |
| 32 | 4-[(1E)-3-[(4R,5S,6S,7R)-4,7-dibenzyl-5,6-dihydroxy-3-[(2E)-3-[1-(methylcarbamoyl)-1H-pyrazol-4-yl]prop-2-en-1-yl]-2-oxo-1,3-diazepan-1-yl]prop-1-en-1-yl]-N-methyl-1H-pyrazole-1-carboxamide | HIV 1 | 10.1021/jm9602571 | Lam et al | ZINC96126612 | |
| 33 | (4R,5S,6S,7R)-4,7-dibenzyl-5,6-dihydroxy-1,3-bis(4-methylpentyl)-1,3-diazepan-2-one | HIV 1 | 10.1021/jm9602571 | Lam et al | ZINC13604429 | |
| 34 | (4R,5S,6S,7R)-4,7-dibenzyl-5,6-dihydroxy-3-(5-hydroxypentyl)-1-(5-methoxypentyl)-1,3-diazepan-2-one | HIV 1 | 10.1021/jm9602571 | Lam et al | ZINC14907420 | |
| 35 | (4R,5S,6S,7R)-4,7-dibenzyl-5,6-dihydroxy-1,3-dipropyl-1,3-diazepan-2-one | HIV 1 | 10.1021/jm9602571 | Lam et al | ZINC06380343 | |
| 36 | (4R,5S,6S,7R)-4,7-dibenzyl-1,3-bis(hex-5-en-1-yl)-5,6-dihydroxy-1,3-diazepan-2-one | HIV 1 | 10.1021/jm9602571 | Lam et al | ZINC14907422 | |
| 37 | (4R,5S,6S,7R)-4,7-dibenzyl-5,6-dihydroxy-1,3-bis(3-methylbutyl)-1,3-diazepan-2-one: | HIV 1 | 10.1021/jm9602571 | Lam et al | ZINC13604427 | |
| 38 | (4R,5S,6S,7R)-4,7-dibenzyl-5,6-dihydroxy-1,3-bis[4-(oxiran-2-yl)butyl]-1,3-diazepan-2-one | HIV 1 | 10.1021/jm9602571 | Lam et al | ZINC14907429 | |
| 39 | (4R,5S,6S,7R)-4,7-dibenzyl-5,6-dihydroxy-1,3-bis[(5Z)-5-(hydroxyimino)hexyl]-1,3-diazepan-2-one | HIV 1 | 10.1021/jm9602571 | Lam et al | ZINC14907431 | |
| 40 | (4R,5S,6S,7R)-4,7-dibenzyl-1-(6-chlorohexyl)-5,6-dihydroxy-3-(6-hydroxyhexyl)-1,3-diazepan-2-one | HIV 1 | 10.1021/jm9602571 | Lam et al | ZINC14907433 | |
| 41 | (4R,5S,6S,7R)-4,7-dibenzyl-1,3-bis(6,6-dibromohexyl)-5,6-dihydroxy-1,3-diazepan-2-one | HIV 1 | 10.1021/jm9602571 | Lam et al | ZINC96126626 | |
| 42 | (4R,5S,6S,7R)-4,7-dibenzyl-5,6-dihydroxy-1,3-bis[(2E)-3-(2-hydroxyphenyl)prop-2-en-1-yl]-1,3-diazepan-2-one | HIV 1 | 10.1021/jm9602571 | Lam et al | ZINC14907434 | |
| 43 | (4R,5S,6S,7R)-4,7-dibenzyl-5,6-dihydroxy-1-(5-hydroxypentyl)-3-[5-(methylsulfanyl)pentyl]-1,3-diazepan-2-one | HIV 1 | 10.1021/jm9602571 | Lam et al | ZINC14907437 | |
| 44 | (4R,5S,6S,7R)-4,7-dibenzyl-5,6-dihydroxy-1,3-bis(5-methylhexyl)-1,3-diazepan-2-one | HIV 1 | 10.1021/jm9602571 | Lam et al | ZINC14907441 | |
| 45 | (4R,5S,6S,7R)-4,7-dibenzyl-5,6-dihydroxy-1,3-bis({[2-(hydroxymethyl)cyclopropyl]methyl})-1,3-diazepan-2-one | HIV 1 | 10.1021/jm9602571 | Lam et al | ZINC14907451 | |
| 46 | (4R,5S,6S,7R)-4,7-dibenzyl-5,6-dihydroxy-1,3-bis(7-hydroxyheptyl)-1,3-diazepan-2-one | HIV 1 | 10.1021/jm9602571 | Lam et al | ZINC14907453 | |
| 47 | (4R,5S,6S,7R)-4,7-dibenzyl-1,3-bis(3,3-dimethylbutyl)-5,6-dihydroxy-1,3-diazepan-2-one | HIV 1 | 10.1021/jm9602571 | Lam et al | ZINC13604430 | |
| 48 | (4R,5S,6S,7R)-4,7-dibenzyl-1,3-bis(cyclohexylmethyl)-5,6-dihydroxy-1,3-diazepan-2-one | HIV 1 | 10.1021/jm9602571 | Lam et al | ZINC14907456 | |
| 49 | (4R,5S,6S,7R)-4,7-dibenzyl-5,6-dihydroxy-1,3-bis(3-phenylpropyl)-1,3-diazepan-2-one | HIV 1 | 10.1021/jm9602571 | Lam et al | ZINC14907458 | |
| 50 | (4R,5S,6S,7R)-4,7-dibenzyl-5,6-dihydroxy-1,3-bis[(2E)-3-{1-[(2-methoxyethoxy)methyl]-1H-pyrazol-4-yl}prop-2-en-1-yl]-1,3-diazepan-2-one | HIV 1 | 10.1021/jm9602571 | Lam et al | ZINC96126628 | |
| 51 | (4R,5S,6S,7R)-4,7-dibenzyl-5,6-dihydroxy-1,3-bis(4-hydroxybutyl)-1,3-diazepan-2-one | HIV 1 | 10.1021/jm9602571 | Lam et al | ZINC14907460 | |
| 52 | (4R,5S,6S,7R)-4,7-dibenzyl-5,6-dihydroxy-1,3-bis(5-hydroxy-5-methylhexyl)-1,3-diazepan-2-one | HIV 1 | 10.1021/jm9602571 | Lam et al | ZINC14907463 | |
| 53 | (4R,5S,6S,7R)-4,7-dibenzyl-5,6-dihydroxy-1,3-bis(2-methylpropyl)-1,3-diazepan-2-one | HIV 1 | 10.1021/jm9602571 | Lam et al | ZINC13604426 | |
| 54 | (4R,5S,6S,7R)-4,7-dibenzyl-5,6-dihydroxy-1,3-bis(3-hydroxypropyl)-1,3-diazepan-2-one | HIV 1 | 10.1021/jm9602571 | Lam et al | ZINC14907466 | |
| 55 | (4R,5S,6S,7R)-4,7-dibenzyl-1,3-bis[2-(ethenyloxy)ethyl]-5,6-dihydroxy-1,3-diazepan-2-one | HIV 1 | 10.1021/jm9602571 | Lam et al | ZINC13604436 | |
| 56 | (4R,5S,6S,7R)-4,7-dibenzyl-5,6-dihydroxy-1,3-bis(5-oxohexyl)-1,3-diazepan-2-one | HIV 1 | 10.1021/jm9602571 | Lam et al | ZINC14907468 | |
| 57 | (4R,5S,6S,7R)-4,7-dibenzyl-1-butyl-5,6-dihydroxy-3-(2-phenylethyl)-1,3-diazepan-2-one | HIV 1 | 10.1021/jm9602571 | Lam et al | ZINC14907471 | |
| 58 | (4R,5S,6S,7R)-4,7-dibenzyl-5,6-dihydroxy-1,3-bis(5-methoxypentyl)-1,3-diazepan-2-one | HIV 1 | 10.1021/jm9602571 | Lam et al | ZINC14907472 | |
| 59 | (4R,5S,6S,7R)-4,7-dibenzyl-1,3-diethyl-5,6-dihydroxy-1,3-diazepan-2-one | HIV 1 | 10.1021/jm9602571 | Lam et al | ZINC06385085 | |
| 60 | (4R,5S,6S,7R)-4,7-dibenzyl-5,6-dihydroxy-1,3-bis(6-methylheptyl)-1,3-diazepan-2-one | HIV 1 | 10.1021/jm9602571 | Lam et al | ZINC14907474 | |
| 61 | (4R,5S,6S,7R)-4,7-dibenzyl-1,3-bis[4-(4-fluorophenyl)-4-(hydroxyimino)butyl]-5,6-dihydroxy-1,3-diazepan-2-one | HIV 1 | 10.1021/jm9602571 | Lam et al | ZINC96126630 | |
| 62 | (4R,5S,6S,7R)-4,7-dibenzyl-1,3-diheptyl-5,6-dihydroxy-1,3-diazepan-2-one | HIV 1 | 10.1021/jm9602571 | Lam et al | ZINC14908001 | |
| 63 | (4R,5S,6S,7R)-4,7-dibenzyl-5,6-dihydroxy-1,3-bis[(2E)-4-{[5-(methylsulfanyl)-1H-1,2,4-triazol-3-yl]amino}but-2-en-1-yl]-1,3-diazepan-2-one | HIV 1 | 10.1021/jm9602571 | Lam et al | ZINC96126641 | |
| 64 | (4R,5S,6S,7R)-4,7-dibenzyl-1,3-bis({[(1R)-2,2-diphenylcyclopropyl]methyl})-5,6-dihydroxy-1,3-diazepan-2-one | HIV 1 | 10.1021/jm9602571 | Lam et al | ZINC96126643 | |
| 65 | (4R,5S,6S,7R)-4,7-dibenzyl-5,6-dihydroxy-3-{[(1R,2R)-2-phenylcyclopropyl]methyl}-1-{[(1S,2S)-2-phenylcyclopropyl]methyl}-1,3-diazepan-2-one | HIV 1 | 10.1021/jm9602571 | Lam et al | ZINC96126645 | |
| 66 | (4R,5S,6S,7R)-4,7-dibenzyl-5,6-dihydroxy-1,3-bis(8-hydroxyoctyl)-1,3-diazepan-2-one | HIV 1 | 10.1021/jm9602571 | Lam et al | ZINC14908007 | |
| 67 | (4R,5S,6S,7R)-4,7-dibenzyl-5,6-dihydroxy-1,3-bis(2-methoxyethyl)-1,3-diazepan-2-one | HIV 1 | 10.1021/jm9602571 | Lam et al | ZINC13604422 | |
| 68 | (4R,5S,6S,7R)-4,7-dibenzyl-1,3-bis[4-(dimethylamino)butyl]-5,6-dihydroxy-1,3-diazepan-2-one dihydrochloride | HIV 1 | 10.1021/jm9602571 | Lam et al | ZINC14908009 | |
| 69 | (4R,5S,6S,7R)-1,3-bis[2-(adamantan-1-yl)ethyl]-4,7-dibenzyl-5,6-dihydroxy-1,3-diazepan-2-one | HIV 1 | 10.1021/jm9602571 | Lam et al | ZINC96126648 | |
| 70 | (4R,5S,6S,7R)-4,7-dibenzyl-1-[(4R)-4-(4-fluorophenyl)-4-hydroxybutyl]-3-[(4S)-4-(4-fluorophenyl)-4-hydroxybutyl]-5,6-dihydroxy-1,3-diazepan-2-one | HIV 1 | 10.1021/jm9602571 | Lam et al | ZINC96126649 | |
| 71 | (4S,5R,6R,7S)-4,7-dibenzyl-1,3-bis(2-ethoxyethyl)-5,6-dihydroxy-1,3-diazepan-2-one | HIV 1 | 10.1021/jm9602571 | Lam et al | ZINC14908011 | |
| 72 | (4R,5S,6S,7R)-4,7-dibenzyl-5,6-dihydroxy-1,3-bis(2-hydroxy-3-phenylpropyl)-1,3-diazepan-2-one | HIV 1 | 10.1021/jm9602571 | Lam et al | ZINC14908018 | |
| 73 | (4R,5S,6S,7R)-1,3-bis(5-aminopentyl)-4,7-dibenzyl-5,6-dihydroxy-1,3-diazepan-2-one | HIV 1 | 10.1021/jm9602571 | Lam et al | ZINC14908020 | |
| 74 | (4R,5S,6S,7R)-1,3-bis(4-aminobutyl)-4,7-dibenzyl-5,6-dihydroxy-1,3-diazepan-2-one dihydrate dihydrochloride | HIV 1 | 10.1021/jm9602571 | Lam et al | ZINC14908023 | |
| 75 | (4R,5S,6S,7R)-1,3-bis[4-amino-4-(4-fluorophenyl)butyl]-4,7-dibenzyl-5,6-dihydroxy-1,3-diazepan-2-one | HIV 1 | 10.1021/jm9602571 | Lam et al | ZINC96126658 | |
| 76 | (4R,5S,6S,7R)-4,7-dibenzyl-5,6-dihydroxy-1,3-bis[5-(1H-pyrazol-1-yl)pentyl]-1,3-diazepan-2-one | HIV 1 | 10.1021/jm9602571 | Lam et al | ZINC14908025 | |
| 77 | (4R,5S,6S,7R)-4,7-dibenzyl-5,6-dihydroxy-1,3-bis(3-phenylprop-2-yn-1-yl)-1,3-diazepan-2-one | HIV 1 | 10.1021/jm9602571 | Lam et al | ZINC14908027 | |
| 78 | (4R,5S,6S,7R)-4,7-dibenzyl-1,3-bis[(2E)-4-[(3,5-dimethylphenyl)amino]but-2-en-1-yl]-5,6-dihydroxy-1,3-diazepan-2-one dihydrochloride | HIV 1 | 10.1021/jm9602571 | Lam et al | ZINC96126660 | |
| 79 | (4R,5S,6S,7R)-4,7-dibenzyl-5,6-dihydroxy-1,3-bis[2-(2-hydroxyethoxy)ethyl]-1,3-diazepan-2-one | HIV 1 | 10.1021/jm9602571 | Lam et al | ZINC14908031 | |
| 80 | (4R,5S,6S,7R)-4,7-dibenzyl-1,3-bis[2-(dimethylamino)ethyl]-5,6-dihydroxy-1,3-diazepan-2-one | HIV 1 | 10.1021/jm9602571 | Lam et al | ZINC14908032 | |
| 81 | (4R,5S,6S,7R)-4,7-dibenzyl-5,6-dihydroxy-1,3-bis({2-[3-(2-hydroxypropyl)-2,2-dimethylcyclopropyl]ethyl})-1,3-diazepan-2-one | HIV 1 | 10.1021/jm9602571 | Lam et al | ZINC70454613 | |
| 82 | (4R,5S,6S,7R)-4,7-dibenzyl-1,3-bis[4-(4-fluorophenyl)-4-oxobutyl]-5,6-dihydroxy-1,3-diazepan-2-one | HIV 1 | 10.1021/jm9602571 | Lam et al | ZINC96126662 | |
| 83 | (4R,5S,6S,7R)-4,7-dibenzyl-5,6-dihydroxy-1,3-bis[(3-methyl-4,5-dihydro-1,2-oxazol-5-yl)methyl]-1,3-diazepan-2-one | HIV 1 | 10.1021/jm9602571 | Lam et al | ZINC14908048 | |
| 84 | (4R,5S,6S,7R)-4,7-dibenzyl-5,6-dihydroxy-1,3-bis[2-(morpholin-4-yl)ethyl]-1,3-diazepan-2-one | HIV 1 | 10.1021/jm9602571 | Lam et al | ZINC14908050 | |
| 85 | (4R,5S,6S,7R)-4,7-dibenzyl-5,6-dihydroxy-1,3-bis(2-hydroxybutyl)-1,3-diazepan-2-one | HIV 1 | 10.1021/jm9602571 | Lam et al | ZINC14908058 | |
| 86 | (4R,5S,6S,7R)-4,7-dibenzyl-5,6-dihydroxy-1,3-bis[2-(piperidin-4-yl)ethyl]-1,3-diazepan-2-one | HIV 1 | 10.1021/jm9602571 | Lam et al | ZINC14908059 | |
| 87 | (4R,5S,6S,7R)-4,7-dibenzyl-5,6-dihydroxy-1,3-bis[5-(1H-imidazol-1-yl)pentyl]-1,3-diazepan-2-one dihydrochloride | HIV 1 | 10.1021/jm9602571 | Lam et al | ZINC14908063 | |
| 88 | (4R,5S,6S,7R)-4,7-dibenzyl-5,6-dihydroxy-1,3-bis[5-(morpholin-4-yl)pentyl]-1,3-diazepan-2-one dihydrochloride | HIV 1 | 10.1021/jm9602571 | Lam et al | ZINC14908065 | |
| 89 | (4R,5S,6S,7R)-4,7-dibenzyl-5,6-dihydroxy-1,3-bis(2-hydroxy-3,3-dimethylbutyl)-1,3-diazepan-2-one | HIV 1 | 10.1021/jm9602571 | Lam et al | ZINC14908074 | |
| 90 | (4R,5S,6S,7R)-4,7-dibenzyl-5,6-dihydroxy-1,3-bis(2-hydroxyethyl)-1,3-diazepan-2-one | HIV 1 | 10.1021/jm9602571 | Lam et al | ZINC14908076 | |
| 91 | (4R,5S,6S,7R)-4,7-dibenzyl-5,6-dihydroxy-1,3-bis({[4-(hydroxymethyl)phenyl]methyl})-1,3-diazepan-2-one | HIV 1 | 10.1021/jm9602571 | Lam et al | ZINC03833858 | |
| 92 | (4R,5S,6S,7R)-1,3-bis[(3-aminophenyl)methyl]-4,7-dibenzyl-5,6-dihydroxy-1,3-diazepan-2-one | HIV 1 | 10.1021/jm9602571 | Lam et al | ZINC03831996 | |
| 93 | (4R,5S,6S,7R)-4,7-dibenzyl-5,6-dihydroxy-1,3-bis(prop-2-en-1-yl)-1,3-diazepan-2-one | HIV 1 | 10.1021/jm9602571 | Lam et al | ZINC03786324 | |
| 94 | (4R,5S,6S,7R)-4,7-dibenzyl-5,6-dihydroxy-1,3-bis(naphthalen-2-ylmethyl)-1,3-diazepan-2-one | HIV 1 | 10.1021/jm9602571 | Lam et al | ZINC03833856 | |
| 95 | 3-{[(4R,5S,6S,7R)-4,7-dibenzyl-5,6-dihydroxy-2-oxo-3-{[3-(1,3-thiazol-2-ylcarbamoyl)phenyl]methyl}-1,3-diazepan-1-yl]methyl}-N-(1,3-thiazol-2-yl)benzamide | HIV 1 | 10.1021/jm9602571 | Lam et al | ZINC85548877 | |
| 96 | 3-{[(4R,5S,6S,7R)-4,7-dibenzyl-3-[(3-carbamoylphenyl)methyl]-5,6-dihydroxy-2-oxo-1,3-diazepan-1-yl]methyl}benzamide | HIV 1 | 10.1021/jm9602571 | Lam et al | ZINC14908078 | |
| 97 | 3-{[(4R,5S,6S,7R)-4,7-dibenzyl-5,6-dihydroxy-3-{[3-(methylcarbamoyl)phenyl]methyl}-2-oxo-1,3-diazepan-1-yl]methyl}-N-methylbenzamide | HIV 1 | 10.1021/jm9602571 | Lam et al | ZINC14908079 | |
| 98 | 3-{[(4R,5S,6S,7R)-4,7-dibenzyl-3-{[3-(ethylcarbamoyl)phenyl]methyl}-5,6-dihydroxy-2-oxo-1,3-diazepan-1-yl]methyl}-N-ethylbenzamide | HIV 1 | 10.1021/jm9602571 | Lam et al | ZINC26493670 | |
| 99 | 3-{[(4R,5S,6S,7R)-4,7-dibenzyl-5,6-dihydroxy-2-oxo-3-{[3-(pyridin-2-ylcarbamoyl)phenyl]methyl}-1,3-diazepan-1-yl]methyl}-N-(pyridin-2-yl)benzamide | HIV 1 | 10.1021/jm9602571 | Lam et al | ZINC95543992 | |
| 100 | 3-{[(4R,5S,6S,7R)-4,7-dibenzyl-5,6-dihydroxy-3-{[3-(1H-imidazol-2-ylcarbamoyl)phenyl]methyl}-2-oxo-1,3-diazepan-1-yl]methyl}-N-(1H-imidazol-2-yl)benzamide | HIV 1 | 10.1021/jm9602571 | Lam et al | ZINC29556734 | |
| 101 | (4R,5S,6S,7R)-4,7-dibenzyl-5,6-dihydroxy-1,3-bis(1H-indazol-5-ylmethyl)-1,3-diazepan-2-one | HIV 1 | 10.1021/jm9602571 | Lam et al | ZINC03935743 | |
| 102 | 3-{[(4R,5S,6S,7R)-4,7-dibenzyl-5,6-dihydroxy-3-{[3-(N'-hydroxycarbamimidoyl)phenyl]methyl}-2-oxo-1,3-diazepan-1-yl]methyl}-N'-hydroxybenzene-1-carboximidamide[[Tetrahydro-5,6-dihydroxy-2-oxo-4,7-bis(phenylmethyl)-1H-1,3-diazepine-1,3(2H)-diyl]bis(methylene)]bis[N-hydroxybenzenecarboximidamide] | HIV 1 | 10.1021/jm9602571 | Lam et al | ZINC14908086 | |
| 103 | (4R,5R,6R)-4-benzyl-5-hydroxy-1,3-bis(1H-indazol-5-ylmethyl)-6-(2-phenylethyl)-1,3-diazinan-2-one | HIV 1 | 10.1021/jm9602571 | Lam et al | ZINC14908088 | |
| 104 | (4R,5S,6S,7R)-4,7-dibenzyl-5,6-dihydroxy-1-(1H-indazol-5-ylmethyl)-1,3-diazepan-2-one | HIV 1 | 10.1021/jm9602571 | Lam et al | ZINC13780010 | |
| 105 | 3-{[(4R,5S,6S,7R)-4,7-dibenzyl-5,6-dihydroxy-3-(1H-indazol-5-ylmethyl)-2-oxo-1,3-diazepan-1-yl]methyl}benzoic acid | HIV 1 | 10.1021/jm9602571 | Lam et al | ZINC14908089 | |
| 106 | (4R,5S,6S,7R)-4,7-dibenzyl-5,6-dihydroxy-3-[(3-hydroxyphenyl)methyl]-1-(1H-indazol-5-ylmethyl)-1,3-diazepan-2-one | HIV 1 | 10.1021/jm9602571 | Lam et al | ZINC14908090 | |
| 107 | (4R,5S,6S,7R)-4,7-dibenzyl-5,6-dihydroxy-3-{[3-(2-hydroxyethyl)phenyl]methyl}-1-(1H-indazol-5-ylmethyl)-1,3-diazepan-2-one | HIV 1 | 10.1021/jm9602571 | Lam et al | ZINC14908092 | |
| 108 | 3-{[(4R,5S,6S,7R)-4,7-dibenzyl-3-[(3-formylphenyl)methyl]-5,6-dihydroxy-2-oxo-1,3-diazepan-1-yl]methyl}benzaldehyde | HIV 1 | 10.1021/jm9602571 | Lam et al | ZINC14908096 | |
| 109 | (4R,5S,6S,7R)-4,7-dibenzyl-1,3-bis[(3-acetylphenyl)methyl]-5,6-dihydroxy-1,3-diazepan-2-one::(4R,5S,6S,7R)-Hexahydro-5,6-dihydroxy-1,3-bis[(3-acetylphenyl)methyl]-4,7-bis(phenylmethyl)-2H-1,3-diazepin-2-one | HIV 1 | 10.1021/jm9602571 | Lam et al | ZINC14908098 | |
| 110 | (4R,5S,6S,7R)-4,7-dibenzyl-5,6-dihydroxy-1,3-bis[(3-propanoylphenyl)methyl]-1,3-diazepan-2-one | HIV 1 | 10.1021/jm9602571 | Lam et al | ZINC14908100 | |
| 111 | (4R,5S,6S,7R)-4,7-dibenzyl-1,3-bis[(3-butanoylphenyl)methyl]-5,6-dihydroxy-1,3-diazepan-2-one | HIV 1 | 10.1021/jm9602571 | Lam et al | ZINC26151297 | |
| 112 | (4R,5S,6S,7R)-4,7-dibenzyl-5,6-dihydroxy-1,3-bis({[3-(2,2,2-trifluoroacetyl)phenyl]methyl})-1,3-diazepan-2-one::(4R,5S,6S,7R)-Hexahydro-5,6-dihydroxy-1,3-bis[[3-(trifluoroacetyl)phenyl]methyl]-4,7-bis(phenylmethyl)-2H-1,3-diazepin-2-one | HIV 1 | 10.1021/jm9602571 | Lam et al | ZINC26161293 | |
| 113 | (4R,5S,6S,7R)-4,7-dibenzyl-1,3-bis({[3-(2,2-dimethylpropanoyl)phenyl]methyl})-5,6-dihydroxy-1,3-diazepan-2-one::(4R,5S,6S,7R)-Hexahydro-5,6-dihydroxy-1,3-bis[(3-pivaloylphenyl)methyl]-4,7-bis(phenylmethyl)-2H-1,3-diazepin-2-one | HIV 1 | 10.1021/jm9602571 | Lam et al | ZINC26151288 | |
| 114 | (4R,5S,6S,7R)-4,7-dibenzyl-5,6-dihydroxy-1,3-bis({3-[(hydroxyimino)methyl]phenyl}methyl)-1,3-diazepan-2-one | HIV 1 | 10.1021/jm9602571 | Lam et al | ZINC14908107 | |
| 115 | (4R,5S,6S,7R)-4,7-dibenzyl-5,6-dihydroxy-1,3-bis({3-[1-(hydroxyimino)ethyl]phenyl}methyl)-1,3-diazepan-2-one::4R,5S,6S,7R)-Hexahydro-5,6-dihydroxy-1,3-bis[[3-[1-(hydroxyimino)ethyl]phenyl]methyl]-4,7-bis(phenylmethyl)-2H-1,3-diazepin-2-one | HIV 1 | 10.1021/jm9602571 | Lam et al | ZINC14908114 | |
| 116 | (4R,5S,6S,7R)-4,7-dibenzyl-5,6-dihydroxy-1,3-bis({3-[1-(hydroxyimino)propyl]phenyl}methyl)-1,3-diazepan-2-one | HIV 1 | 10.1021/jm9602571 | Lam et al | ZINC26167193 | |
| 117 | (4R,5S,6S,7R)-4,7-dibenzyl-5,6-dihydroxy-1,3-bis({3-[1-(hydroxyimino)butyl]phenyl}methyl)-1,3-diazepan-2-one | HIV 1 | 10.1021/jm9602571 | Lam et al | ZINC26162491 | |
| 118 | (4R,5S,6S,7R)-4,7-dibenzyl-5,6-dihydroxy-1,3-bis({3-[2,2,2-trifluoro-1-(hydroxyimino)ethyl]phenyl}methyl)-1,3-diazepan-2-one | HIV 1 | 10.1021/jm9602571 | Lam et al | ZINC26166723 | |
| 119 | (4R,5S,6S,7R)-4,7-dibenzyl-5,6-dihydroxy-1,3-bis({[3-(1H-pyrazol-3-yl)phenyl]methyl})-1,3-diazepan-2-one::(4R,5S,6S,7R)-Hexahydro-5,6-dihydroxy-1,3-bis[[3-(1H-pyrazol-3-yl)phenyl]methyl]-4,7-bis(phenylmethyl)-2H-1,3-diazepin-2-one | HIV 1 | 10.1021/jm9602571 | Lam et al | ZINC03937353 | |
| 120 | (4R,5S,6S,7R)-4,7-dibenzyl-5,6-dihydroxy-1,3-bis({[3-(1H-pyrazol-4-yl)phenyl]methyl})-1,3-diazepan-2-one::(4R,5S,6S,7R)-Hexahydro-5,6-dihydroxy-1,3-bis[[3-(1H-prazol-4-yl)phenyl]methyl]-4,7-bis(phenylmethyl)-2H-1,3-diazepin-2-one | HIV 1 | 10.1021/jm9602571 | Lam et al | ZINC26168566 | |
| 121 | (4R,5S,6S,7R)-4,7-dibenzyl-5,6-dihydroxy-1,3-bis({[3-(1H-imidazol-2-yl)phenyl]methyl})-1,3-diazepan-2-one::(4R,5S,6S,7R)-Hexahydro-5,6-dihydroxy-1,3-bis[[3-(1H-imidazol-2-yl)phenyl]methyl]-4,7-bis(phenylmethyl)-2H-1,3-diazepin-2-one | HIV 1 | 10.1021/jm9602571 | Lam et al | ZINC26165759 | |
| 122 | (4R,5S,6S,7R)-4,7-dibenzyl-5,6-dihydroxy-1,3-bis({[3-(1H-imidazol-4-yl)phenyl]methyl})-1,3-diazepan-2-one | HIV 1 | 10.1021/jm9602571 | Lam et al | ZINC26162995 | |
| 123 | (4R,5S,6S,7R)-4,7-dibenzyl-5,6-dihydroxy-1,3-bis({[3-(2H-1,2,3-triazol-4-yl)phenyl]methyl})-1,3-diazepan-2-one | HIV 1 | 10.1021/jm9602571 | Lam et al | ZINC26165871 | |
| 124 | (4R,5S,6S,7R)-4,7-dibenzyl-5,6-dihydroxy-1,3-bis({[3-(1H-1,2,4-triazol-3-yl)phenyl]methyl})-1,3-diazepan-2-one | HIV 1 | 10.1021/jm9602571 | Lam et al | ZINC26167582 | |
| 125 | (4R,5S,6S,7R)-4,7-dibenzyl-5,6-dihydroxy-1,3-bis({[3-(2H-1,2,3,4-tetrazol-5-yl)phenyl]methyl})-1,3-diazepan-2-one | HIV 1 | 10.1021/jm9602571 | Lam et al | ZINC26174612 | |
| 126 | (4R,5S,6S,7R)-4,7-dibenzyl-5,6-dihydroxy-1,3-dimethyl-1,3-diazepan-2-one | HIV 1 | 10.1021/jm9602571 | Lam et al | ZINC06481191 | |
| 127 | (4R,5S,6S,7R)-4,7-dibenzyl-1,3-bis(2-ethoxyethyl)-5,6-dihydroxy-1,3-diazepan-2-one | HIV 1 | 10.1021/jm9602571 | Lam et al | ZINC13604424 | |
| 128 | (4R,5S,6S,7R)-4,7-dibenzyl-5,6-dihydroxy-1,3-bis[2-(2-methoxyethoxy)ethyl]-1,3-diazepan-2-one | HIV 1 | 10.1021/jm9602571 | Lam et al | ZINC14946430 | |
| 129 | (4R,5S,6S,7R)-4,7-dibenzyl-5,6-dihydroxy-1,3-bis(2-methylprop-2-en-1-yl)-1,3-diazepan-2-one | HIV 1 | 10.1021/jm9602571 | Lam et al | ZINC13604432 | |
| 130 | (4R,5S,6S,7R)-4,7-dibenzyl-5,6-dihydroxy-1,3-bis(prop-2-yn-1-yl)-1,3-diazepan-2-one | HIV 1 | 10.1021/jm9602571 | Lam et al | ZINC05827983 | |
| 131 | (4R,5S,6S,7R)-4,7-dibenzyl-5,6-dihydroxy-1,3-bis(pyridin-2-ylmethyl)-1,3-diazepan-2-one | HIV 1 | 10.1021/jm9602571 | Lam et al | ZINC14946431 | |
| 132 | (4R,5S,6S,7R)-4,7-dibenzyl-5,6-dihydroxy-1,3-bis(pyridin-3-ylmethyl)-1,3-diazepan-2-one | HIV 1 | 10.1021/jm9602571 | Lam et al | ZINC14946432 | |
| 133 | (4R,5S,6S,7R)-4,7-dibenzyl-5,6-dihydroxy-1,3-bis(pyridin-4-ylmethyl)-1,3-diazepan-2-one | HIV 1 | 10.1021/jm9602571 | Lam et al | ZINC14946433 | |
| 134 | (4R,5S,6S,7R)-4,7-dibenzyl-5,6-dihydroxy-1-(naphthalen-2-ylmethyl)-3-(pyridin-4-ylmethyl)-1,3-diazepan-2-one | HIV 1 | 10.1021/jm9602571 | Lam et al | ZINC14946434 | |
| 135 | (4R,5S,6S,7R)-4,7-dibenzyl-1-[(4-fluorophenyl)methyl]-5,6-dihydroxy-3-(naphthalen-2-ylmethyl)-1,3-diazepan-2-one | HIV 1 | 10.1021/jm9602571 | Lam et al | ZINC14946435 | |
| 136 | (4R,5S,6S,7R)-4,7-dibenzyl-5,6-dihydroxy-3-{[4-(hydroxymethyl)phenyl]methyl}-1-(naphthalen-2-ylmethyl)-1,3-diazepan-2-one | HIV 1 | 10.1021/jm9602571 | Lam et al | ZINC14946436 | |
| 137 | (4R,5S,6S,7R)-1-[(3-aminophenyl)methyl]-4,7-dibenzyl-5,6-dihydroxy-3-(naphthalen-2-ylmethyl)-1,3-diazepan-2-one | HIV 1 | 10.1021/jm9602571 | Lam et al | ZINC14946437 | |
| 138 | (4R,5S,6S,7R)-4,7-dibenzyl-5,6-dihydroxy-3-[(3-hydroxyphenyl)methyl]-1-(naphthalen-2-ylmethyl)-1,3-diazepan-2-one | HIV 1 | 10.1021/jm9602571 | Lam et al | ZINC14946438 | |
| 139 | (4R,5S,6S,7R)-4,7-dibenzyl-5,6-dihydroxy-1,3-bis(naphthalen-1-ylmethyl)-1,3-diazepan-2-one | HIV 1 | 10.1021/jm9602571 | Lam et al | ZINC14946439 | |
| 140 | (4R,5S,6S,7R)-4,7-dibenzyl-1,3-bis[(2-fluorophenyl)methyl]-5,6-dihydroxy-1,3-diazepan-2-one | HIV 1 | 10.1021/jm9602571 | Lam et al | ZINC14946440 | |
| 141 | (4R,5S,6S,7R)-4,7-dibenzyl-1,3-bis[(3-fluorophenyl)methyl]-5,6-dihydroxy-1,3-diazepan-2-one | HIV 1 | 10.1021/jm9602571 | Lam et al | ZINC14946441 | |
| 142 | (4R,5S,6S,7R)-4,7-dibenzyl-1,3-bis[(4-fluorophenyl)methyl]-5,6-dihydroxy-1,3-diazepan-2-one | HIV 1 | 10.1021/jm9602571 | Lam et al | ZINC14946442 | |
| 143 | (4R,5S,6S,7R)-4,7-dibenzyl-1,3-bis[(2-chlorophenyl)methyl]-5,6-dihydroxy-1,3-diazepan-2-one | HIV 1 | 10.1021/jm9602571 | Lam et al | ZINC14946443 | |
| 144 | (4R,5S,6S,7R)-4,7-dibenzyl-1,3-bis[(3-chlorophenyl)methyl]-5,6-dihydroxy-1,3-diazepan-2-one | HIV 1 | 10.1021/jm9602571 | Lam et al | ZINC14946444 | |
| 145 | (4R,5S,6S,7R)-4,7-dibenzyl-1,3-bis[(4-chlorophenyl)methyl]-5,6-dihydroxy-1,3-diazepan-2-one | HIV 1 | 10.1021/jm9602571 | Lam et al | ZINC14946445 | |
| 146 | (4R,5S,6S,7R)-4,7-dibenzyl-1,3-bis[(3-bromophenyl)methyl]-5,6-dihydroxy-1,3-diazepan-2-one | HIV 1 | 10.1021/jm9602571 | Lam et al | ZINC26574605 | |
| 147 | (4R,5S,6S,7R)-4,7-dibenzyl-1,3-bis[(4-bromophenyl)methyl]-5,6-dihydroxy-1,3-diazepan-2-one | HIV 1 | 10.1021/jm9602571 | Lam et al | ZINC26576034 | |
| 148 | (4R,5S,6S,7R)-4,7-dibenzyl-5,6-dihydroxy-1,3-bis[(3-methylphenyl)methyl]-1,3-diazepan-2-one | HIV 1 | 10.1021/jm9602571 | Lam et al | ZINC14946446 | |
| 149 | (4R,5S,6S,7R)-4,7-dibenzyl-5,6-dihydroxy-1,3-bis[(4-methylphenyl)methyl]-1,3-diazepan-2-one | HIV 1 | 10.1021/jm9602571 | Lam et al | ZINC14946447 | |
| 150 | (4R,5S,6S,7R)-4,7-dibenzyl-5,6-dihydroxy-1,3-bis({[3-(trifluoromethyl)phenyl]methyl})-1,3-diazepan-2-one | HIV 1 | 10.1021/jm9602571 | Lam et al | ZINC26576005 | |
| 151 | (4R,5S,6S,7R)-4,7-dibenzyl-5,6-dihydroxy-1,3-bis({[4-(trifluoromethyl)phenyl]methyl})-1,3-diazepan-2-one | HIV 1 | 10.1021/jm9602571 | Lam et al | ZINC26576004 | |
| 152 | (4R,5S,6S,7R)-4,7-dibenzyl-5,6-dihydroxy-1,3-bis[(2-methoxyphenyl)methyl]-1,3-diazepan-2-one | HIV 1 | 10.1021/jm9602571 | Lam et al | ZINC14946448 | |
| 153 | (4R,5S,6S,7R)-4,7-dibenzyl-5,6-dihydroxy-1,3-bis[(3-methoxyphenyl)methyl]-1,3-diazepan-2-one | HIV 1 | 10.1021/jm9602571 | Lam et al | ZINC14946449 | |
| 154 | (4R,5S,6S,7R)-4,7-dibenzyl-5,6-dihydroxy-1,3-bis[(4-methoxyphenyl)methyl]-1,3-diazepan-2-one | HIV 1 | 10.1021/jm9602571 | Lam et al | ZINC14946450 | |
| 155 | (4R,5S,6S,7R)-4,7-dibenzyl-5,6-dihydroxy-1,3-bis[(3-iodophenyl)methyl]-1,3-diazepan-2-one | HIV 1 | 10.1021/jm9602571 | Lam et al | ZINC95544621 | |
| 156 | (4S,5R,6R,7S)-4,7-dibenzyl-5,6-dihydroxy-1,3-bis({[4-(hydroxymethyl)phenyl]methyl})-1,3-diazepan-2-one | HIV 1 | 10.1021/jm9602571 | Lam et al | ZINC14946451 | |
| 157 | (4R,5S,6S,7R)-4,7-dibenzyl-5,6-dihydroxy-1-(naphthalen-2-ylmethyl)-3-propyl-1,3-diazepan-2-one | HIV 1 | 10.1021/jm9602571 | Lam et al | ZINC14946452 | |
| 158 | (4R,5S,6S,7R)-4,7-dibenzyl-1-butyl-5,6-dihydroxy-3-(naphthalen-2-ylmethyl)-1,3-diazepan-2-one | HIV 1 | 10.1021/jm9602571 | Lam et al | ZINC14946453 | |
| 159 | (4R,5S,6S,7R)-4,7-dibenzyl-5,6-dihydroxy-1-(naphthalen-2-ylmethyl)-3-(prop-2-en-1-yl)-1,3-diazepan-2-one | HIV 1 | 10.1021/jm9602571 | Lam et al | ZINC14946454 | |
| 160 | (4R,5S,6S,7R)-4,7-dibenzyl-1-(cyclopropylmethyl)-5,6-dihydroxy-3-(naphthalen-2-ylmethyl)-1,3-diazepan-2-one | HIV 1 | 10.1021/jm9602571 | Lam et al | ZINC14946455 | |
| 161 | (4R,5S,6S,7R)-4,7-dibenzyl-1-cyclopentyl-5,6-dihydroxy-3-(naphthalen-2-ylmethyl)-1,3-diazepan-2-one | HIV 1 | 10.1021/jm9602571 | Lam et al | ZINC14946456 | |
| 162 | (4R,5S,6S,7R)-1,4,7-tribenzyl-5,6-dihydroxy-3-(naphthalen-2-ylmethyl)-1,3-diazepan-2-one | HIV 1 | 10.1021/jm9602571 | Lam et al | ZINC14946457 | |
| 163 | (4R,5S,6S,7R)-4,7-dibenzyl-5,6-dihydroxy-1-(naphthalen-2-ylmethyl)-3-(pyridin-3-ylmethyl)-1,3-diazepan-2-one | HIV 1 | 10.1021/jm9602571 | Lam et al | ZINC14946458 | |
| 164 | (2S)-N-[(2S,3R,4R,5S)-3,4-dihydroxy-5-[(2S)-3-methyl-2-{[methyl(pyridin-2-ylmethyl)carbamoyl]amino}butanamido]-1,6-diphenylhexan-2-yl]-3-methyl-2-{[methyl(pyridin-2-ylmethyl)carbamoyl]amino}butanamide | HIV 1 | 10.1021/jm0305568 | Madhusoodan et al | ZINC95541462 | |
| 165 | (2S)-N-[(2S,3S,4S,5S)-3,4-dihydroxy-5-[(2S)-3-methyl-2-{[methyl(pyridin-2-ylmethyl)carbamoyl]amino}butanamido]-1,6-diphenylhexan-2-yl]-3-methyl-2-{[methyl(pyridin-2-ylmethyl)carbamoyl]amino}butanamide | HIV 1 | 10.1021/jm0305568 | Madhusoodan et al | ZINC95546435 | |
| 166 | (2S)-N-[(2S,3R,4S,5S)-3,4-dihydroxy-5-[(2S)-3-methyl-2-{[methyl(pyridin-2-ylmethyl)carbamoyl]amino}butanamido]-1,6-diphenylhexan-2-yl]-3-methyl-2-{[methyl(pyridin-2-ylmethyl)carbamoyl]amino}butanamide | HIV 1 | 10.1021/jm0305568 | Madhusoodan et al | ZINC85548251 | |
| 167 | (2S)-N-[(2S,3R,5S)-3-hydroxy-5-[(2S)-3-methyl-2-{[methyl(pyridin-2-ylmethyl)carbamoyl]amino}butanamido]-1,6-diphenylhexan-2-yl]-3-methyl-2-{[methyl(pyridin-2-ylmethyl)carbamoyl]amino}butanamide | HIV 1 | 10.1021/jm0305568 | Madhusoodan et al | ZINC95610155 | |
| 168 | (2S)-N-[(2S,3S)-4-[(2S)-N'-(cyclohexylmethyl)-2-acetamido-3-methylbutanehydrazido]-3-hydroxy-1-phenylbutan-2-yl]-2-acetamido-3-methylbutanamide | HIV 1 | 10.1021/jm960022p | Priestle et al | ZINC03922084 | |
| 169 | (2R,3R)-1,1-dioxo-2-(propan-2-yl)--thiolan-3-yl N-[(2S,3R)-4-[(2S)-2-(tert-butylcarbamoyl)-4-propylpiperazin-1-yl]-3-hydroxy-1-phenylbutan-2-yl]carbamate | HIV 1 |  | Kim et al | ZINC23357710 | |
| 170 | (2R,3R)-1,1-dioxo-2-(propan-2-yl)--thiolan-3-yl N-[(2S,3R)-4-[(2S)-2-(tert-butylcarbamoyl)-4-octylpiperazin-1-yl]-3-hydroxy-1-phenylbutan-2-yl]carbamate | HIV 1 |  | Kim et al | ZINC49720451 | |
| 171 | (2R,3R)-1,1-dioxo-2-(propan-2-yl)--thiolan-3-yl N-[(2S,3R)-4-[(2S)-2-(tert-butylcarbamoyl)-4-(propan-2-yl)piperazin-1-yl]-3-hydroxy-1-phenylbutan-2-yl]carbamate | HIV 1 |  | Kim et al | ZINC23357714 | |
| 172 | (2R,3R)-1,1-dioxo-2-(propan-2-yl)--thiolan-3-yl N-[(2S,3R)-4-[(2S)-2-(tert-butylcarbamoyl)-4-(pentan-3-yl)piperazin-1-yl]-3-hydroxy-1-phenylbutan-2-yl]carbamate | HIV 1 |  | Kim et al | ZINC23357716 | |
| 173 | (2R,3R)-1,1-dioxo-2-(propan-2-yl)--thiolan-3-yl N-[(2S,3R)-4-[(2S)-2-(tert-butylcarbamoyl)-4-(2-methylpropyl)piperazin-1-yl]-3-hydroxy-1-phenylbutan-2-yl]carbamate | HIV 1 |  | Kim et al | ZINC23357719 | |
| 174 | (2R,3R)-1,1-dioxo-2-(propan-2-yl)--thiolan-3-yl N-[(2S,3R)-4-[(2S)-2-(tert-butylcarbamoyl)-4-(2,2-dimethylpropyl)piperazin-1-yl]-3-hydroxy-1-phenylbutan-2-yl]carbamate | HIV 1 |  | Kim et al | ZINC23357722 | |
| 175 | (2R,3R)-1,1-dioxo-2-(propan-2-yl)--thiolan-3-yl N-[(2S,3R)-4-[(2S)-2-(tert-butylcarbamoyl)-4-cycloheptylpiperazin-1-yl]-3-hydroxy-1-phenylbutan-2-yl]carbamate | HIV 1 |  | Kim et al | ZINC23357725 | |
| 176 | (2R,3R)-1,1-dioxo-2-(propan-2-yl)--thiolan-3-yl N-[(2S,3R)-4-[(2S)-2-(tert-butylcarbamoyl)-4-cyclohexylpiperazin-1-yl]-3-hydroxy-1-phenylbutan-2-yl]carbamate | HIV 1 |  | Kim et al | ZINC23357728 | |
| 177 | (2R,3R)-1,1-dioxo-2-(propan-2-yl)--thiolan-3-yl N-[(2S,3R)-4-[(2S)-2-(tert-butylcarbamoyl)-4-cyclopentylpiperazin-1-yl]-3-hydroxy-1-phenylbutan-2-yl]carbamate | HIV 1 |  | Kim et al | ZINC23357731 | |
| 178 | (2R,3R)-1,1-dioxo-2-(propan-2-yl)--thiolan-3-yl N-[(2S,3R)-4-[(2S)-2-(tert-butylcarbamoyl)-4-cyclobutylpiperazin-1-yl]-3-hydroxy-1-phenylbutan-2-yl]carbamate | HIV 1 |  | Kim et al | ZINC23357734 | |
| 179 | (2R,3R)-1,1-dioxo-2-(propan-2-yl)--thiolan-3-yl N-[(2S,3R)-4-[(2S)-2-(tert-butylcarbamoyl)-4-cyclohexylpiperazin-1-yl]-3-hydroxy-1-phenylbutan-2-yl]carbamate | HIV 1 | 10.1016/j.bmcl.2004.02.035 | Kim et al | ZINC23357728 | |
| 180 | (2R,3R)-1,1-dioxo-2-(propan-2-yl)--thiolan-3-yl N-[(2S,3R)-4-[(2S)-2-(tert-butylcarbamoyl)-4-cyclopentylpiperazin-1-yl]-3-hydroxy-1-phenylbutan-2-yl]carbamate | HIV 1 | 10.1016/j.bmcl.2004.02.035 | Kim et al | ZINC23357731 | |
| 181 | (2R,3R)-1,1-dioxo-2-(propan-2-yl)--thiolan-3-yl N-[(2S,3R)-4-[(2S)-2-(tert-butylcarbamoyl)-4-cyclobutylpiperazin-1-yl]-3-hydroxy-1-phenylbutan-2-yl]carbamate | HIV 1 | 10.1016/j.bmcl.2004.02.035 | Kim et al | ZINC23357734 | |
| 182 | (2R,3R)-1,1-dioxo-2-(propan-2-yl)--thiolan-3-yl N-[(2S,3R)-4-[(2S)-2-(tert-butylcarbamoyl)-4-cyclopropylpiperazin-1-yl]-3-hydroxy-1-phenylbutan-2-yl]carbamate | HIV 1 | 10.1016/j.bmcl.2004.02.035 | Kim et al | ZINC19366833 | |
| 183 | (2R,3R)-1,1-dioxo-2-(propan-2-yl)--thiolan-3-yl N-[(2S,3R)-4-[(2S)-2-(tert-butylcarbamoyl)-4-(oxan-4-yl)piperazin-1-yl]-3-hydroxy-1-phenylbutan-2-yl]carbamate | HIV 1 | 10.1016/j.bmcl.2004.02.035 | Kim et al | ZINC23357737 | |
| 184 | (2R,3R)-1,1-dioxo-2-(propan-2-yl)--thiolan-3-yl N-[(2S,3R)-4-[(2S)-2-(tert-butylcarbamoyl)-4-(thian-4-yl)piperazin-1-yl]-3-hydroxy-1-phenylbutan-2-yl]carbamate | HIV 1 | 10.1016/j.bmcl.2004.02.035 | Kim et al | ZINC26649249 | |
| 185 | (2R,3R)-1,1-dioxo-2-(propan-2-yl)--thiolan-3-yl N-[(2S,3R)-4-[(2S)-2-(tert-butylcarbamoyl)-4-(thiolan-3-yl)piperazin-1-yl]-3-hydroxy-1-phenylbutan-2-yl]carbamate | HIV 1 | 10.1016/j.bmcl.2004.02.035 | Kim et al | ZINC23357740 | |
| 186 | (2R,3R)-1,1-dioxo-2-(propan-2-yl)--thiolan-3-yl N-[(2S,3R)-4-[(2S)-2-(tert-butylcarbamoyl)-4-(2,3-dihydro-1H-inden-2-yl)piperazin-1-yl]-3-hydroxy-1-phenylbutan-2-yl]carbamate | HIV 1 | 10.1016/j.bmcl.2004.02.035 | Kim et al | ZINC26643690 | |
| 187 | (2R,3R)-1,1-dioxo-2-(propan-2-yl)--thiolan-3-yl N-[(2S,3R)-4-[(2S)-2-(tert-butylcarbamoyl)-4-(3-phenylcyclobutyl)piperazin-1-yl]-3-hydroxy-1-phenylbutan-2-yl]carbamate | HIV 1 | 10.1016/j.bmcl.2004.02.035 | Kim et al | ZINC26649254 | |
| 188 | (2S,3S)-2-methyloxolan-3-yl N-[(2R,3R)-4-[(3aR,7aS)-6-(tert-butylcarbamoyl)-octahydrothieno[3,2-c]pyridin-5-yl]-3-hydroxy-1-(phenylsulfanyl)butan-2-yl]carbamate | HIV 1 | 10.1016/j.bmcl.2005.01.004 | Hornback et al | ZINC14942840 | |
| 189 | (2R,3R)-2-methyloxolan-3-yl N-[(2R,3R)-4-[(3aR,7aS)-6-(tert-butylcarbamoyl)-octahydrothieno[3,2-c]pyridin-5-yl]-3-hydroxy-1-(phenylsulfanyl)butan-2-yl]carbamate | HIV 1 | 10.1016/j.bmcl.2005.01.004 | Hornback et al | ZINC14942842 | |
| 190 | (2R,3R)-2-methyl-1,1-dioxo--thiolan-3-yl N-[(2S,3R)-4-[(4aS,8aS)-3-(tert-butylcarbamoyl)-decahydroisoquinolin-2-yl]-3-hydroxy-1-phenylbutan-2-yl]carbamate | HIV 1 | 10.1016/j.bmcl.2005.01.004 | Hornback et al | ZINC14942848 | |
| 191 | (2R,3S)-2-methyl-1,1-dioxo--thiolan-3-yl N-[(2R,3R)-4-[(3aR,7aS)-6-(tert-butylcarbamoyl)-octahydrothieno[3,2-c]pyridin-5-yl]-3-hydroxy-1-(phenylsulfanyl)butan-2-yl]carbamate | HIV 1 | 10.1016/j.bmcl.2005.01.004 | Hornback et al | ZINC14942850 | |
| 192 | (2S,3R)-2-methyl-1,1-dioxo--thiolan-3-yl N-[(2R,3R)-4-[(3aR,7aS)-6-(tert-butylcarbamoyl)-octahydrothieno[3,2-c]pyridin-5-yl]-3-hydroxy-1-(phenylsulfanyl)butan-2-yl]carbamate | HIV 1 | 10.1016/j.bmcl.2005.01.004 | Hornback et al | ZINC14942852 | |
| 193 | (2S,3S)-2-methyl-1,1-dioxo--thiolan-3-yl N-[(2R,3R)-4-[(3aR,7aS)-6-(tert-butylcarbamoyl)-octahydrothieno[3,2-c]pyridin-5-yl]-3-hydroxy-1-(phenylsulfanyl)butan-2-yl]carbamate | HIV 1 | 10.1016/j.bmcl.2005.01.004 | Hornback et al | ZINC14942854 | |
| 194 | (2R,3R)-2-methyl-1,1-dioxo--thiolan-3-yl N-[(2R,3R)-4-[(3aR,7aS)-6-(tert-butylcarbamoyl)-octahydrothieno[3,2-c]pyridin-5-yl]-3-hydroxy-1-(phenylsulfanyl)butan-2-yl]carbamate | HIV 1 | 10.1016/j.bmcl.2005.01.004 | Hornback et al | ZINC14942856 | |
| 195 | (2S,3S)-1,1-dioxo-2-(propan-2-yl)--thiolan-3-yl N-[(2S,3R)-4-[(4aS,8aS)-3-(tert-butylcarbamoyl)-decahydroisoquinolin-2-yl]-3-hydroxy-1-phenylbutan-2-yl]carbamate | HIV 1 | 10.1016/j.bmcl.2005.01.004 | Hornback et al | ZINC14942858 | |
| 196 | (2R,3R)-1,1-dioxo-2-(propan-2-yl)--thiolan-3-yl N-[(2S,3R)-4-[(4aS,8aS)-3-(tert-butylcarbamoyl)-decahydroisoquinolin-2-yl]-3-hydroxy-1-phenylbutan-2-yl]carbamate | HIV 1 |  | Hornback et al | ZINC14263864 | |
| 197 | (2S,3S)-2-(propan-2-yl)thiolan-3-yl N-[(2S,3R)-4-[(3aR,7aS)-6-(tert-butylcarbamoyl)-octahydrothieno[3,2-c]pyridin-5-yl]-3-hydroxy-1-phenylbutan-2-yl]carbamate | HIV 1 |  | Hornback et al | ZINC14942860 | |
| 198 | (2S,3S)-1,1-dioxo-2-(propan-2-yl)--thiolan-3-yl N-[(2S,3R)-4-[(3aR,7aS)-6-(tert-butylcarbamoyl)-octahydrothieno[3,2-c]pyridin-5-yl]-3-hydroxy-1-phenylbutan-2-yl]carbamate | HIV 1 |  | Hornback et al | ZINC14942864 | |
| 199 | (2R,3R)-1,1-dioxo-2-(propan-2-yl)--thiolan-3-yl N-[(2S,3R)-4-[(3aR,7aS)-6-(tert-butylcarbamoyl)-octahydrothieno[3,2-c]pyridin-5-yl]-3-hydroxy-1-phenylbutan-2-yl]carbamate | HIV 1 |  | Hornback et al | ZINC14942866 | |
| 200 | (2S,3S)-1,1-dioxo-2-(propan-2-yl)--thiolan-3-yl N-[(2R,3R)-4-[(3aR,7aS)-6-(tert-butylcarbamoyl)-octahydrothieno[3,2-c]pyridin-5-yl]-3-hydroxy-1-(phenylsulfanyl)butan-2-yl]carbamate | HIV 1 |  | Hornback et al | ZINC96126689 | |
| 201 | (2R,3R)-1,1-dioxo-2-(propan-2-yl)--thiolan-3-yl N-[(2R,3R)-4-[(3aR,7aS)-6-(tert-butylcarbamoyl)-octahydrothieno[3,2-c]pyridin-5-yl]-3-hydroxy-1-(phenylsulfanyl)butan-2-yl]carbamate | HIV 1 |  | Hornback et al | ZINC96126691 | |
| 202 | (3R,4S,5S,6R)-2,7-Bismethyl-3,6-bis(phenoxymethyl)-4,5-dihydroxy-1,2,7-thiadiazepine 1,1-Dioxide::(3R,4S,5S,6R)-4,5-dihydroxy-2,7-dimethyl-3,6-bis(phenoxymethyl)-1$l^{6},2,7-thiadiazepane-1,1-dione | HIV 1 |  | Schaal et al | ZINC13861443 | |
| 203 | (3R,4S,5S,6R)-2,7-Bis[(N-methylcarbamoyl)methyl]-3,6-bis(phenoxymethyl)-4,5-dihydroxy-1,2,7-thiadiazepine 1,1-Dioxide | HIV 1 |  | Schaal et al | ZINC14942867 | |
| 204 | (3R,4S,5S,6R)-3,6-Bis(phenoxymethyl)-4,5-dihydroxy-2,7-bis[(N-2-thiazolylcarbamoyl)methyl]-1,2,7-thiadiazepine 1,1-Dioxide | HIV 1 |  | Schaal et al | ZINC27618082 | |
| 205 | (3R,4S,5S,6R)-2,7-Bis[3-(N-methylcarbamoyl)benzyl]-3,6-bis(phenoxymethyl)-4,5-dihydroxy-1,2,7-thiadiazepine 1,1-Dioxide | HIV 1 |  | Schaal et al | ZINC27621833 | |
| 206 | (3R,4S,5S,6R)-2,7-Bis[3-Iodobenzyl]-3,6-bis(phenoxymethyl)-4,5-dihydroxy-1,2,7-thiadiazepine 1,1-Dioxide | HIV 1 |  | Schaal et al | ZINC95614939 | |
| 207 | (3R,4S,5S,6R)-3,6-Bis(phenoxymethyl)-2,7-bis[3-(2-thienyl)benzyl]-4,5-dihydroxy-1,2,7-thiadiazepine 1,1-Dioxide | HIV 1 |  | Schaal et al | ZINC96126693 | |
| 208 | (3R,4S,5S,6R)-3,6-Bis(phenoxymethyl)-2,7-bis[4-(2-thienyl)benzyl]-4,5-dihydroxy-1,2,7-thiadiazepine 1,1-Dioxide | HIV 1 |  | Schaal et al | ZINC95612116 | |
| 209 | (3R,4S,5S,6R)-2-benzyl-4,5-dihydroxy-7-methyl-3,6-bis(phenoxymethyl)-1$l^{6},2,7-thiadiazepane-1,1-dione | HIV 1 |  | Schaal et al | ZINC13861444 | |
| 210 | (3R,4S,5S,6R)-3,6-Bis(phenoxymethyl)-4,5-dihydroxy-2-[(N-methylcarbamoyl)methyl]-7-benzyl-1,2,7-thiadiazepine 1,1-Dioxide | HIV 1 |  | Schaal et al | ZINC14942868 | |
| 211 | (3R,4S,5S,6R)-3,6-Bis(phenoxymethyl)-4,5-dihydroxy-7-benzyl-2-[(N-2-thiazolylcarbamoyl)methyl]-1,2,7-thiadiazepine 1,1-Dioxide | HIV 1 |  | Schaal et al | ZINC14942869 | |
| 212 | (3R,4S,5S,6R)-2-benzyl-4,5-dihydroxy-3,6-bis(phenoxymethyl)-7-{[4-(thiophen-2-yl)phenyl]methyl}-1$l^{6},2,7-thiadiazepane-1,1-dione | HIV 1 |  | Schaal et al | ZINC96126696 | |
| 213 | (3R,4S,5S,6R)-2-benzyl-4,5-dihydroxy-3,6-bis(phenoxymethyl)-7-{[3-(thiophen-2-yl)phenyl]methyl}-1$l^{6},2,7-thiadiazepane-1,1-dione | HIV 1 |  | Schaal et al | ZINC96126698 | |
| 214 | (3R,4S,5S,6R)-3,6-Bis(phenoxymethyl)-4,5-dihydroxy-2-[3-(N-methylcarbamoyl)benzyl]-7-benzyl-1,2,7-thiadiazepine 1,1-Dioxide | HIV 1 |  | Schaal et al | ZINC03833854 | |
| 215 | (3R,4S,5S,6R)-3,6-Bis(phenoxymethyl)-4,5-dihydroxy-7-(3-iodobenzyl)-2-[(N-2-thiazolylcarbamoyl)methyl]-1,2,7-thiadiazepine 1,1-Dioxide | HIV 1 |  | Schaal et al | ZINC96126699 | |
| 216 | (3R,4S,5S,6R)-3,6-Bis(phenoxymethyl)-4,5-dihydroxy-2-[(N-2-thiazolylcarbamoyl)methyl]-7-[3-(2-thienyl)benzyl]-1,2,7-thiadiazepine 1,1-Dioxide | HIV 1 |  | Schaal et al | ZINC96126702 | |
| 217 | (3R,4S,5S,6R)-3,6-Bis(benzyl)-4,5-dihydroxy-2-[(N-2-thiazolylcarbamoyl)methyl]-7-[4-(2-thienyl)benzyl]-1,2,7-thiadiazepine 1,1-Dioxide | HIV 1 |  | Schaal et al | ZINC27618076 | |
| 218 | (3R,4S,5S,6R)-3,6-Bis(phenoxymethyl)-4,5-dihydroxy-2-[3-(2-thienyl)benzyl]-7-[4-(2-thienyl)benzyl]-1,2,7-thiadiazepine 1,1-Dioxide | HIV 1 |  | Schaal et al | ZINC96126704 | |
| 219 | (3R,4S,5S,6R)-3,6-Bis(phenoxymethyl)-4,5-dihydroxy-7-[4-(hydroxymethyl)benzyl]-2-[4-(methoxycarbonyl)benzyl]-1,2,7-thiadiazepine-1,1-Dioxide | HIV 1 |  | Schaal et al | ZINC96126707 | |
| 220 | (3R,4S,5S,6R)-2,7-Dibenzyl-3,6-bis(phenoxymethyl)-4,5-dihydroxy-1,2,7-thiadiazepan 1,1-Dioxide | HIV 1 |  | Schaal et al | ZINC03833860 | |
| 221 | (3R,4S,5S,6R)-4,5-dihydroxy-2,7-bis({3-[(1E)-1-(hydroxyimino)ethyl]phenyl}methyl)-3,6-bis(phenoxymethyl)-1$l^{6},2,7-thiadiazepane-1,1-dione | HIV 1 |  | Schaal et al | ZINC27332515 | |
| 222 | (3R,4S,5S,6R)-2,7-bis[(3-acetylphenyl)methyl]-4,5-dihydroxy-3,6-bis(phenoxymethyl)-1$l^{6},2,7-thiadiazepane-1,1-dione | HIV 1 |  | Schaal et al | ZINC27323467 | |
| 223 | (3R,4S,5S,6R)-4,5-dihydroxy-2,7-bis({[3-(2-hydroxyethyl)phenyl]methyl})-3,6-bis(phenoxymethyl)-1$l^{6},2,7-thiadiazepane-1,1-dione | HIV 1 |  | Schaal et al | ZINC27329575 | |
| 224 | (3R,4S,5S,6R)-2,7-bis({[3-(2,2-dimethoxyethyl)phenyl]methyl})-4,5-dihydroxy-3,6-bis(phenoxymethyl)-1$l^{6},2,7-thiadiazepane-1,1-dione | HIV 1 |  | Schaal et al | ZINC95548342 | |
| 225 | (3R,4S,5S,6R)-4,5-dihydroxy-2,7-bis({[4-(hydroxymethyl)phenyl]methyl})-3,6-bis(phenoxymethyl)-1$l^{6},2,7-thiadiazepane-1,1-dione | HIV 1 |  | Schaal et al | ZINC27329895 | |
| 226 | (2R,3R,4R,5R)-2,5-bis(benzyloxy)-3,4-dihydroxy-N,N'-bis[(1S)-2-methyl-1-(methylcarbamoyl)propyl]hexanediamide | HIV 1 |  | Pyring et al | ZINC14942870 | |
| 227 | (2R,3R,4R,5R)-2,5-bis[(2-fluorophenyl)methoxy]-3,4-dihydroxy-N,N'-bis[(1S)-2-methyl-1-(methylcarbamoyl)propyl]hexanediamide | HIV 1 |  | Pyring et al | ZINC26835262 | |
| 228 | (2R,3R,4R,5R)-2,5-bis[(3-fluorophenyl)methoxy]-3,4-dihydroxy-N,N'-bis[(1S)-2-methyl-1-(methylcarbamoyl)propyl]hexanediamide | HIV 1 |  | Pyring et al | ZINC26828270 | |
| 229 | (2R,3R,4R,5R)-2,5-bis[(4-fluorophenyl)methoxy]-3,4-dihydroxy-N,N'-bis[(1S)-2-methyl-1-(methylcarbamoyl)propyl]hexanediamide | HIV 1 |  | Pyring et al | ZINC26836508 | |
| 230 | (2R,3R,4R,5R)-2,5-bis[(2,3-difluorophenyl)methoxy]-3,4-dihydroxy-N,N'-bis[(1S)-2-methyl-1-(methylcarbamoyl)propyl]hexanediamide | HIV 1 |  | Pyring et al | ZINC26834085 | |
| 231 | (2R,3R,4R,5R)-2,5-bis[(2,4-difluorophenyl)methoxy]-3,4-dihydroxy-N,N'-bis[(1S)-2-methyl-1-(methylcarbamoyl)propyl]hexanediamide | HIV 1 |  | Pyring et al | ZINC26839627 | |
| 232 | (2R,3R,4R,5R)-2,5-bis[(2,5-difluorophenyl)methoxy]-3,4-dihydroxy-N,N'-bis[(1S)-2-methyl-1-(methylcarbamoyl)propyl]hexanediamide | HIV 1 |  | Pyring et al | ZINC26834066 | |
| 233 | (2R,3R,4R,5R)-2,5-bis[(2,6-difluorophenyl)methoxy]-3,4-dihydroxy-N,N'-bis[(1S)-2-methyl-1-(methylcarbamoyl)propyl]hexanediamide | HIV 1 |  | Pyring et al | ZINC26832387 | |
| 234 | (2R,3R,4R,5R)-2,5-bis[(3,4-difluorophenyl)methoxy]-3,4-dihydroxy-N,N'-bis[(1S)-2-methyl-1-(methylcarbamoyl)propyl]hexanediamide | HIV 1 |  | Pyring et al | ZINC26834061 | |
| 235 | (2R,3R,4R,5R)-2,5-bis[(3,5-difluorophenyl)methoxy]-3,4-dihydroxy-N,N'-bis[(1S)-2-methyl-1-(methylcarbamoyl)propyl]hexanediamide | HIV 1 |  | Pyring et al | ZINC26832691 | |
| 236 | (2R,3R,4R,5R)-2,5-bis(benzyloxy)-3,4-dihydroxy-N,N'-bis[(1S,2R)-2-hydroxy-2,3-dihydro-1H-inden-1-yl]hexanediamide | HIV 1 |  | Pyring et al | ZINC04424152 | |
| 237 | (2R,3R,4R,5R)-2,5-bis[(2-fluorophenyl)methoxy]-3,4-dihydroxy-N,N'-bis[(1S,2R)-2-hydroxy-2,3-dihydro-1H-inden-1-yl]hexanediamide | HIV 1 |  | Pyring et al | ZINC24495662 | |
| 238 | (2R,3R,4R,5R)-2,5-bis[(3-fluorophenyl)methoxy]-3,4-dihydroxy-N,N'-bis[(1S,2R)-2-hydroxy-2,3-dihydro-1H-inden-1-yl]hexanediamide | HIV 1 |  | Pyring et al | ZINC24691918 | |
| 239 | (2R,3R,4R,5R)-2,5-bis[(4-fluorophenyl)methoxy]-3,4-dihydroxy-N,N'-bis[(1S,2R)-2-hydroxy-2,3-dihydro-1H-inden-1-yl]hexanediamide | HIV 1 |  | Pyring et al | ZINC26838072 | |
| 240 | (2R,3R,4R,5R)-2,5-bis[(2,3-difluorophenyl)methoxy]-3,4-dihydroxy-N,N'-bis[(1S,2R)-2-hydroxy-2,3-dihydro-1H-inden-1-yl]hexanediamide | HIV 1 |  | Pyring et al | ZINC24691948 | |
| 241 | (2R,3R,4R,5R)-2,5-bis[(2,4-difluorophenyl)methoxy]-3,4-dihydroxy-N,N'-bis[(1S,2R)-2-hydroxy-2,3-dihydro-1H-inden-1-yl]hexanediamide | HIV 1 |  | Pyring et al | ZINC24691913 | |
| 242 | (2R,3R,4R,5R)-2,5-bis[(2,5-difluorophenyl)methoxy]-3,4-dihydroxy-N,N'-bis[(1S,2R)-2-hydroxy-2,3-dihydro-1H-inden-1-yl]hexanediamide | HIV 1 |  | Pyring et al | ZINC24692160 | |
| 243 | (2R,3R,4R,5R)-2,5-bis[(2,6-difluorophenyl)methoxy]-3,4-dihydroxy-N,N'-bis[(1S,2R)-2-hydroxy-2,3-dihydro-1H-inden-1-yl]hexanediamide | HIV 1 |  | Pyring et al | ZINC26824821 | |
| 244 | (2R,3R,4R,5R)-2,5-bis[(3,4-difluorophenyl)methoxy]-3,4-dihydroxy-N,N'-bis[(1S,2R)-2-hydroxy-2,3-dihydro-1H-inden-1-yl]hexanediamide | HIV 1 |  | Pyring et al | ZINC26829493 | |
| 245 | (2R,3R,4R,5R)-2,5-bis[(3,5-difluorophenyl)methoxy]-3,4-dihydroxy-N,N'-bis[(1S,2R)-2-hydroxy-2,3-dihydro-1H-inden-1-yl]hexanediamide | HIV 1 |  | Pyring et al | ZINC26824267 | |
| 246 | (3S)-oxolan-3-yl N-[(2S,3R)-4-[(4aS,8aS)-3-(tert-butylcarbamoyl)-decahydroisoquinolin-2-yl]-3-hydroxy-1-phenylbutan-2-yl]carbamate | HIV 1 |  | Ghosh et al | ZINC14942871 | |
| 247 | (2S,3S)-2-methyloxolan-3-yl N-[(2S,3R)-4-[(4aS,8aS)-3-(tert-butylcarbamoyl)-decahydroisoquinolin-2-yl]-3-hydroxy-1-phenylbutan-2-yl]carbamate | HIV 1 |  | Ghosh et al | ZINC14942878 | |
| 248 | (2R,3R)-2-methyloxolan-3-yl N-[(2S,3R)-4-[(4aS,8aS)-3-(tert-butylcarbamoyl)-decahydroisoquinolin-2-yl]-3-hydroxy-1-phenylbutan-2-yl]carbamate | HIV 1 |  | Ghosh et al | ZINC14942880 | |
| 249 | (2S,3S)-2-methyl-1,1-dioxo-1$l^{6}-thiolan-3-yl N-[(2S,3R)-4-[(4aS,8aS)-3-(tert-butylcarbamoyl)-decahydroisoquinolin-2-yl]-3-hydroxy-1-phenylbutan-2-yl]carbamate | HIV 1 |  | Ghosh et al | ZINC14942882 | |
| 250 | (2R,3R)-2-methyl-1,1-dioxo-1$l^{6}-thiolan-3-yl N-[(2S,3R)-4-[(4aS,8aS)-3-(tert-butylcarbamoyl)-decahydroisoquinolin-2-yl]-3-hydroxy-1-phenylbutan-2-yl]carbamate | HIV 1 |  | Ghosh et al | ZINC14942848 | |
| 251 | (2S,3S)-2-ethyl-1,1-dioxo-1$l^{6}-thiolan-3-yl N-[(2S,3R)-4-[(4aS,8aS)-3-(tert-butylcarbamoyl)-decahydroisoquinolin-2-yl]-3-hydroxy-1-phenylbutan-2-yl]carbamate | HIV 1 |  | Ghosh et al | ZINC14942884 | |
| 252 | (2R,3R)-2-ethyl-1,1-dioxo-1$l^{6}-thiolan-3-yl N-[(2S,3R)-4-[(4aS,8aS)-3-(tert-butylcarbamoyl)-decahydroisoquinolin-2-yl]-3-hydroxy-1-phenylbutan-2-yl]carbamate | HIV 1 |  | Ghosh et al | ZINC14942886 | |
| 253 | (2S,3S)-1,1-dioxo-2-(propan-2-yl)-1$l^{6}-thiolan-3-yl N-[(2S,3R)-4-[(4aS,8aS)-3-(tert-butylcarbamoyl)-decahydroisoquinolin-2-yl]-3-hydroxy-1-phenylbutan-2-yl]carbamate | HIV 1 |  | Ghosh et al | ZINC14942858 | |
| 254 | (2R,3R)-1,1-dioxo-2-(propan-2-yl)-1$l^{6}-thiolan-3-yl N-[(2S,3R)-4-[(4aS,8aS)-3-(tert-butylcarbamoyl)-decahydroisoquinolin-2-yl]-3-hydroxy-1-phenylbutan-2-yl]carbamate | HIV 1 |  | Ghosh et al | ZINC14263864 | |
| 255 | (2S,3S)-2-(2-methylpropyl)-1,1-dioxo-1$l^{6}-thiolan-3-yl N-[(2S,3R)-4-[(4aS,8aS)-3-(tert-butylcarbamoyl)-decahydroisoquinolin-2-yl]-3-hydroxy-1-phenylbutan-2-yl]carbamate | HIV 1 |  | Ghosh et al | ZINC14942888 | |
| 256 | (3S)-oxolan-3-yl N-[(2S,3S,5R)-5-benzyl-3-hydroxy-5-{[(1S,2R)-2-hydroxy-2,3-dihydro-1H-inden-1-yl]carbamoyl}-1-phenylpentan-2-yl]carbamate | HIV 1 |  | Ghosh et al | ZINC06148171 | |
| 257 | (3R)-oxolan-3-yl N-[(2S,3S,5R)-5-benzyl-3-hydroxy-5-{[(1S,2R)-2-hydroxy-2,3-dihydro-1H-inden-1-yl]carbamoyl}-1-phenylpentan-2-yl]carbamate | HIV 1 |  | Ghosh et al | ZINC14942891 | |
| 258 | (3S)-oxan-3-yl N-[(2S,3S,5R)-5-benzyl-3-hydroxy-5-{[(1S,2R)-2-hydroxy-2,3-dihydro-1H-inden-1-yl]carbamoyl}-1-phenylpentan-2-yl]carbamate | HIV 1 |  | Ghosh et al | ZINC14942893 | |
| 259 | (3R)-oxan-3-yl N-[(2S,3S,5R)-5-benzyl-3-hydroxy-5-{[(1S,2R)-2-hydroxy-2,3-dihydro-1H-inden-1-yl]carbamoyl}-1-phenylpentan-2-yl]carbamate | HIV 1 |  | Ghosh et al | ZINC14942894 | |
| 260 | (3R)-oxolan-3-yl N-[(2S,3R)-4-[(4aS,8aS)-3-(tert-butylcarbamoyl)-decahydroisoquinolin-2-yl]-3-hydroxy-1-phenylbutan-2-yl]carbamate | HIV 1 |  | Ghosh et al | ZINC14942898 | |
| 261 | (3S)-oxan-3-yl N-[(2S,3R)-4-[(4aS,8aS)-3-(tert-butylcarbamoyl)-decahydroisoquinolin-2-yl]-3-hydroxy-1-phenylbutan-2-yl]carbamate | HIV 1 |  | Ghosh et al | ZINC14942904 | |
| 262 | (3R)-oxan-3-yl N-[(2S,3R)-4-[(4aS,8aS)-3-(tert-butylcarbamoyl)-decahydroisoquinolin-2-yl]-3-hydroxy-1-phenylbutan-2-yl]carbamate | HIV 1 |  | Ghosh et al | ZINC14942906 | |
| 263 | 3-[(4-amino-2-tert-butyl-5-methylphenyl)sulfanyl]-4-hydroxy-6-[2-(4-hydroxyphenyl)ethyl]-6-(propan-2-yl)-5,6-dihydro-2H-pyran-2-one | HIV 1 |  | n/a | ZINC29306160 | |
| 264 | 3-[(4-amino-2-tert-butyl-5-methylphenyl)sulfanyl]-4-hydroxy-6-[2-(4-hydroxyphenyl)ethyl]-6-methyl-5,6-dihydro-2H-pyran-2-one | HIV 1 |  | n/a | ZINC29253278 | |
| 265 | 3-[(4-amino-2-tert-butyl-5-methylphenyl)sulfanyl]-4-hydroxy-6-(2-phenylethyl)-6-(propan-2-yl)-5,6-dihydro-2H-pyran-2-one | HIV 1 |  | n/a | ZINC61959297 | |
| 266 | 3-[(2-tert-butyl-4-hydroxy-5-methylphenyl)sulfanyl]-4-hydroxy-6-[2-(4-hydroxyphenyl)ethyl]-6-(propan-2-yl)-5,6-dihydro-2H-pyran-2-one | HIV 1 |  | n/a | ZINC32037013 | |
| 267 | 5-tert-butyl-4-({4-hydroxy-6-[2-(4-hydroxyphenyl)ethyl]-2-oxo-6-(propan-2-yl)-5,6-dihydro-2H-pyran-3-yl}sulfanyl)-2-methylphenyl methanesulfonate | HIV 1 |  | n/a | ZINC27899058 | |
| 268 | 5-tert-butyl-4-({4-hydroxy-6-[2-(4-hydroxyphenyl)ethyl]-2-oxo-6-(propan-2-yl)-5,6-dihydro-2H-pyran-3-yl}sulfanyl)-2-methylphenyl benzenesulfonate | HIV 1 |  | n/a | ZINC27978854 | |
| 269 | 5-tert-butyl-4-({4-hydroxy-6-[2-(4-hydroxyphenyl)ethyl]-2-oxo-6-(propan-2-yl)-5,6-dihydro-2H-pyran-3-yl}sulfanyl)-2-methylphenyl 4-fluorobenzene-1-sulfonate | HIV 1 |  | n/a | ZINC27980846 | |
| 270 | 5-tert-butyl-4-({4-hydroxy-6-[2-(4-hydroxyphenyl)ethyl]-2-oxo-6-(propan-2-yl)-5,6-dihydro-2H-pyran-3-yl}sulfanyl)-2-methylphenyl 4-cyanobenzene-1-sulfonate | HIV 1 |  | n/a | ZINC27980019 | |
| 271 | 5-tert-butyl-4-({4-hydroxy-6-[2-(4-hydroxyphenyl)ethyl]-2-oxo-6-(propan-2-yl)-5,6-dihydro-2H-pyran-3-yl}sulfanyl)-2-methylphenyl thiophene-2-sulfonate | HIV 1 |  | n/a | ZINC27986501 | |
| 272 | 5-tert-butyl-4-({4-hydroxy-6-[2-(4-hydroxyphenyl)ethyl]-2-oxo-6-(propan-2-yl)-5,6-dihydro-2H-pyran-3-yl}sulfanyl)-2-methylphenyl 1-methyl-1H-imidazole-2-sulfonate | HIV 1 |  | n/a | ZINC70454627 | |
| 273 | 5-tert-butyl-4-({4-hydroxy-6-[2-(4-hydroxyphenyl)ethyl]-2-oxo-6-(propan-2-yl)-5,6-dihydro-2H-pyran-3-yl}sulfanyl)-2-methylphenyl pyridine-2-sulfonate | HIV 1 |  | n/a | ZINC27989655 | |
| 274 | 5-tert-butyl-4-({4-hydroxy-6-[2-(4-hydroxyphenyl)ethyl]-2-oxo-6-(propan-2-yl)-5,6-dihydro-2H-pyran-3-yl}sulfanyl)-2-methylphenyl pyridine-3-sulfonate | HIV 1 |  | n/a | ZINC27993875 | |
| 275 | 5-tert-butyl-4-({4-hydroxy-6-[2-(4-hydroxyphenyl)ethyl]-6-methyl-2-oxo-5,6-dihydro-2H-pyran-3-yl}sulfanyl)-2-methylphenyl benzenesulfonate | HIV 1 |  | n/a | ZINC27997320 | |
| 276 | 5-tert-butyl-4-({4-hydroxy-6-[2-(4-hydroxyphenyl)ethyl]-6-methyl-2-oxo-5,6-dihydro-2H-pyran-3-yl}sulfanyl)-2-methylphenyl 4-fluorobenzene-1-sulfonate | HIV 1 |  | n/a | ZINC27983632 | |
| 277 | 5-tert-butyl-4-({4-hydroxy-6-[2-(4-hydroxyphenyl)ethyl]-6-methyl-2-oxo-5,6-dihydro-2H-pyran-3-yl}sulfanyl)-2-methylphenyl 1-methyl-1H-imidazole-2-sulfonate | HIV 1 |  | n/a | ZINC70454629 | |
| 278 | 5-tert-butyl-4-({6-cyclohexyl-4-hydroxy-6-[2-(4-hydroxyphenyl)ethyl]-2-oxo-5,6-dihydro-2H-pyran-3-yl}sulfanyl)-2-methylphenyl benzenesulfonate | HIV 1 |  | n/a | ZINC96126909 | |
| 279 | 5-tert-butyl-4-({6-cyclohexyl-4-hydroxy-6-[2-(4-hydroxyphenyl)ethyl]-2-oxo-5,6-dihydro-2H-pyran-3-yl}sulfanyl)-2-methylphenyl 4-cyanobenzene-1-sulfonate | HIV 1 |  | n/a | ZINC96126911 | |
| 280 | 5-tert-butyl-4-({6-cyclohexyl-4-hydroxy-6-[2-(4-hydroxyphenyl)ethyl]-2-oxo-5,6-dihydro-2H-pyran-3-yl}sulfanyl)-2-methylphenyl 4-fluorobenzene-1-sulfonate | HIV 1 |  | n/a | ZINC96126913 | |
| 281 | 5-tert-butyl-4-({6-cyclohexyl-4-hydroxy-6-[2-(4-hydroxyphenyl)ethyl]-2-oxo-5,6-dihydro-2H-pyran-3-yl}sulfanyl)-2-methylphenyl 1-methyl-1H-imidazole-2-sulfonate | HIV 1 |  | n/a | ZINC96126739 | |
| 282 | 5-tert-butyl-4-({4-hydroxy-6-[2-(4-hydroxyphenyl)ethyl]-2-oxo-6-propyl-5,6-dihydro-2H-pyran-3-yl}sulfanyl)-2-methylphenyl 1-methyl-1H-imidazole-2-sulfonate | HIV 1 |  | n/a | ZINC70454658 | |
| 283 | 5-tert-butyl-4-({4-hydroxy-6-[2-(4-hydroxyphenyl)ethyl]-6-(2-methylpropyl)-2-oxo-5,6-dihydro-2H-pyran-3-yl}sulfanyl)-2-methylphenyl 1-methyl-1H-imidazole-2-sulfonate | HIV 1 |  | n/a | ZINC70454631 | |
| 284 | 3-[(2-tert-butyl-4-hydroxy-5-methylphenyl)sulfanyl]-4-hydroxy-6-(2-phenylethyl)-6-(propan-2-yl)-5,6-dihydro-2H-pyran-2-one | HIV 1 |  | n/a | ZINC61959299 | |
| 285 | 5-tert-butyl-4-{[4-hydroxy-2-oxo-6-(2-phenylethyl)-6-(propan-2-yl)-5,6-dihydro-2H-pyran-3-yl]sulfanyl}-2-methylphenyl 1-methyl-1H-imidazole-2-sulfonate | HIV 1 |  | n/a | ZINC70454633 | |
| 286 | 5-tert-butyl-4-{[4-hydroxy-2-oxo-6-(2-phenylethyl)-6-(propan-2-yl)-5,6-dihydro-2H-pyran-3-yl]sulfanyl}-2-methylphenyl pyridine-2-sulfonate | HIV 1 |  | n/a | ZINC27979613 | |
| 287 | 5-tert-butyl-4-{[4-hydroxy-2-oxo-6-(2-phenylethyl)-6-(propan-2-yl)-5,6-dihydro-2H-pyran-3-yl]sulfanyl}-2-methylphenyl pyridine-3-sulfonate | HIV 1 |  | n/a | ZINC27981927 | |
| 288 | 4-({6-[2-(4-aminophenyl)ethyl]-4-hydroxy-2-oxo-6-(propan-2-yl)-5,6-dihydro-2H-pyran-3-yl}sulfanyl)-5-tert-butyl-2-methylphenyl 4-cyanobenzene-1-sulfonate | HIV 1 |  | n/a | ZINC70454659 | |
| 289 | 4-({6-[2-(4-aminophenyl)ethyl]-4-hydroxy-2-oxo-6-(propan-2-yl)-5,6-dihydro-2H-pyran-3-yl}sulfanyl)-5-tert-butyl-2-methylphenyl 3-(pyridin-3-yl)benzene-1-sulfonate | HIV 1 |  | n/a | ZINC96126748 | |
| 290 | 4-({6-[2-(4-aminophenyl)ethyl]-4-hydroxy-2-oxo-6-(propan-2-yl)-5,6-dihydro-2H-pyran-3-yl}sulfanyl)-5-tert-butyl-2-methylphenyl 1-methyl-1H-imidazole-2-sulfonate | HIV 1 |  | n/a | ZINC70454635 | |
| 291 | 4-({6-[2-(4-aminophenyl)ethyl]-4-hydroxy-2-oxo-6-(propan-2-yl)-5,6-dihydro-2H-pyran-3-yl}sulfanyl)-5-tert-butyl-2-methylphenyl 2-(thiophen-2-yl)benzene-1-sulfonate | HIV 1 |  | n/a | ZINC96126752 | |
| 292 | 3-({2-tert-butyl-4-[(dimethylsulfamoyl)amino]-5-methylphenyl}sulfanyl)-4-hydroxy-6-[2-(4-hydroxyphenyl)ethyl]-6-(propan-2-yl)-5,6-dihydro-2H-pyran-2-one | HIV 1 |  | n/a | ZINC27980582 | |
| 293 | 3-({2-tert-butyl-4-[(ethylsulfamoyl)amino]-5-methylphenyl}sulfanyl)-4-hydroxy-6-[2-(4-hydroxyphenyl)ethyl]-6-(propan-2-yl)-5,6-dihydro-2H-pyran-2-one | HIV 1 |  | n/a | ZINC27994449 | |
| 294 | 3-({2-tert-butyl-4-[(ethylsulfamoyl)amino]-5-methylphenyl}sulfanyl)-4-hydroxy-6-(2-phenylethyl)-6-(propan-2-yl)-5,6-dihydro-2H-pyran-2-one | HIV 1 |  | n/a | ZINC64293151 | |
| 295 | 5-tert-butyl-4-({4-hydroxy-6-[2-(4-hydroxyphenyl)ethyl]-2-oxo-6-(propan-2-yl)-5,6-dihydro-2H-pyran-3-yl}sulfanyl)-2-methylphenyl N,N-dimethylsulfamate | HIV 1 |  | n/a | ZINC26376513 | |
| 296 | 5-tert-butyl-4-({4-hydroxy-6-[2-(4-hydroxyphenyl)ethyl]-2-oxo-6-(propan-2-yl)-5,6-dihydro-2H-pyran-3-yl}sulfanyl)-2-methylphenyl N-ethylsulfamate | HIV 1 |  | n/a | ZINC26285117 | |
| 297 | 4-({6-[2-(4-aminophenyl)ethyl]-4-hydroxy-2-oxo-6-(propan-2-yl)-5,6-dihydro-2H-pyran-3-yl}sulfanyl)-5-tert-butyl-2-methylphenyl N,N-dimethylsulfamate | HIV 1 |  | n/a | ZINC70454660 | |
| 298 | 4-({6-[2-(4-aminophenyl)ethyl]-4-hydroxy-2-oxo-6-(propan-2-yl)-5,6-dihydro-2H-pyran-3-yl}sulfanyl)-5-tert-butyl-2-methylphenyl N-ethylsulfamate | HIV 1 |  | n/a | ZINC70454661 | |
| 299 | 5-tert-butyl-4-{[4-hydroxy-2-oxo-6-(2-phenylethyl)-6-(propan-2-yl)-5,6-dihydro-2H-pyran-3-yl]sulfanyl}-2-methylphenyl N-ethylsulfamate | HIV 1 |  | n/a | ZINC49799685 | |
| 300 | 5-tert-butyl-4-{[(6R)-4-hydroxy-2-oxo-6-(2-phenylethyl)-6-(propan-2-yl)-5,6-dihydro-2H-pyran-3-yl]sulfanyl}-2-methylphenyl 4-cyanobenzene-1-sulfonate | HIV 1 |  | n/a | ZINC27994392 | |
| 301 | 5-tert-butyl-4-{[(6S)-4-hydroxy-6-[2-(4-hydroxyphenyl)ethyl]-2-oxo-6-(propan-2-yl)-5,6-dihydro-2H-pyran-3-yl]sulfanyl}-2-methylphenyl methanesulfonate | HIV 1 |  | n/a |  | |
| 302 | benzyl N-[(1S)-2-carbamoyl-1-{[(2S,3R)-3-hydroxy-4-[methyl(methylcarbamoyl)amino]-1-phenylbutan-2-yl]carbamoyl}ethyl]carbamate | HIV 1 |  | Getman et al | ZINC14943003 | |
| 303 | benzyl N-[(1S)-1-{[(2S,3R)-4-[(butylcarbamoyl)(methyl)amino]-3-hydroxy-1-phenylbutan-2-yl]carbamoyl}-2-carbamoylethyl]carbamate | HIV 1 |  | Getman et al | ZINC14943004 | |
| 304 | benzyl N-[(1S)-2-carbamoyl-1-{[(2S,3R)-3-hydroxy-4-[(methylcarbamoyl)(2-methylpropyl)amino]-1-phenylbutan-2-yl]carbamoyl}ethyl]carbamate | HIV 1 |  | Getman et al | ZINC14943005 | |
| 305 | benzyl N-[(1S)-1-{[(2S,3R)-4-[(butylcarbamoyl)(2-methylpropyl)amino]-3-hydroxy-1-phenylbutan-2-yl]carbamoyl}-2-carbamoylethyl]carbamate | HIV 1 |  | Getman et al | ZINC14943006 | |
| 306 | (2S)-N-[(2S,3R)-4-[(butylcarbamoyl)(2-methylpropyl)amino]-3-hydroxy-1-phenylbutan-2-yl]-2-(quinolin-2-ylformamido)butanediamide | HIV 1 |  | Getman et al | ZINC14943007 | |
| 307 | benzyl N-[(1S)-2-carbamoyl-1-{[(2S,3R)-3-hydroxy-4-[(2-methylpropyl)(propylcarbamoyl)amino]-1-phenylbutan-2-yl]carbamoyl}ethyl]carbamate | HIV 1 |  | Getman et al | ZINC14943008 | |
| 308 | benzyl N-[(1S)-2-carbamoyl-1-{[(2S,3R)-4-[(ethylcarbamoyl)(2-methylpropyl)amino]-3-hydroxy-1-phenylbutan-2-yl]carbamoyl}ethyl]carbamate | HIV 1 |  | Getman et al | ZINC14943009 | |
| 309 | benzyl N-[(1S)-2-carbamoyl-1-{[(2S,3R)-3-hydroxy-4-[(2-methylpropyl)(propan-2-ylcarbamoyl)amino]-1-phenylbutan-2-yl]carbamoyl}ethyl]carbamate | HIV 1 |  | Getman et al | ZINC14943010 | |
| 310 | benzyl N-[(1S)-1-{[(2S,3R)-4-[(tert-butylcarbamoyl)(2-methylpropyl)amino]-3-hydroxy-1-phenylbutan-2-yl]carbamoyl}-2-carbamoylethyl]carbamate | HIV 1 |  | Getman et al | ZINC14943011 | |
| 311 | benzyl N-[(1S)-1-{[(2S,3S)-4-[(tert-butylcarbamoyl)(2-methylpropyl)amino]-3-hydroxy-1-phenylbutan-2-yl]carbamoyl}-2-carbamoylethyl]carbamate | HIV 1 |  | Getman et al | ZINC14943012 | |
| 312 | (2S)-N-[(2S,3R)-4-[(tert-butylcarbamoyl)(2-methylpropyl)amino]-3-hydroxy-1-phenylbutan-2-yl]-2-(quinolin-2-ylformamido)butanediamide | HIV 1 |  | Getman et al | ZINC03915259 | |
| 313 | (2S)-N-[(2S,3S)-4-[(tert-butylcarbamoyl)(2-methylpropyl)amino]-3-hydroxy-1-phenylbutan-2-yl]-2-(quinolin-2-ylformamido)butanediamide | HIV 1 |  | Getman et al | ZINC14943013 | |
| 314 | benzyl N-[(1S)-1-{[(2S,3R)-4-[(tert-butylcarbamoyl)(3-methylbutyl)amino]-3-hydroxy-1-phenylbutan-2-yl]carbamoyl}-2-carbamoylethyl]carbamate | HIV 1 |  | Getman et al | ZINC14943014 | |
| 315 | (2S)-N-[(2S,3R)-4-[(tert-butylcarbamoyl)(3-methylbutyl)amino]-3-hydroxy-1-phenylbutan-2-yl]-2-(quinolin-2-ylformamido)butanediamide | HIV 1 |  | Getman et al | ZINC14943015 | |
| 316 | benzyl N-[(1S)-1-{[(2S,3R)-4-[(tert-butylcarbamoyl)(cyclohexylmethyl)amino]-3-hydroxy-1-phenylbutan-2-yl]carbamoyl}-2-carbamoylethyl]carbamate | HIV 1 |  | Getman et al | ZINC14943016 | |
| 317 | (2S)-N-[(2S,3R)-4-[(tert-butylcarbamoyl)(cyclohexylmethyl)amino]-3-hydroxy-1-phenylbutan-2-yl]-2-(quinolin-2-ylformamido)butanediamide | HIV 1 |  | Getman et al | ZINC14943017 | |
| 318 | benzyl N-[(1S)-1-{[(2S,3R)-4-[benzyl(tert-butylcarbamoyl)amino]-3-hydroxy-1-phenylbutan-2-yl]carbamoyl}-2-carbamoylethyl]carbamate | HIV 1 |  | Getman et al | ZINC14943018 | |
| 319 | (2S)-N-[(2S,3R)-4-[benzyl(tert-butylcarbamoyl)amino]-3-hydroxy-1-phenylbutan-2-yl]-2-(quinolin-2-ylformamido)butanediamide | HIV 1 |  | Getman et al | ZINC14943019 | |
| 320 | benzyl N-[(1S)-1-{[(2S,3R)-4-[(tert-butylcarbamoyl)[(1R)-1-phenylethyl]amino]-3-hydroxy-1-phenylbutan-2-yl]carbamoyl}-2-carbamoylethyl]carbamate | HIV 1 |  | Getman et al | ZINC14943020 | |
| 321 | benzyl N-[(1S)-1-{[(2S,3R)-4-[(tert-butylcarbamoyl)[(1S)-1-phenylethyl]amino]-3-hydroxy-1-phenylbutan-2-yl]carbamoyl}-2-carbamoylethyl]carbamate | HIV 1 |  | Getman et al | ZINC14943021 | |
| 322 | benzyl N-[(1S)-1-{[(2S,3R)-4-[(tert-butylcarbamoyl)(pyridin-4-ylmethyl)amino]-3-hydroxy-1-phenylbutan-2-yl]carbamoyl}-2-carbamoylethyl]carbamate | HIV 1 |  | Getman et al | ZINC14943022 | |
| 323 | (2S)-N-[(2S,3R)-4-[(tert-butylcarbamoyl)(pyridin-4-ylmethyl)amino]-3-hydroxy-1-phenylbutan-2-yl]-2-(quinolin-2-ylformamido)butanediamide | HIV 1 |  | Getman et al | ZINC26283325 | |
| 324 | benzyl N-[(1S)-2-carbamoyl-1-{[(2S,3S)-3-hydroxy-4-[methyl(methylcarbamoyl)amino]-1-phenylbutan-2-yl]carbamoyl}ethyl]carbamate | HIV 1 |  | Getman et al | ZINC14943023 | |
| 325 | benzyl N-[(1S)-1-{[(2S,3S)-4-[(butylcarbamoyl)(methyl)amino]-3-hydroxy-1-phenylbutan-2-yl]carbamoyl}-2-carbamoylethyl]carbamate | HIV 1 |  | Getman et al | ZINC14943024 | |
| 326 | benzyl N-[(1S)-2-carbamoyl-1-{[(2S,3S)-3-hydroxy-4-[(methylcarbamoyl)(2-methylpropyl)amino]-1-phenylbutan-2-yl]carbamoyl}ethyl]carbamate | HIV 1 |  | Getman et al | ZINC14943025 | |
| 327 | benzyl N-[(1S)-1-{[(2S,3S)-4-[(butylcarbamoyl)(2-methylpropyl)amino]-3-hydroxy-1-phenylbutan-2-yl]carbamoyl}-2-carbamoylethyl]carbamate | HIV 1 |  | Getman et al | ZINC14943026 | |
| 328 | 4-hydroxy-3-[(4-hydroxy-2-oxo-3,4-dihydro-2H-1-benzopyran-3-yl)sulfanyl]-2H-chromen-2-one | HIV 1 | 10.1021/jm950874+ | Wang et al | ZINC13604221 | |
| 329 | 3-benzyl-4,5-dihydroxy-2H-chromen-2-one | HIV 1 | 10.1021/jm950874+ | Wang et al | ZINC06575285 | |
| 330 | 3-[2H-1,3-benzodioxol-5-yl(4-hydroxy-2-oxo-2H-chromen-3-yl)methyl]-4-hydroxy-2H-chromen-2-one | HIV 1 | 10.1021/jm950874+ | Wang et al | ZINC04512503 | |
| 331 | 3-({4-[bis(4-hydroxy-2-oxo-2H-chromen-3-yl)methyl]phenyl}(4-hydroxy-2-oxo-2H-chromen-3-yl)methyl)-4-hydroxy-2H-chromen-2-one | HIV 1 | 10.1021/jm950874+ | Wang et al | ZINC95543764 | |
| 332 | (2S,3R,4R,4aS,6aR,6bR,8aS,12aS,14aS,14bR)-2,3-dihydroxy-4,6a,6b,11,11,14b-hexamethyl-1,2,3,4,4a,5,6,6a,6b,7,8,8a,9,10,11,12,12a,14,14a,14b-icosahydropicene-4,8a-dicarboxylic acid | HIV 1 | 10.1021/jm950874+ | Wang et al | ZINC14943034 | |
| 333 | 4-hydroxy-3,8-diphenyl-2H-chromen-2-one | HIV 1 | 10.1021/jm950874+ | Wang et al | ZINC06575897 | |
| 334 | 4-hydroxy-3-phenyl-2H-chromen-2-one | HIV 1 | 10.1021/jm950874+ | Wang et al | ZINC00068728 | |
| 335 | (3S)-oxolan-3-yl N-[(2S,3R)-4-[(3S,4aS,7aS)-3-(tert-butylcarbamoyl)-octahydro-1H-cyclopenta[c]pyridin-2-yl]-3-hydroxy-1-phenylbutan-2-yl]carbamate | HIV 1 |  | Ghosh et al | ZINC14943036 | |
| 336 | (3S)-thiolan-3-yl N-[(2S,3R)-4-[(3S,4aS,8aS)-3-(tert-butylcarbamoyl)-decahydroisoquinolin-2-yl]-3-hydroxy-1-phenylbutan-2-yl]carbamate | HIV 1 |  | Ghosh et al | ZINC14943037 | |
| 337 | (3R)-thiolan-3-yl N-[(2S,3R)-4-[(3S,4aS,8aS)-3-(tert-butylcarbamoyl)-decahydroisoquinolin-2-yl]-3-hydroxy-1-phenylbutan-2-yl]carbamate | HIV 1 |  | Ghosh et al | ZINC14943038 | |
| 338 | (2S,3S)-2-methyloxolan-3-yl N-[(2S,3R)-4-[(3S,4aS,8aS)-3-(tert-butylcarbamoyl)-decahydroisoquinolin-2-yl]-3-hydroxy-1-phenylbutan-2-yl]carbamate | HIV 1 |  | Ghosh et al |  | |
| 339 | (2R,3R)-2-methyloxolan-3-yl N-[(2S,3R)-4-[(3S,4aS,8aS)-3-(tert-butylcarbamoyl)-decahydroisoquinolin-2-yl]-3-hydroxy-1-phenylbutan-2-yl]carbamate | HIV 1 |  | Ghosh et al |  | |
| 340 | (2S,3S)-2-methyl-1,1-dioxo-1$l^{6}-thiolan-3-yl N-[(2S,3R)-4-[(3S,4aS,8aS)-3-(tert-butylcarbamoyl)-decahydroisoquinolin-2-yl]-3-hydroxy-1-phenylbutan-2-yl]carbamate | HIV 1 |  | Ghosh et al |  | |
| 341 | (2S,3S)-2-methyl-1,1-dioxo-1$l^{6}-thiolan-3-yl N-[(2S,3R)-4-[(3S,4aS,7aS)-3-(tert-butylcarbamoyl)-octahydro-1H-cyclopenta[c]pyridin-2-yl]-3-hydroxy-1-phenylbutan-2-yl]carbamate | HIV 1 |  | Ghosh et al | ZINC14943043 | |
| 342 | (2R,3R)-2-methyl-1,1-dioxo-1$l^{6}-thiolan-3-yl N-[(2S,3R)-4-[(3S,4aS,8aS)-3-(tert-butylcarbamoyl)-decahydroisoquinolin-2-yl]-3-hydroxy-1-phenylbutan-2-yl]carbamate | HIV 1 |  | Ghosh et al |  | |
| 343 | (3S,4R)-4-methyl-1,1-dioxo-1$l^{6}-thiolan-3-yl N-[(2S,3R)-4-[(3S,4aS,8aS)-3-(tert-butylcarbamoyl)-decahydroisoquinolin-2-yl]-3-hydroxy-1-phenylbutan-2-yl]carbamate | HIV 1 |  | Ghosh et al | ZINC14943044 | |
| 344 | (3R,4S)-4-methyl-1,1-dioxo-1$l^{6}-thiolan-3-yl N-[(2S,3R)-4-[(3S,4aS,8aS)-3-(tert-butylcarbamoyl)-decahydroisoquinolin-2-yl]-3-hydroxy-1-phenylbutan-2-yl]carbamate | HIV 1 |  | Ghosh et al | ZINC14943045 | |
| 345 | (3S,5R)-5-methyl-1,1-dioxo-1$l^{6}-thiolan-3-yl N-[(2S,3R)-4-[(3S,4aS,8aS)-3-(tert-butylcarbamoyl)-decahydroisoquinolin-2-yl]-3-hydroxy-1-phenylbutan-2-yl]carbamate | HIV 1 |  | Ghosh et al | ZINC14943046 | |
| 346 | (3R,5S)-5-methyl-1,1-dioxo-1$l^{6}-thiolan-3-yl N-[(2S,3R)-4-[(3S,4aS,8aS)-3-(tert-butylcarbamoyl)-decahydroisoquinolin-2-yl]-3-hydroxy-1-phenylbutan-2-yl]carbamate | HIV 1 |  | Ghosh et al | ZINC14943047 | |
| 347 | (2S,3S)-1,1-dioxo-2-propyl-1$l^{6}-thiolan-3-yl N-[(2S,3R)-4-[(3S,4aS,8aS)-3-(tert-butylcarbamoyl)-decahydroisoquinolin-2-yl]-3-hydroxy-1-phenylbutan-2-yl]carbamate | HIV 1 |  | Ghosh et al | ZINC14943048 | |
| 348 | (2R,3R)-1,1-dioxo-2-propyl-1$l^{6}-thiolan-3-yl N-[(2S,3R)-4-[(3S,4aS,8aS)-3-(tert-butylcarbamoyl)-decahydroisoquinolin-2-yl]-3-hydroxy-1-phenylbutan-2-yl]carbamate | HIV 1 |  | Ghosh et al | ZINC14943049 | |
| 349 | (2R,3R)-1,1-dioxo-2-(propan-2-yl)-1$l^{6}-thiolan-3-yl N-[(2S,3R)-4-[(3S,4aS,7aS)-3-(tert-butylcarbamoyl)-octahydro-1H-cyclopenta[c]pyridin-2-yl]-3-hydroxy-1-phenylbutan-2-yl]carbamate | HIV 1 |  | Ghosh et al | ZINC14943050 | |
| 350 | (2S,3S)-2-cyclopentyl-1,1-dioxo-1$l^{6}-thiolan-3-yl N-[(2S,3R)-4-[(3S,4aS,8aS)-3-(tert-butylcarbamoyl)-decahydroisoquinolin-2-yl]-3-hydroxy-1-phenylbutan-2-yl]carbamate | HIV 1 |  | Ghosh et al | ZINC49605951 | |
| 351 | (2S,3S)-2-butyl-1,1-dioxo-1$l^{6}-thiolan-3-yl N-[(2S,3R)-4-[(3S,4aS,8aS)-3-(tert-butylcarbamoyl)-decahydroisoquinolin-2-yl]-3-hydroxy-1-phenylbutan-2-yl]carbamate | HIV 1 |  | Ghosh et al | ZINC14943051 | |
| 352 | 4-cyano-N-(3-{1-[4-hydroxy-2-oxo-6-(2-phenylethyl)-6-propyl-5,6-dihydro-2H-pyran-3-yl]propyl}phenyl)benzene-1-sulfonamide | HIV 1 | 10.1021/jm960541s | Thaisrivongs et al | ZINC36124704 | |
| 353 | 5-cyano-N-{3-[(1R)-1-[(6S)-4-hydroxy-2-oxo-6-(2-phenylethyl)-6-propyl-5,6-dihydro-2H-pyran-3-yl]propyl]phenyl}pyridine-2-sulfonamide | HIV 1 | 10.1021/jm960541s | Thaisrivongs et al | ZINC36124711 | |
| 354 | 5-cyano-N-{3-[(1R)-1-[(6R)-4-hydroxy-2-oxo-6-(2-phenylethyl)-6-propyl-5,6-dihydro-2H-pyran-3-yl]propyl]phenyl}pyridine-2-sulfonamide | HIV 1 | 10.1021/jm960541s | Thaisrivongs et al | ZINC70454636 | |
| 355 | 5-cyano-N-{3-[(1S)-1-[(6S)-4-hydroxy-2-oxo-6-(2-phenylethyl)-6-propyl-5,6-dihydro-2H-pyran-3-yl]propyl]phenyl}pyridine-2-sulfonamide | HIV 1 | 10.1021/jm960541s | Thaisrivongs et al | ZINC36124707 | |
| 356 | 5-cyano-N-{3-[(1S)-1-[(6R)-4-hydroxy-2-oxo-6-(2-phenylethyl)-6-propyl-5,6-dihydro-2H-pyran-3-yl]propyl]phenyl}pyridine-2-sulfonamide | HIV 1 | 10.1021/jm960541s | Thaisrivongs et al | ZINC08536339 | |
| 357 | (2S,3S)-2-(methoxymethyl)thiolan-3-yl N-[(2S,3R)-4-[(3S,4aS,8aS)-3-(tert-butylcarbamoyl)-decahydroisoquinolin-2-yl]-3-hydroxy-1-phenylbutan-2-yl]carbamate | HIV 1 | 10.1021/jm960128k | Ghosh et al | ZINC14943071 | |
| 358 | (2S,3S)-2-(methoxymethyl)-1,1-dioxo-1$l^{6}-thiolan-3-yl N-[(2S,3R)-4-[(3S,4aS,8aS)-3-(tert-butylcarbamoyl)-decahydroisoquinolin-2-yl]-3-hydroxy-1-phenylbutan-2-yl]carbamate | HIV 1 | 10.1021/jm960128k | Ghosh et al | ZINC14943072 | |
| 359 | (3R,3aS,6aR)-hexahydrofuro[2,3-b]furan-3-yl (1S,2R)-1-benzyl-3-[(3S,4aS,8aS)-3-[(tert-butylamino)carbonyl]octahydroisoquinolin-2(1H)-yl]-2-hydroxypropylcarbamate | HIV 1 | 10.1021/jm960128k | Ghosh et al | ZINC03944633 | |
| 360 | (3S,3aR,6aS)-hexahydrofuro[2,3-b]furan-3-yl N-[(2S,3R)-4-[(3S,4aS,8aS)-3-(tert-butylcarbamoyl)-decahydroisoquinolin-2-yl]-3-hydroxy-1-phenylbutan-2-yl]carbamate | HIV 1 | 10.1021/jm960128k | Ghosh et al | ZINC03930686 | |
| 361 | (3R,3aS,7aR)-hexahydro-2H-furo[2,3-b]pyran-3-yl N-[(2S,3R)-4-[(3S,4aS,8aS)-3-(tert-butylcarbamoyl)-decahydroisoquinolin-2-yl]-3-hydroxy-1-phenylbutan-2-yl]carbamate | HIV 1 | 10.1021/jm960128k | Ghosh et al | ZINC03930691 | |
| 362 | (3S,3aR,7aS)-hexahydro-2H-furo[2,3-b]pyran-3-yl N-[(2S,3R)-4-[(3S,4aS,8aS)-3-(tert-butylcarbamoyl)-decahydroisoquinolin-2-yl]-3-hydroxy-1-phenylbutan-2-yl]carbamate | HIV 1 | 10.1021/jm960128k | Ghosh et al | ZINC03930684 | |
| 363 | (3R,3aR,6aS)-hexahydro-2H-cyclopenta[b]furan-3-yl N-[(2S,3R)-4-[(3S,4aS,8aS)-3-(tert-butylcarbamoyl)-decahydroisoquinolin-2-yl]-3-hydroxy-1-phenylbutan-2-yl]carbamate | HIV 1 | 10.1021/jm960128k | Ghosh et al | ZINC14943073 | |
| 364 | (3S,4aS,8aS,2 R,3 S,3 aS,4 S,6 aR)-N-tert-Butyl-2-[2 -hydroxy-4 -phenyl-3 -[[[(4 -hexahydro-2H-cyclopenta[b]-furanyl)oxy]carbonyl]amino]butyl]decahydroiso-quinoline-3-carboxamide | HIV 1 | 10.1021/jm960128k | Ghosh et al | ZINC14943074 | |
| 365 | (3S,4aS,8aS,2 R,3 S)-N-tert-Butyl-2-[2 -hydroxy-4 -phenyl-3 -[[[(3a -hexahydrofuro[2,3-b]furanyl)oxy]carbonyl]-amino]butyl]decahydroisoquinoline-3-carboxamide | HIV 1 | 10.1021/jm960128k | Ghosh et al | ZINC14943075 | |
| 366 | (3R,3aR,6aR)-hexahydrofuro[2,3-c]furan-3-yl N-[(2S,3R)-4-[(3S,4aS,8aS)-3-(tert-butylcarbamoyl)-decahydroisoquinolin-2-yl]-3-hydroxy-1-phenylbutan-2-yl]carbamate | HIV 1 | 10.1021/jm960128k | Ghosh et al | ZINC14943076 | |
| 367 | (3R,3aS,6aR)-hexahydrofuro[3,2-b]furan-3-yl N-[(2S,3R)-4-[(3S,4aS,8aS)-3-(tert-butylcarbamoyl)-decahydroisoquinolin-2-yl]-3-hydroxy-1-phenylbutan-2-yl]carbamate | HIV 1 | 10.1021/jm960128k | Ghosh et al | ZINC14943077 | |
| 368 | (3R,3aS,6aR)-6a-methyl-hexahydrofuro[2,3-b]furan-3-yl N-[(2S,3R)-4-[(3S,4aS,8aS)-3-(tert-butylcarbamoyl)-decahydroisoquinolin-2-yl]-3-hydroxy-1-phenylbutan-2-yl]carbamate | HIV 1 | 10.1021/jm960128k | Ghosh et al | ZINC14943078 | |
| 369 | (3R,3aS,5R,6aS)-5-methyl-hexahydrofuro[2,3-b]furan-3-yl N-[(2S,3R)-4-[(3S,4aS,8aS)-3-(tert-butylcarbamoyl)-decahydroisoquinolin-2-yl]-3-hydroxy-1-phenylbutan-2-yl]carbamate | HIV 1 | 10.1021/jm960128k | Ghosh et al | ZINC14943079 | |
| 370 | (3R,3aS,5S,6aS)-5-methyl-hexahydrofuro[2,3-b]furan-3-yl N-[(2S,3R)-4-[(3S,4aS,8aS)-3-(tert-butylcarbamoyl)-decahydroisoquinolin-2-yl]-3-hydroxy-1-phenylbutan-2-yl]carbamate | HIV 1 | 10.1021/jm960128k | Ghosh et al | ZINC14943080 | |
| 371 | (2R,3R,4R,5R)-2,5-bis(benzyloxy)-3,4-dihydroxy-N,N'-bis[(1S,2R)-2-hydroxy-2,3-dihydro-1H-inden-1-yl]hexanediamide | HIV 1 |  | Muhlman et al | ZINC04424152 | |
| 372 | (2R,3R,4R,5R)-2,5-bis(benzyloxy)-3,4-dihydroxy-N,N'-bis[(1S)-2-methyl-1-(methylcarbamoyl)propyl]hexanediamide | HIV 1 |  | Muhlman et al | ZINC14942870 | |
| 373 | (2R,3R,4S,5R)-3,4-dihydroxy-N,N'-bis[(1S,2R)-2-hydroxy-2,3-dihydro-1H-inden-1-yl]-2,5-bis(2-phenylethyl)hexanediamide | HIV 1 |  | Muhlman et al | ZINC27087540 | |
| 374 | (2R,3R,4R,5R)-3,4-dihydroxy-N,N'-bis[(1S,2R)-2-hydroxy-2,3-dihydro-1H-inden-1-yl]-2,5-bis(2-phenylethyl)hexanediamide | HIV 1 |  | Muhlman et al | ZINC03964411 | |
| 375 | (2R,3R,4S,5R)-3,4-dihydroxy-N,N'-bis[(1S)-2-methyl-1-(methylcarbamoyl)propyl]-2,5-bis(2-phenylethyl)hexanediamide | HIV 1 |  | Muhlman et al | ZINC14943085 | |
| 376 | (2R,3R,4R,5R)-3,4-dihydroxy-N,N'-bis[(1S)-2-methyl-1-(methylcarbamoyl)propyl]-2,5-bis(2-phenylethyl)hexanediamide | HIV 1 |  | Muhlman et al | ZINC14943086 | |
| 377 | (2R,4S)-2-[(R)-carbamoyl(1-phenylacetamido)methyl]-N-(2-{[(2R,4S)-2-[(R)-carbamoyl(1-phenylacetamido)methyl]-5,5-dimethyl-1,3-thiazolidin-4-yl]formamido}ethyl)-5,5-dimethyl-1,3-thiazolidine-4-carboxamide | HIV 1 |  | Humber et al | ZINC26831133 | |
| 378 | (2R,4S)-2-[(R)-(ethylcarbamoyl)(1-phenylacetamido)methyl]-N-(2-{[(2R,4S)-2-[(R)-(ethylcarbamoyl)(1-phenylacetamido)methyl]-5,5-dimethyl-1,3-thiazolidin-4-yl]formamido}ethyl)-5,5-dimethyl-1,3-thiazolidine-4-carboxamide | HIV 1 |  | Humber et al | ZINC95542263 | |
| 379 | (2R,4S)-2-[(R)-(benzylcarbamoyl)(1-phenylacetamido)methyl]-N-(2-{[(2R,4S)-2-[(R)-(benzylcarbamoyl)(1-phenylacetamido)methyl]-5,5-dimethyl-1,3-thiazolidin-4-yl]formamido}ethyl)-5,5-dimethyl-1,3-thiazolidine-4-carboxamide | HIV 1 |  | Humber et al |  | |
| 380 | (2R,4S)-2-[(R)-(dimethylcarbamoyl)(1-phenylacetamido)methyl]-N-(2-{[(2R,4S)-2-[(R)-(dimethylcarbamoyl)(1-phenylacetamido)methyl]-5,5-dimethyl-1,3-thiazolidin-4-yl]formamido}ethyl)-5,5-dimethyl-1,3-thiazolidine-4-carboxamide | HIV 1 |  | Humber et al | ZINC95610744 | |
| 381 | (2R,4S)-N-(2-{[(2R,4S)-5,5-dimethyl-2-[(R)-(1-phenylacetamido)[(2,2,2-trifluoroethyl)carbamoyl]methyl]-1,3-thiazolidin-4-yl]formamido}ethyl)-5,5-dimethyl-2-[(R)-(1-phenylacetamido)[(2,2,2-trifluoroethyl)carbamoyl]methyl]-1,3-thiazolidine-4-carboxamide | HIV 1 |  | Humber et al |  | |
| 382 | (2R,4S)-N-(2-{[(2R,4S)-5,5-dimethyl-2-[(1R)-2-oxo-1-(1-phenylacetamido)-2-(piperidin-1-yl)ethyl]-1,3-thiazolidin-4-yl]formamido}ethyl)-5,5-dimethyl-2-[(1R)-2-oxo-1-(1-phenylacetamido)-2-(piperidin-1-yl)ethyl]-1,3-thiazolidine-4-carboxamide | HIV 1 |  | Humber et al |  | |
| 383 | (2R,4S)-2-[(R)-[(cyclohexylmethyl)carbamoyl](1-phenylacetamido)methyl]-N-(2-{[(2R,4S)-2-[(R)-[(cyclohexylmethyl)carbamoyl](1-phenylacetamido)methyl]-5,5-dimethyl-1,3-thiazolidin-4-yl]formamido}ethyl)-5,5-dimethyl-1,3-thiazolidine-4-carboxamide | HIV 1 |  | Humber et al |  | |
| 384 | (2R,4S)-2-[(R)-[benzyl(methyl)carbamoyl](1-phenylacetamido)methyl]-N-(2-{[(2R,4S)-2-[(R)-[benzyl(methyl)carbamoyl](1-phenylacetamido)methyl]-5,5-dimethyl-1,3-thiazolidin-4-yl]formamido}ethyl)-5,5-dimethyl-1,3-thiazolidine-4-carboxamide | HIV 1 |  | Humber et al |  | |
| 385 | (2R,4S)-2-[(R)-({[4-(dimethylamino)phenyl]methyl}carbamoyl)(1-phenylacetamido)methyl]-N-(2-{[(2R,4S)-2-[(R)-({[4-(dimethylamino)phenyl]methyl}carbamoyl)(1-phenylacetamido)methyl]-5,5-dimethyl-1,3-thiazolidin-4-yl]formamido}ethyl)-5,5-dimethyl-1,3-thiazolidine-4-carboxamide | HIV 1 |  | Humber et al |  | |
| 386 | (2R,4S)-N-(2-{[(2R,4S)-5,5-dimethyl-2-[(R)-(1-phenylacetamido)[(pyridin-2-ylmethyl)carbamoyl]methyl]-1,3-thiazolidin-4-yl]formamido}ethyl)-5,5-dimethyl-2-[(R)-(1-phenylacetamido)[(pyridin-2-ylmethyl)carbamoyl]methyl]-1,3-thiazolidine-4-carboxamide | HIV 1 |  | Humber et al |  | |
| 387 | (2R,4S)-2-[(R)-[(2-hydroxyethyl)carbamoyl](1-phenylacetamido)methyl]-N-(2-{[(2R,4S)-2-[(R)-[(2-hydroxyethyl)carbamoyl](1-phenylacetamido)methyl]-5,5-dimethyl-1,3-thiazolidin-4-yl]formamido}ethyl)-5,5-dimethyl-1,3-thiazolidine-4-carboxamide | HIV 1 |  | Humber et al | ZINC95610911 | |
| 388 | (2R,4S)-2-[(R)-[bis(2-hydroxyethyl)carbamoyl](1-phenylacetamido)methyl]-N-(2-{[(2R,4S)-2-[(R)-[bis(2-hydroxyethyl)carbamoyl](1-phenylacetamido)methyl]-5,5-dimethyl-1,3-thiazolidin-4-yl]formamido}ethyl)-5,5-dimethyl-1,3-thiazolidine-4-carboxamide | HIV 1 |  | Humber et al |  | |
| 389 | (2R,4S)-2-[(1R)-2-hydroxy-1-(1-phenylacetamido)ethyl]-N-(2-{[(2R,4S)-2-[(1R)-2-hydroxy-1-(1-phenylacetamido)ethyl]-5,5-dimethyl-1,3-thiazolidin-4-yl]formamido}ethyl)-5,5-dimethyl-1,3-thiazolidine-4-carboxamide | HIV 1 |  | Humber et al | ZINC26845773 | |
| 390 | (2R,4S)-N-(2-{[(2R,4S)-5,5-dimethyl-2-[(1-phenylacetamido)methyl]-1,3-thiazolidin-4-yl]formamido}ethyl)-5,5-dimethyl-2-[(1-phenylacetamido)methyl]-1,3-thiazolidine-4-carboxamide | HIV 1 |  | Humber et al | ZINC26841958 | |
| 391 | (2R,4S)-N-(2-{[(2R,4S)-2-[(R)-(ethylcarbamoyl)(1-phenylacetamido)methyl]-5,5-dimethyl-1,3-thiazolidin-4-yl]formamido}ethyl)-5,5-dimethyl-2-[(1-phenylacetamido)methyl]-1,3-thiazolidine-4-carboxamide | HIV 1 |  | Humber et al | ZINC26841952 | |
| 392 | (2R,4S)-2-[(R)-amino(ethylcarbamoyl)methyl]-N-(2-{[(2R,4S)-2-[(R)-amino(ethylcarbamoyl)methyl]-5,5-dimethyl-1,3-thiazolidin-4-yl]formamido}ethyl)-5,5-dimethyl-1,3-thiazolidine-4-carboxamide | HIV 1 |  | Humber et al | ZINC23357743 | |
| 393 | (2R,4S)-2-[(R)-(ethylcarbamoyl)(phenylformamido)methyl]-N-(2-{[(2R,4S)-2-[(R)-(ethylcarbamoyl)(phenylformamido)methyl]-5,5-dimethyl-1,3-thiazolidin-4-yl]formamido}ethyl)-5,5-dimethyl-1,3-thiazolidine-4-carboxamide | HIV 1 |  | Humber et al | ZINC95610553 | |
| 394 | (2R,4S)-2-[(R)-(ethylcarbamoyl)(3-phenylpropanamido)methyl]-N-(2-{[(2R,4S)-2-[(R)-(ethylcarbamoyl)(3-phenylpropanamido)methyl]-5,5-dimethyl-1,3-thiazolidin-4-yl]formamido}ethyl)-5,5-dimethyl-1,3-thiazolidine-4-carboxamide | HIV 1 |  | Humber et al | ZINC95546162 | |
| 395 | (2R,4S)-2-[(R)-(ethylcarbamoyl)[1-(naphthalen-2-yl)acetamido]methyl]-N-(2-{[(2R,4S)-2-[(R)-(ethylcarbamoyl)[1-(naphthalen-2-yl)acetamido]methyl]-5,5-dimethyl-1,3-thiazolidin-4-yl]formamido}ethyl)-5,5-dimethyl-1,3-thiazolidine-4-carboxamide | HIV 1 |  | Humber et al |  | |
| 396 | (2R,4S)-2-[(R)-(ethylcarbamoyl)[1-(pyridin-2-yl)acetamido]methyl]-N-(2-{[(2R,4S)-2-[(R)-(ethylcarbamoyl)[1-(pyridin-2-yl)acetamido]methyl]-5,5-dimethyl-1,3-thiazolidin-4-yl]formamido}ethyl)-5,5-dimethyl-1,3-thiazolidine-4-carboxamide | HIV 1 |  | Humber et al | ZINC95614715 | |
| 397 | (2R,4S)-2-[(R)-(ethylcarbamoyl)[1-(thiophen-2-yl)acetamido]methyl]-N-(2-{[(2R,4S)-2-[(R)-(ethylcarbamoyl)[1-(thiophen-2-yl)acetamido]methyl]-5,5-dimethyl-1,3-thiazolidin-4-yl]formamido}ethyl)-5,5-dimethyl-1,3-thiazolidine-4-carboxamide | HIV 1 |  | Humber et al | ZINC95546265 | |
| 398 | (2R,4S)-2-[(R)-(ethylcarbamoyl)(phenylmethane)sulfonamidomethyl]-N-(2-{[(2R,4S)-2-[(R)-(ethylcarbamoyl)(phenylmethane)sulfonamidomethyl]-5,5-dimethyl-1,3-thiazolidin-4-yl]formamido}ethyl)-5,5-dimethyl-1,3-thiazolidine-4-carboxamide | HIV 1 |  | Humber et al |  | |
| 399 | (2R,4S)-2-[(R)-(ethylcarbamoyl)[1-(4-hydroxyphenyl)acetamido]methyl]-N-(2-{[(2R,4S)-2-[(R)-(ethylcarbamoyl)[1-(4-hydroxyphenyl)acetamido]methyl]-5,5-dimethyl-1,3-thiazolidin-4-yl]formamido}ethyl)-5,5-dimethyl-1,3-thiazolidine-4-carboxamide | HIV 1 |  | Humber et al | ZINC95546260 | |
| 400 | (2R,4S)-2-[(R)-(ethylcarbamoyl)(3-methylbutanamido)methyl]-N-(2-{[(2R,4S)-2-[(R)-(ethylcarbamoyl)(3-methylbutanamido)methyl]-5,5-dimethyl-1,3-thiazolidin-4-yl]formamido}ethyl)-5,5-dimethyl-1,3-thiazolidine-4-carboxamide | HIV 1 |  | Humber et al | ZINC26838537 | |
| 401 | (2R,4S)-2-[(R)-(ethylcarbamoyl)[(2-phenylphenyl)formamido]methyl]-N-(2-{[(2R,4S)-2-[(R)-(ethylcarbamoyl)[(2-phenylphenyl)formamido]methyl]-5,5-dimethyl-1,3-thiazolidin-4-yl]formamido}ethyl)-5,5-dimethyl-1,3-thiazolidine-4-carboxamide | HIV 1 |  | Humber et al |  | |
| 402 | (2R,4S)-2-[(R)-[(2-benzylphenyl)formamido](ethylcarbamoyl)methyl]-N-(2-{[(2R,4S)-2-[(R)-[(2-benzylphenyl)formamido](ethylcarbamoyl)methyl]-5,5-dimethyl-1,3-thiazolidin-4-yl]formamido}ethyl)-5,5-dimethyl-1,3-thiazolidine-4-carboxamide | HIV 1 |  | Humber et al |  | |
| 403 | (2R,4S)-2-[(R)-(ethylcarbamoyl)[(2-phenoxyphenyl)formamido]methyl]-N-(2-{[(2R,4S)-2-[(R)-(ethylcarbamoyl)[(2-phenoxyphenyl)formamido]methyl]-5,5-dimethyl-1,3-thiazolidin-4-yl]formamido}ethyl)-5,5-dimethyl-1,3-thiazolidine-4-carboxamide | HIV 1 |  | Humber et al |  | |
| 404 | (2R,4S)-2-[(R)-(ethylcarbamoyl)({[2-(phenylamino)phenyl]formamido})methyl]-N-(2-{[(2R,4S)-2-[(R)-(ethylcarbamoyl)({[2-(phenylamino)phenyl]formamido})methyl]-5,5-dimethyl-1,3-thiazolidin-4-yl]formamido}ethyl)-5,5-dimethyl-1,3-thiazolidine-4-carboxamide | HIV 1 |  | Humber et al |  | |
| 405 | (2R,4S)-2-[(R)-(ethylcarbamoyl)[(2Z)-3-phenylprop-2-enamido]methyl]-N-(2-{[(2R,4S)-2-[(R)-(ethylcarbamoyl)[(2Z)-3-phenylprop-2-enamido]methyl]-5,5-dimethyl-1,3-thiazolidin-4-yl]formamido}ethyl)-5,5-dimethyl-1,3-thiazolidine-4-carboxamide | HIV 1 |  | Humber et al | ZINC95546165 | |
| 406 | (2R,4S)-2-[(R)-(benzylcarbamoyl)[1-(pyridin-2-yl)acetamido]methyl]-N-(2-{[(2R,4S)-2-[(R)-(benzylcarbamoyl)[1-(pyridin-2-yl)acetamido]methyl]-5,5-dimethyl-1,3-thiazolidin-4-yl]formamido}ethyl)-5,5-dimethyl-1,3-thiazolidine-4-carboxamide | HIV 1 |  | Humber et al |  | |
| 407 | (2R,4S)-2-[(R)-{[(4-chlorophenyl)methyl]carbamoyl}[1-(pyridin-2-yl)acetamido]methyl]-N-(2-{[(2R,4S)-2-[(R)-{[(4-chlorophenyl)methyl]carbamoyl}[1-(pyridin-2-yl)acetamido]methyl]-5,5-dimethyl-1,3-thiazolidin-4-yl]formamido}ethyl)-5,5-dimethyl-1,3-thiazolidine-4-carboxamide | HIV 1 |  | Humber et al |  | |
| 408 | (2R,4S)-2-[(R)-{[(2,4-dichlorophenyl)methyl]carbamoyl}[1-(pyridin-2-yl)acetamido]methyl]-N-(2-{[(2R,4S)-2-[(R)-{[(2,4-dichlorophenyl)methyl]carbamoyl}[1-(pyridin-2-yl)acetamido]methyl]-5,5-dimethyl-1,3-thiazolidin-4-yl]formamido}ethyl)-5,5-dimethyl-1,3-thiazolidine-4-carboxamide | HIV 1 |  | Humber et al |  | |
| 409 | (2R,4S)-2-[(R)-(benzylcarbamoyl)[(2-phenylphenyl)formamido]methyl]-N-(2-{[(2R,4S)-2-[(R)-(benzylcarbamoyl)[(2-phenylphenyl)formamido]methyl]-5,5-dimethyl-1,3-thiazolidin-4-yl]formamido}ethyl)-5,5-dimethyl-1,3-thiazolidine-4-carboxamide | HIV 1 |  | Humber et al |  | |
| 410 | (2R,4S)-2-[(R)-({[4-(dimethylamino)phenyl]methyl}carbamoyl)[(2-phenylphenyl)formamido]methyl]-N-(2-{[(2R,4S)-2-[(R)-({[4-(dimethylamino)phenyl]methyl}carbamoyl)[(2-phenylphenyl)formamido]methyl]-5,5-dimethyl-1,3-thiazolidin-4-yl]formamido}ethyl)-5,5-dimethyl-1,3-thiazolidine-4-carboxamide | HIV 1 |  | Humber et al |  | |
| 411 | (2S)-N-[(2S,3R)-4-[(3S,4aS,8aS)-3-(tert-butylcarbamoyl)-decahydroisoquinolin-2-yl]-3-hydroxy-1-phenylbutan-2-yl]-2-(quinolin-2-ylformamido)butanediamide | HIV 1 |  | Humber et al | ZINC03914596 | |
| 412 | (2R,4S)-2-[(R)-(benzylcarbamoyl)(1-phenylacetamido)methyl]-N-(2-{[(2R,4S)-2-[(R)-(benzylcarbamoyl)(1-phenylacetamido)methyl]-5,5-dimethyl-1,3-thiazolidin-4-yl]formamido}ethyl)-5,5-dimethyl-1,3-thiazolidine-4-carboxamide | HIV 1 |  | Holmes et al |  | |
| 413 | (2R,4S)-2-[(R)-(benzylcarbamoyl)(1-phenylacetamido)methyl]-N-(3-{[(2R,4S)-2-[(R)-(benzylcarbamoyl)(1-phenylacetamido)methyl]-5,5-dimethyl-1,3-thiazolidin-4-yl]formamido}propyl)-5,5-dimethyl-1,3-thiazolidine-4-carboxamide | HIV 1 |  | Holmes et al |  | |
| 414 | (2R,4S)-2-[(R)-(benzylcarbamoyl)(1-phenylacetamido)methyl]-N-(3-{[(2R,4S)-2-[(R)-(benzylcarbamoyl)(1-phenylacetamido)methyl]-5,5-dimethyl-1,3-thiazolidin-4-yl]formamido}-2-hydroxypropyl)-5,5-dimethyl-1,3-thiazolidine-4-carboxamide | HIV 1 |  | Holmes et al |  | |
| 415 | (2R,4S)-2-[(R)-(benzylcarbamoyl)(1-phenylacetamido)methyl]-5,5-dimethyl-1,3-thiazolidine-4-carboxamide::[2R-[2a(R*),4B]]-4-Carbamoyl-5,5-dimethyl-a-[(phenylacetyl)amino]-N-(phenylmethyl)-2-thiazolidineacetamide | HIV 1 |  | Holmes et al | ZINC13833384 | |
| 416 | (2R,4S)-2-[(R)-(benzylcarbamoyl)(1-phenylacetamido)methyl]-N-(2-hydroxyethyl)-5,5-dimethyl-1,3-thiazolidine-4-carboxamide | HIV 1 |  | Holmes et al | ZINC13833385 | |
| 417 | (2R,4S)-2-[(R)-(benzylcarbamoyl)(1-phenylacetamido)methyl]-N-[(2S)-1-hydroxy-3-phenylpropan-2-yl]-5,5-dimethyl-1,3-thiazolidine-4-carboxamide | HIV 1 |  | Holmes et al | ZINC14943089 | |
| 418 | (2R,4S)-2-[(R)-(benzylcarbamoyl)(1-phenylacetamido)methyl]-N-[(2R)-1-hydroxy-3-phenylpropan-2-yl]-5,5-dimethyl-1,3-thiazolidine-4-carboxamide | HIV 1 |  | Holmes et al | ZINC36139649 | |
| 419 | (2R,4S)-2-[(R)-(benzylcarbamoyl)(1-phenylacetamido)methyl]-N-[(2S)-1-hydroxy-4-phenylbutan-2-yl]-5,5-dimethyl-1,3-thiazolidine-4-carboxamide | HIV 1 |  | Holmes et al | ZINC14943090 | |
| 420 | (2R,4S)-2-[(R)-(benzylcarbamoyl)(1-phenylacetamido)methyl]-N-[(2R)-1-hydroxy-4-phenylbutan-2-yl]-5,5-dimethyl-1,3-thiazolidine-4-carboxamide | HIV 1 |  | Holmes et al | ZINC14943091 | |
| 421 | (2R,4S)-2-[(R)-(benzylcarbamoyl)(1-phenylacetamido)methyl]-N-[(2R)-1-hydroxy-4-methylpentan-2-yl]-5,5-dimethyl-1,3-thiazolidine-4-carboxamide | HIV 1 |  | Holmes et al | ZINC14943092 | |
| 422 | (2R,4S)-2-[(R)-(benzylcarbamoyl)(1-phenylacetamido)methyl]-N-[(2R)-1-hydroxy-3-methylbutan-2-yl]-5,5-dimethyl-1,3-thiazolidine-4-carboxamide | HIV 1 |  | Holmes et al | ZINC14943093 | |
| 423 | (2R,4S)-2-[(R)-(benzylcarbamoyl)(1-phenylacetamido)methyl]-N-(1-hydroxyhexan-2-yl)-5,5-dimethyl-1,3-thiazolidine-4-carboxamide | HIV 1 |  | Holmes et al | ZINC14943095 | |
| 424 | (2R,4S)-2-[(R)-(benzylcarbamoyl)(1-phenylacetamido)methyl]-N-(1-hydroxy-3-methylpentan-2-yl)-5,5-dimethyl-1,3-thiazolidine-4-carboxamide | HIV 1 |  | Holmes et al | ZINC14943099 | |
| 425 | (2R,4S)-2-[(R)-(benzylcarbamoyl)(1-phenylacetamido)methyl]-N-[(2R)-1-hydroxypropan-2-yl]-5,5-dimethyl-1,3-thiazolidine-4-carboxamide | HIV 1 |  | Holmes et al | ZINC13833386 | |
| 426 | (2R,4S)-2-[(R)-(benzylcarbamoyl)(1-phenylacetamido)methyl]-5,5-dimethyl-N-[(2S)-1-phenylpropan-2-yl]-1,3-thiazolidine-4-carboxamide | HIV 1 |  | Holmes et al | ZINC14943100 | |
| 427 | (2R,4S)-2-[(R)-(benzylcarbamoyl)(1-phenylacetamido)methyl]-5,5-dimethyl-N-[(2R)-1-phenylpropan-2-yl]-1,3-thiazolidine-4-carboxamide | HIV 1 |  | Holmes et al | ZINC14943101 | |
| 428 | (3S,4R)-4-{[(2R,4S)-2-[(R)-(benzylcarbamoyl)(1-phenylacetamido)methyl]-5,5-dimethyl-1,3-thiazolidin-4-yl]formamido}-3-hydroxy-5-phenylpentanamide | HIV 1 |  | Holmes et al | ZINC14943102 | |
| 429 | (3R,4R)-4-{[(2R,4S)-2-[(R)-(benzylcarbamoyl)(1-phenylacetamido)methyl]-5,5-dimethyl-1,3-thiazolidin-4-yl]formamido}-3-hydroxy-5-phenylpentanamide | HIV 1 |  | Holmes et al | ZINC14943103 | |
| 430 | (3S,4R)-4-{[(2R,4S)-2-[(R)-(benzylcarbamoyl)(1-phenylacetamido)methyl]-5,5-dimethyl-1,3-thiazolidin-4-yl]formamido}-3-hydroxy-N-(2-methylpropyl)-5-phenylpentanamide | HIV 1 |  | Holmes et al | ZINC26837898 | |
| 431 | (3R,4R)-4-{[(2R,4S)-2-[(R)-(benzylcarbamoyl)(1-phenylacetamido)methyl]-5,5-dimethyl-1,3-thiazolidin-4-yl]formamido}-3-hydroxy-N-(2-methylpropyl)-5-phenylpentanamide | HIV 1 |  | Holmes et al | ZINC26842420 | |
| 432 | (3S,4R)-N-benzyl-4-{[(2R,4S)-2-[(R)-(benzylcarbamoyl)(1-phenylacetamido)methyl]-5,5-dimethyl-1,3-thiazolidin-4-yl]formamido}-3-hydroxy-5-phenylpentanamide | HIV 1 |  | Holmes et al | ZINC26836034 | |
| 433 | (3R,4R)-N-benzyl-4-{[(2R,4S)-2-[(R)-(benzylcarbamoyl)(1-phenylacetamido)methyl]-5,5-dimethyl-1,3-thiazolidin-4-yl]formamido}-3-hydroxy-5-phenylpentanamide | HIV 1 |  | Holmes et al | ZINC26850813 | |
| 434 | (3S,4R)-N-(1H-1,3-benzodiazol-2-yl)-4-{[(2R,4S)-2-[(R)-(benzylcarbamoyl)(1-phenylacetamido)methyl]-5,5-dimethyl-1,3-thiazolidin-4-yl]formamido}-3-hydroxy-5-phenylpentanamide | HIV 1 |  | Holmes et al | ZINC95610826 | |
| 435 | (3R,4R)-N-(1H-1,3-benzodiazol-2-yl)-4-{[(2R,4S)-2-[(R)-(benzylcarbamoyl)(1-phenylacetamido)methyl]-5,5-dimethyl-1,3-thiazolidin-4-yl]formamido}-3-hydroxy-5-phenylpentanamide | HIV 1 |  | Holmes et al | ZINC95546167 | |
| 436 | (3R,4R)-4-{[(2R,4S)-2-[(R)-(benzylcarbamoyl)(1-phenylacetamido)methyl]-5,5-dimethyl-1,3-thiazolidin-4-yl]formamido}-3-hydroxy-N-[(1R)-2-hydroxy-1-phenylethyl]-5-phenylpentanamide | HIV 1 |  | Holmes et al | ZINC95546248 | |
| 437 | (3R,4R)-4-{[(2R,4S)-2-[(R)-(benzylcarbamoyl)(1-phenylacetamido)methyl]-5,5-dimethyl-1,3-thiazolidin-4-yl]formamido}-3-hydroxy-N-[(1S)-2-hydroxy-1-phenylethyl]-5-phenylpentanamide | HIV 1 |  | Holmes et al | ZINC95610565 | |
| 438 | (3R,4R)-4-{[(2R,4S)-2-[(R)-(benzylcarbamoyl)(1-phenylacetamido)methyl]-5,5-dimethyl-1,3-thiazolidin-4-yl]formamido}-3-hydroxy-N-[(2R)-1-hydroxy-3-phenylpropan-2-yl]-5-phenylpentanamide | HIV 1 |  | Holmes et al | ZINC95610693 | |
| 439 | (3R,4R)-4-{[(2R,4S)-2-[(R)-(benzylcarbamoyl)(1-phenylacetamido)methyl]-5,5-dimethyl-1,3-thiazolidin-4-yl]formamido}-3-hydroxy-N-[(2S)-1-hydroxy-3-phenylpropan-2-yl]-5-phenylpentanamide | HIV 1 |  | Holmes et al | ZINC95610504 | |
| 440 | (3R,4R)-4-{[(2R,4S)-2-[(R)-(benzylcarbamoyl)(1-phenylacetamido)methyl]-5,5-dimethyl-1,3-thiazolidin-4-yl]formamido}-3-hydroxy-N-[2-(1H-imidazol-2-yl)ethyl]-5-phenylpentanamide | HIV 1 |  | Holmes et al | ZINC26838529 | |
| 441 | 3-{4-[(2S,3R)-2-{[(tert-butoxy)carbonyl]amino}-4-{[(2R,3S)-3-{[(tert-butoxy)carbonyl]amino}-2-hydroxy-4-phenylbutyl]amino}-3-hydroxybutyl]phenoxy}propanoic acid | HIV 1 | 10.1021/jm950717a | Chen et al | ZINC14943113 | |
| 442 | (2R,3R,4R,5R)-3,4-dihydroxy-N,N'-bis[(1S,2R)-2-hydroxy-2,3-dihydro-1H-inden-1-yl]-2,5-bis(phenylsulfanyl)hexanediamide | HIV 1 |  | Muhlman et al | ZINC27079456 | |
| 443 | (2R,3R,4R,5R)-2,5-bis(benzylsulfanyl)-3,4-dihydroxy-N,N'-bis[(1S,2R)-2-hydroxy-2,3-dihydro-1H-inden-1-yl]hexanediamide | HIV 1 |  | Muhlman et al | ZINC27077936 | |
| 444 | (2R,3R,4R,5R)-2,5-bis[(2-fluorophenyl)sulfanyl]-3,4-dihydroxy-N,N'-bis[(1S,2R)-2-hydroxy-2,3-dihydro-1H-inden-1-yl]hexanediamide | HIV 1 |  | Muhlman et al | ZINC27079437 | |
| 445 | (2R,3R,4R,5R)-2,5-bis({[(2-fluorophenyl)methyl]sulfanyl})-3,4-dihydroxy-N,N'-bis[(1S,2R)-2-hydroxy-2,3-dihydro-1H-inden-1-yl]hexanediamide | HIV 1 |  | Muhlman et al | ZINC27082108 | |
| 446 | (2R,3R,4R,5R)-3,4-dihydroxy-N,N'-bis[(1S,2R)-2-hydroxy-2,3-dihydro-1H-inden-1-yl]-2,5-bis(pyridin-2-ylsulfanyl)hexanediamide | HIV 1 |  | Muhlman et al | ZINC27087020 | |
| 447 | (2R,3R,4R,5R)-3,4-dihydroxy-N,N'-bis[(1S,2R)-2-hydroxy-2,3-dihydro-1H-inden-1-yl]-2,5-bis(prop-2-en-1-ylsulfanyl)hexanediamide | HIV 1 |  | Muhlman et al | ZINC14943115 | |
| 448 | (2R,3R,4R,5R)-3,4-dihydroxy-N,N'-bis[(1S,2R)-2-hydroxy-2,3-dihydro-1H-inden-1-yl]-2,5-bis(thiophen-2-ylsulfanyl)hexanediamide | HIV 1 |  | Muhlman et al | ZINC27081094 | |
| 449 | (2R,3R,4R,5R)-3,4-dihydroxy-N,N'-bis[(1S)-2-methyl-1-(methylcarbamoyl)propyl]-2,5-bis(phenylsulfanyl)hexanediamide | HIV 1 |  | Muhlman et al | ZINC14943116 | |
| 450 | (2R,3R,4R,5R)-2,5-bis[(2-fluorophenyl)sulfanyl]-3,4-dihydroxy-N,N'-bis[(1S)-2-methyl-1-(methylcarbamoyl)propyl]hexanediamide | HIV 1 |  | Muhlman et al | ZINC27080606 | |
| 451 | (2R,3R,4R,5R)-3,4-dihydroxy-N,N'-bis[(1S)-2-methyl-1-(methylcarbamoyl)propyl]-2,5-bis(thiophen-2-ylsulfanyl)hexanediamide | HIV 1 |  | Muhlman et al | ZINC14943117 | |
| 452 | (4R)-N-tert-butyl-3-[(2S,3S)-2-hydroxy-3-(2-phenoxyacetamido)-4-phenylbutanoyl]-1,3-thiazolidine-4-carboxamide::(R)-N-tert-Butyl-3-[(2S,3S)-2-hydroxy-3-(phenoxyacetyl)amino-4-phenylbutanoyl]-1,3-thiazolidine-4-carboxamide | HIV 1 | 10.1021/jm980637h | Mimoto et al | ZINC13797485 | |
| 453 | (4R)-N-tert-butyl-3-[(2S,3S)-2-hydroxy-3-[2-(2-methylphenoxy)acetamido]-4-phenylbutanoyl]-1,3-thiazolidine-4-carboxamide::(R)-N-tert-Butyl-3-[(2S,3S)-2-hydroxy-3-(2-methylphenoxyacetyl)amino-4-phenylbutanoyl]-1,3-thiazolindine-4-carboxamide | HIV 1 | 10.1021/jm980637h | Mimoto et al | ZINC13797490 | |
| 454 | (4R)-N-tert-butyl-3-[(2S,3S)-2-hydroxy-3-[2-(3-methylphenoxy)acetamido]-4-phenylbutanoyl]-1,3-thiazolidine-4-carboxamide | HIV 1 | 10.1021/jm980637h | Mimoto et al | ZINC13797494 | |
| 455 | (4R)-N-tert-butyl-3-[(2S,3S)-2-hydroxy-3-[2-(4-methylphenoxy)acetamido]-4-phenylbutanoyl]-1,3-thiazolidine-4-carboxamide | HIV 1 | 10.1021/jm980637h | Mimoto et al | ZINC13797498 | |
| 456 | (4R)-N-tert-butyl-3-[(2S,3S)-3-[2-(2,6-dimethylphenoxy)acetamido]-2-hydroxy-4-phenylbutanoyl]-1,3-thiazolidine-4-carboxamide | HIV 1 | 10.1021/jm980637h | Mimoto et al | ZINC04392456 | |
| 457 | (4R)-N-tert-butyl-3-[(2S,3S)-3-[2-(2-ethyl-6-methylphenoxy)acetamido]-2-hydroxy-4-phenylbutanoyl]-1,3-thiazolidine-4-carboxamide | HIV 1 | 10.1021/jm980637h | Mimoto et al | ZINC14943118 | |
| 458 | (4R)-N-tert-butyl-3-[(2S,3S)-2-hydroxy-3-[2-(2-methyl-6-propylphenoxy)acetamido]-4-phenylbutanoyl]-1,3-thiazolidine-4-carboxamide | HIV 1 | 10.1021/jm980637h | Mimoto et al | ZINC14943119 | |
| 459 | (4R)-N-tert-butyl-3-[(2S,3S)-3-[2-(2,6-diethylphenoxy)acetamido]-2-hydroxy-4-phenylbutanoyl]-1,3-thiazolidine-4-carboxamide | HIV 1 | 10.1021/jm980637h | Mimoto et al | ZINC14943120 | |
| 460 | (4R)-N-tert-butyl-3-[(2S,3S)-3-[2-(2,6-dimethylphenoxy)acetamido]-2-hydroxy-4-phenylbutanoyl]-5,5-dimethyl-1,3-thiazolidine-4-carboxamide | HIV 1 | 10.1021/jm980637h | Mimoto et al | ZINC14943121 | |
| 461 | (4R)-N-tert-butyl-3-[(2S,3S)-2-hydroxy-3-[(3-hydroxy-2-methylphenyl)formamido]-4-phenylbutanoyl]-1,3-thiazolidine-4-carboxamide | HIV 1 | 10.1021/jm980637h | Mimoto et al | ZINC13797465 | |
| 462 | (4R)-N-tert-butyl-3-[(2S,3S)-2-hydroxy-3-[(3-hydroxy-2-methylphenyl)formamido]-4-phenylbutanoyl]-5,5-dimethyl-1,3-thiazolidine-4-carboxamide | HIV 1 | 10.1021/jm980637h | Mimoto et al | ZINC13797470 | |
| 463 | (4R)-N-tert-butyl-3-[(2S,3S)-3-[(2-ethyl-3-hydroxyphenyl)formamido]-2-hydroxy-4-phenylbutanoyl]-5,5-dimethyl-1,3-thiazolidine-4-carboxamide | HIV 1 | 10.1021/jm980637h | Mimoto et al | ZINC14943122 | |
| 464 | (4R)-3-[(2S,3S)-2-hydroxy-3-[(3-hydroxy-2-methylphenyl)formamido]-4-phenylbutanoyl]-N-[(2-methylphenyl)methyl]-1,3-thiazolidine-4-carboxamide | HIV 1 | 10.1021/jm980637h | Mimoto et al | ZINC14943123 | |
| 465 | (4R)-3-[(2S,3S)-2-hydroxy-3-[(3-hydroxy-2-methylphenyl)formamido]-4-phenylbutanoyl]-5,5-dimethyl-N-[(2-methylphenyl)methyl]-1,3-thiazolidine-4-carboxamide | HIV 1 | 10.1021/jm980637h | Mimoto et al | ZINC03941126 | |
| 466 | (4R)-N-[(2-chlorophenyl)methyl]-3-[(2S,3S)-2-hydroxy-3-[(3-hydroxy-2-methylphenyl)formamido]-4-phenylbutanoyl]-5,5-dimethyl-1,3-thiazolidine-4-carboxamide | HIV 1 | 10.1021/jm980637h | Mimoto et al | ZINC14943124 | |
| 467 | (4R)-3-[(2S,3S)-3-[(2-ethyl-3-hydroxyphenyl)formamido]-2-hydroxy-4-phenylbutanoyl]-5,5-dimethyl-N-[(2-methylphenyl)methyl]-1,3-thiazolidine-4-carboxamide | HIV 1 | 10.1021/jm980637h | Mimoto et al | ZINC03941127 | |
| 468 | (4R)-N-tert-butyl-3-[(2S,3S)-2-hydroxy-3-[(2R)-2-[2-(isoquinolin-5-yloxy)acetamido]-3-(methylsulfanyl)propanamido]-4-phenylbutanoyl]-5,5-dimethyl-1,3-thiazolidine-4-carboxamide | HIV 1 | 10.1021/jm980637h | Mimoto et al | ZINC03915520 | |
| 469 | (4R)-N-tert-butyl-3-[(2S,3S)-2-hydroxy-3-[(2R)-2-[2-(isoquinolin-5-yloxy)acetamido]-3-(methylsulfanyl)propanamido]-4-phenylbutanoyl]-1,3-thiazolidine-4-carboxamide | HIV 1 | 10.1021/jm980637h | Mimoto et al | ZINC03915521 | |
| 470 | (3S)-oxolan-3-yl N-[(2S,3S)-4-[(2S)-2-benzyl-4-[(2S)-4-[(1R)-1-carbamoyl-2-phenylethyl]-2-(2-methylpropyl)-3-oxo-2,3-dihydro-1H-pyrrol-2-yl]-3-oxo-2,3-dihydro-1H-pyrrol-2-yl]-3-hydroxy-1-phenylbutan-2-yl]carbamate | HIV 1 | 10.1021/jm0204587 | Smith et al | ZINC96126776 | |
| 471 | (3S)-oxolan-3-yl N-[(2S,3S,5R)-5-benzyl-3-hydroxy-5-{[(2R)-2-hydroxy-2,3-dihydro-1H-inden-1-yl]carbamoyl}-1-phenylpentan-2-yl]carbamate | HIV 1 | 10.1021/jm0204587 | Smith et al | ZINC14943126 | |
| 472 | (3S)-oxolan-3-yl N-[(2S,3S)-4-[(2S)-2-benzyl-4-[(2R)-2-hydroxy-2,3-dihydro-1H-inden-1-yl]-3-oxo-2,3-dihydro-1H-pyrrol-2-yl]-3-hydroxy-1-phenylbutan-2-yl]carbamate | HIV 1 | 10.1021/jm0204587 | Smith et al | ZINC14943130 | |
| 473 | (3S)-oxolan-3-yl N-[(2S,3S)-4-[(2S)-2-benzyl-4-[(2R)-2-(carbamoyloxy)-2,3-dihydro-1H-inden-1-yl]-3-oxo-2,3-dihydro-1H-pyrrol-2-yl]-3-hydroxy-1-phenylbutan-2-yl]carbamate | HIV 1 | 10.1021/jm0204587 | Smith et al | ZINC14943132 | |
| 474 | (3S)-oxolan-3-yl N-[(2S,3S)-4-[(2S)-2-benzyl-3-oxo-4-(1-oxo-1,2,3,4-tetrahydroisoquinolin-4-yl)-2,3-dihydro-1H-pyrrol-2-yl]-3-hydroxy-1-phenylbutan-2-yl]carbamate | HIV 1 | 10.1021/jm0204587 | Smith et al | ZINC14943134 | |
| 475 | (2S)-1-[(2S,4R)-4-benzyl-2-hydroxy-4-{[(1S,2R)-2-hydroxy-2,3-dihydro-1H-inden-1-yl]carbamoyl}butyl]-N-tert-butyl-4-(pyridin-3-ylmethyl)piperazine-2-carboxamide | HIV 1 | 10.1021/jm0204587 | Smith et al | ZINC22448696 | |
| 476 | (2S,4R)-N-tert-butyl-1-[(2R,3S)-2-hydroxy-3-[(2S)-3-methyl-2-(quinolin-2-ylformamido)butanamido]-4-phenylbutyl]-4-(pyridin-4-ylmethoxy)piperidine-2-carboxamide | HIV 1 |  | Beaulieu et al | ZINC03936474 | |
| 477 | (2S,4R)-N-tert-butyl-1-[(2R,3S)-2-hydroxy-3-(2-phenoxyacetamido)-4-phenylbutyl]-4-(pyridin-4-ylmethoxy)piperidine-2-carboxamide | HIV 1 |  | Beaulieu et al | ZINC14943136 | |
| 478 | (2S,4R)-N-tert-butyl-1-[(2R,3S)-2-hydroxy-4-phenyl-3-(3-phenylpropanamido)butyl]-4-(pyridin-4-ylmethoxy)piperidine-2-carboxamide | HIV 1 |  | Beaulieu et al | ZINC14943137 | |
| 479 | (2S,4R)-N-tert-butyl-1-[(2R,3S)-2-hydroxy-4-phenyl-3-[2-(phenylsulfanyl)acetamido]butyl]-4-(pyridin-4-ylmethoxy)piperidine-2-carboxamide | HIV 1 |  | Beaulieu et al | ZINC14943138 | |
| 480 | (2S,4R)-N-tert-butyl-1-[(2R,3S)-2-hydroxy-4-phenyl-3-(4-phenylbutanamido)butyl]-4-(pyridin-4-ylmethoxy)piperidine-2-carboxamide | HIV 1 |  | Beaulieu et al | ZINC14943139 | |
| 481 | (2S,4R)-N-tert-butyl-1-[(2R,3S)-2-hydroxy-4-phenyl-3-[(2E)-3-phenylprop-2-enamido]butyl]-4-(pyridin-4-ylmethoxy)piperidine-2-carboxamide | HIV 1 |  | Beaulieu et al | ZINC14943140 | |
| 482 | (2S,4R)-N-tert-butyl-1-[(2R,3S)-2-hydroxy-3-[3-(2-methylphenyl)propanamido]-4-phenylbutyl]-4-(pyridin-4-ylmethoxy)piperidine-2-carboxamide | HIV 1 |  | Beaulieu et al | ZINC14943142 | |
| 483 | (2S,4R)-N-tert-butyl-1-[(2R,3S)-2-hydroxy-3-[2-(2-methylphenoxy)acetamido]-4-phenylbutyl]-4-(pyridin-4-ylmethoxy)piperidine-2-carboxamide | HIV 1 |  | Beaulieu et al | ZINC14943143 | |
| 484 | (2S,4R)-N-tert-butyl-1-[(2R,3S)-3-[3-(2,6-dimethylphenyl)propanamido]-2-hydroxy-4-phenylbutyl]-4-(pyridin-4-ylmethoxy)piperidine-2-carboxamide | HIV 1 |  | Beaulieu et al | ZINC14943145 | |
| 485 | (2S,4R)-N-tert-butyl-1-[(2R,3S)-3-[2-(2,6-dimethylphenoxy)acetamido]-2-hydroxy-4-phenylbutyl]-4-(pyridin-4-ylmethoxy)piperidine-2-carboxamide | HIV 1 |  | Beaulieu et al | ZINC14943146 | |
| 486 | (2S,4R)-N-tert-butyl-1-[(2R,3S)-3-[2-(2,6-dimethylphenoxy)propanamido]-2-hydroxy-4-phenylbutyl]-4-(pyridin-4-ylmethoxy)piperidine-2-carboxamide | HIV 1 |  | Beaulieu et al | ZINC14943148 | |
| 487 | (2S,4R)-N-tert-butyl-1-[(2R,3S)-2-hydroxy-3-[(3-hydroxy-2-methylphenyl)formamido]-4-phenylbutyl]-4-(pyridin-4-ylmethoxy)piperidine-2-carboxamide | HIV 1 |  | Beaulieu et al | ZINC14943149 | |
| 488 | (3S)-oxolan-3-yl N-[(2S,3R)-4-[(2S,4R)-2-(tert-butylcarbamoyl)-4-(pyridin-4-ylmethoxy)piperidin-1-yl]-3-hydroxy-1-phenylbutan-2-yl]carbamate | HIV 1 |  | Beaulieu et al | ZINC14943150 | |
| 489 | (2S,4R)-N-tert-butyl-1-[(2R,3S)-3-[2-(2,6-dimethylphenoxy)acetamido]-2-hydroxy-4-phenylbutyl]-4-[(pyridin-4-ylmethyl)sulfanyl]piperidine-2-carboxamide | HIV 1 |  | Beaulieu et al | ZINC14943151 | |
| 490 | (2S,4R)-N-tert-butyl-1-[(2R,3S)-3-[2-(2,6-dimethylphenoxy)acetamido]-2-hydroxy-4-phenylbutyl]-4-[(pyridin-3-ylmethyl)sulfanyl]piperidine-2-carboxamide | HIV 1 |  | Beaulieu et al | ZINC14943152 | |
| 491 | (2S,4R)-N-tert-butyl-1-[(2R,3S)-3-[2-(2,6-dimethylphenoxy)acetamido]-2-hydroxy-4-phenylbutyl]-4-(pyridin-4-ylsulfanyl)piperidine-2-carboxamide | HIV 1 |  | Beaulieu et al | ZINC03918440 | |
| 492 | (2S,4R)-N-tert-butyl-1-[(2R,3S)-3-[2-(2,6-dimethylphenoxy)acetamido]-2-hydroxy-4-phenylbutyl]-4-[(2,6-dimethylpyrimidin-4-yl)sulfanyl]piperidine-2-carboxamide | HIV 1 |  | Beaulieu et al | ZINC49605877 | |
| 493 | (2S)-N-[(2S,3R)-4-[(3S,4aS,8aS)-3-(tert-butylcarbamoyl)-decahydroisoquinolin-2-yl]-3-hydroxy-1-phenylbutan-2-yl]-2-(quinolin-2-ylformamido)butanediamide | HIV 1 |  | Beaulieu et al | ZINC03914596 | |
| 494 | (3S)-oxolan-3-yl N-[(2S,3R)-4-[(4-aminobenzene)(2-methylpropyl)sulfonamido]-3-hydroxy-1-phenylbutan-2-yl]carbamate | HIV 1 |  | Beaulieu et al | ZINC03809192 | |
| 495 | (2R,4S,5S)-2-benzyl-N-[(1S)-1-{[(1S)-1-carbamoyl-2-phenylethyl]carbamoyl}-3-methylbutyl]-5-(3,3-dimethylbutanamido)-4-hydroxy-6-phenylhexanamide | HIV 1 |  | Vacca et al | ZINC03935320 | |
| 496 | (2S)-1-[(2S,4R)-4-benzyl-2-hydroxy-4-{[(1S,2R)-2-hydroxy-2,3-dihydro-1H-inden-1-yl]carbamoyl}butyl]-N-tert-butyl-4-(pyridin-3-ylmethyl)piperazine-2-carboxamide | HIV 1 |  | Vacca et al | ZINC22448696 | |
| 497 | 4-Cyano-N-[3-[cyclopropyl(5,6,7,8,9,10-hexahydro-4-hydroxy-2-oxo-2H-cycloocta[b]pyran-3-yl)methyl]phenyl]-benzenesulfonamide | HIV 1 |  | Skulnick et al | ZINC03873405 | |
| 498 | (2S)-1-[(2S,4R)-4-benzyl-2-hydroxy-4-{[(1S,2R)-2-hydroxy-2,3-dihydro-1H-inden-1-yl]carbamoyl}butyl]-N-tert-butyl-4-(pyridin-3-ylmethyl)piperazine-2-carboxamide | HIV 1 | 10.1021/bi035701y | Clemente et al | ZINC22448696 | |
| 499 | (3S,4aS,8aS)-N-tert-butyl-2-[(2R,3R)-2-hydroxy-3-[(3-hydroxy-2-methylphenyl)formamido]-4-(phenylsulfanyl)butyl]-decahydroisoquinoline-3-carboxamide | HIV 1 | 10.1021/bi035701y | Clemente et al | ZINC03833846 | |
| 500 | (4R)-3-[(2S,3S)-2-hydroxy-3-[(3-hydroxy-2-methylphenyl)formamido]-4-phenylbutanoyl]-5,5-dimethyl-N-[(2-methylphenyl)methyl]-1,3-thiazolidine-4-carboxamide | HIV 1 | 10.1021/bi035701y | Clemente et al | ZINC03941126 | |
| 501 | (4R,5S,6S,7R)-4,7-dibenzyl-5,6-dihydroxy-1,3-bis[(2E)-3-(1H-pyrazol-4-yl)prop-2-en-1-yl]-1,3-diazepan-2-one | HIV 1 | 10.1021/jm9602571 | Lam et al | ZINC03948988 | |
| 502 | (4R,5S,6S,7R)-4,7-dibenzyl-1-(cyclopropylmethyl)-5,6-dihydroxy-3-[(2E)-3-(1H-pyrazol-4-yl)prop-2-en-1-yl]-1,3-diazepan-2-one | HIV 1 | 10.1021/jm9602571 | Lam et al | ZINC03826079 | |
| 503 | (4R,5S,6S,7R)-4,7-dibenzyl-1-(cyclopropylmethyl)-5,6-dihydroxy-3-(6-hydroxyhexyl)-1,3-diazepan-2-one | HIV 1 | 10.1021/jm9602571 | Lam et al | ZINC14907367 | |
| 504 | (4R,5S,6S,7R)-4,7-dibenzyl-1-(cyclopropylmethyl)-5,6-dihydroxy-3-(5-hydroxypentyl)-1,3-diazepan-2-one | HIV 1 | 10.1021/jm9602571 | Lam et al | ZINC14907369 | |
| 505 | (4R,5S,6S,7R)-4,7-dibenzyl-1-butyl-3-(cyclopropylmethyl)-5,6-dihydroxy-1,3-diazepan-2-one | HIV 1 | 10.1021/jm9602571 | Lam et al | ZINC14907371 | |
| 506 | (4R,5S,6S,7R)-4,7-dibenzyl-5,6-dihydroxy-1,3-bis(5-hydroxypentyl)-1,3-diazepan-2-one | HIV 1 | 10.1021/jm9602571 | Lam et al | ZINC14907373 | |
| 507 | (4R,5S,6S,7R)-4,7-dibenzyl-5,6-dihydroxy-1,3-bis(3-methylbut-2-en-1-yl)-1,3-diazepan-2-one | HIV 1 | 10.1021/jm9602571 | Lam et al | ZINC13604434 | |
| 508 | (4R,5S,6S,7R)-4,7-dibenzyl-1-butyl-5,6-dihydroxy-3-(3-methylbut-2-en-1-yl)-1,3-diazepan-2-one | HIV 1 | 10.1021/jm9602571 | Lam et al | ZINC14907375 | |
| 509 | (4R,5S,6S,7R)-4,7-dibenzyl-5,6-dihydroxy-1,3-bis(6-hydroxyhexyl)-1,3-diazepan-2-one | HIV 1 | 10.1021/jm9602571 | Lam et al | ZINC14907377 | |
| 510 | (4R,5S,6S,7R)-4,7-dibenzyl-1-(cyclopropylmethyl)-5,6-dihydroxy-3-(3-methylbut-2-en-1-yl)-1,3-diazepan-2-one | HIV 1 | 10.1021/jm9602571 | Lam et al | ZINC14907379 | |
| 511 | (4R,5S,6S,7R)-4,7-dibenzyl-5,6-dihydroxy-3-(5-hydroxypentyl)-1-(5-methanesulfonylpentyl)-1,3-diazepan-2-one | HIV 1 | 10.1021/jm9602571 | Lam et al | ZINC14907381 | |
| 512 | (4R,5S,6S,7R)-4,7-dibenzyl-5,6-dihydroxy-1-(6-hydroxyhexyl)-3-(5-hydroxypentyl)-1,3-diazepan-2-one | HIV 1 | 10.1021/jm9602571 | Lam et al | ZINC14907383 | |
| 513 | (4R,5S,6S,7R)-4,7-dibenzyl-5,6-dihydroxy-3-(3-methylbut-2-en-1-yl)-1-(prop-2-en-1-yl)-1,3-diazepan-2-one | HIV 1 | 10.1021/jm9602571 | Lam et al | ZINC14907384 | |
| 514 | (4R,5S,6S,7R)-4,7-dibenzyl-5,6-dihydroxy-1,3-bis(5-hydroxyhexyl)-1,3-diazepan-2-one | HIV 1 | 10.1021/jm9602571 | Lam et al | ZINC14907392 | |
| 515 | (4R,5S,6S,7R)-4,7-dibenzyl-5,6-dihydroxy-3-(5-hydroxypentyl)-1-(5-methanesulfinylpentyl)-1,3-diazepan-2-one | HIV 1 | 10.1021/jm9602571 | Lam et al | ZINC14907397 | |
| 516 | (4R,5S,6S,7R)-4,7-dibenzyl-1-(cyclopropylmethyl)-5,6-dihydroxy-3-(prop-2-en-1-yl)-1,3-diazepan-2-one | HIV 1 | 10.1021/jm9602571 | Lam et al | ZINC14907398 | |
| 517 | (4R,5S,6S,7R)-4,7-dibenzyl-1-butyl-5,6-dihydroxy-3-(prop-2-en-1-yl)-1,3-diazepan-2-one | HIV 1 | 10.1021/jm9602571 | Lam et al | ZINC14907407 | |
| 518 | (4R,5S,6S,7R)-4,7-dibenzyl-5,6-dihydroxy-1,3-dipentyl-1,3-diazepan-2-one | HIV 1 | 10.1021/jm9602571 | Lam et al | ZINC13604418 | |
| 519 | (4R,5S,6S,7R)-4,7-dibenzyl-1-butyl-3-(cyclopentylmethyl)-5,6-dihydroxy-1,3-diazepan-2-one | HIV 1 | 10.1021/jm9602571 | Lam et al | ZINC14907413 | |
| 520 | (4R,5S,6S,7R)-4,7-dibenzyl-5,6-dihydroxy-1,3-bis(prop-2-en-1-yl)-1,3-diazepan-2-one | HIV 1 | 10.1021/jm9602571 | Lam et al | ZINC03786324 | |
| 521 | (4R,5S,6S,7R)-4,7-dibenzyl-1-(cyclopropylmethyl)-5,6-dihydroxy-3-[(2E)-3-phenylprop-2-en-1-yl]-1,3-diazepan-2-one | HIV 1 | 10.1021/jm9602571 | Lam et al | ZINC14907414 | |
| 522 | (4R,5S,6S,7R)-4,7-dibenzyl-5,6-dihydroxy-1,3-bis[(2E)-3-phenylprop-2-en-1-yl]-1,3-diazepan-2-one | HIV 1 | 10.1021/jm9602571 | Lam et al | ZINC14907416 | |
| 523 | 4-[(1E)-3-[(4R,5S,6S,7R)-4,7-dibenzyl-5,6-dihydroxy-3-[(2E)-3-[1-(methylcarbamoyl)-1H-pyrazol-4-yl]prop-2-en-1-yl]-2-oxo-1,3-diazepan-1-yl]prop-1-en-1-yl]-N-methyl-1H-pyrazole-1-carboxamide | HIV 1 | 10.1021/jm9602571 | Lam et al | ZINC96126612 | |
| 524 | (4R,5S,6S,7R)-4,7-dibenzyl-5,6-dihydroxy-1,3-bis(4-methylpentyl)-1,3-diazepan-2-one | HIV 1 | 10.1021/jm9602571 | Lam et al | ZINC13604429 | |
| 525 | (4R,5S,6S,7R)-4,7-dibenzyl-5,6-dihydroxy-3-(5-hydroxypentyl)-1-(5-methoxypentyl)-1,3-diazepan-2-one | HIV 1 | 10.1021/jm9602571 | Lam et al | ZINC14907420 | |
| 526 | (4R,5S,6S,7R)-4,7-dibenzyl-5,6-dihydroxy-1,3-dipropyl-1,3-diazepan-2-one | HIV 1 | 10.1021/jm9602571 | Lam et al | ZINC06380343 | |
| 527 | (4R,5S,6S,7R)-4,7-dibenzyl-5,6-dihydroxy-1,3-bis(3-methylbutyl)-1,3-diazepan-2-one | HIV 1 | 10.1021/jm9602571 | Lam et al | ZINC13604427 | |
| 528 | (4R,5S,6S,7R)-4,7-dibenzyl-5,6-dihydroxy-1,3-bis[4-(oxiran-2-yl)butyl]-1,3-diazepan-2-one | HIV 1 | 10.1021/jm9602571 | Lam et al | ZINC14907429 | |
| 529 | (4R,5S,6S,7R)-4,7-dibenzyl-5,6-dihydroxy-1,3-bis[(5Z)-5-(hydroxyimino)hexyl]-1,3-diazepan-2-one | HIV 1 | 10.1021/jm9602571 | Lam et al | ZINC14907431 | |
| 530 | (4R,5S,6S,7R)-4,7-dibenzyl-5,6-dihydroxy-1,3-bis[(2E)-3-(2-hydroxyphenyl)prop-2-en-1-yl]-1,3-diazepan-2-one | HIV 1 | 10.1021/jm9602571 | Lam et al | ZINC14907434 | |
| 531 | (4R,5S,6S,7R)-4,7-dibenzyl-5,6-dihydroxy-1-(5-hydroxypentyl)-3-[5-(methylsulfanyl)pentyl]-1,3-diazepan-2-one | HIV 1 | 10.1021/jm9602571 | Lam et al | ZINC14907437 | |
| 532 | (4R,5S,6S,7R)-4,7-dibenzyl-5,6-dihydroxy-1,3-bis(5-methylhexyl)-1,3-diazepan-2-one | HIV 1 | 10.1021/jm9602571 | Lam et al | ZINC14907441 | |
| 533 | (4R,5S,6S,7R)-4,7-dibenzyl-5,6-dihydroxy-1,3-bis({[2-(hydroxymethyl)cyclopropyl]methyl})-1,3-diazepan-2-one | HIV 1 | 10.1021/jm9602571 | Lam et al | ZINC14907451 | |
| 534 | (4R,5S,6S,7R)-4,7-dibenzyl-5,6-dihydroxy-1,3-bis(7-hydroxyheptyl)-1,3-diazepan-2-one | HIV 1 | 10.1021/jm9602571 | Lam et al | ZINC14907453 | |
| 535 | (4R,5S,6S,7R)-4,7-dibenzyl-5,6-dihydroxy-1,3-bis(3-phenylpropyl)-1,3-diazepan-2-one | HIV 1 | 10.1021/jm9602571 | Lam et al | ZINC14907458 | |
| 536 | (4R,5S,6S,7R)-4,7-dibenzyl-5,6-dihydroxy-1,3-bis[(2E)-3-{1-[(2-methoxyethoxy)methyl]-1H-pyrazol-4-yl}prop-2-en-1-yl]-1,3-diazepan-2-one | HIV 1 | 10.1021/jm9602571 | Lam et al | ZINC96126628 | |
| 537 | (4R,5S,6S,7R)-4,7-dibenzyl-5,6-dihydroxy-1,3-bis(4-hydroxybutyl)-1,3-diazepan-2-one | HIV 1 | 10.1021/jm9602571 | Lam et al | ZINC14907460 | |
| 538 | (4R,5S,6S,7R)-4,7-dibenzyl-5,6-dihydroxy-1,3-bis(5-hydroxy-5-methylhexyl)-1,3-diazepan-2-one | HIV 1 | 10.1021/jm9602571 | Lam et al | ZINC14907463 | |
| 539 | (4R,5S,6S,7R)-4,7-dibenzyl-5,6-dihydroxy-1,3-bis(2-methylpropyl)-1,3-diazepan-2-one | HIV 1 | 10.1021/jm9602571 | Lam et al | ZINC13604426 | |
| 540 | (4R,5S,6S,7R)-4,7-dibenzyl-5,6-dihydroxy-1,3-bis(3-hydroxypropyl)-1,3-diazepan-2-one | HIV 1 | 10.1021/jm9602571 | Lam et al | ZINC14907466 | |
| 541 | (4R,5S,6S,7R)-4,7-dibenzyl-5,6-dihydroxy-1,3-bis(5-oxohexyl)-1,3-diazepan-2-one | HIV 1 | 10.1021/jm9602571 | Lam et al | ZINC14907468 | |
| 542 | (4R,5S,6S,7R)-4,7-dibenzyl-1-butyl-5,6-dihydroxy-3-(2-phenylethyl)-1,3-diazepan-2-one | HIV 1 | 10.1021/jm9602571 | Lam et al | ZINC14907471 | |
| 543 | (4R,5S,6S,7R)-4,7-dibenzyl-5,6-dihydroxy-1,3-bis(5-methoxypentyl)-1,3-diazepan-2-one | HIV 1 | 10.1021/jm9602571 | Lam et al | ZINC14907472 | |
| 544 | (4R,5S,6S,7R)-4,7-dibenzyl-5,6-dihydroxy-1,3-bis(6-methylheptyl)-1,3-diazepan-2-one | HIV 1 | 10.1021/jm9602571 | Lam et al | ZINC14907474 | |
| 545 | (4R,5S,6S,7R)-4,7-dibenzyl-5,6-dihydroxy-1,3-bis[(2E)-4-{[5-(methylsulfanyl)-1H-1,2,4-triazol-3-yl]amino}but-2-en-1-yl]-1,3-diazepan-2-one | HIV 1 | 10.1021/jm9602571 | Lam et al | ZINC96126641 | |
| 546 | (4R,5S,6S,7R)-4,7-dibenzyl-5,6-dihydroxy-3-{[(1R,2R)-2-phenylcyclopropyl]methyl}-1-{[(1S,2S)-2-phenylcyclopropyl]methyl}-1,3-diazepan-2-one | HIV 1 | 10.1021/jm9602571 | Lam et al | ZINC96126645 | |
| 547 | (4R,5S,6S,7R)-4,7-dibenzyl-5,6-dihydroxy-1,3-bis(8-hydroxyoctyl)-1,3-diazepan-2-one | HIV 1 | 10.1021/jm9602571 | Lam et al | ZINC14908007 | |
| 548 | (4R,5S,6S,7R)-4,7-dibenzyl-5,6-dihydroxy-1,3-bis(2-methoxyethyl)-1,3-diazepan-2-one | HIV 1 | 10.1021/jm9602571 | Lam et al | ZINC13604422 | |
| 549 | (4R,5S,6S,7R)-4,7-dibenzyl-1-[(4R)-4-(4-fluorophenyl)-4-hydroxybutyl]-3-[(4S)-4-(4-fluorophenyl)-4-hydroxybutyl]-5,6-dihydroxy-1,3-diazepan-2-one | HIV 1 | 10.1021/jm9602571 | Lam et al | ZINC96126649 | |
| 550 | (4S,5R,6R,7S)-4,7-dibenzyl-1,3-bis(2-ethoxyethyl)-5,6-dihydroxy-1,3-diazepan-2-one | HIV 1 | 10.1021/jm9602571 | Lam et al | ZINC14908011 | |
| 551 | (4R,5S,6S,7R)-4,7-dibenzyl-5,6-dihydroxy-1,3-bis(2-hydroxy-3-phenylpropyl)-1,3-diazepan-2-one | HIV 1 | 10.1021/jm9602571 | Lam et al | ZINC14908018 | |
| 552 | (4R,5S,6S,7R)-4,7-dibenzyl-5,6-dihydroxy-1,3-bis[5-(1H-pyrazol-1-yl)pentyl]-1,3-diazepan-2-one | HIV 1 | 10.1021/jm9602571 | Lam et al | ZINC14908025 | |
| 553 | (4R,5S,6S,7R)-4,7-dibenzyl-5,6-dihydroxy-1,3-bis(3-phenylprop-2-yn-1-yl)-1,3-diazepan-2-one | HIV 1 | 10.1021/jm9602571 | Lam et al | ZINC14908027 | |
| 554 | (4R,5S,6S,7R)-4,7-dibenzyl-5,6-dihydroxy-1,3-bis[2-(2-hydroxyethoxy)ethyl]-1,3-diazepan-2-one | HIV 1 | 10.1021/jm9602571 | Lam et al | ZINC14908031 | |
| 555 | (4R,5S,6S,7R)-4,7-dibenzyl-5,6-dihydroxy-1,3-bis({2-[3-(2-hydroxypropyl)-2,2-dimethylcyclopropyl]ethyl})-1,3-diazepan-2-one | HIV 1 | 10.1021/jm9602571 | Lam et al | ZINC70454613 | |
| 556 | (4R,5S,6S,7R)-4,7-dibenzyl-5,6-dihydroxy-1,3-bis[(3-methyl-4,5-dihydro-1,2-oxazol-5-yl)methyl]-1,3-diazepan-2-one | HIV 1 | 10.1021/jm9602571 | Lam et al | ZINC14908048 | |
| 557 | (4R,5S,6S,7R)-4,7-dibenzyl-5,6-dihydroxy-1,3-bis[2-(morpholin-4-yl)ethyl]-1,3-diazepan-2-one | HIV 1 | 10.1021/jm9602571 | Lam et al | ZINC14908050 | |
| 558 | (4R,5S,6S,7R)-4,7-dibenzyl-5,6-dihydroxy-1,3-bis(2-hydroxybutyl)-1,3-diazepan-2-one | HIV 1 | 10.1021/jm9602571 | Lam et al | ZINC14908058 | |
| 559 | (4R,5S,6S,7R)-4,7-dibenzyl-5,6-dihydroxy-1,3-bis[2-(piperidin-4-yl)ethyl]-1,3-diazepan-2-one | HIV 1 | 10.1021/jm9602571 | Lam et al | ZINC14908059 | |
| 560 | (4R,5S,6S,7R)-4,7-dibenzyl-5,6-dihydroxy-1,3-bis[5-(1H-imidazol-1-yl)pentyl]-1,3-diazepan-2-one dihydrochloride | HIV 1 | 10.1021/jm9602571 | Lam et al | ZINC14908063 | |
| 561 | (4R,5S,6S,7R)-4,7-dibenzyl-5,6-dihydroxy-1,3-bis[5-(morpholin-4-yl)pentyl]-1,3-diazepan-2-one dihydrochloride | HIV 1 | 10.1021/jm9602571 | Lam et al | ZINC14908065 | |
| 562 | (4R,5S,6S,7R)-4,7-dibenzyl-5,6-dihydroxy-1,3-bis(2-hydroxy-3,3-dimethylbutyl)-1,3-diazepan-2-one | HIV 1 | 10.1021/jm9602571 | Lam et al | ZINC14908074 | |
| 563 | (4R,5S,6S,7R)-4,7-dibenzyl-5,6-dihydroxy-1,3-bis(2-hydroxyethyl)-1,3-diazepan-2-one | HIV 1 | 10.1021/jm9602571 | Lam et al | ZINC14908076 | |
| 564 | (4R,5S,6S,7R)-4,7-dibenzyl-5,6-dihydroxy-1,3-bis({[4-(hydroxymethyl)phenyl]methyl})-1,3-diazepan-2-one | HIV 1 | 10.1021/jm9602571 | Lam et al | ZINC03833858 | |
| 565 | (4R,5S,6S,7R)-4,7-dibenzyl-5,6-dihydroxy-1,3-bis(prop-2-en-1-yl)-1,3-diazepan-2-one | HIV 1 | 10.1021/jm9602571 | Lam et al | ZINC03786324 | |
| 566 | (4R,5S,6S,7R)-4,7-dibenzyl-5,6-dihydroxy-1,3-bis(naphthalen-2-ylmethyl)-1,3-diazepan-2-one | HIV 1 | 10.1021/jm9602571 | Lam et al | ZINC03833856 | |
| 567 | 3-{[(4R,5S,6S,7R)-4,7-dibenzyl-5,6-dihydroxy-2-oxo-3-{[3-(1,3-thiazol-2-ylcarbamoyl)phenyl]methyl}-1,3-diazepan-1-yl]methyl}-N-(1,3-thiazol-2-yl)benzamide | HIV 1 | 10.1021/jm9602571 | Lam et al | ZINC85548877 | |
| 568 | 3-{[(4R,5S,6S,7R)-4,7-dibenzyl-3-[(3-carbamoylphenyl)methyl]-5,6-dihydroxy-2-oxo-1,3-diazepan-1-yl]methyl}benzamide | HIV 1 | 10.1021/jm9602571 | Lam et al | ZINC14908078 | |
| 569 | 3-{[(4R,5S,6S,7R)-4,7-dibenzyl-5,6-dihydroxy-3-{[3-(methylcarbamoyl)phenyl]methyl}-2-oxo-1,3-diazepan-1-yl]methyl}-N-methylbenzamide | HIV 1 | 10.1021/jm9602571 | Lam et al | ZINC14908079 | |
| 570 | 3-{[(4R,5S,6S,7R)-4,7-dibenzyl-3-{[3-(ethylcarbamoyl)phenyl]methyl}-5,6-dihydroxy-2-oxo-1,3-diazepan-1-yl]methyl}-N-ethylbenzamide | HIV 1 | 10.1021/jm9602571 | Lam et al | ZINC26493670 | |
| 571 | 3-{[(4R,5S,6S,7R)-4,7-dibenzyl-5,6-dihydroxy-2-oxo-3-{[3-(pyridin-2-ylcarbamoyl)phenyl]methyl}-1,3-diazepan-1-yl]methyl}-N-(pyridin-2-yl)benzamide | HIV 1 | 10.1021/jm9602571 | Lam et al | ZINC95543992 | |
| 572 | 3-{[(4R,5S,6S,7R)-4,7-dibenzyl-5,6-dihydroxy-3-{[3-(1H-imidazol-2-ylcarbamoyl)phenyl]methyl}-2-oxo-1,3-diazepan-1-yl]methyl}-N-(1H-imidazol-2-yl)benzamide | HIV 1 | 10.1021/jm9602571 | Lam et al | ZINC29556734 | |
| 573 | (4R,5S,6S,7R)-4,7-dibenzyl-5,6-dihydroxy-1,3-bis(1H-indazol-5-ylmethyl)-1,3-diazepan-2-one | HIV 1 | 10.1021/jm9602571 | Lam et al | ZINC03935743 | |
| 574 | 3-{[(4R,5S,6S,7R)-4,7-dibenzyl-5,6-dihydroxy-3-{[3-(N'-hydroxycarbamimidoyl)phenyl]methyl}-2-oxo-1,3-diazepan-1-yl]methyl}-N'-hydroxybenzene-1-carboximidamide | HIV 1 | 10.1021/jm9602571 | Lam et al | ZINC14908086 | |
| 575 | (4R,5S,6S,7R)-4,7-dibenzyl-5,6-dihydroxy-1-(1H-indazol-5-ylmethyl)-1,3-diazepan-2-one | HIV 1 | 10.1021/jm9602571 | Lam et al | ZINC13780010 | |
| 576 | 3-{[(4R,5S,6S,7R)-4,7-dibenzyl-5,6-dihydroxy-3-(1H-indazol-5-ylmethyl)-2-oxo-1,3-diazepan-1-yl]methyl}benzoic acid | HIV 1 | 10.1021/jm9602571 | Lam et al | ZINC14908089 | |
| 577 | (4R,5S,6S,7R)-4,7-dibenzyl-5,6-dihydroxy-3-[(3-hydroxyphenyl)methyl]-1-(1H-indazol-5-ylmethyl)-1,3-diazepan-2-one | HIV 1 | 10.1021/jm9602571 | Lam et al | ZINC14908090 | |
| 578 | (4R,5S,6S,7R)-4,7-dibenzyl-5,6-dihydroxy-3-{[3-(2-hydroxyethyl)phenyl]methyl}-1-(1H-indazol-5-ylmethyl)-1,3-diazepan-2-one | HIV 1 | 10.1021/jm9602571 | Lam et al | ZINC14908092 | |
| 579 | 3-{[(4R,5S,6S,7R)-4,7-dibenzyl-3-[(3-formylphenyl)methyl]-5,6-dihydroxy-2-oxo-1,3-diazepan-1-yl]methyl}benzaldehyde | HIV 1 | 10.1021/jm9602571 | Lam et al | ZINC14908096 | |
| 580 | (4R,5S,6S,7R)-4,7-dibenzyl-5,6-dihydroxy-1,3-bis[(3-propanoylphenyl)methyl]-1,3-diazepan-2-one | HIV 1 | 10.1021/jm9602571 | Lam et al | ZINC14908100 | |
| 581 | (4R,5S,6S,7R)-4,7-dibenzyl-5,6-dihydroxy-1,3-bis({[3-(2,2,2-trifluoroacetyl)phenyl]methyl})-1,3-diazepan-2-one | HIV 1 | 10.1021/jm9602571 | Lam et al | ZINC26161293 | |
| 582 | (4R,5S,6S,7R)-4,7-dibenzyl-5,6-dihydroxy-1,3-bis({3-[(hydroxyimino)methyl]phenyl}methyl)-1,3-diazepan-2-one | HIV 1 | 10.1021/jm9602571 | Lam et al | ZINC14908107 | |
| 583 | (4R,5S,6S,7R)-4,7-dibenzyl-5,6-dihydroxy-1,3-bis({3-[1-(hydroxyimino)ethyl]phenyl}methyl)-1,3-diazepan-2-one::4R,5S,6S,7R)-Hexahydro-5,6-dihydroxy-1,3-bis[[3-[1-(hydroxyimino)ethyl]phenyl]methyl]-4,7-bis(phenylmethyl)-2H-1,3-diazepin-2-one | HIV 1 | 10.1021/jm9602571 | Lam et al | ZINC14908114 | |
| 584 | (4R,5S,6S,7R)-4,7-dibenzyl-5,6-dihydroxy-1,3-bis({3-[1-(hydroxyimino)propyl]phenyl}methyl)-1,3-diazepan-2-one | HIV 1 | 10.1021/jm9602571 | Lam et al | ZINC26167193 | |
| 585 | (4R,5S,6S,7R)-4,7-dibenzyl-5,6-dihydroxy-1,3-bis({3-[1-(hydroxyimino)butyl]phenyl}methyl)-1,3-diazepan-2-one | HIV 1 | 10.1021/jm9602571 | Lam et al | ZINC26162491 | |
| 586 | (4R,5S,6S,7R)-4,7-dibenzyl-5,6-dihydroxy-1,3-bis({3-[2,2,2-trifluoro-1-(hydroxyimino)ethyl]phenyl}methyl)-1,3-diazepan-2-one | HIV 1 | 10.1021/jm9602571 | Lam et al | ZINC26166723 | |
| 587 | (4R,5S,6S,7R)-4,7-dibenzyl-5,6-dihydroxy-1,3-bis({[3-(1H-pyrazol-3-yl)phenyl]methyl})-1,3-diazepan-2-one::(4R,5S,6S,7R)-Hexahydro-5,6-dihydroxy-1,3-bis[[3-(1H-pyrazol-3-yl)phenyl]methyl]-4,7-bis(phenylmethyl)-2H-1,3-diazepin-2-one | HIV 1 | 10.1021/jm9602571 | Lam et al | ZINC03937353 | |
| 588 | (4R,5S,6S,7R)-4,7-dibenzyl-5,6-dihydroxy-1,3-bis({[3-(1H-pyrazol-4-yl)phenyl]methyl})-1,3-diazepan-2-one::(4R,5S,6S,7R)-Hexahydro-5,6-dihydroxy-1,3-bis[[3-(1H-prazol-4-yl)phenyl]methyl]-4,7-bis(phenylmethyl)-2H-1,3-diazepin-2-one | HIV 1 | 10.1021/jm9602571 | Lam et al | ZINC26168566 | |
| 589 | (4R,5S,6S,7R)-4,7-dibenzyl-5,6-dihydroxy-1,3-bis({[3-(1H-imidazol-2-yl)phenyl]methyl})-1,3-diazepan-2-one::(4R,5S,6S,7R)-Hexahydro-5,6-dihydroxy-1,3-bis[[3-(1H-imidazol-2-yl)phenyl]methyl]-4,7-bis(phenylmethyl)-2H-1,3-diazepin-2-one | HIV 1 | 10.1021/jm9602571 | Lam et al | ZINC26165759 | |
| 590 | (4R,5S,6S,7R)-4,7-dibenzyl-5,6-dihydroxy-1,3-bis({[3-(1H-imidazol-4-yl)phenyl]methyl})-1,3-diazepan-2-one | HIV 1 | 10.1021/jm9602571 | Lam et al | ZINC26162995 | |
| 591 | (4R,5S,6S,7R)-4,7-dibenzyl-5,6-dihydroxy-1,3-bis({[3-(2H-1,2,3-triazol-4-yl)phenyl]methyl})-1,3-diazepan-2-one | HIV 1 | 10.1021/jm9602571 | Lam et al | ZINC26165871 | |
| 592 | (4R,5S,6S,7R)-4,7-dibenzyl-5,6-dihydroxy-1,3-bis({[3-(1H-1,2,4-triazol-3-yl)phenyl]methyl})-1,3-diazepan-2-one | HIV 1 | 10.1021/jm9602571 | Lam et al | ZINC26167582 | |
| 593 | (4R,5S,6S,7R)-4,7-dibenzyl-5,6-dihydroxy-1,3-bis({[3-(2H-1,2,3,4-tetrazol-5-yl)phenyl]methyl})-1,3-diazepan-2-one | HIV 1 | 10.1021/jm9602571 | Lam et al | ZINC26174612 | |
| 594 | (4R,5S,6S,7R)-4,7-dibenzyl-5,6-dihydroxy-1,3-dimethyl-1,3-diazepan-2-one | HIV 1 | 10.1021/jm9602571 | Lam et al | ZINC06481191 | |
| 595 | (4R,5S,6S,7R)-4,7-dibenzyl-5,6-dihydroxy-1,3-bis[2-(2-methoxyethoxy)ethyl]-1,3-diazepan-2-one | HIV 1 | 10.1021/jm9602571 | Lam et al | ZINC14946430 | |
| 596 | (4R,5S,6S,7R)-4,7-dibenzyl-5,6-dihydroxy-1,3-bis(2-methylprop-2-en-1-yl)-1,3-diazepan-2-one | HIV 1 | 10.1021/jm9602571 | Lam et al | ZINC13604432 | |
| 597 | (4R,5S,6S,7R)-4,7-dibenzyl-5,6-dihydroxy-1,3-bis(prop-2-yn-1-yl)-1,3-diazepan-2-one | HIV 1 | 10.1021/jm9602571 | Lam et al | ZINC05827983 | |
| 598 | (4R,5S,6S,7R)-4,7-dibenzyl-5,6-dihydroxy-1,3-bis(pyridin-2-ylmethyl)-1,3-diazepan-2-one | HIV 1 | 10.1021/jm9602571 | Lam et al | ZINC14946431 | |
| 599 | (4R,5S,6S,7R)-4,7-dibenzyl-5,6-dihydroxy-1,3-bis(pyridin-3-ylmethyl)-1,3-diazepan-2-one | HIV 1 | 10.1021/jm9602571 | Lam et al | ZINC14946432 | |
| 600 | (4R,5S,6S,7R)-4,7-dibenzyl-5,6-dihydroxy-1,3-bis(pyridin-4-ylmethyl)-1,3-diazepan-2-one | HIV 1 | 10.1021/jm9602571 | Lam et al | ZINC14946433 | |
| 601 | (4R,5S,6S,7R)-4,7-dibenzyl-5,6-dihydroxy-1-(naphthalen-2-ylmethyl)-3-(pyridin-4-ylmethyl)-1,3-diazepan-2-one | HIV 1 | 10.1021/jm9602571 | Lam et al | ZINC14946434 | |
| 602 | (4R,5S,6S,7R)-4,7-dibenzyl-1-[(4-fluorophenyl)methyl]-5,6-dihydroxy-3-(naphthalen-2-ylmethyl)-1,3-diazepan-2-one | HIV 1 | 10.1021/jm9602571 | Lam et al | ZINC14946435 | |
| 603 | (4R,5S,6S,7R)-4,7-dibenzyl-5,6-dihydroxy-3-{[4-(hydroxymethyl)phenyl]methyl}-1-(naphthalen-2-ylmethyl)-1,3-diazepan-2-one | HIV 1 | 10.1021/jm9602571 | Lam et al | ZINC14946436 | |
| 604 | (4R,5S,6S,7R)-4,7-dibenzyl-5,6-dihydroxy-3-[(3-hydroxyphenyl)methyl]-1-(naphthalen-2-ylmethyl)-1,3-diazepan-2-one | HIV 1 | 10.1021/jm9602571 | Lam et al | ZINC14946438 | |
| 605 | (4R,5S,6S,7R)-4,7-dibenzyl-5,6-dihydroxy-1,3-bis(naphthalen-1-ylmethyl)-1,3-diazepan-2-one | HIV 1 | 10.1021/jm9602571 | Lam et al | ZINC14946439 | |
| 606 | (4R,5S,6S,7R)-4,7-dibenzyl-5,6-dihydroxy-1,3-bis[(3-methylphenyl)methyl]-1,3-diazepan-2-one | HIV 1 | 10.1021/jm9602571 | Lam et al | ZINC14946446 | |
| 607 | (4R,5S,6S,7R)-4,7-dibenzyl-5,6-dihydroxy-1,3-bis[(4-methylphenyl)methyl]-1,3-diazepan-2-one | HIV 1 | 10.1021/jm9602571 | Lam et al | ZINC14946447 | |
| 608 | (4R,5S,6S,7R)-4,7-dibenzyl-5,6-dihydroxy-1,3-bis({[3-(trifluoromethyl)phenyl]methyl})-1,3-diazepan-2-one | HIV 1 | 10.1021/jm9602571 | Lam et al | ZINC26576005 | |
| 609 | (4R,5S,6S,7R)-4,7-dibenzyl-5,6-dihydroxy-1,3-bis({[4-(trifluoromethyl)phenyl]methyl})-1,3-diazepan-2-one | HIV 1 | 10.1021/jm9602571 | Lam et al | ZINC26576004 | |
| 610 | (4R,5S,6S,7R)-4,7-dibenzyl-5,6-dihydroxy-1,3-bis[(2-methoxyphenyl)methyl]-1,3-diazepan-2-one | HIV 1 | 10.1021/jm9602571 | Lam et al | ZINC14946448 | |
| 611 | (4R,5S,6S,7R)-4,7-dibenzyl-5,6-dihydroxy-1,3-bis[(3-methoxyphenyl)methyl]-1,3-diazepan-2-one | HIV 1 | 10.1021/jm9602571 | Lam et al | ZINC14946449 | |
| 612 | (4R,5S,6S,7R)-4,7-dibenzyl-5,6-dihydroxy-1,3-bis[(4-methoxyphenyl)methyl]-1,3-diazepan-2-one | HIV 1 | 10.1021/jm9602571 | Lam et al | ZINC14946450 | |
| 613 | (4R,5S,6S,7R)-4,7-dibenzyl-5,6-dihydroxy-1,3-bis[(3-iodophenyl)methyl]-1,3-diazepan-2-one | HIV 1 | 10.1021/jm9602571 | Lam et al | ZINC95544621 | |
| 614 | (4S,5R,6R,7S)-4,7-dibenzyl-5,6-dihydroxy-1,3-bis({[4-(hydroxymethyl)phenyl]methyl})-1,3-diazepan-2-one | HIV 1 | 10.1021/jm9602571 | Lam et al | ZINC14946451 | |
| 615 | (4R,5S,6S,7R)-4,7-dibenzyl-5,6-dihydroxy-1-(naphthalen-2-ylmethyl)-3-propyl-1,3-diazepan-2-one | HIV 1 | 10.1021/jm9602571 | Lam et al | ZINC14946452 | |
| 616 | (4R,5S,6S,7R)-4,7-dibenzyl-1-butyl-5,6-dihydroxy-3-(naphthalen-2-ylmethyl)-1,3-diazepan-2-one | HIV 1 | 10.1021/jm9602571 | Lam et al | ZINC14946453 | |
| 617 | (4R,5S,6S,7R)-4,7-dibenzyl-5,6-dihydroxy-1-(naphthalen-2-ylmethyl)-3-(prop-2-en-1-yl)-1,3-diazepan-2-one | HIV 1 | 10.1021/jm9602571 | Lam et al | ZINC14946454 | |
| 618 | (4R,5S,6S,7R)-4,7-dibenzyl-1-(cyclopropylmethyl)-5,6-dihydroxy-3-(naphthalen-2-ylmethyl)-1,3-diazepan-2-one | HIV 1 | 10.1021/jm9602571 | Lam et al | ZINC14946455 | |
| 619 | (4R,5S,6S,7R)-4,7-dibenzyl-1-cyclopentyl-5,6-dihydroxy-3-(naphthalen-2-ylmethyl)-1,3-diazepan-2-one | HIV 1 | 10.1021/jm9602571 | Lam et al | ZINC14946456 | |
| 620 | (4R,5S,6S,7R)-4,7-dibenzyl-5,6-dihydroxy-1-(naphthalen-2-ylmethyl)-3-(pyridin-3-ylmethyl)-1,3-diazepan-2-one | HIV 1 | 10.1021/jm9602571 | Lam et al | ZINC14946458 | |
| 621 | 3-[(4-amino-2-tert-butyl-5-methylphenyl)sulfanyl]-4-hydroxy-6-[2-(4-hydroxyphenyl)ethyl]-6-(propan-2-yl)-5,6-dihydro-2H-pyran-2-one | HIV 1 |  | n/a | ZINC29306160 | |
| 622 | 3-[(4-amino-2-tert-butyl-5-methylphenyl)sulfanyl]-4-hydroxy-6-[2-(4-hydroxyphenyl)ethyl]-6-methyl-5,6-dihydro-2H-pyran-2-one | HIV 1 |  | n/a | ZINC29253278 | |
| 623 | 3-[(4-amino-2-tert-butyl-5-methylphenyl)sulfanyl]-4-hydroxy-6-(2-phenylethyl)-6-(propan-2-yl)-5,6-dihydro-2H-pyran-2-one | HIV 1 |  | n/a | ZINC61959297 | |
| 624 | 3-[(2-tert-butyl-4-hydroxy-5-methylphenyl)sulfanyl]-4-hydroxy-6-[2-(4-hydroxyphenyl)ethyl]-6-(propan-2-yl)-5,6-dihydro-2H-pyran-2-one | HIV 1 |  | n/a | ZINC32037013 | |
| 625 | 5-tert-butyl-4-({4-hydroxy-6-[2-(4-hydroxyphenyl)ethyl]-2-oxo-6-(propan-2-yl)-5,6-dihydro-2H-pyran-3-yl}sulfanyl)-2-methylphenyl methanesulfonate | HIV 1 |  | n/a | ZINC27899058 | |
| 626 | 5-tert-butyl-4-({4-hydroxy-6-[2-(4-hydroxyphenyl)ethyl]-2-oxo-6-(propan-2-yl)-5,6-dihydro-2H-pyran-3-yl}sulfanyl)-2-methylphenyl benzenesulfonate | HIV 1 |  | n/a | ZINC27978854 | |
| 627 | 5-tert-butyl-4-({4-hydroxy-6-[2-(4-hydroxyphenyl)ethyl]-2-oxo-6-(propan-2-yl)-5,6-dihydro-2H-pyran-3-yl}sulfanyl)-2-methylphenyl 4-fluorobenzene-1-sulfonate | HIV 1 |  | n/a | ZINC27980846 | |
| 628 | 5-tert-butyl-4-({4-hydroxy-6-[2-(4-hydroxyphenyl)ethyl]-2-oxo-6-(propan-2-yl)-5,6-dihydro-2H-pyran-3-yl}sulfanyl)-2-methylphenyl 4-cyanobenzene-1-sulfonate | HIV 1 |  | n/a | ZINC27980019 | |
| 629 | 5-tert-butyl-4-({4-hydroxy-6-[2-(4-hydroxyphenyl)ethyl]-2-oxo-6-(propan-2-yl)-5,6-dihydro-2H-pyran-3-yl}sulfanyl)-2-methylphenyl thiophene-2-sulfonate | HIV 1 |  | n/a | ZINC27986501 | |
| 630 | 5-tert-butyl-4-({4-hydroxy-6-[2-(4-hydroxyphenyl)ethyl]-2-oxo-6-(propan-2-yl)-5,6-dihydro-2H-pyran-3-yl}sulfanyl)-2-methylphenyl 1-methyl-1H-imidazole-2-sulfonate | HIV 1 |  | n/a | ZINC70454627 | |
| 631 | 5-tert-butyl-4-({4-hydroxy-6-[2-(4-hydroxyphenyl)ethyl]-2-oxo-6-(propan-2-yl)-5,6-dihydro-2H-pyran-3-yl}sulfanyl)-2-methylphenyl pyridine-2-sulfonate | HIV 1 |  | n/a | ZINC27989655 | |
| 632 | 5-tert-butyl-4-({4-hydroxy-6-[2-(4-hydroxyphenyl)ethyl]-2-oxo-6-(propan-2-yl)-5,6-dihydro-2H-pyran-3-yl}sulfanyl)-2-methylphenyl pyridine-3-sulfonate | HIV 1 |  | n/a | ZINC27993875 | |
| 633 | 5-tert-butyl-4-({4-hydroxy-6-[2-(4-hydroxyphenyl)ethyl]-6-methyl-2-oxo-5,6-dihydro-2H-pyran-3-yl}sulfanyl)-2-methylphenyl benzenesulfonate | HIV 1 |  | n/a | ZINC27997320 | |
| 634 | 5-tert-butyl-4-({4-hydroxy-6-[2-(4-hydroxyphenyl)ethyl]-6-methyl-2-oxo-5,6-dihydro-2H-pyran-3-yl}sulfanyl)-2-methylphenyl 4-fluorobenzene-1-sulfonate | HIV 1 |  | n/a | ZINC27983632 | |
| 635 | 5-tert-butyl-4-({4-hydroxy-6-[2-(4-hydroxyphenyl)ethyl]-6-methyl-2-oxo-5,6-dihydro-2H-pyran-3-yl}sulfanyl)-2-methylphenyl 1-methyl-1H-imidazole-2-sulfonate | HIV 1 |  | n/a | ZINC70454629 | |
| 636 | 5-tert-butyl-4-({6-cyclohexyl-4-hydroxy-6-[2-(4-hydroxyphenyl)ethyl]-2-oxo-5,6-dihydro-2H-pyran-3-yl}sulfanyl)-2-methylphenyl benzenesulfonate | HIV 1 |  | n/a | ZINC96126909 | |
| 637 | 5-tert-butyl-4-({6-cyclohexyl-4-hydroxy-6-[2-(4-hydroxyphenyl)ethyl]-2-oxo-5,6-dihydro-2H-pyran-3-yl}sulfanyl)-2-methylphenyl 4-cyanobenzene-1-sulfonate | HIV 1 |  | n/a | ZINC96126911 | |
| 638 | 5-tert-butyl-4-({6-cyclohexyl-4-hydroxy-6-[2-(4-hydroxyphenyl)ethyl]-2-oxo-5,6-dihydro-2H-pyran-3-yl}sulfanyl)-2-methylphenyl 4-fluorobenzene-1-sulfonate | HIV 1 |  | n/a | ZINC96126913 | |
| 639 | 5-tert-butyl-4-({6-cyclohexyl-4-hydroxy-6-[2-(4-hydroxyphenyl)ethyl]-2-oxo-5,6-dihydro-2H-pyran-3-yl}sulfanyl)-2-methylphenyl 1-methyl-1H-imidazole-2-sulfonate | HIV 1 |  | n/a | ZINC96126739 | |
| 640 | 5-tert-butyl-4-({4-hydroxy-6-[2-(4-hydroxyphenyl)ethyl]-2-oxo-6-propyl-5,6-dihydro-2H-pyran-3-yl}sulfanyl)-2-methylphenyl 1-methyl-1H-imidazole-2-sulfonate | HIV 1 |  | n/a | ZINC70454658 | |
| 641 | 5-tert-butyl-4-({4-hydroxy-6-[2-(4-hydroxyphenyl)ethyl]-6-(2-methylpropyl)-2-oxo-5,6-dihydro-2H-pyran-3-yl}sulfanyl)-2-methylphenyl 1-methyl-1H-imidazole-2-sulfonate | HIV 1 |  | n/a | ZINC70454631 | |
| 642 | 3-[(2-tert-butyl-4-hydroxy-5-methylphenyl)sulfanyl]-4-hydroxy-6-(2-phenylethyl)-6-(propan-2-yl)-5,6-dihydro-2H-pyran-2-one | HIV 1 |  | n/a | ZINC61959299 | |
| 643 | 5-tert-butyl-4-{[4-hydroxy-2-oxo-6-(2-phenylethyl)-6-(propan-2-yl)-5,6-dihydro-2H-pyran-3-yl]sulfanyl}-2-methylphenyl 1-methyl-1H-imidazole-2-sulfonate | HIV 1 |  | n/a | ZINC70454633 | |
| 644 | 5-tert-butyl-4-{[4-hydroxy-2-oxo-6-(2-phenylethyl)-6-(propan-2-yl)-5,6-dihydro-2H-pyran-3-yl]sulfanyl}-2-methylphenyl pyridine-2-sulfonate | HIV 1 |  | n/a | ZINC27979613 | |
| 645 | 5-tert-butyl-4-{[4-hydroxy-2-oxo-6-(2-phenylethyl)-6-(propan-2-yl)-5,6-dihydro-2H-pyran-3-yl]sulfanyl}-2-methylphenyl pyridine-3-sulfonate | HIV 1 |  | n/a | ZINC27981927 | |
| 646 | 4-({6-[2-(4-aminophenyl)ethyl]-4-hydroxy-2-oxo-6-(propan-2-yl)-5,6-dihydro-2H-pyran-3-yl}sulfanyl)-5-tert-butyl-2-methylphenyl 4-cyanobenzene-1-sulfonate | HIV 1 |  | n/a | ZINC70454659 | |
| 647 | 4-({6-[2-(4-aminophenyl)ethyl]-4-hydroxy-2-oxo-6-(propan-2-yl)-5,6-dihydro-2H-pyran-3-yl}sulfanyl)-5-tert-butyl-2-methylphenyl 3-(pyridin-3-yl)benzene-1-sulfonate | HIV 1 |  | n/a | ZINC96126748 | |
| 648 | 4-({6-[2-(4-aminophenyl)ethyl]-4-hydroxy-2-oxo-6-(propan-2-yl)-5,6-dihydro-2H-pyran-3-yl}sulfanyl)-5-tert-butyl-2-methylphenyl 1-methyl-1H-imidazole-2-sulfonate | HIV 1 |  | n/a | ZINC70454635 | |
| 649 | 4-({6-[2-(4-aminophenyl)ethyl]-4-hydroxy-2-oxo-6-(propan-2-yl)-5,6-dihydro-2H-pyran-3-yl}sulfanyl)-5-tert-butyl-2-methylphenyl 2-(thiophen-2-yl)benzene-1-sulfonate | HIV 1 |  | n/a | ZINC96126752 | |
| 650 | 3-({2-tert-butyl-4-[(dimethylsulfamoyl)amino]-5-methylphenyl}sulfanyl)-4-hydroxy-6-[2-(4-hydroxyphenyl)ethyl]-6-(propan-2-yl)-5,6-dihydro-2H-pyran-2-one | HIV 1 |  | n/a | ZINC27980582 | |
| 651 | 3-({2-tert-butyl-4-[(ethylsulfamoyl)amino]-5-methylphenyl}sulfanyl)-4-hydroxy-6-[2-(4-hydroxyphenyl)ethyl]-6-(propan-2-yl)-5,6-dihydro-2H-pyran-2-one | HIV 1 |  | n/a | ZINC27994449 | |
| 652 | 3-({2-tert-butyl-4-[(ethylsulfamoyl)amino]-5-methylphenyl}sulfanyl)-4-hydroxy-6-(2-phenylethyl)-6-(propan-2-yl)-5,6-dihydro-2H-pyran-2-one | HIV 1 |  | n/a | ZINC64293151 | |
| 653 | 5-tert-butyl-4-({4-hydroxy-6-[2-(4-hydroxyphenyl)ethyl]-2-oxo-6-(propan-2-yl)-5,6-dihydro-2H-pyran-3-yl}sulfanyl)-2-methylphenyl N,N-dimethylsulfamate | HIV 1 |  | n/a | ZINC26376513 | |
| 654 | 5-tert-butyl-4-({4-hydroxy-6-[2-(4-hydroxyphenyl)ethyl]-2-oxo-6-(propan-2-yl)-5,6-dihydro-2H-pyran-3-yl}sulfanyl)-2-methylphenyl N-ethylsulfamate::6-Alkyl-5,6-Dihydropyran-2-one | HIV 1 |  | n/a | ZINC26285117 | |
| 655 | 4-({6-[2-(4-aminophenyl)ethyl]-4-hydroxy-2-oxo-6-(propan-2-yl)-5,6-dihydro-2H-pyran-3-yl}sulfanyl)-5-tert-butyl-2-methylphenyl N,N-dimethylsulfamate | HIV 1 |  | n/a | ZINC70454660 | |
| 656 | 4-({6-[2-(4-aminophenyl)ethyl]-4-hydroxy-2-oxo-6-(propan-2-yl)-5,6-dihydro-2H-pyran-3-yl}sulfanyl)-5-tert-butyl-2-methylphenyl N-ethylsulfamate | HIV 1 |  | n/a | ZINC70454661 | |
| 657 | 5-tert-butyl-4-{[4-hydroxy-2-oxo-6-(2-phenylethyl)-6-(propan-2-yl)-5,6-dihydro-2H-pyran-3-yl]sulfanyl}-2-methylphenyl N-ethylsulfamate | HIV 1 |  | n/a | ZINC49799685 | |
| 658 | 5-tert-butyl-4-{[(6R)-4-hydroxy-2-oxo-6-(2-phenylethyl)-6-(propan-2-yl)-5,6-dihydro-2H-pyran-3-yl]sulfanyl}-2-methylphenyl 4-cyanobenzene-1-sulfonate | HIV 1 |  | n/a | ZINC27994392 | |
| 659 | 5-tert-butyl-4-{[(6S)-4-hydroxy-6-[2-(4-hydroxyphenyl)ethyl]-2-oxo-6-(propan-2-yl)-5,6-dihydro-2H-pyran-3-yl]sulfanyl}-2-methylphenyl methanesulfonate | HIV 1 |  | n/a |  | |
| 660 | benzyl N-[(1S)-2-carbamoyl-1-{[(2S,3R)-3-hydroxy-4-[methyl(methylcarbamoyl)amino]-1-phenylbutan-2-yl]carbamoyl}ethyl]carbamate | HIV 1 |  | Getman et al | ZINC14943003 | |
| 661 | benzyl N-[(1S)-1-{[(2S,3R)-4-[(butylcarbamoyl)(methyl)amino]-3-hydroxy-1-phenylbutan-2-yl]carbamoyl}-2-carbamoylethyl]carbamate | HIV 1 |  | Getman et al | ZINC14943004 | |
| 662 | benzyl N-[(1S)-2-carbamoyl-1-{[(2S,3R)-3-hydroxy-4-[(methylcarbamoyl)(2-methylpropyl)amino]-1-phenylbutan-2-yl]carbamoyl}ethyl]carbamate | HIV 1 |  | Getman et al | ZINC14943005 | |
| 663 | benzyl N-[(1S)-1-{[(2S,3R)-4-[(butylcarbamoyl)(2-methylpropyl)amino]-3-hydroxy-1-phenylbutan-2-yl]carbamoyl}-2-carbamoylethyl]carbamate | HIV 1 |  | Getman et al | ZINC14943006 | |
| 664 | benzyl N-[(1S)-2-carbamoyl-1-{[(2S,3R)-3-hydroxy-4-[(2-methylpropyl)(propylcarbamoyl)amino]-1-phenylbutan-2-yl]carbamoyl}ethyl]carbamate | HIV 1 |  | Getman et al | ZINC14943008 | |
| 665 | benzyl N-[(1S)-2-carbamoyl-1-{[(2S,3R)-4-[(ethylcarbamoyl)(2-methylpropyl)amino]-3-hydroxy-1-phenylbutan-2-yl]carbamoyl}ethyl]carbamate | HIV 1 |  | Getman et al | ZINC14943009 | |
| 666 | benzyl N-[(1S)-2-carbamoyl-1-{[(2S,3R)-3-hydroxy-4-[(2-methylpropyl)(propan-2-ylcarbamoyl)amino]-1-phenylbutan-2-yl]carbamoyl}ethyl]carbamate | HIV 1 |  | Getman et al | ZINC14943010 | |
| 667 | :benzyl N-[(1S)-1-{[(2S,3R)-4-[(tert-butylcarbamoyl)(2-methylpropyl)amino]-3-hydroxy-1-phenylbutan-2-yl]carbamoyl}-2-carbamoylethyl]carbamate | HIV 1 |  | Getman et al | ZINC14943011 | |
| 668 | benzyl N-[(1S)-1-{[(2S,3S)-4-[(tert-butylcarbamoyl)(2-methylpropyl)amino]-3-hydroxy-1-phenylbutan-2-yl]carbamoyl}-2-carbamoylethyl]carbamate | HIV 1 |  | Getman et al | ZINC14943012 | |
| 669 | benzyl N-[(1S)-1-{[(2S,3R)-4-[(tert-butylcarbamoyl)(3-methylbutyl)amino]-3-hydroxy-1-phenylbutan-2-yl]carbamoyl}-2-carbamoylethyl]carbamate | HIV 1 |  | Getman et al | ZINC14943014 | |
| 670 | benzyl N-[(1S)-1-{[(2S,3R)-4-[(tert-butylcarbamoyl)(cyclohexylmethyl)amino]-3-hydroxy-1-phenylbutan-2-yl]carbamoyl}-2-carbamoylethyl]carbamate | HIV 1 |  | Getman et al | ZINC14943016 | |
| 671 | benzyl N-[(1S)-1-{[(2S,3R)-4-[benzyl(tert-butylcarbamoyl)amino]-3-hydroxy-1-phenylbutan-2-yl]carbamoyl}-2-carbamoylethyl]carbamate | HIV 1 |  | Getman et al | ZINC14943018 | |
| 672 | benzyl N-[(1S)-1-{[(2S,3R)-4-[(tert-butylcarbamoyl)[(1R)-1-phenylethyl]amino]-3-hydroxy-1-phenylbutan-2-yl]carbamoyl}-2-carbamoylethyl]carbamate | HIV 1 |  | Getman et al | ZINC14943020 | |
| 673 | benzyl N-[(1S)-1-{[(2S,3R)-4-[(tert-butylcarbamoyl)[(1S)-1-phenylethyl]amino]-3-hydroxy-1-phenylbutan-2-yl]carbamoyl}-2-carbamoylethyl]carbamate | HIV 1 |  | Getman et al | ZINC14943021 | |
| 674 | benzyl N-[(1S)-1-{[(2S,3R)-4-[(tert-butylcarbamoyl)(pyridin-4-ylmethyl)amino]-3-hydroxy-1-phenylbutan-2-yl]carbamoyl}-2-carbamoylethyl]carbamate | HIV 1 |  | Getman et al | ZINC14943022 | |
| 675 | benzyl N-[(1S)-2-carbamoyl-1-{[(2S,3S)-3-hydroxy-4-[methyl(methylcarbamoyl)amino]-1-phenylbutan-2-yl]carbamoyl}ethyl]carbamate | HIV 1 |  | Getman et al | ZINC14943023 | |
| 676 | benzyl N-[(1S)-1-{[(2S,3S)-4-[(butylcarbamoyl)(methyl)amino]-3-hydroxy-1-phenylbutan-2-yl]carbamoyl}-2-carbamoylethyl]carbamate | HIV 1 |  | Getman et al | ZINC14943024 | |
| 677 | benzyl N-[(1S)-2-carbamoyl-1-{[(2S,3S)-3-hydroxy-4-[(methylcarbamoyl)(2-methylpropyl)amino]-1-phenylbutan-2-yl]carbamoyl}ethyl]carbamate | HIV 1 |  | Getman et al | ZINC14943025 | |
| 678 | benzyl N-[(1S)-1-{[(2S,3S)-4-[(butylcarbamoyl)(2-methylpropyl)amino]-3-hydroxy-1-phenylbutan-2-yl]carbamoyl}-2-carbamoylethyl]carbamate | HIV 1 |  | Getman et al | ZINC14943026 | |
| 679 | 4-hydroxy-3-[(4-hydroxy-2-oxo-3,4-dihydro-2H-1-benzopyran-3-yl)sulfanyl]-2H-chromen-2-one | HIV 1 | 10.1021/jm950874+ | Wang et al | ZINC13604221 | |
| 680 | 3-benzyl-4,5-dihydroxy-2H-chromen-2-one | HIV 1 | 10.1021/jm950874+ | Wang et al | ZINC06575285 | |
| 681 | 3-[2H-1,3-benzodioxol-5-yl(4-hydroxy-2-oxo-2H-chromen-3-yl)methyl]-4-hydroxy-2H-chromen-2-one | HIV 1 | 10.1021/jm950874+ | Wang et al | ZINC04512503 | |
| 682 | 3-({4-[bis(4-hydroxy-2-oxo-2H-chromen-3-yl)methyl]phenyl}(4-hydroxy-2-oxo-2H-chromen-3-yl)methyl)-4-hydroxy-2H-chromen-2-one | HIV 1 | 10.1021/jm950874+ | Wang et al | ZINC95543764 | |
| 683 | 4-hydroxy-3,8-diphenyl-2H-chromen-2-one | HIV 1 | 10.1021/jm950874+ | Wang et al | ZINC06575897 | |
| 684 | 4-hydroxy-3-phenyl-2H-chromen-2-one | HIV 1 | 10.1021/jm950874+ | Wang et al | ZINC00068728 | |
| 685 | 4-cyano-N-(3-{1-[4-hydroxy-2-oxo-6-(2-phenylethyl)-6-propyl-5,6-dihydro-2H-pyran-3-yl]propyl}phenyl)benzene-1-sulfonamide | HIV 1 | 10.1021/jm960541s | Thaisrivongs et al | ZINC36124704 | |
| 686 | 5-cyano-N-{3-[(1R)-1-[(6S)-4-hydroxy-2-oxo-6-(2-phenylethyl)-6-propyl-5,6-dihydro-2H-pyran-3-yl]propyl]phenyl}pyridine-2-sulfonamide | HIV 1 | 10.1021/jm960541s | Thaisrivongs et al | ZINC36124711 | |
| 687 | 5-cyano-N-{3-[(1R)-1-[(6R)-4-hydroxy-2-oxo-6-(2-phenylethyl)-6-propyl-5,6-dihydro-2H-pyran-3-yl]propyl]phenyl}pyridine-2-sulfonamide | HIV 1 | 10.1021/jm960541s | Thaisrivongs et al | ZINC70454636 | |
| 688 | 5-cyano-N-{3-[(1S)-1-[(6S)-4-hydroxy-2-oxo-6-(2-phenylethyl)-6-propyl-5,6-dihydro-2H-pyran-3-yl]propyl]phenyl}pyridine-2-sulfonamide | HIV 1 | 10.1021/jm960541s | Thaisrivongs et al | ZINC36124707 | |
| 689 | 5-cyano-N-{3-[(1S)-1-[(6R)-4-hydroxy-2-oxo-6-(2-phenylethyl)-6-propyl-5,6-dihydro-2H-pyran-3-yl]propyl]phenyl}pyridine-2-sulfonamide | HIV 1 | 10.1021/jm960541s | Thaisrivongs et al | ZINC08536339 | |
| 690 | 3-{4-[(2S,3R)-2-{[(tert-butoxy)carbonyl]amino}-4-{[(2R,3S)-3-{[(tert-butoxy)carbonyl]amino}-2-hydroxy-4-phenylbutyl]amino}-3-hydroxybutyl]phenoxy}propanoic acid | HIV 1 | 10.1021/jm950717a | Chen et al | ZINC14943113 | |
| 691 | 4-Cyano-N-[3-[cyclopropyl(5,6,7,8,9,10-hexahydro-4-hydroxy-2-oxo-2H-cycloocta[b]pyran-3-yl)methyl]phenyl]-benzenesulfonamide::4-cyano-N-{3-[(S)-cyclopropyl({4-hydroxy-2-oxo-2H,5H,6H,7H,8H,9H,10H-cycloocta[b]pyran-3-yl})methyl]phenyl}benzene-1-sulfonamide | HIV 1 |  | Skulnick et al | ZINC03873405 | |
| 692 | 4-hydroxy-3-(1-phenylethyl)-2H,5H,6H,7H,8H,9H,10H-cycloocta[b]pyran-2-one | HIV 1 |  | Romines et al | ZINC14943171 | |
| 693 | 4-hydroxy-3-(1-phenylpropyl)-2H,5H,6H,7H,8H,9H,10H-cycloocta[b]pyran-2-one::5,6,7,8,9,10-Hexahydro-4-hydroxy-3-( 1-phenylpropyl).W-cycloocta[b]pyran-2-one | HIV 1 |  | Romines et al | ZINC13741432 | |
| 694 | 4-hydroxy-3-(1-phenylbutyl)-2H,5H,6H,7H,8H,9H,10H-cycloocta[b]pyran-2-one::5,6,7,8,9,l0-Hexahydro-4-hydroxy-3-(l-phenylbutyl)-W-cycloocta[blpyran-2-one | HIV 1 |  | Romines et al | ZINC13741439 | |
| 695 | 4-hydroxy-3-(2-methyl-1-phenylpropyl)-2H,5H,6H,7H,8H,9H,10H-cycloocta[b]pyran-2-one::5,6,7,8,9,1O-Hexahydro-4-hydroxy-3-(2-methyl-nylpropyl)-W-cycloocta[b]pyran-2-one | HIV 1 |  | Romines et al | ZINC13741441 | |
| 696 | 3-[cyclopropyl(phenyl)methyl]-4-hydroxy-2H,5H,6H,7H,8H,9H,10H-cycloocta[b]pyran-2-one | HIV 1 |  | Romines et al | ZINC13741434 | |
| 697 | 4-hydroxy-3-(1-phenylpentyl)-2H,5H,6H,7H,8H,9H,10H-cycloocta[b]pyran-2-one::5,6,7,8,9,10-Hexahydroro-4-hydroxy- 1-phenylpentyl)-W-cycloocta[blpyran-2-one | HIV 1 |  | Romines et al | ZINC13741443 | |
| 698 | 4-hydroxy-3-(3-methyl-1-phenylbutyl)-2H,5H,6H,7H,8H,9H,10H-cycloocta[b]pyran-2-one::5,6,7,8,9,1O-Hexahydro-4-hydroxy-3-(3-methyl- l-phe-nylbutyl)-W-cycloocta[b]pyran-2-one | HIV 1 |  | Romines et al | ZINC13741445 | |
| 699 | 3-benzyl-4-hydroxy-2H,5H,6H,7H,8H,9H,10H-cycloocta[b]pyran-2-one | HIV 1 |  | Romines et al | ZINC14943172 | |
| 700 | 4-hydroxy-3-(2-phenylethyl)-2H,5H,6H,7H,8H,9H,10H-cycloocta[b]pyran-2-one | HIV 1 |  | Romines et al | ZINC14943173 | |
| 701 | 4-hydroxy-3-(3-phenylpropyl)-2H,5H,6H,7H,8H,9H,10H-cycloocta[b]pyran-2-one | HIV 1 |  | Romines et al | ZINC14943174 | |
| 702 | benzyl N-[(1S)-1-{[(1S,2S)-1-[(9R,12R)-8,11-dioxo-9-(propan-2-yl)-2-oxa-7,10,13-triazabicyclo[13.2.2]nonadeca-1(17),15,18-trien-12-yl]-1-hydroxy-3-phenylpropan-2-yl]carbamoyl}-2-methylpropyl]carbamate | HIV 1 | 10.1021/jm950641i | Ettmayer et al | ZINC96126793 | |
| 703 | benzyl N-[(1S)-1-{[(1S,2S)-1-[(10R,13R)-9,12-dioxo-10-(propan-2-yl)-2,5-dioxa-8,11,14-triazabicyclo[14.2.2]icosa-1(18),16,19-trien-13-yl]-1-hydroxy-3-phenylpropan-2-yl]carbamoyl}-2-methylpropyl]carbamate | HIV 1 | 10.1021/jm950641i | Ettmayer et al | ZINC96126801 | |
| 704 | benzyl N-[(1S)-1-{[(1S,2S)-1-[(13R,16R)-12,15-dioxo-13-(propan-2-yl)-2,5,8-trioxa-11,14,17-triazabicyclo[17.2.2]tricosa-1(21),19,22-trien-16-yl]-1-hydroxy-3-phenylpropan-2-yl]carbamoyl}-2-methylpropyl]carbamate | HIV 1 | 10.1021/jm950641i | Ettmayer et al | ZINC96126806 | |
| 705 | benzyl N-[(1S)-1-{[(1S,2S)-1-[(16R,19R)-15,18-dioxo-16-(propan-2-yl)-2,5,8,11-tetraoxa-14,17,20-triazabicyclo[20.2.2]hexacosa-1(24),22,25-trien-19-yl]-1-hydroxy-3-phenylpropan-2-yl]carbamoyl}-2-methylpropyl]carbamate | HIV 1 | 10.1021/jm950641i | Ettmayer et al | ZINC96126812 | |
| 706 | tert-butyl N-[(1S,2S)-1-[(9R,12R)-8,11-dioxo-9-(propan-2-yl)-2-oxa-7,10,13-triazabicyclo[13.2.2]nonadeca-1(17),15,18-trien-12-yl]-1-hydroxy-3-phenylpropan-2-yl]carbamate | HIV 1 | 10.1021/jm950641i | Ettmayer et al | ZINC14943192 | |
| 707 | tert-butyl N-[(1S,2S)-1-[(13R,16R)-12,15-dioxo-13-(propan-2-yl)-2,5,8-trioxa-11,14,17-triazabicyclo[17.2.2]tricosa-1(21),19,22-trien-16-yl]-1-hydroxy-3-phenylpropan-2-yl]carbamate | HIV 1 | 10.1021/jm950641i | Ettmayer et al | ZINC14943200 | |
| 708 | tert-butyl N-[(1S,2S)-1-[(16R,19R)-15,18-dioxo-16-(propan-2-yl)-2,5,8,11-tetraoxa-14,17,20-triazabicyclo[20.2.2]hexacosa-1(24),22,25-trien-19-yl]-1-hydroxy-3-phenylpropan-2-yl]carbamate | HIV 1 | 10.1021/jm950641i | Ettmayer et al | ZINC96126817 | |
| 709 | benzyl N-[(1S)-1-{[(1S,2S)-1-[(9R,12R)-8,11-dioxo-9-(propan-2-yl)-2-oxa-7,10,13-triazabicyclo[13.3.1]nonadeca-1(18),15(19),16-trien-12-yl]-1-hydroxy-3-phenylpropan-2-yl]carbamoyl}-2-methylpropyl]carbamate | HIV 1 | 10.1021/jm950641i | Ettmayer et al | ZINC96126824 | |
| 710 | benzyl N-[(1S)-1-{[(1S,2S)-1-[(10R,13R)-9,12-dioxo-10-(propan-2-yl)-2,5-dioxa-8,11,14-triazabicyclo[14.3.1]icosa-1(19),16(20),17-trien-13-yl]-1-hydroxy-3-phenylpropan-2-yl]carbamoyl}-2-methylpropyl]carbamate | HIV 1 | 10.1021/jm950641i | Ettmayer et al | ZINC96126832 | |
| 711 | benzyl N-[(1S)-1-{[(1S,2S)-1-[(13R,16R)-12,15-dioxo-13-(propan-2-yl)-2,5,8-trioxa-11,14,17-triazabicyclo[17.3.1]tricosa-1(22),19(23),20-trien-16-yl]-1-hydroxy-3-phenylpropan-2-yl]carbamoyl}-2-methylpropyl]carbamate | HIV 1 | 10.1021/jm950641i | Ettmayer et al | ZINC96126840 | |
| 712 | tert-butyl N-[(1S,2S)-1-[(9R,12R)-8,11-dioxo-9-(propan-2-yl)-2-oxa-7,10,13-triazabicyclo[13.3.1]nonadeca-1(18),15(19),16-trien-12-yl]-1-hydroxy-3-phenylpropan-2-yl]carbamate | HIV 1 | 10.1021/jm950641i | Ettmayer et al | ZINC14943204 | |
| 713 | tert-butyl N-[(1S,2S)-1-[(10R,13R)-9,12-dioxo-10-(propan-2-yl)-2,5-dioxa-8,11,14-triazabicyclo[14.3.1]icosa-1(19),16(20),17-trien-13-yl]-1-hydroxy-3-phenylpropan-2-yl]carbamate | HIV 1 | 10.1021/jm950641i | Ettmayer et al | ZINC14943208 | |
| 714 | tert-butyl N-[(1S,2S)-1-[(13R,16R)-12,15-dioxo-13-(propan-2-yl)-2,5,8-trioxa-11,14,17-triazabicyclo[17.3.1]tricosa-1(22),19(23),20-trien-16-yl]-1-hydroxy-3-phenylpropan-2-yl]carbamate | HIV 1 | 10.1021/jm950641i | Ettmayer et al | ZINC14943212 | |
| 715 | benzyl N-[(1S)-1-{[(1S,2S)-1-[(10R,13R)-9,12-dioxo-10-(propan-2-yl)-2,5-dioxa-8,11,14-triazabicyclo[14.2.2]icosa-1(18),16,19-trien-13-yl]-1-hydroxy-3-phenylpropan-2-yl]carbamoyl}-2,2-dimethylpropyl]carbamate | HIV 1 | 10.1021/jm950641i | Ettmayer et al | ZINC96126848 | |
| 716 | benzyl N-[(1S)-1-{[(2S,3S,4R)-4-{[(1R)-1-[(1H-1,3-benzodiazol-2-ylmethyl)carbamoyl]-2-methylpropyl]carbamoyl}-3-hydroxy-4-{[(4-methoxyphenyl)methyl]amino}-1-phenylbutan-2-yl]carbamoyl}-2-methylpropyl]carbamate | HIV 1 | 10.1021/jm950641i | Ettmayer et al | ZINC96126871 | |
| 717 | benzyl N-[(1S)-1-{[(2S,3S,4R)-3-hydroxy-4-{[(1S,2S)-2-hydroxy-2,3-dihydro-1H-inden-1-yl]carbamoyl}-4-{[(4-methoxyphenyl)methyl]amino}-1-phenylbutan-2-yl]carbamoyl}-2,2-dimethylpropyl]carbamate | HIV 1 | 10.1021/jm950641i | Ettmayer et al | ZINC96126878 | |
| 718 | 4-(2-{4-[(2R)-2-[(2S,3S)-3-{[(tert-butoxy)carbonyl]amino}-2-hydroxy-4-phenylbutyl]-2-{[(1S,2R)-2-hydroxy-2,3-dihydro-1H-inden-1-yl]carbamoyl}ethyl]phenoxy}ethyl)-4-hydroxymorpholin-4-ium | HIV 1 |  | Thompson et al | ZINC96126879 | |
| 719 | (4S,5S)-5-[(2S)-2-[(2S)-2-aminopropanamido]propanamido]-N-(diphenylmethyl)-4-hydroxy-6-phenylhexanamide::5-(L-Alanyl-L-alanylamino)-4-hydroxy-6-phenylhexanoic Acid Benzhydrylamide | HIV 1 |  | Varney et al | ZINC14943220 | |
| 720 | (4S,5S)-5-[(2S)-2-[(2S)-2-aminopropanamido]propanamido]-4-hydroxy-N-[1H-indol-2-yl(phenyl)methyl]-6-phenylhexanamide | HIV 1 |  | Varney et al | ZINC14943222 | |
| 721 | (4S,5S)-5-[(2S)-2-[(2S)-2-aminopropanamido]propanamido]-N-[1H-1,3-benzodiazol-2-yl(phenyl)methyl]-4-hydroxy-6-phenylhexanamide | HIV 1 |  | Varney et al | ZINC14943224 | |
| 722 | benzyl N-[(1S)-1-{[(2S,3R,4R)-4-(benzylamino)-4-{[(1S)-1-(benzylcarbamoyl)-2-methylpropyl]carbamoyl}-3-hydroxy-1-phenylbutan-2-yl]carbamoyl}-2-methylpropyl]carbamate | HIV 1 |  | Scholz et al | ZINC26826059 | |
| 723 | benzyl N-[(1S)-1-{[(2S,3R,4R)-4-{[(1S)-1-(benzylcarbamoyl)-2-methylpropyl]carbamoyl}-4-(benzylsulfanyl)-3-hydroxy-1-phenylbutan-2-yl]carbamoyl}-2-methylpropyl]carbamate | HIV 1 |  | Scholz et al | ZINC95540762 | |
| 724 | benzyl N-[(1S)-1-{[(2S,3R,4R)-4-{[(1S)-1-(benzylcarbamoyl)-2-methylpropyl]carbamoyl}-3-hydroxy-1-phenyl-4-(phenylamino)butan-2-yl]carbamoyl}-2-methylpropyl]carbamate | HIV 1 |  | Scholz et al | ZINC26829536 | |
| 725 | benzyl N-[(1S)-1-{[(2S,3R,4R)-4-{[(1S)-1-(benzylcarbamoyl)-2-methylpropyl]carbamoyl}-3-hydroxy-1-phenyl-4-[(2-phenylethyl)amino]butan-2-yl]carbamoyl}-2-methylpropyl]carbamate | HIV 1 |  | Scholz et al | ZINC26829458 | |
| 726 | benzyl N-[(1S)-1-{[(2S,3R,4R)-4-{[(1S)-1-(benzylcarbamoyl)-2-methylpropyl]carbamoyl}-4-(butylamino)-3-hydroxy-1-phenylbutan-2-yl]carbamoyl}-2-methylpropyl]carbamate | HIV 1 |  | Scholz et al | ZINC26828276 | |
| 727 | benzyl N-[(1S)-1-{[(2S,3R,4R)-4-{[(1S)-1-(benzylcarbamoyl)-2-methylpropyl]carbamoyl}-4-(cyclohexylamino)-3-hydroxy-1-phenylbutan-2-yl]carbamoyl}-2-methylpropyl]carbamate | HIV 1 |  | Scholz et al | ZINC26833046 | |
| 728 | benzyl N-[(1S)-1-{[(2S,3R,4R)-4-{[(1S)-1-(benzylcarbamoyl)-2-methylpropyl]carbamoyl}-3-hydroxy-4-[(naphthalen-1-ylmethyl)amino]-1-phenylbutan-2-yl]carbamoyl}-2-methylpropyl]carbamate | HIV 1 |  | Scholz et al | ZINC95546119 | |
| 729 | benzyl N-[(1S)-1-{[(2S,3R,4R)-4-{[(1S)-1-(benzylcarbamoyl)-2-methylpropyl]carbamoyl}-3-hydroxy-1-phenyl-4-[(4-phenylphenyl)amino]butan-2-yl]carbamoyl}-2-methylpropyl]carbamate | HIV 1 |  | Scholz et al | ZINC95546182 | |
| 730 | benzyl N-[(1S)-1-{[(2S,3R,4R)-4-{[(1S)-1-(benzylcarbamoyl)-2-methylpropyl]carbamoyl}-3-hydroxy-4-{[2-(1H-indol-3-yl)ethyl]amino}-1-phenylbutan-2-yl]carbamoyl}-2-methylpropyl]carbamate | HIV 1 |  | Scholz et al | ZINC95546158 | |
| 731 | benzyl N-[(1S)-1-{[(2S,3R,4R)-4-{[(1S)-1-(benzylcarbamoyl)-2-methylpropyl]carbamoyl}-3-hydroxy-1-phenyl-4-{[2-(pyridin-2-yl)ethyl]amino}butan-2-yl]carbamoyl}-2-methylpropyl]carbamate | HIV 1 |  | Scholz et al | ZINC95610538 | |
| 732 | benzyl N-[(1S)-1-{[(2S,3R,4R)-4-{[(1S)-1-(benzylcarbamoyl)-2-methylpropyl]carbamoyl}-3-hydroxy-4-{[(4-methoxyphenyl)methyl]amino}-1-phenylbutan-2-yl]carbamoyl}-2-methylpropyl]carbamate | HIV 1 |  | Scholz et al | ZINC95546171 | |
| 733 | benzyl N-[(1S)-1-{[(2S,3R,4R)-4-{[(1S)-1-(benzylcarbamoyl)-2-methylpropyl]carbamoyl}-4-{[(4-chlorophenyl)methyl]amino}-3-hydroxy-1-phenylbutan-2-yl]carbamoyl}-2-methylpropyl]carbamate | HIV 1 |  | Scholz et al | ZINC95546170 | |
| 734 | benzyl N-[(1S)-1-{[(2S,3R,4R)-4-{[(1S)-1-(benzylcarbamoyl)-2-methylpropyl]carbamoyl}-4-{[(4-bromophenyl)methyl]amino}-3-hydroxy-1-phenylbutan-2-yl]carbamoyl}-2-methylpropyl]carbamate | HIV 1 |  | Scholz et al | ZINC95540660 | |
| 735 | benzyl N-[(1S)-1-{[(2S,3R,4R)-4-(benzylamino)-4-{[(1S)-1-(benzylcarbamoyl)-2-methylpropyl]carbamoyl}-3-hydroxy-1-phenylbutan-2-yl]carbamoyl}ethyl]carbamate | HIV 1 |  | Scholz et al | ZINC26827277 | |
| 736 | benzyl N-[(1S)-1-{[(2S,3R,4R)-4-(benzylamino)-4-{[(1S)-1-(benzylcarbamoyl)-2-methylpropyl]carbamoyl}-3-hydroxy-1-phenylbutan-2-yl]carbamoyl}-3-methylbutyl]carbamate | HIV 1 |  | Scholz et al | ZINC26827273 | |
| 737 | benzyl N-[(1S,2R)-1-{[(2S,3R,4R)-4-(benzylamino)-4-{[(1S)-1-(benzylcarbamoyl)-2-methylpropyl]carbamoyl}-3-hydroxy-1-phenylbutan-2-yl]carbamoyl}-2-methylbutyl]carbamate | HIV 1 |  | Scholz et al | ZINC26830886 | |
| 738 | benzyl N-[(1S)-1-{[(2S,3R,4R)-4-(benzylamino)-4-{[(1S)-1-(benzylcarbamoyl)-2-methylpropyl]carbamoyl}-3-hydroxy-1-phenylbutan-2-yl]carbamoyl}-2,2-dimethylpropyl]carbamate | HIV 1 |  | Scholz et al | ZINC26834399 | |
| 739 | benzyl N-[(1S)-1-{[(2S,3R,4R)-4-(benzylamino)-4-{[(1S)-1-(benzylcarbamoyl)-2-methylpropyl]carbamoyl}-3-hydroxy-1-phenylbutan-2-yl]carbamoyl}propyl]carbamate | HIV 1 |  | Scholz et al | ZINC26835569 | |
| 740 | benzyl N-[(1S)-1-{[(2S,3R,4R)-4-(benzylamino)-4-{[(1S)-1-(benzylcarbamoyl)-2-methylpropyl]carbamoyl}-3-hydroxy-1-phenylbutan-2-yl]carbamoyl}-2-(1H-indol-3-yl)ethyl]carbamate | HIV 1 |  | Scholz et al | ZINC95546168 | |
| 741 | benzyl N-[(S)-{[(2S,3R,4R)-4-(benzylamino)-4-{[(1S)-1-(benzylcarbamoyl)-2-methylpropyl]carbamoyl}-3-hydroxy-1-phenylbutan-2-yl]carbamoyl}(phenyl)methyl]carbamate | HIV 1 |  | Scholz et al | ZINC95546160 | |
| 742 | benzyl N-[(1S)-1-{[(2S,3R,4R)-4-(benzylamino)-4-{[(1S)-1-(benzylcarbamoyl)-2-methylpropyl]carbamoyl}-3-hydroxy-1-phenylbutan-2-yl]carbamoyl}-2-carbamoylethyl]carbamate | HIV 1 |  | Scholz et al | ZINC95542273 | |
| 743 | benzyl N-[(1S)-1-{[(2S,3R,4R)-4-(benzylamino)-4-{[(1S)-1-(benzylcarbamoyl)-2-methylpropyl]carbamoyl}-3-hydroxy-1-phenylbutan-2-yl]carbamoyl}-2-hydroxyethyl]carbamate | HIV 1 |  | Scholz et al | ZINC26834800 | |
| 744 | benzyl N-[(1S)-1-{[(2S,3R,4R)-4-(benzylamino)-4-{[(1S)-1-(benzylcarbamoyl)-2-methylpropyl]carbamoyl}-3-hydroxy-1-phenylbutan-2-yl]carbamoyl}-2-(1H-imidazol-4-yl)ethyl]carbamate | HIV 1 |  | Scholz et al | ZINC95610625 | |
| 745 | benzyl N-[(1S)-1-{[(2S,3R,4R)-4-(benzylamino)-4-{[(1S)-1-(benzylcarbamoyl)-3-methylbutyl]carbamoyl}-3-hydroxy-1-phenylbutan-2-yl]carbamoyl}-2-methylpropyl]carbamate | HIV 1 |  | Scholz et al | ZINC26834529 | |
| 746 | benzyl N-[(1S)-1-{[(2S,3R,4R)-4-(benzylamino)-4-{[(1S,2R)-1-(benzylcarbamoyl)-2-methylbutyl]carbamoyl}-3-hydroxy-1-phenylbutan-2-yl]carbamoyl}-2-methylpropyl]carbamate | HIV 1 |  | Scholz et al | ZINC26837790 | |
| 747 | benzyl N-[(1S)-1-{[(2S,3R,4R)-4-(benzylamino)-4-{[(1S)-1-(benzylcarbamoyl)-2,2-dimethylpropyl]carbamoyl}-3-hydroxy-1-phenylbutan-2-yl]carbamoyl}-2-methylpropyl]carbamate | HIV 1 |  | Scholz et al | ZINC26837781 | |
| 748 | 6-amino-N-[(1S)-1-{[(2S,3R,4R)-4-(benzylamino)-4-{[(1S)-1-(benzylcarbamoyl)-2-methylpropyl]carbamoyl}-3-hydroxy-1-phenylbutan-2-yl]carbamoyl}-2-methylpropyl]hexanamide | HIV 1 |  | Scholz et al | ZINC26825996 | |
| 749 | pyridin-2-ylmethyl N-[(1S)-1-{[(2S,3R,4R)-4-(benzylamino)-4-{[(1S)-1-(benzylcarbamoyl)-2-methylpropyl]carbamoyl}-3-hydroxy-1-phenylbutan-2-yl]carbamoyl}-2-methylpropyl]carbamate | HIV 1 |  | Scholz et al | ZINC26840753 | |
| 750 | ethyl (2S)-2-[(2R,3R,4S)-2-(benzylamino)-4-[(2S)-2-{[(benzyloxy)carbonyl]amino}-3-methylbutanamido]-3-hydroxy-5-phenylpentanamido]-3-methylbutanoate | HIV 1 |  | Scholz et al | ZINC26836820 | |
| 751 | benzyl N-[(1S)-1-{[(2S,3R,4R)-4-(benzylamino)-4-{[(1S)-1-(tert-butylcarbamoyl)-2-methylpropyl]carbamoyl}-3-hydroxy-1-phenylbutan-2-yl]carbamoyl}-2-methylpropyl]carbamate | HIV 1 |  | Scholz et al | ZINC26837765 | |
| 752 | benzyl N-[(1S)-1-{[(2S,3R,4R)-4-(benzylamino)-3-hydroxy-4-{[(1S)-2-methyl-1-[(pyridin-2-ylmethyl)carbamoyl]propyl]carbamoyl}-1-phenylbutan-2-yl]carbamoyl}-2-methylpropyl]carbamate | HIV 1 |  | Scholz et al | ZINC26837761 | |
| 753 | benzyl N-[(1S)-1-{[(2S,3R,4R)-4-{[(1S)-1-[(1H-1,3-benzodiazol-2-ylmethyl)carbamoyl]-2-methylpropyl]carbamoyl}-4-(benzylamino)-3-hydroxy-1-phenylbutan-2-yl]carbamoyl}-2-methylpropyl]carbamate | HIV 1 |  | Scholz et al | ZINC95546172 | |
| 754 | benzyl N-[(1S)-1-{[(2S,3R,4R)-4-(benzylamino)-3-hydroxy-4-{[(1S)-1-{[2-(1H-indol-3-yl)ethyl]carbamoyl}-2-methylpropyl]carbamoyl}-1-phenylbutan-2-yl]carbamoyl}-2-methylpropyl]carbamate | HIV 1 |  | Scholz et al | ZINC95546183 | |
| 755 | benzyl N-[(1S)-1-{[(2S,3R,4R)-4-{[(1S)-1-[(1H-1,3-benzodiazol-2-ylmethyl)carbamoyl]-2-methylpropyl]carbamoyl}-3-hydroxy-4-{[(4-methoxyphenyl)methyl]amino}-1-phenylbutan-2-yl]carbamoyl}-2-methylpropyl]carbamate | HIV 1 |  | Scholz et al | ZINC95610694 | |
| 756 | benzyl N-[(1S)-1-{[(2S,3R,4R)-4-{[(1S)-1-[(1H-1,3-benzodiazol-2-ylmethyl)carbamoyl]-2-methylpropyl]carbamoyl}-4-{[(4-chlorophenyl)methyl]amino}-3-hydroxy-1-phenylbutan-2-yl]carbamoyl}-2-methylpropyl]carbamate | HIV 1 |  | Scholz et al | ZINC95546173 | |
| 757 | benzyl N-[(1S)-1-{[(2S,3R,4R)-4-{[(1S)-1-[(1H-1,3-benzodiazol-2-ylmethyl)carbamoyl]-2-methylpropyl]carbamoyl}-4-{[(4-chlorophenyl)methyl]amino}-3-hydroxy-1-phenylbutan-2-yl]carbamoyl}-2,2-dimethylpropyl]carbamate | HIV 1 |  | Scholz et al | ZINC95610539 | |
| 758 | benzyl N-[(1S)-1-{[(2S,3R,4R)-4-{[(1S)-1-[(1H-1,3-benzodiazol-2-ylmethyl)carbamoyl]-2-methylpropyl]carbamoyl}-3-hydroxy-4-{[(4-methoxyphenyl)methyl]amino}-1-phenylbutan-2-yl]carbamoyl}-2,2-dimethylpropyl]carbamate | HIV 1 |  | Scholz et al | ZINC95537937 | |
| 759 | benzyl N-[(1S)-1-{[(2S,3R,4R)-4-(benzylamino)-4-{[(1R)-1-(benzylcarbamoyl)-2,2-dimethylpropyl]carbamoyl}-3-hydroxy-1-phenylbutan-2-yl]carbamoyl}-2-methylpropyl]carbamate | HIV 1 |  | Scholz et al | ZINC26837777 | |
| 760 | benzyl N-[(1S)-1-{[(2S,3R,4R)-4-(benzylamino)-4-{[(1S)-1-(benzylcarbamoyl)-2-carbamoylethyl]carbamoyl}-3-hydroxy-1-phenylbutan-2-yl]carbamoyl}-2-methylpropyl]carbamate | HIV 1 |  | Scholz et al | ZINC95541742 | |
| 761 | tert-butyl N-[(1S)-1-{[(2S,3R,4R)-4-(benzylamino)-4-{[(1S)-1-(benzylcarbamoyl)-2-methylpropyl]carbamoyl}-3-hydroxy-1-phenylbutan-2-yl]carbamoyl}-2-methylpropyl]carbamate | HIV 1 |  | Scholz et al | ZINC26826991 | |
| 762 | 4-hydroxy-3-(3-phenoxypropyl)-2H-chromen-2-one | HIV 1 |  | Lunney et al | ZINC13738186 | |
| 763 | 4-hydroxy-3-(4-phenylbutyl)-2H-chromen-2-one | HIV 1 |  | Lunney et al | ZINC13738187 | |
| 764 | 4-hydroxy-3-[3-(phenylsulfanyl)propyl]-2H-chromen-2-one | HIV 1 |  | Lunney et al | ZINC13738188 | |
| 765 | 3-{3-[(4-fluorophenyl)(phenyl)amino]propyl}-4-hydroxy-2H-chromen-2-one | HIV 1 |  | Lunney et al | ZINC13738189 | |
| 766 | 3-{3-[benzyl(phenyl)amino]propyl}-4-hydroxy-2H-chromen-2-one | HIV 1 |  | Lunney et al | ZINC13738190 | |
| 767 | 3-[2-(benzyloxy)ethyl]-4-hydroxy-2H-chromen-2-one | HIV 1 |  | Lunney et al | ZINC13738191 | |
| 768 | 4-hydroxy-3-[4-(4-methoxyphenyl)butyl]-2H-chromen-2-one | HIV 1 |  | Lunney et al | ZINC13738192 | |
| 769 | 4-hydroxy-3-[4-(2-methoxyphenyl)butyl]-2H-chromen-2-one | HIV 1 |  | Lunney et al | ZINC13738193 | |
| 770 | 4-hydroxy-3-(2-phenoxyethyl)-2H-chromen-2-one | HIV 1 |  | Lunney et al | ZINC13738194 | |
| 771 | 4-hydroxy-3-(4-phenoxybutyl)-2H-chromen-2-one | HIV 1 |  | Lunney et al | ZINC13738195 | |
| 772 | 3-[3-(benzyloxy)propyl]-4-hydroxy-2H-chromen-2-one | HIV 1 |  | Lunney et al | ZINC13738196 | |
| 773 | 4-hydroxy-3-[3-(2-methoxyphenoxy)propyl]-2H-chromen-2-one | HIV 1 |  | Lunney et al | ZINC13738197 | |
| 774 | 4-hydroxy-3-[3-(3-nitrophenoxy)propyl]-2H-chromen-2-one | HIV 1 |  | Lunney et al | ZINC13738198 | |
| 775 | 3-[3-(3-aminophenoxy)propyl]-4-hydroxy-2H-chromen-2-one | HIV 1 |  | Lunney et al | ZINC13738199 | |
| 776 | 3-[3-(4-hydroxy-2-oxo-2H-chromen-3-yl)propoxy]benzoic acid | HIV 1 |  | Lunney et al | ZINC13738201 | |
| 777 | 4-hydroxy-3-{3-[3-(hydroxymethyl)phenoxy]propyl}-2H-chromen-2-one | HIV 1 |  | Lunney et al | ZINC13738202 | |
| 778 | 3-[3-(3-chlorophenoxy)propyl]-4-hydroxy-2H-chromen-2-one | HIV 1 |  | Lunney et al | ZINC13738203 | |
| 779 | 3-[3-(4-hydroxy-2-oxo-2H-chromen-3-yl)propoxy]benzonitrile | HIV 1 |  | Lunney et al | ZINC13738204 | |
| 780 | 3-[3-(4-hydroxy-2-oxo-2H-chromen-3-yl)propoxy]benzamide | HIV 1 |  | Lunney et al | ZINC13738205 | |
| 781 | 4,7-dihydroxy-3-[4-(2-methoxyphenyl)butyl]-2H-chromen-2-one | HIV 1 |  | Lunney et al | ZINC13738206 | |
| 782 | 4-hydroxy-7-methoxy-3-[4-(2-methoxyphenyl)butyl]-2H-chromen-2-one | HIV 1 |  | Lunney et al | ZINC13738208 | |
| 783 | 3-[3-(cyclohexyloxy)propyl]-4-hydroxy-2H-chromen-2-one | HIV 1 |  | Lunney et al | ZINC13738209 | |
| 784 | 4-hydroxy-3-(3-hydroxypropyl)-2H-chromen-2-one | HIV 1 |  | Lunney et al | ZINC13738210 | |
| 785 | 4-hydroxy-3-(3-oxo-3-phenylpropyl)-2H-chromen-2-one | HIV 1 |  | Lunney et al | ZINC05848927 | |
| 786 | 4-hydroxy-3-[(3Z)-3-(hydroxyimino)-3-phenylpropyl]-2H-chromen-2-one | HIV 1 |  | Lunney et al | ZINC13738211 | |
| 787 | 4-hydroxy-3-[(2-phenoxyethyl)sulfanyl]-2H-chromen-2-one | HIV 1 |  | Lunney et al | ZINC13738212 | |
| 788 | 4-hydroxy-3-(3-phenoxypropyl)-1,2-dihydroquinolin-2-one | HIV 1 |  | Lunney et al | ZINC13738213 | |
| 789 | 4-cyano-N-(3-{1-[4-hydroxy-2-oxo-6-(2-phenylethyl)-6-propyl-5,6-dihydro-2H-pyran-3-yl]propyl}phenyl)benzene-1-sulfonamide | HIV 1 | 10.1021/jm9802158 | Turner et al | ZINC36124704 | |
| 790 | 5-cyano-N-(3-{1-[4-hydroxy-2-oxo-6-(2-phenylethyl)-6-propyl-5,6-dihydro-2H-pyran-3-yl]propyl}phenyl)pyridine-2-sulfonamide | HIV 1 | 10.1021/jm9802158 | Turner et al |  | |
| 791 | 4-cyano-N-(3-{1-[4-hydroxy-2-oxo-6-(2-phenylethyl)-6-propyl-5,6-dihydro-2H-pyran-3-yl]-2,2-dimethylpropyl}phenyl)benzene-1-sulfonamide | HIV 1 | 10.1021/jm9802158 | Turner et al | ZINC70454645 | |
| 792 | 5-cyano-N-(3-{1-[4-hydroxy-2-oxo-6-(2-phenylethyl)-6-propyl-5,6-dihydro-2H-pyran-3-yl]-2,2-dimethylpropyl}phenyl)pyridine-2-sulfonamide | HIV 1 | 10.1021/jm9802158 | Turner et al | ZINC70454647 | |
| 793 | 5-amino-N-(3-{1-[4-hydroxy-2-oxo-6-(2-phenylethyl)-6-propyl-5,6-dihydro-2H-pyran-3-yl]-2,2-dimethylpropyl}phenyl)pyridine-2-sulfonamide | HIV 1 | 10.1021/jm9802158 | Turner et al | ZINC14943268 | |
| 794 | (4S,5S,6S)-4-benzyl-5-hydroxy-1,3-bis({[3-(hydroxymethyl)phenyl]methyl})-6-(2-phenylethyl)-1,3-diazinan-2-one | HIV 1 | 10.1021/jm970081i | De Lucca et al | ZINC14943287 | |
| 795 | (4S,5S,6S)-Tetrahydro-1,3-bis[(3-cyanophenyl)methyl]-5-hydroxy-4-(2-phenylethyl)-6-(phenylmethyl)-2(1H)-pyrimidinone | HIV 1 | 10.1021/jm970081i | De Lucca et al | ZINC14943290 | |
| 796 | (4S,5S,6S)-Tetrahydro-1,3-bis[(3-carbomethoxyphenyl)methyl]-5-hydroxy-4-(2-phenylethyl)-6-(phenylmethyl)-2(1H)-pyrimidinone | HIV 1 | 10.1021/jm970081i | De Lucca et al | ZINC14943291 | |
| 797 | (4S,5S,6S)-Tetrahydro-1,3-bis[(3-benzamido)methyl]-5-hydroxy-4-(2-phenylethyl)-6-(phenylmethyl)-2(1H)-pyrimidinone | HIV 1 | 10.1021/jm970081i | De Lucca et al | ZINC14943292 | |
| 798 | (4S,5S,6S)-Tetrahydro-1,3-bis[(3-benzamide oxime)methyl]-5-hydroxy-4-(2-phenylethyl)-6-(phenylmethyl)-2(1H)-pyrimidinone | HIV 1 | 10.1021/jm970081i | De Lucca et al | ZINC14943293 | |
| 799 | (4R,5S,6S,7R)-4,7-dibenzyl-5,6-dihydroxy-1,3-bis({[4-(hydroxymethyl)phenyl]methyl})-1,3-diazepan-2-one | HIV 1 | 10.1021/jm960728j | Hulten et al | ZINC03833858 | |
| 800 | (4S,5R,6R,7S)-1,3-Dibenzyl-4,7-bis(phenoxymethyl)-5,6-dihydroxy-1,3-diazepan-2-one::(4S,5R,6R,7S)-1,3-dibenzyl-5,6-dihydroxy-4,7-bis(phenoxymethyl)-1,3-diazepan-2-one | HIV 1 | 10.1021/jm960728j | Hulten et al | ZINC03871457 | |
| 801 | (4S,5S,6S,7S)-1,3-Dibenzyl-4,7-bis(phenoxymethyl)-5,6-dihydroxy-1,3-diazepan-2-one::(4S,5S,6S,7S)-1,3-dibenzyl-5,6-dihydroxy-4,7-bis(phenoxymethyl)-1,3-diazepan-2-one | HIV 1 | 10.1021/jm960728j | Hulten et al | ZINC14943328 | |
| 802 | 4-cyano-N-{3-[cyclopropyl(4-hydroxy-7-methoxy-2-oxo-2H-chromen-3-yl)methyl]phenyl}benzene-1-sulfonamide | HIV 1 | 10.1021/jm950888f | Thaisrivongs et al | ZINC13756552 | |
| 803 | 4-cyano-N-(3-{cyclopropyl[6-(1,3-dicyclopropylpropan-2-yl)-4-hydroxy-2-oxo-2H-pyran-3-yl]methyl}phenyl)benzene-1-sulfonamide | HIV 1 | 10.1021/jm950888f | Thaisrivongs et al | ZINC14943344 | |
| 804 | 4-chloro-N-(3-{cyclopropyl[4-hydroxy-2-oxo-6-(1-phenylbutan-2-yl)-2H-pyran-3-yl]methyl}phenyl)benzene-1-sulfonamide | HIV 1 | 10.1021/jm950888f | Thaisrivongs et al | ZINC14943351 | |
| 805 | 4-cyano-N-(3-{cyclopropyl[4-hydroxy-2-oxo-6-(1-phenylbutan-2-yl)-2H-pyran-3-yl]methyl}phenyl)benzene-1-sulfonamide | HIV 1 | 10.1021/jm950888f | Thaisrivongs et al | ZINC14943354 | |
| 806 | benzyl N-[(1S)-1-{[(2S,3R,4R)-4-{[(1S)-1-[(1H-1,3-benzodiazol-2-ylmethyl)carbamoyl]-2-methylpropyl]carbamoyl}-3-hydroxy-4-{[(4-methoxyphenyl)methyl]amino}-1-phenylbutan-2-yl]carbamoyl}-2-methylpropyl]carbamate | HIV 1 | 10.1021/jm9508696 | Lehr et al | ZINC95610694 | |
| 807 | tert-butyl N-[(2S,3R,4R)-4-(benzylamino)-4-(benzylcarbamoyl)-3-hydroxy-1-phenylbutan-2-yl]carbamate | HIV 1 | 10.1021/jm9508696 | Lehr et al | ZINC13744833 | |
| 808 | tert-butyl N-[(2S,3R,4R)-4-({[(1H-1,3-benzodiazol-2-ylmethyl)carbamoyl](phenyl)methyl}carbamoyl)-3-hydroxy-4-{[(4-methoxyphenyl)methyl]amino}-1-phenylbutan-2-yl]carbamate | HIV 1 | 10.1021/jm9508696 | Lehr et al | ZINC26181239 | |
| 809 | tert-butyl N-[(2S,3R,4R)-4-{[(1S)-1-[(1H-1,3-benzodiazol-2-ylmethyl)carbamoyl]-2-methylpropyl]carbamoyl}-3-hydroxy-4-{[(4-methoxyphenyl)methyl]amino}-1-phenylbutan-2-yl]carbamate | HIV 1 | 10.1021/jm9508696 | Lehr et al | ZINC26181295 | |
| 810 | tert-butyl N-[(2S,3R,4R)-4-(benzylcarbamoyl)-3-hydroxy-4-({[4-(2-hydroxyethoxy)phenyl]methyl}amino)-1-phenylbutan-2-yl]carbamate | HIV 1 | 10.1021/jm9508696 | Lehr et al | ZINC14943362 | |
| 811 | tert-butyl N-[(2S,3R,4R)-4-{[(4-{[3-(1H-1,3-benzodiazol-2-yl)propanamido]methyl}phenyl)methyl]amino}-4-(benzylcarbamoyl)-3-hydroxy-1-phenylbutan-2-yl]carbamate | HIV 1 | 10.1021/jm9508696 | Lehr et al | ZINC26172882 | |
| 812 | tert-butyl N-[(2S,3R,4R)-4-{[(4-ethoxyphenyl)methyl]amino}-3-hydroxy-4-{[(1S,2R)-2-hydroxy-2,3-dihydro-1H-inden-1-yl]carbamoyl}-1-phenylbutan-2-yl]carbamate | HIV 1 | 10.1021/jm9508696 | Lehr et al | ZINC14943363 | |
| 813 | tert-butyl N-[(2S,3R,4R)-3-hydroxy-4-{[(1S,2R)-2-hydroxy-2,3-dihydro-1H-inden-1-yl]carbamoyl}-4-({[4-(2-hydroxyethoxy)phenyl]methyl}amino)-1-phenylbutan-2-yl]carbamate | HIV 1 | 10.1021/jm9508696 | Lehr et al | ZINC14943364 | |
| 814 | tert-butyl N-[(2S,3R,4R)-3-hydroxy-4-{[(1S,2R)-2-hydroxy-2,3-dihydro-1H-inden-1-yl]carbamoyl}-4-[({4-[2-(morpholin-4-yl)ethoxy]phenyl}methyl)amino]-1-phenylbutan-2-yl]carbamate | HIV 1 | 10.1021/jm9508696 | Lehr et al | ZINC26177511 | |
| 815 | tert-butyl N-[(2S,3R,4R)-4-({[4-(acetamidomethyl)phenyl]methyl}amino)-3-hydroxy-4-{[(1S,2R)-2-hydroxy-2,3-dihydro-1H-inden-1-yl]carbamoyl}-1-phenylbutan-2-yl]carbamate | HIV 1 | 10.1021/jm9508696 | Lehr et al | ZINC14943365 | |
| 816 | tert-butyl N-[(2S,3R,4R)-3-hydroxy-4-{[(1S,2R)-2-hydroxy-2,3-dihydro-1H-inden-1-yl]carbamoyl}-1-phenyl-4-{[(4-{[(phenylcarbamoyl)amino]methyl}phenyl)methyl]amino}butan-2-yl]carbamate | HIV 1 | 10.1021/jm9508696 | Lehr et al | ZINC26177492 | |
| 817 | tert-butyl N-[(2S,3R,4R)-4-({[4-({[(benzyloxy)carbonyl]amino}methyl)phenyl]methyl}amino)-3-hydroxy-4-{[(1S,2R)-2-hydroxy-2,3-dihydro-1H-inden-1-yl]carbamoyl}-1-phenylbutan-2-yl]carbamate | HIV 1 | 10.1021/jm9508696 | Lehr et al | ZINC26173867 | |
| 818 | benzyl N-(1-{[(2S,3R,4R)-3-hydroxy-4-{[(1S,2R)-2-hydroxy-2,3-dihydro-1H-inden-1-yl]carbamoyl}-4-{[(4-methoxyphenyl)methyl]amino}-1-phenylbutan-2-yl]carbamoyl}-2-methylpropyl)carbamate | HIV 1 | 10.1021/jm9508696 | Lehr et al | ZINC96126884 | |
| 819 | benzyl N-[(1S)-1-{[(2S,3R,4R)-3-hydroxy-4-{[(1S,2R)-2-hydroxy-2,3-dihydro-1H-inden-1-yl]carbamoyl}-4-{[(4-methoxyphenyl)methyl]amino}-1-phenylbutan-2-yl]carbamoyl}-2,2-dimethylpropyl]carbamate | HIV 1 | 10.1021/jm9508696 | Lehr et al | ZINC58513299 | |
| 820 | benzyl N-(1-{[(2S,3R,4R)-3-hydroxy-4-{[(1S,2R)-2-hydroxy-2,3-dihydro-1H-inden-1-yl]carbamoyl}-4-{[(4-methoxyphenyl)methyl]amino}-1-phenylbutan-2-yl]carbamoyl}-2-methylbutyl)carbamate | HIV 1 | 10.1021/jm9508696 | Lehr et al | ZINC96126888 | |
| 821 | 3-{[(benzyloxy)carbonyl]amino}-3-{[(2S,3R,4R)-3-hydroxy-4-{[(1S,2R)-2-hydroxy-2,3-dihydro-1H-inden-1-yl]carbamoyl}-4-{[(4-methoxyphenyl)methyl]amino}-1-phenylbutan-2-yl]carbamoyl}propanoic acid | HIV 1 | 10.1021/jm9508696 | Lehr et al | ZINC26177183 | |
| 822 | benzyl N-(2-carbamoyl-1-{[(2S,3R,4R)-3-hydroxy-4-{[(1S,2R)-2-hydroxy-2,3-dihydro-1H-inden-1-yl]carbamoyl}-4-{[(4-methoxyphenyl)methyl]amino}-1-phenylbutan-2-yl]carbamoyl}ethyl)carbamate | HIV 1 | 10.1021/jm9508696 | Lehr et al | ZINC26177872 | |
| 823 | tert-butyl N-[(1S)-1-{[(2S,3R,4R)-3-hydroxy-4-{[(1S,2R)-2-hydroxy-2,3-dihydro-1H-inden-1-yl]carbamoyl}-4-{[(4-methoxyphenyl)methyl]amino}-1-phenylbutan-2-yl]carbamoyl}-2,2-dimethylpropyl]carbamate | HIV 1 | 10.1021/jm9508696 | Lehr et al | ZINC26177853 | |
| 824 | benzyl N-[(1S)-1-{[(2S,3R,4R)-4-{[(4-ethoxyphenyl)methyl]amino}-3-hydroxy-4-{[(1S,2R)-2-hydroxy-2,3-dihydro-1H-inden-1-yl]carbamoyl}-1-phenylbutan-2-yl]carbamoyl}-2,2-dimethylpropyl]carbamate | HIV 1 | 10.1021/jm9508696 | Lehr et al | ZINC26179126 | |
| 825 | benzyl N-[(1S)-1-{[(2S,3R,4R)-3-hydroxy-4-{[(1S,2R)-2-hydroxy-2,3-dihydro-1H-inden-1-yl]carbamoyl}-4-({[4-(2-hydroxyethoxy)phenyl]methyl}amino)-1-phenylbutan-2-yl]carbamoyl}-2,2-dimethylpropyl]carbamate | HIV 1 | 10.1021/jm9508696 | Lehr et al | ZINC95558011 | |
| 826 | benzyl N-[(1S)-1-{[(2S,3R,4R)-3-hydroxy-4-{[(1S,2R)-2-hydroxy-2,3-dihydro-1H-inden-1-yl]carbamoyl}-4-[({4-[2-(morpholin-4-yl)ethoxy]phenyl}methyl)amino]-1-phenylbutan-2-yl]carbamoyl}-2,2-dimethylpropyl]carbamate | HIV 1 | 10.1021/jm9508696 | Lehr et al | ZINC95540188 | |
| 827 | benzyl N-[(1S)-1-{[(2S,3R,4R)-4-({[4-(acetamidomethyl)phenyl]methyl}amino)-3-hydroxy-4-{[(1S,2R)-2-hydroxy-2,3-dihydro-1H-inden-1-yl]carbamoyl}-1-phenylbutan-2-yl]carbamoyl}-2,2-dimethylpropyl]carbamate | HIV 1 | 10.1021/jm9508696 | Lehr et al | ZINC95558067 | |
| 828 | benzyl N-[(1S)-1-{[(2S,3R,4R)-3-hydroxy-4-{[(1S,2R)-2-hydroxy-2,3-dihydro-1H-inden-1-yl]carbamoyl}-1-phenyl-4-{[(4-{[(phenylcarbamoyl)amino]methyl}phenyl)methyl]amino}butan-2-yl]carbamoyl}-2,2-dimethylpropyl]carbamate | HIV 1 | 10.1021/jm9508696 | Lehr et al | ZINC95543131 | |
| 829 | benzyl N-[(1S)-1-{[(2S,3R,4R)-4-({[4-({[(benzyloxy)carbonyl]amino}methyl)phenyl]methyl}amino)-3-hydroxy-4-{[(1S,2R)-2-hydroxy-2,3-dihydro-1H-inden-1-yl]carbamoyl}-1-phenylbutan-2-yl]carbamoyl}-2,2-dimethylpropyl]carbamate | HIV 1 | 10.1021/jm9508696 | Lehr et al |  | |
| 830 | benzyl N-[(1S)-1-{[(2S,3R,4R)-4-{[(4-{[3-(1H-1,3-benzodiazol-2-yl)propanamido]methyl}phenyl)methyl]amino}-3-hydroxy-4-{[(1S,2R)-2-hydroxy-2,3-dihydro-1H-inden-1-yl]carbamoyl}-1-phenylbutan-2-yl]carbamoyl}-2,2-dimethylpropyl]carbamate | HIV 1 | 10.1021/jm9508696 | Lehr et al |  | |
| 831 | benzyl N-[(1S)-1-{[(2S,3R)-4-[(2S)-2-(tert-butylcarbamoyl)piperidin-1-yl]-3-hydroxy-1-phenylbutan-2-yl]carbamoyl}-2-methylpropyl]carbamate | HIV 1 | 10.1021/jm9606608 | Beaulieu et al | ZINC14943366 | |
| 832 | benzyl N-[(1S)-1-{[(2S,3R)-4-{[2-(tert-butylcarbamoyl)cyclohexyl]formamido}-3-hydroxy-1-phenylbutan-2-yl]carbamoyl}-2-methylpropyl]carbamate | HIV 1 | 10.1021/jm9606608 | Beaulieu et al | ZINC14943370 | |
| 833 | benzyl N-[(1S)-1-{[(2S,3R)-4-[(2R)-N,2-di-tert-butylbutanediamido]-3-hydroxy-1-phenylbutan-2-yl]carbamoyl}-2-methylpropyl]carbamate | HIV 1 | 10.1021/jm9606608 | Beaulieu et al | ZINC14943371 | |
| 834 | benzyl N-[(1S)-1-{[(2S,3R)-4-[(2S)-N,2-di-tert-butylbutanediamido]-3-hydroxy-1-phenylbutan-2-yl]carbamoyl}-2-methylpropyl]carbamate | HIV 1 | 10.1021/jm9606608 | Beaulieu et al | ZINC14943372 | |
| 835 | benzyl N-[(1S)-1-{[(2S,3R)-4-[(2S)-N,2-di-tert-butylbutanediamido]-3-hydroxy-1-phenylbutan-2-yl]carbamoyl}-2-methylpropyl]carbamate | HIV 1 | 10.1021/jm9606608 | Beaulieu et al | ZINC14943373 | |
| 836 | benzyl N-[(1S)-1-{[(2S,3R)-4-[(2R)-N,2-di-tert-butylbutanediamido]-3-hydroxy-1-phenylbutan-2-yl]carbamoyl}-2-methylpropyl]carbamate | HIV 1 | 10.1021/jm9606608 | Beaulieu et al | ZINC14943374 | |
| 837 | benzyl N-[(1S)-1-{[(2S,3R)-4-(N-tert-butylbutanediamido)-3-hydroxy-1-phenylbutan-2-yl]carbamoyl}-2-methylpropyl]carbamate | HIV 1 | 10.1021/jm9606608 | Beaulieu et al | ZINC14943375 | |
| 838 | benzyl N-[(1S)-1-{[(2S,3S)-4-[(2R)-N,2-di-tert-butylbutanediamido]-3-hydroxy-1-phenylbutan-2-yl]carbamoyl}-2-methylpropyl]carbamate | HIV 1 | 10.1021/jm9606608 | Beaulieu et al | ZINC14943376 | |
| 839 | benzyl N-[(2S,3R)-4-[(2R)-N,2-di-tert-butylbutanediamido]-3-hydroxy-1-phenylbutan-2-yl]carbamate | HIV 1 | 10.1021/jm9606608 | Beaulieu et al | ZINC14943377 | |
| 840 | benzyl N-[(2S,3R)-4-[(2R)-2-tert-butyl-N-(propan-2-yl)butanediamido]-3-hydroxy-1-phenylbutan-2-yl]carbamate | HIV 1 | 10.1021/jm9606608 | Beaulieu et al | ZINC13763666 | |
| 841 | benzyl N-[(2S,3R)-4-[(2R)-2-tert-butyl-N-(pentan-3-yl)butanediamido]-3-hydroxy-1-phenylbutan-2-yl]carbamate | HIV 1 | 10.1021/jm9606608 | Beaulieu et al | ZINC14943378 | |
| 842 | benzyl N-[(2S,3R)-4-[(2R)-2-tert-butyl-N-(heptan-4-yl)butanediamido]-3-hydroxy-1-phenylbutan-2-yl]carbamate | HIV 1 | 10.1021/jm9606608 | Beaulieu et al | ZINC14943379 | |
| 843 | benzyl N-[(2S,3R)-4-[(2R)-2-tert-butyl-N-cyclohexylbutanediamido]-3-hydroxy-1-phenylbutan-2-yl]carbamate | HIV 1 | 10.1021/jm9606608 | Beaulieu et al | ZINC14943380 | |
| 844 | benzyl N-[(2S,3R)-4-[(2R)-2-tert-butyl-N-(2,4-dimethylpentan-3-yl)butanediamido]-3-hydroxy-1-phenylbutan-2-yl]carbamate | HIV 1 | 10.1021/jm9606608 | Beaulieu et al | ZINC14943381 | |
| 845 | benzyl N-[(2S,3R)-4-[(2R)-2-tert-butyl-N-phenylbutanediamido]-3-hydroxy-1-phenylbutan-2-yl]carbamate | HIV 1 | 10.1021/jm9606608 | Beaulieu et al | ZINC14943382 | |
| 846 | benzyl N-[(1S)-1-{[(2S,3R)-4-[(2R)-2-tert-butyl-N-(pentan-3-yl)butanediamido]-3-hydroxy-1-phenylbutan-2-yl]carbamoyl}-2-methylpropyl]carbamate | HIV 1 | 10.1021/jm9606608 | Beaulieu et al | ZINC14943383 | |
| 847 | benzyl N-[(1S)-1-{[(2S,3R)-4-[(2R)-2-tert-butyl-N-(pentan-3-yl)butanediamido]-3-hydroxy-1-phenylbutan-2-yl]carbamoyl}-2-carbamoylethyl]carbamate | HIV 1 | 10.1021/jm9606608 | Beaulieu et al | ZINC26269007 | |
| 848 | benzyl N-[(1S,2S)-1-{[(2S,3R)-4-[(2R)-2-tert-butyl-N-(pentan-3-yl)butanediamido]-3-hydroxy-1-phenylbutan-2-yl]carbamoyl}-2-hydroxypropyl]carbamate | HIV 1 | 10.1021/jm9606608 | Beaulieu et al | ZINC14943385 | |
| 849 | 3-chloro-N-{3-[cyclopropyl({4-hydroxy-2-oxo-2H,5H,6H,7H,8H,9H,10H-cycloocta[b]pyran-3-yl})methyl]phenyl}benzene-1-sulfonamide | HIV 1 | 10.1021/jm960441m | Skulnick et al | ZINC13761636 | |
| 850 | 3-bromo-N-{3-[cyclopropyl({4-hydroxy-2-oxo-2H,5H,6H,7H,8H,9H,10H-cycloocta[b]pyran-3-yl})methyl]phenyl}benzene-1-sulfonamide | HIV 1 | 10.1021/jm960441m | Skulnick et al | ZINC14943409 | |
| 851 | 3-({3-[cyclopropyl({4-hydroxy-2-oxo-2H,5H,6H,7H,8H,9H,10H-cycloocta[b]pyran-3-yl})methyl]phenyl}sulfamoyl)benzoic acid | HIV 1 | 10.1021/jm960441m | Skulnick et al | ZINC13761640 | |
| 852 | 3-amino-N-{3-[cyclopropyl({4-hydroxy-2-oxo-2H,5H,6H,7H,8H,9H,10H-cycloocta[b]pyran-3-yl})methyl]phenyl}benzene-1-sulfonamide | HIV 1 | 10.1021/jm960441m | Skulnick et al | ZINC13761642 | |
| 853 | 3-cyano-N-{3-[cyclopropyl({4-hydroxy-2-oxo-2H,5H,6H,7H,8H,9H,10H-cycloocta[b]pyran-3-yl})methyl]phenyl}benzene-1-sulfonamide | HIV 1 | 10.1021/jm960441m | Skulnick et al | ZINC13761644 | |
| 854 | 4-chloro-N-{3-[cyclopropyl({4-hydroxy-2-oxo-2H,5H,6H,7H,8H,9H,10H-cycloocta[b]pyran-3-yl})methyl]phenyl}benzene-1-sulfonamide | HIV 1 | 10.1021/jm960441m | Skulnick et al | ZINC13761656 | |
| 855 | 4-bromo-N-{3-[cyclopropyl({4-hydroxy-2-oxo-2H,5H,6H,7H,8H,9H,10H-cycloocta[b]pyran-3-yl})methyl]phenyl}benzene-1-sulfonamide | HIV 1 | 10.1021/jm960441m | Skulnick et al | ZINC14943415 | |
| 856 | 4-Cyano-N-[3-[cyclopropyl(5,6,7,8,9,10-hexahydro-4-hydroxy-2-oxo-2H-cycloocta[b]pyran-3-yl)methyl]phenyl]-benzenesulfonamide | HIV 1 | 10.1021/jm960441m | Skulnick et al | ZINC03873405 | |
| 857 | 4-({3-[cyclopropyl({4-hydroxy-2-oxo-2H,5H,6H,7H,8H,9H,10H-cycloocta[b]pyran-3-yl})methyl]phenyl}sulfamoyl)benzoic acid | HIV 1 | 10.1021/jm960441m | Skulnick et al | ZINC13761660 | |
| 858 | 4-({3-[cyclopropyl({4-hydroxy-2-oxo-2H,5H,6H,7H,8H,9H,10H-cycloocta[b]pyran-3-yl})methyl]phenyl}sulfamoyl)benzamide | HIV 1 | 10.1021/jm960441m | Skulnick et al | ZINC13761662 | |
| 859 | 4-butoxy-N-{3-[cyclopropyl({4-hydroxy-2-oxo-2H,5H,6H,7H,8H,9H,10H-cycloocta[b]pyran-3-yl})methyl]phenyl}benzene-1-sulfonamide | HIV 1 | 10.1021/jm960441m | Skulnick et al | ZINC14943421 | |
| 860 | 4-amino-N-{3-[cyclopropyl({4-hydroxy-2-oxo-2H,5H,6H,7H,8H,9H,10H-cycloocta[b]pyran-3-yl})methyl]phenyl}benzene-1-sulfonamide | HIV 1 | 10.1021/jm960441m | Skulnick et al | ZINC13761666 | |
| 861 | 4-azido-N-{3-[cyclopropyl({4-hydroxy-2-oxo-2H,5H,6H,7H,8H,9H,10H-cycloocta[b]pyran-3-yl})methyl]phenyl}benzene-1-sulfonamide | HIV 1 | 10.1021/jm960441m | Skulnick et al | ZINC13761670 | |
| 862 | 5-cyano-N-{3-[cyclopropyl({4-hydroxy-2-oxo-2H,5H,6H,7H,8H,9H,10H-cycloocta[b]pyran-3-yl})methyl]phenyl}pyridine-2-sulfonamide | HIV 1 | 10.1021/jm960441m | Skulnick et al | ZINC13761678 | |
| 863 | 4-chloro-N-[3-(1-{4-hydroxy-2-oxo-2H,5H,6H,7H,8H,9H,10H-cycloocta[b]pyran-3-yl}propyl)phenyl]benzene-1-sulfonamide | HIV 1 | 10.1021/jm960441m | Skulnick et al | ZINC13761692 | |
| 864 | 4-chloro-N-[3-(1-{4-hydroxy-2-oxo-2H,5H,6H,7H,8H,9H,10H-cycloocta[b]pyran-3-yl}butyl)phenyl]benzene-1-sulfonamide | HIV 1 | 10.1021/jm960441m | Skulnick et al | ZINC13761694 | |
| 865 | 4-chloro-N-[3-(1-{4-hydroxy-2-oxo-2H,5H,6H,7H,8H,9H,10H-cycloocta[b]pyran-3-yl}-2-methylpropyl)phenyl]benzene-1-sulfonamide | HIV 1 | 10.1021/jm960441m | Skulnick et al | ZINC13761696 | |
| 866 | 4-Cyano-N-[3-[1-(5,6,7,8,9,10-hexahydro-4-hydroxy-2-oxo-2H-cycloocta[b]pyran-3-yl)propyl]phenyl]benzenesulfonamide | HIV 1 | 10.1021/jm960441m | Skulnick et al | ZINC13761698 | |
| 867 | 4-Cyano-N-[3-[1-(5,6,7,8,9,10-hexahydro-4-hydroxy-2-oxo-2H-cycloocta[b]pyran-3-yl)propyl]butyl]benzenesulfonamide | HIV 1 | 10.1021/jm960441m | Skulnick et al | ZINC13761700 | |
| 868 | 4-Cyano-N-[3-[1-(5,6,7,8,9,10-hexahydro-4-hydroxy-2-oxo-2H-cycloocta[b]pyran-3-yl)propyl]pentyl]benzenesulfonamide | HIV 1 | 10.1021/jm960441m | Skulnick et al | ZINC13761702 | |
| 869 | 4-Cyano-N-[3-[1-(5,6,7,8,9,10-hexahydro-4-hydroxy-2-oxo-2H-cycloocta[b]pyran-3-yl)propyl]-2-methylpropyl]-benzenesulfonamide | HIV 1 | 10.1021/jm960441m | Skulnick et al | ZINC13761704 | |
| 870 | 4-Cyano-N-[3-[1-(5,6,7,8,9,10-hexahydro-4-hydroxy-2-oxo-2H-cycloocta[b]pyran-3-yl)propyl]-3-methylbutyl]-benzenesulfonamide | HIV 1 | 10.1021/jm960441m | Skulnick et al | ZINC13761706 | |
| 871 | (4S,5R,6R,7S)-1,3,4,7-tetrabenzyl-5,6-dihydroxy-1,3-diazepan-2-one | HIV 1 | 10.1021/jm980255b | Kaltenbach et al | ZINC14943463 | |
| 872 | (4S,5S,6S,7S)-1,3,4,7-tetrabenzyl-5,6-dihydroxy-1,3-diazepan-2-one | HIV 1 | 10.1021/jm980255b | Kaltenbach et al | ZINC14943465 | |
| 873 | (4R,5S,6S,7S)-1,3,4,7-tetrabenzyl-5,6-dihydroxy-1,3-diazepan-2-one | HIV 1 | 10.1021/jm980255b | Kaltenbach et al | ZINC14943466 | |
| 874 | (4S,5R,6R,7R)-1,3,4,7-tetrabenzyl-5,6-dihydroxy-1,3-diazepan-2-one | HIV 1 | 10.1021/jm980255b | Kaltenbach et al | ZINC14943467 | |
| 875 | (4S,5R,6S,7S)-1,3,4,7-tetrabenzyl-5,6-dihydroxy-1,3-diazepan-2-one | HIV 1 | 10.1021/jm980255b | Kaltenbach et al | ZINC14943469 | |
| 876 | 3-{[(4R,5S,6S,7R)-4,7-dibenzyl-2-(cyanoimino)-3-[(3-cyanophenyl)methyl]-5,6-dihydroxy-1,3-diazepan-1-yl]methyl}benzonitrile | HIV 1 | 10.1021/jm970524i | Jadhav et al | ZINC14943479 | |
| 877 | (4R,5S,6S,7R)-4,7-dibenzyl-5,6-dihydroxy-1,3-diazepan-2-one | HIV 1 | 10.1021/jm970524i | Jadhav et al | ZINC00008108 | |
| 878 | (4R,5S,6S,7R)-4,7-dibenzyl-5,6-dihydroxy-1,3-bis[(3-nitrophenyl)methyl]-1,3-diazepan-2-one | HIV 1 | 10.1021/jm970524i | Jadhav et al | ZINC14943485 | |
| 879 | (4R,5S,6S,7R)-4,7-dibenzyl-5,6-dihydroxy-1,3-bis[(4-nitrophenyl)methyl]-1,3-diazepan-2-one | HIV 1 | 10.1021/jm970524i | Jadhav et al | ZINC14943487 | |
| 880 | 3-{[(4R,5S,6S,7R)-4,7-dibenzyl-3-[(3-cyanophenyl)methyl]-5,6-dihydroxy-2-oxo-1,3-diazepan-1-yl]methyl}benzonitrile | HIV 1 | 10.1021/jm970524i | Jadhav et al | ZINC14943491 | |
| 881 | (4R,5S,6S,7R)-4,7-dibenzyl-5,6-dihydroxy-1,3-bis[(3-hydroxyphenyl)methyl]-1,3-diazepan-2-one | HIV 1 | 10.1021/jm970524i | Jadhav et al | ZINC03932734 | |
| 882 | (4R,5S,6S,7R)-4,7-dibenzyl-5,6-dihydroxy-1,3-bis[(4-hydroxyphenyl)methyl]-1,3-diazepan-2-one | HIV 1 | 10.1021/jm970524i | Jadhav et al | ZINC03932737 | |
| 883 | (4R,5S,6S,7R)-4,7-dibenzyl-5,6-dihydroxy-1,3-bis({[3-(hydroxymethyl)phenyl]methyl})-1,3-diazepan-2-one | HIV 1 | 10.1021/jm970524i | Jadhav et al | ZINC03932740 | |
| 884 | 3-(benzylsulfanyl)-4-hydroxy-6-phenyl-5,6-dihydro-2H-pyran-2-one | HIV 1 | 10.1021/jm970615f | Tait et al | ZINC61959291 | |
| 885 | 4-hydroxy-6-phenyl-3-[(2-phenylethyl)sulfanyl]-5,6-dihydro-2H-pyran-2-one | HIV 1 | 10.1021/jm970615f | Tait et al | ZINC61959292 | |
| 886 | 5,6-Dihydro-4-hydroxy-6-phenyl-3-[(phenylmethyl)thio]-6-propyl-2H-pyran-2-one | HIV 1 | 10.1021/jm970615f | Tait et al | ZINC13766401 | |
| 887 | 4-hydroxy-6-phenyl-3-[(2-phenylethyl)sulfanyl]-6-propyl-5,6-dihydro-2H-pyran-2-one | HIV 1 | 10.1021/jm970615f | Tait et al | ZINC13766466 | |
| 888 | 3-(benzylsulfanyl)-6-butyl-4-hydroxy-6-phenyl-5,6-dihydro-2H-pyran-2-one | HIV 1 | 10.1021/jm970615f | Tait et al | ZINC13766404 | |
| 889 | 6-Butyl-5,6-dihydro-4-hydroxy-6-phenyl-3-[(2-phenylethyl)thio]-2H-pyran-2-one | HIV 1 | 10.1021/jm970615f | Tait et al | ZINC13766470 | |
| 890 | 3-(benzylsulfanyl)-4-hydroxy-6-pentyl-6-phenyl-5,6-dihydro-2H-pyran-2-one | HIV 1 | 10.1021/jm970615f | Tait et al | ZINC17992169 | |
| 891 | 4-hydroxy-6-pentyl-6-phenyl-3-[(2-phenylethyl)sulfanyl]-5,6-dihydro-2H-pyran-2-one | HIV 1 | 10.1021/jm970615f | Tait et al | ZINC13766474 | |
| 892 | 3-(benzylsulfanyl)-6-hexyl-4-hydroxy-6-phenyl-5,6-dihydro-2H-pyran-2-one | HIV 1 | 10.1021/jm970615f | Tait et al | ZINC13766412 | |
| 893 | 5,6-Dihydro-6-hexyl-4-hydroxy-6-phenyl-3-[(2-phenylethyl)thio]-2H-pyran-2-one | HIV 1 | 10.1021/jm970615f | Tait et al | ZINC13766478 | |
| 894 | 3-(benzylsulfanyl)-4-hydroxy-6-(2-methylpropyl)-6-phenyl-5,6-dihydro-2H-pyran-2-one | HIV 1 | 10.1021/jm970615f | Tait et al | ZINC13766416 | |
| 895 | 4-hydroxy-6-(2-methylpropyl)-6-phenyl-3-[(2-phenylethyl)sulfanyl]-5,6-dihydro-2H-pyran-2-one | HIV 1 | 10.1021/jm970615f | Tait et al | ZINC13766482 | |
| 896 | 4-hydroxy-6-(3-methylbutyl)-6-phenyl-3-(phenylsulfanyl)-5,6-dihydro-2H-pyran-2-one | HIV 1 | 10.1021/jm970615f | Tait et al | ZINC13766452 | |
| 897 | 3-(benzylsulfanyl)-4-hydroxy-6-(3-methylbutyl)-6-phenyl-5,6-dihydro-2H-pyran-2-one | HIV 1 | 10.1021/jm970615f | Tait et al | ZINC13766420 | |
| 898 | 4-hydroxy-6-(3-methylbutyl)-6-phenyl-3-[(2-phenylethyl)sulfanyl]-5,6-dihydro-2H-pyran-2-one | HIV 1 | 10.1021/jm970615f | Tait et al | ZINC13766486 | |
| 899 | 3-(benzylsulfanyl)-4-hydroxy-6-(4-methylpentyl)-6-phenyl-5,6-dihydro-2H-pyran-2-one | HIV 1 | 10.1021/jm970615f | Tait et al | ZINC13766424 | |
| 900 | 4-hydroxy-6-(4-methylpentyl)-6-phenyl-3-[(2-phenylethyl)sulfanyl]-5,6-dihydro-2H-pyran-2-one | HIV 1 | 10.1021/jm970615f | Tait et al | ZINC13766490 | |
| 901 | 3-(benzylsulfanyl)-6-(cyclopentylmethyl)-4-hydroxy-6-phenyl-5,6-dihydro-2H-pyran-2-one | HIV 1 | 10.1021/jm970615f | Tait et al | ZINC06580324 | |
| 902 | 6-(cyclopentylmethyl)-4-hydroxy-6-phenyl-3-[(2-phenylethyl)sulfanyl]-5,6-dihydro-2H-pyran-2-one | HIV 1 | 10.1021/jm970615f | Tait et al | ZINC61959293 | |
| 903 | 3-(benzylsulfanyl)-6-(cyclohexylmethyl)-4-hydroxy-6-phenyl-5,6-dihydro-2H-pyran-2-one | HIV 1 | 10.1021/jm970615f | Tait et al | ZINC06580325 | |
| 904 | 4-hydroxy-6,6-diphenyl-3-(phenylsulfanyl)-5,6-dihydro-2H-pyran-2-one | HIV 1 | 10.1021/jm970615f | Tait et al | ZINC13766454 | |
| 905 | 4-hydroxy-6,6-diphenyl-3-[(2-phenylethyl)sulfanyl]-5,6-dihydro-2H-pyran-2-one | HIV 1 | 10.1021/jm970615f | Tait et al | ZINC61959295 | |
| 906 | 4-hydroxy-6-phenyl-6-(2-phenylethyl)-3-(phenylsulfanyl)-5,6-dihydro-2H-pyran-2-one | HIV 1 | 10.1021/jm970615f | Tait et al | ZINC13766458 | |
| 907 | 3-(benzylsulfanyl)-4-hydroxy-6-phenyl-6-(2-phenylethyl)-5,6-dihydro-2H-pyran-2-one | HIV 1 | 10.1021/jm970615f | Tait et al | ZINC18067723 | |
| 908 | 4-hydroxy-6-phenyl-6-(2-phenylethyl)-3-[(2-phenylethyl)sulfanyl]-5,6-dihydro-2H-pyran-2-one | HIV 1 | 10.1021/jm970615f | Tait et al | ZINC13766500 | |
| 909 | 4-{4-hydroxy-6-oxo-2-phenyl-5-[(2-phenylethyl)sulfanyl]-3,6-dihydro-2H-pyran-2-yl}butanoic acid | HIV 1 | 10.1021/jm970615f | Tait et al | ZINC27209952 | |
| 910 | 5-(3,6-Dihydro-4-hydroxy-6-oxo-2-phenyl-5-[(2-phenylethyl)thio]-2H-pyran-2-yl)pentanoic Acid | HIV 1 | 10.1021/jm970615f | Tait et al | ZINC27210020 | |
| 911 | 4-(3,6-Dihydro-4-hydroxy-6-oxo-2-phenyl-5-[(phenylethyl)thio]-2H-pyran-2-yl)butyramid | HIV 1 | 10.1021/jm970615f | Tait et al | ZINC13766519 | |
| 912 | 5-(3,6-Dihydro-4-hydroxy-6-oxo-2-phenyl-5-[(2-phenylethyl)thio]-2H-pyran-2-yl)pentanoic Acid Amide | HIV 1 | 10.1021/jm970615f | Tait et al | ZINC13766523 | |
| 913 | 4-hydroxy-6-phenyl-3-[(2-phenylethyl)sulfanyl]-6-(pyridin-4-yl)-5,6-dihydro-2H-pyran-2-one | HIV 1 | 10.1021/jm970615f | Tait et al | ZINC13766527 | |
| 914 | 5,6-Dihydro-4-hydroxy-6-[(methylphenylamino)methyl]-6-phenyl-3-[(2-phenylethyl)thio]-2H-pyran-2-one | HIV 1 | 10.1021/jm970615f | Tait et al | ZINC13766531 | |
| 915 | 3-(benzylsulfanyl)-4-hydroxy-6-(phenoxymethyl)-6-phenyl-5,6-dihydro-2H-pyran-2-one | HIV 1 | 10.1021/jm970615f | Tait et al | ZINC06580327 | |
| 916 | 4-hydroxy-3-[(2-methylphenyl)sulfanyl]-6,6-diphenyl-5,6-dihydro-2H-pyran-2-one | HIV 1 | 10.1021/jm970615f | Tait et al | ZINC13766542 | |
| 917 | 4-hydroxy-3-[(2-methylphenyl)sulfanyl]-6-phenyl-6-(2-phenylethyl)-5,6-dihydro-2H-pyran-2-one | HIV 1 | 10.1021/jm970615f | Tait et al | ZINC06523285 | |
| 918 | 4-hydroxy-6,6-diphenyl-3-{[2-(propan-2-yl)phenyl]sulfanyl}-5,6-dihydro-2H-pyran-2-one | HIV 1 | 10.1021/jm970615f | Tait et al | ZINC13766544 | |
| 919 | 4-hydroxy-6-phenyl-6-(2-phenylethyl)-3-{[2-(propan-2-yl)phenyl]sulfanyl}-5,6-dihydro-2H-pyran-2-one | HIV 1 | 10.1021/jm970615f | Tait et al | ZINC27207561 | |
| 920 | 3-[(2-sec-Butylphenyl)sulfanyl]-5,6-dihydro-4-hydroxy-6,6-diphenyl-2H-pyran-2-one | HIV 1 | 10.1021/jm970615f | Tait et al | ZINC27209521 | |
| 921 | 3-[(2-sec-Butylphenyl)sulfanyl]-5,6-dihydro-4-hydroxy-6-phenyl-6-(2-phenylethyl)-2H-pyran-2-one | HIV 1 | 10.1021/jm970615f | Tait et al | ZINC27203725 | |
| 922 | 3-[(2-Cyclopentylphenyl)sulfanyl]-5,6-dihydro-4-hydroxy-6,6-diphenyl-2H-pyran-2-one | HIV 1 | 10.1021/jm970615f | Tait et al | ZINC13766550 | |
| 923 | 3-[(2-Cyclohexylphenyl)sulfanyl]-5,6-dihydro-4-hydroxy-6,6-diphenyl-2H-pyran-2-one | HIV 1 | 10.1021/jm970615f | Tait et al | ZINC13766552 | |
| 924 | 3-[(2-Cyclohexylphenyl)sulfanyl]-5,6-dihydro-4-hydroxy-6-phenyl-6-(2-phenylethyl)-2H-pyran-2-one | HIV 1 | 10.1021/jm970615f | Tait et al | ZINC13766579 | |
| 925 | 3-[(2-tert-Butylphenyl)sulfanyl]-5,6-dihydro-4-hydroxy-6,6-diphenyl-2H-pyran-2-one | HIV 1 | 10.1021/jm970615f | Tait et al | ZINC27207703 | |
| 926 | 3-[(2-tert-Butylphenyl)sulfanyl]-5,6-dihydro-4-hydroxy-6-phenyl-6-(2-phenylethyl)-2H-pyran-2-one | HIV 1 | 10.1021/jm970615f | Tait et al | ZINC27210172 | |
| 927 | 4-hydroxy-3-{[5-methyl-2-(propan-2-yl)phenyl]sulfanyl}-6,6-diphenyl-5,6-dihydro-2H-pyran-2-one | HIV 1 | 10.1021/jm970615f | Tait et al | ZINC27201559 | |
| 928 | 4-hydroxy-3-{[5-methyl-2-(propan-2-yl)phenyl]sulfanyl}-6-phenyl-6-(2-phenylethyl)-5,6-dihydro-2H-pyran-2-one | HIV 1 | 10.1021/jm970615f | Tait et al | ZINC27201553 | |
| 929 | 3-{[2,5-bis(propan-2-yl)phenyl]sulfanyl}-4-hydroxy-6,6-diphenyl-5,6-dihydro-2H-pyran-2-one | HIV 1 | 10.1021/jm970615f | Tait et al | ZINC27211352 | |
| 930 | 3-{[2,5-bis(propan-2-yl)phenyl]sulfanyl}-4-hydroxy-6-phenyl-6-(2-phenylethyl)-5,6-dihydro-2H-pyran-2-one | HIV 1 | 10.1021/jm970615f | Tait et al | ZINC27202012 | |
| 931 | 3-[(2-tert-Butyl-5-methylphenyl)sulfanyl]-5,6-dihydro-4-hydroxy-6,6-diphenyl-2H-pyran-2-one | HIV 1 | 10.1021/jm970615f | Tait et al | ZINC27204272 | |
| 932 | 3-[(2-tert-butyl-5-methylphenyl)sulfanyl]-4-hydroxy-6-phenyl-6-(2-phenylethyl)-5,6-dihydro-2H-pyran-2-one | HIV 1 | 10.1021/jm970615f | Tait et al | ZINC26150451 | |
| 933 | 3-[(2-tert-Butyl-5-isopropylphenyl)sulfanyl]-5,6-dihydro-4-hydroxy-6-phenyl-6-(2-phenylethyl)-2H-pyran-2-one | HIV 1 | 10.1021/jm970615f | Tait et al | ZINC27200716 | |
| 934 | 4-Hydroxy-3-[1-[3-[[[[(tert-butyloxycarbonyl)amino]methyl]carbonyl]amino]phenyl]propyl]coumarin | HIV 1 |  | Thaisrivongs et al | ZINC03875238 | |
| 935 | 4-Hydroxy-3-[1-[3-[[3-[(tert-butyloxycarbonyl)amino]-1-oxopropyl]amino]phenyl]propyl]coumarin | HIV 1 |  | Thaisrivongs et al | ZINC13743227 | |
| 936 | 4-Hydroxy-3-[1-3-[[4-[(tert-butyloxycarbonyl)amino]-1-oxobutyl]amino]phenyl]propyl]coumarin | HIV 1 |  | Thaisrivongs et al | ZINC13743229 | |
| 937 | 4-Hydroxy-3-[1-[3-[[3-(1H-indol-1-yl)-1-oxopropyl]amino]phenyl]propyl]coumarin | HIV 1 |  | Thaisrivongs et al | ZINC13743234 | |
| 938 | 4-hydroxy-7-methoxy-3-(1-phenylpropyl)-2H-chromen-2-one | HIV 1 |  | Thaisrivongs et al | ZINC13671651 | |
| 939 | 4-Hydroxy-3-[1-3-[[3-[(tert-butyloxycarbonyl)amino]-1-oxopropyl]amino]phenyl]propyl]-7-methoxycoumarin | HIV 1 |  | Thaisrivongs et al | ZINC13743236 | |
| 940 | 3-[cyclopropyl(phenyl)methyl]-4-hydroxy-2H-chromen-2-one | HIV 1 |  | Thaisrivongs et al | ZINC13743238 | |
| 941 | 3-[cyclopropyl(phenyl)methyl]-4-hydroxy-7-methoxy-2H-chromen-2-one | HIV 1 |  | Thaisrivongs et al | ZINC13743242 | |
| 942 | 3-[1-[3-[[[(2-Benzimidazolyl)methyl]amino]carbonyl]phenyl]propyl]-4-hydroxycoumarin | HIV 1 |  | Thaisrivongs et al | ZINC13743247 | |
| 943 | 3-[(2-tert-butyl-5-methylphenyl)sulfanyl]-4-hydroxy-6-[2-(4-hydroxyphenyl)ethyl]-6-phenyl-5,6-dihydro-2H-pyran-2-one | HIV 1 |  | Prasad et al | ZINC27199115 | |
| 944 | 3-{[2-tert-butyl-4-(hydroxymethyl)-5-methylphenyl]sulfanyl}-4-hydroxy-6-[2-(4-hydroxyphenyl)ethyl]-6-methyl-5,6-dihydro-2H-pyran-2-one | HIV 1 |  | Prasad et al | ZINC27195075 | |
| 945 | 3-{[2-tert-butyl-4-(hydroxymethyl)-5-methylphenyl]sulfanyl}-4-hydroxy-6-[2-(4-hydroxyphenyl)ethyl]-6-(propan-2-yl)-5,6-dihydro-2H-pyran-2-one | HIV 1 |  | Prasad et al |  | |
| 946 | 3-{[2-tert-butyl-4-(hydroxymethyl)-5-methylphenyl]sulfanyl}-6-cyclohexyl-4-hydroxy-6-[2-(4-hydroxyphenyl)ethyl]-5,6-dihydro-2H-pyran-2-one | HIV 1 |  | Prasad et al | ZINC61959326 | |
| 947 | 3-[(2-tert-butyl-4-hydroxy-5-methylphenyl)sulfanyl]-4-hydroxy-6-[2-(4-hydroxyphenyl)ethyl]-6-(propan-2-yl)-5,6-dihydro-2H-pyran-2-one | HIV 1 |  | Prasad et al | ZINC32037013 | |
| 948 | 3-{[2-tert-butyl-4-(hydroxymethyl)-5-methylphenyl]sulfanyl}-4-hydroxy-6-[2-(4-hydroxyphenyl)ethyl]-6-(propan-2-yl)-5,6-dihydro-2H-pyran-2-one | HIV 1 |  | Prasad et al |  | |
| 949 | 3-{[2-tert-butyl-4-(hydroxymethyl)-5-methylphenyl]sulfanyl}-4-hydroxy-6-phenyl-6-(2-phenylethyl)-5,6-dihydro-2H-pyran-2-one | HIV 1 |  | Prasad et al | ZINC27197155 | |
| 950 | 3-[(2-tert-butyl-4-hydroxy-5-methylphenyl)sulfanyl]-4-hydroxy-6-(2-phenylethyl)-6-(propan-2-yl)-5,6-dihydro-2H-pyran-2-one | HIV 1 |  | Prasad et al | ZINC61959299 | |
| 951 | 3-{[2-tert-butyl-4-(2-hydroxyethoxy)-5-methylphenyl]sulfanyl}-4-hydroxy-6-phenyl-6-(2-phenylethyl)-5,6-dihydro-2H-pyran-2-one | HIV 1 |  | Prasad et al | ZINC27196083 | |
| 952 | 4-hydroxy-6-phenyl-3-[(phenylsulfanyl)methyl]-2H-pyran-2-one::4Hydroxy-6-phenyl-3-[(phenylthio)methyl]-2H-pyran-2-one | HIV 1 |  | Prasad et al | ZINC13740561 | |
| 953 | 3-[(2-tert-butyl-5-methylphenyl)sulfanyl]-4-hydroxy-6-phenyl-6-(2-phenylethyl)-5,6-dihydro-2H-pyran-2-one | HIV 1 | 10.1021/jm970522y | Hagen et al | ZINC26150451 | |
| 954 | 3-[(2-tert-butyl-4-hydroxy-5-methylphenyl)sulfanyl]-4-hydroxy-6-phenyl-6-(2-phenylethyl)-5,6-dihydro-2H-pyran-2-one | HIV 1 | 10.1021/jm970522y | Hagen et al | ZINC27206694 | |
| 955 | 3-{[2-tert-butyl-4-(2-hydroxyethoxy)-5-methylphenyl]sulfanyl}-4-hydroxy-6-phenyl-6-(2-phenylethyl)-5,6-dihydro-2H-pyran-2-one | HIV 1 | 10.1021/jm970522y | Hagen et al | ZINC27196083 | |
| 956 | 3-{[2-tert-butyl-4-(hydroxymethyl)-5-methylphenyl]sulfanyl}-4-hydroxy-6-phenyl-6-(2-phenylethyl)-5,6-dihydro-2H-pyran-2-one | HIV 1 | 10.1021/jm970522y | Hagen et al | ZINC27197155 | |
| 957 | 3-[(2-tert-butyl-4-methoxy-5-methylphenyl)sulfanyl]-4-hydroxy-6-phenyl-6-(2-phenylethyl)-5,6-dihydro-2H-pyran-2-one | HIV 1 | 10.1021/jm970522y | Hagen et al | ZINC27200478 | |
| 958 | 3-[(2-tert-butyl-5-methylphenyl)sulfanyl]-4-hydroxy-6-[2-(4-hydroxyphenyl)ethyl]-6-phenyl-5,6-dihydro-2H-pyran-2-one | HIV 1 | 10.1021/jm970522y | Hagen et al | ZINC27199115 | |
| 959 | 3-[(2-tert-butyl-5-methylphenyl)sulfanyl]-4-hydroxy-6-(4-hydroxyphenyl)-6-(2-phenylethyl)-5,6-dihydro-2H-pyran-2-one | HIV 1 | 10.1021/jm970522y | Hagen et al | ZINC27206785 | |
| 960 | 3-[(2-tert-butyl-5-methylphenyl)sulfanyl]-4-hydroxy-6-[4-(2-hydroxyethoxy)phenyl]-6-(2-phenylethyl)-5,6-dihydro-2H-pyran-2-one | HIV 1 | 10.1021/jm970522y | Hagen et al | ZINC27193227 | |
| 961 | 3-{[2-tert-butyl-4-(hydroxymethyl)-5-methylphenyl]sulfanyl}-4-hydroxy-6-[2-(3-hydroxyphenyl)ethyl]-6-phenyl-5,6-dihydro-2H-pyran-2-one | HIV 1 | 10.1021/jm970522y | Hagen et al | ZINC27196770 | |
| 962 | 3-{[2-tert-butyl-4-(hydroxymethyl)-5-methylphenyl]sulfanyl}-4-hydroxy-6-[4-(2-hydroxyethoxy)phenyl]-6-(2-phenylethyl)-5,6-dihydro-2H-pyran-2-one | HIV 1 | 10.1021/jm970522y | Hagen et al | ZINC27201183 | |
| 963 | 3-{[2-tert-butyl-4-(2-hydroxyethoxy)-5-methylphenyl]sulfanyl}-4-hydroxy-6-[4-(2-hydroxyethoxy)phenyl]-6-(2-phenylethyl)-5,6-dihydro-2H-pyran-2-one | HIV 1 | 10.1021/jm970522y | Hagen et al | ZINC27198625 | |
| 964 | 3-[(2-tert-butyl-4-hydroxy-5-methylphenyl)sulfanyl]-4-hydroxy-6-[4-(2-hydroxyethoxy)phenyl]-6-(2-phenylethyl)-5,6-dihydro-2H-pyran-2-one | HIV 1 | 10.1021/jm970522y | Hagen et al | ZINC27198615 | |
| 965 | 3-{[2-tert-butyl-4-(methoxymethyl)-5-methylphenyl]sulfanyl}-4-hydroxy-6-[4-(2-hydroxyethoxy)phenyl]-6-(2-phenylethyl)-5,6-dihydro-2H-pyran-2-one | HIV 1 | 10.1021/jm970522y | Hagen et al | ZINC27199504 | |
| 966 | 3-{[2-tert-butyl-4-(hydroxymethyl)-5-methylphenyl]sulfanyl}-4-hydroxy-6-(4-hydroxyphenyl)-6-[2-(4-hydroxyphenyl)ethyl]-5,6-dihydro-2H-pyran-2-one | HIV 1 | 10.1021/jm970522y | Hagen et al | ZINC27199492 | |
| 967 | 3-{[2-tert-butyl-4-(hydroxymethyl)-5-methylphenyl]sulfanyl}-4-hydroxy-6-[2-(4-hydroxyphenyl)ethyl]-6-(4-methoxyphenyl)-5,6-dihydro-2H-pyran-2-one::6-Phenyl-6-phenethyldihydropyrone 13t | HIV 1 | 10.1021/jm970522y | Hagen et al | ZINC27199481 | |
| 968 | 3-{[2-tert-butyl-4-(hydroxymethyl)-5-methylphenyl]sulfanyl}-6-cyclohexyl-4-hydroxy-6-[2-(4-hydroxyphenyl)ethyl]-5,6-dihydro-2H-pyran-2-one | HIV 1 | 10.1021/jm970522y | Hagen et al | ZINC61959326 | |
| 969 | 3-{[2-tert-butyl-4-(hydroxymethyl)-5-methylphenyl]sulfanyl}-4-hydroxy-6-[2-(4-hydroxyphenyl)ethyl]-6-(propan-2-yl)-5,6-dihydro-2H-pyran-2-one | HIV 1 | 10.1021/jm970522y | Hagen et al |  | |
| 970 | 3-{[2-tert-butyl-4-(hydroxymethyl)-5-methylphenyl]sulfanyl}-4-hydroxy-6-[2-(4-hydroxyphenyl)ethyl]-6-methyl-5,6-dihydro-2H-pyran-2-one | HIV 1 | 10.1021/jm970522y | Hagen et al | ZINC27195075 | |
| 971 | 5-tert-butyl-4-({4-hydroxy-6-[2-(4-hydroxyphenyl)ethyl]-6-methyl-2-oxo-5,6-dihydro-2H-pyran-3-yl}sulfanyl)-2-methylphenyl N-ethylsulfamate | HIV 1 |  | Vara et al | ZINC26284697 | |
| 972 | 5-tert-butyl-4-({4-hydroxy-6-[2-(4-hydroxyphenyl)ethyl]-6-methyl-2-oxo-5,6-dihydro-2H-pyran-3-yl}sulfanyl)-2-methylphenyl N-propylsulfamate | HIV 1 |  | Vara et al | ZINC26289249 | |
| 973 | 5-tert-butyl-4-({4-hydroxy-6-[2-(4-hydroxyphenyl)ethyl]-6-methyl-2-oxo-5,6-dihydro-2H-pyran-3-yl}sulfanyl)-2-methylphenyl N,N-dimethylsulfamate | HIV 1 |  | Vara et al | ZINC26293539 | |
| 974 | 5-tert-butyl-4-({4-hydroxy-6-[2-(4-hydroxyphenyl)ethyl]-6-methyl-2-oxo-5,6-dihydro-2H-pyran-3-yl}sulfanyl)-2-methylphenyl 4-methylpiperazine-1-sulfonate | HIV 1 |  | Vara et al | ZINC55843139 | |
| 975 | 5-tert-butyl-4-({4-hydroxy-6-[2-(4-hydroxyphenyl)ethyl]-2-oxo-6-(propan-2-yl)-5,6-dihydro-2H-pyran-3-yl}sulfanyl)-2-methylphenyl N-methylsulfamate | HIV 1 |  | Vara et al | ZINC26289239 | |
| 976 | 5-tert-butyl-4-({4-hydroxy-6-[2-(4-hydroxyphenyl)ethyl]-2-oxo-6-(propan-2-yl)-5,6-dihydro-2H-pyran-3-yl}sulfanyl)-2-methylphenyl N-ethylsulfamate | HIV 1 |  | Vara et al | ZINC26285117 | |
| 977 | 5-tert-butyl-4-({4-hydroxy-6-[2-(4-hydroxyphenyl)ethyl]-2-oxo-6-(propan-2-yl)-5,6-dihydro-2H-pyran-3-yl}sulfanyl)-2-methylphenyl N-propylsulfamate | HIV 1 |  | Vara et al | ZINC26290530 | |
| 978 | 5-tert-butyl-4-({4-hydroxy-6-[2-(4-hydroxyphenyl)ethyl]-2-oxo-6-(propan-2-yl)-5,6-dihydro-2H-pyran-3-yl}sulfanyl)-2-methylphenyl N,N-dimethylsulfamate | HIV 1 |  | Vara et al | ZINC26376513 | |
| 979 | 5-tert-butyl-4-({4-hydroxy-6-[2-(4-hydroxyphenyl)ethyl]-2-oxo-6-(propan-2-yl)-5,6-dihydro-2H-pyran-3-yl}sulfanyl)-2-methylphenyl 4-methylpiperazine-1-sulfonate | HIV 1 |  | Vara et al | ZINC55843141 | |
| 980 | 5-tert-butyl-4-({4-hydroxy-6-[2-(4-hydroxyphenyl)ethyl]-2-oxo-6-(propan-2-yl)-5,6-dihydro-2H-pyran-3-yl}sulfanyl)-2-methylphenyl morpholine-4-sulfonate | HIV 1 |  | Vara et al | ZINC26290517 | |
| 981 | 5-tert-butyl-4-({6-cyclohexyl-4-hydroxy-6-[2-(4-hydroxyphenyl)ethyl]-2-oxo-5,6-dihydro-2H-pyran-3-yl}sulfanyl)-2-methylphenyl N-ethylsulfamate | HIV 1 |  | Vara et al | ZINC70454664 | |
| 982 | 5-tert-butyl-4-({6-cyclohexyl-4-hydroxy-6-[2-(4-hydroxyphenyl)ethyl]-2-oxo-5,6-dihydro-2H-pyran-3-yl}sulfanyl)-2-methylphenyl N,N-dimethylsulfamate | HIV 1 |  | Vara et al | ZINC70454665 | |
| 983 | 5-tert-butyl-4-({6-cyclohexyl-4-hydroxy-6-[2-(4-hydroxyphenyl)ethyl]-2-oxo-5,6-dihydro-2H-pyran-3-yl}sulfanyl)-2-methylphenyl 4-methylpiperazine-1-sulfonate | HIV 1 |  | Vara et al | ZINC96126915 | |
| 984 | 5-tert-butyl-4-({4-hydroxy-6-[2-(4-hydroxyphenyl)ethyl]-2-oxo-6-phenyl-5,6-dihydro-2H-pyran-3-yl}sulfanyl)-2-methylphenyl N-ethylsulfamate | HIV 1 |  | Vara et al | ZINC70454668 | |
| 985 | 4-({6-[2-(4-aminophenyl)ethyl]-4-hydroxy-2-oxo-6-(propan-2-yl)-5,6-dihydro-2H-pyran-3-yl}sulfanyl)-5-tert-butyl-2-methylphenyl N-ethylsulfamate | HIV 1 |  | Vara et al | ZINC70454661 | |
| 986 | 4-({6-[2-(4-aminophenyl)ethyl]-4-hydroxy-2-oxo-6-(propan-2-yl)-5,6-dihydro-2H-pyran-3-yl}sulfanyl)-5-tert-butyl-2-methylphenyl N,N-dimethylsulfamate | HIV 1 |  | Vara et al | ZINC70454660 | |
| 987 | 4-({6-[2-(4-aminophenyl)ethyl]-4-hydroxy-2-oxo-6-(propan-2-yl)-5,6-dihydro-2H-pyran-3-yl}sulfanyl)-5-tert-butyl-2-methylphenyl 4-methylpiperazine-1-sulfonate | HIV 1 |  | Vara et al | ZINC26375486 | |
| 988 | 5-tert-butyl-4-{[4-hydroxy-2-oxo-6-(2-phenylethyl)-6-(propan-2-yl)-5,6-dihydro-2H-pyran-3-yl]sulfanyl}-2-methylphenyl N-ethylsulfamate | HIV 1 |  | Vara et al | ZINC49799685 | |
| 989 | 5-tert-butyl-4-{[4-hydroxy-2-oxo-6-(2-phenylethyl)-6-(propan-2-yl)-5,6-dihydro-2H-pyran-3-yl]sulfanyl}-2-methylphenyl 4-methylpiperazine-1-sulfonate | HIV 1 |  | Vara et al | ZINC26282287 | |
| 990 | 4-hydroxy-3-(3-phenoxypropyl)-2H-chromen-2-one | HIV 1 |  | Tummino et al | ZINC13738186 | |
| 991 | 3-benzyl-4-hydroxy-2H-chromen-2-one::4-hydroxy-coumarin | HIV 1 |  | Tummino et al | ZINC06580932 | |
| 992 | 4-hydroxy-6-phenyl-3-(phenylsulfanyl)-2H-pyran-2-one | HIV 1 |  | Tummino et al | ZINC13766595 | |
| 993 | 3-(benzylsulfanyl)-4-hydroxy-6-phenyl-2H-pyran-2-one | HIV 1 |  | Tummino et al | ZINC13740595 | |
| 994 | 4-hydroxy-6-phenyl-3-[(2-phenylethyl)sulfanyl]-2H-pyran-2-one | HIV 1 |  | Tummino et al | ZINC14943743 | |
| 995 | 4-hydroxy-6-(4-hydroxyphenyl)-3-[(2-phenylethyl)sulfanyl]-2H-pyran-2-one | HIV 1 |  | Tummino et al | ZINC14943745 | |
| 996 | 4-hydroxy-6-phenyl-3-{[2-(propan-2-yl)phenyl]sulfanyl}-2H-pyran-2-one | HIV 1 |  | Tummino et al | ZINC14943747 | |
| 997 | 4-hydroxy-3-[(2-methylphenyl)sulfanyl]-6-phenyl-2H-pyran-2-one | HIV 1 |  | Tummino et al | ZINC14943749 | |
| 998 | 4-hydroxy-6-phenyl-3-{[2-(propan-2-yl)phenyl]sulfanyl}-2H-pyran-2-one | HIV 1 |  | Tummino et al | ZINC14943747 | |
| 999 | 3-[(2-tert-butylphenyl)sulfanyl]-4-hydroxy-6-phenyl-2H-pyran-2-one | HIV 1 |  | Tummino et al | ZINC14943751 | |
| 1000 | 3-[(2-tert-butylphenyl)sulfanyl]-4-hydroxy-6-(3-methylphenyl)-2H-pyran-2-one | HIV 1 |  | Tummino et al | ZINC06523125 | |

Supplementary table 2. Molecular docking result of reference inhibitor and complete dataset on COVID 19 main protease receptor (PDB ID: 6XBH).

| **Index No** | **Binding Energy**  **(∆G = Kcal/mol)** | **Nflex** | **Hbond** | **Hphob** | **Vwlnt** | **Eintl** | **Dsolv** | **SolEl** | **mfScore** |
| --- | --- | --- | --- | --- | --- | --- | --- | --- | --- |
| REF-IN | -23.5585 | 14 | -3.61026 | -6.19713 | -41.2065 | 14.5944 | 17.7061 | 14.0219 | -41.8047 |
| 1 | -32.5176 | 10 | -7.48419 | -6.76023 | -44.7861 | 19.8861 | 24.8405 | 16.8219 | -109.775 |
| 2 | -22.4068 | 10 | -3.59793 | -7.59194 | -36.9381 | 12.142 | 17.8617 | 14.1279 | -104.028 |
| 3 | -29.8952 | 15 | -7.54899 | -7.72411 | -40.7005 | 15.4554 | 21.5443 | 15.032 | -93.0705 |
| 4 | -19.9719 | 14 | -7.02353 | -8.13659 | -33.5204 | 15.8898 | 19.8686 | 20.4062 | -82.3088 |
| 5 | -19.6425 | 11 | -2.46053 | -8.2077 | -34.992 | 10.7896 | 14.8453 | 14.1513 | -109.393 |
| 6 | -16.0259 | 10 | -2.40152 | -8.27252 | -33.8735 | 15.8878 | 17.284 | 15.9137 | -110.84 |
| 7 | -21.9804 | 18 | -6.10884 | -7.8563 | -40.0674 | 14.6809 | 22.0873 | 17.3343 | -106.138 |
| 8 | -21.0871 | 12 | -2.50612 | -8.49643 | -36.7783 | 10.3029 | 14.5539 | 14.5589 | -109.388 |
| 9 | -22.7886 | 10 | -2.46878 | -8.04881 | -38.0115 | 17.7051 | 14.9071 | 14.6686 | -111.282 |
| 10 | -23.4621 | 11 | -2.49598 | -8.4163 | -38.6658 | 12.9019 | 14.942 | 14.1682 | -107.646 |
| 11 | -16.0242 | 10 | -4.12622 | -6.65053 | -32.4274 | 10.6383 | 19.6929 | 15.9526 | -67.8091 |
| 12 | -23.6801 | 20 | -9.09737 | -7.44121 | -36.4086 | 19.4158 | 25.3571 | 14.7161 | -88.2901 |
| 13 | -23.6992 | 10 | -2.48124 | -8.21197 | -38.6027 | 12.9636 | 14.6713 | 14.6661 | -108.465 |
| 14 | -20.2547 | 20 | -6.64089 | -8.88205 | -41.1892 | 20.8321 | 27.108 | 17.2059 | -73.5981 |
| 15 | -19.2093 | 18 | -8.36363 | -7.98206 | -34.6936 | 19.1691 | 24.3306 | 19.6126 | -97.0391 |
| 16 | -22.6675 | 19 | -8.75169 | -7.98251 | -37.813 | 21.4785 | 26.8922 | 16.7673 | -87.3608 |
| 17 | -22.1226 | 10 | -2.53201 | -7.88312 | -38.0771 | 14.2433 | 16.2115 | 14.369 | -111.109 |
| 18 | -16.205 | 18 | -2.90619 | -9.04054 | -39.7758 | 14.6597 | 21.0209 | 15.7801 | -102.454 |
| 19 | -22.7518 | 10 | -2.49196 | -7.90566 | -37.7248 | 10.2475 | 15.0283 | 14.0958 | -114.134 |
| 20 | -27.3172 | 18 | -6.23706 | -8.10916 | -41.7245 | 16.6723 | 20.8406 | 13.9239 | -101.887 |
| 21 | -16.7495 | 10 | -2.45291 | -7.58929 | -34.0289 | 12.5504 | 16.1158 | 15.8836 | -105.205 |
| 22 | -23.4355 | 22 | -7.97319 | -8.05745 | -41.9648 | 12.9899 | 28.9198 | 13.9548 | -92.3258 |
| 23 | -14.9279 | 10 | -2.08609 | -8.37049 | -33.9014 | 13.4882 | 17.1532 | 16.6586 | -97.874 |
| 24 | -24.4858 | 11 | -2.30199 | -7.32915 | -38.3759 | 13.3299 | 15.2312 | 10.002 | -79.5605 |
| 25 | -17.2046 | 14 | -2.57567 | -8.98254 | -35.8061 | 11.2239 | 17.2616 | 14.8333 | -102.775 |
| 26 | -22.1184 | 10 | -2.45605 | -8.24214 | -38.0584 | 11.0348 | 14.9074 | 15.9022 | -102.751 |
| 27 | -19.4549 | 11 | -2.49237 | -8.33413 | -34.4288 | 12.7549 | 15.0526 | 13.5969 | -98.8906 |
| 28 | -24.0488 | 10 | -2.35101 | -7.12242 | -37.6149 | 12.6094 | 14.3494 | 11.4 | -74.9669 |
| 29 | -18.7293 | 16 | -2.58987 | -8.4776 | -40.1813 | 14.2032 | 19.2402 | 14.6276 | -91.2295 |
| 30 | -20.6151 | 11 | -2.42426 | -8.1898 | -39.0012 | 12.4532 | 17.262 | 15.8487 | -113.843 |
| 31 | -23.5124 | 12 | -1.03466 | -8.22251 | -45.2745 | 9.28383 | 15.7059 | 16.5295 | -106.535 |
| 32 | -30.072 | 14 | -8.23324 | -8.79169 | -46.07 | 20.7487 | 26.8127 | 21.5287 | -46.049 |
| 33 | -17.1248 | 14 | -2.55197 | -8.99035 | -36.3785 | 11.4482 | 17.029 | 16.0031 | -108.171 |
| 34 | -24.4697 | 18 | -7.12729 | -7.6893 | -41.44 | 14.4825 | 23.6733 | 17.5744 | -104.512 |
| 35 | -21.7252 | 10 | -2.36152 | -7.34528 | -36.2406 | 16.3368 | 14.9927 | 12.374 | -84.5325 |
| 36 | -20.6795 | 16 | -2.51782 | -8.26539 | -40.1754 | 13.8252 | 16.303 | 14.51 | -98.5031 |
| 37 | -22.0696 | 12 | -2.5451 | -8.51226 | -38.1612 | 11.1282 | 15.0294 | 14.7907 | -99.8975 |
| 38 | -16.1916 | 16 | -2.56398 | -8.67939 | -37.941 | 11.5848 | 19.4621 | 14.9316 | -96.1608 |
| 39 | -19.1773 | 18 | -2.58656 | -8.25353 | -44.1522 | 15.5418 | 22.9423 | 13.7 | -92.1845 |
| 40 | -17.6867 | 19 | -5.29553 | -9.21369 | -35.7206 | 19.0037 | 19.0522 | 18.0541 | -89.7046 |
| 41 | -26.1131 | 18 | -3.27175 | -8.66143 | -46.1433 | 22.4428 | 18.254 | 14.5763 | -35.8689 |
| 42 | -26.6733 | 10 | -5.15446 | -7.33317 | -41.1625 | 30.3421 | 25.9304 | 10.599 | -64.0164 |
| 43 | -14.9215 | 18 | -2.61698 | -8.67928 | -38.8268 | 12.3573 | 18.6481 | 17.5079 | -83.3696 |
| 44 | -18.6773 | 16 | -2.52405 | -9.25064 | -39.5947 | 14.2703 | 18.3711 | 15.3376 | -104.283 |
| 45 | -22.086 | 14 | -4.3967 | -8.14403 | -34.7582 | 11.2748 | 18.2914 | 10.8087 | -94.1192 |
| 46 | -15.687 | 22 | -9.56727 | -9.00416 | -26.6788 | 22.3329 | 23.2806 | 16.0142 | -40.445 |
| 47 | -17.4607 | 12 | -2.61112 | -9.03952 | -33.6936 | 11.6192 | 16.2963 | 14.3535 | -84.4241 |
| 48 | -12.82 | 10 | -2.39798 | -8.52262 | -28.9767 | 16.5878 | 16.1219 | 14.9125 | -71.1068 |
| 49 | -21.4023 | 14 | -2.63424 | -8.26073 | -41.5571 | 16.4478 | 19.5536 | 14.0742 | -116.898 |
| 50 | -32.8294 | 20 | -9.06068 | -8.88463 | -47.8241 | 25.7986 | 25.5297 | 19.2783 | -35.107 |
| 51 | -19.7214 | 16 | -5.31898 | -7.54709 | -36.7591 | 12.1423 | 21.6552 | 14.7119 | -103.46 |
| 52 | -13.1235 | 18 | -5.2408 | -9.03722 | -33.9595 | 13.2656 | 23.4396 | 17.8341 | -79.6506 |
| 53 | -16.203 | 10 | -2.47892 | -8.04864 | -31.489 | 10.6565 | 15.3426 | 14.308 | -101.359 |
| 54 | -22.5998 | 14 | -5.86029 | -7.1053 | -35.5084 | 12.044 | 19.6931 | 14.1302 | -97.4982 |
| 55 | -18.7431 | 12 | -2.60387 | -7.61655 | -37.8637 | 16.9743 | 18.4164 | 14.8472 | -87.605 |
| 56 | -21.3524 | 16 | -2.5501 | -8.4687 | -40.1904 | 16.0211 | 17.6569 | 12.3047 | -105.346 |
| 57 | -16.9764 | 12 | -3.49836 | -7.3115 | -32.8885 | 10.7855 | 18.7064 | 12.7754 | -76.2627 |
| 58 | -15.2029 | 18 | -3.43431 | -8.22474 | -38.799 | 15.2263 | 23.1197 | 14.6298 | -76.7314 |
| 59 | -20.3568 | 8 | -2.02519 | -6.76521 | -33.3884 | 10.5261 | 13.782 | 11.4416 | -76.8232 |
| 60 | -21.6217 | 18 | -3.31224 | -9.52167 | -41.2522 | 23.0059 | 17.5168 | 15.837 | -79.5938 |
| 61 | -19.0258 | 18 | -3.86411 | -7.71228 | -42.829 | 17.7642 | 24.7789 | 14.1856 | -117.638 |
| 62 | -16.4223 | 18 | -2.6758 | -8.65864 | -38.2968 | 15.1949 | 19.4205 | 13.7579 | -86.1987 |
| 63 | -18.327 | 14 | -4.53557 | -6.55565 | -45.0038 | 34.1577 | 30.3994 | 17.1886 | -15.6923 |
| 64 | -27.8006 | 14 | -4.31106 | -10.3174 | -46.8756 | 34.5822 | 23.7238 | 16.2148 | -121.696 |
| 65 | -25.0224 | 12 | -3.15356 | -9.10838 | -42.8353 | 17.2131 | 17.7826 | 17.2078 | -78.6214 |
| 66 | -22.4955 | 24 | -7.33738 | -10.029 | -42.7254 | 21.4415 | 24.9357 | 18.8049 | -84.397 |
| 67 | -16.2435 | 12 | -2.08036 | -7.33042 | -33.9909 | 14.7686 | 16.1596 | 13.0339 | -77.8389 |
| 68 | -18.0019 | 16 | -2.62421 | -8.20746 | -38.4836 | 13.3511 | 19.1095 | 13.1635 | -85.1747 |
| 69 | -13.5197 | 12 | -1.28817 | -8.93231 | -37.9345 | 0 | 19.9532 | 17.4145 | -97.8394 |
| 70 | -21.7309 | 18 | -4.7941 | -6.76075 | -46.0135 | 13.8095 | 23.129 | 19.3858 | -96.531 |
| 71 | -19.7422 | 14 | -3.0947 | -8.0085 | -36.4169 | 19.9692 | 17.5309 | 12.6087 | -75.6098 |
| 72 | -23.842 | 16 | -4.71311 | -8.12939 | -41.6683 | 20.4374 | 23.1929 | 12.428 | -119.625 |
| 73 | -22.0397 | 18 | -9.55061 | -8.27591 | -35.418 | 16.618 | 25.3347 | 20.1291 | -66.6887 |
| 74 | -18.994 | 16 | -7.24179 | -7.28315 | -34.9053 | 15.3875 | 23.9511 | 17.4992 | -78.9133 |
| 75 | -19.9141 | 18 | -2.74801 | -7.81414 | -47.8379 | 27.2306 | 24.9083 | 16.1067 | -67.0235 |
| 76 | -24.3404 | 18 | -4.25668 | -8.43754 | -46.1476 | 18.8844 | 20.4419 | 18.3307 | -96.7317 |
| 77 | -22.0706 | 10 | -1.52644 | -8.677 | -44.4804 | 17.3639 | 17.8477 | 19.2416 | -95.7014 |
| 78 | -25.5894 | 14 | -2.11881 | -8.76513 | -53.2609 | 24.8964 | 25.4525 | 17.296 | -87.5414 |
| 79 | -19.3703 | 18 | -6.36577 | -7.47184 | -38.156 | 12.7807 | 22.7772 | 18.2035 | -83.4397 |
| 80 | -17.1563 | 12 | -3.08278 | -7.51936 | -35.1995 | 13.8049 | 20.266 | 12.8502 | -91.5369 |
| 81 | -17.2495 | 18 | -4.93675 | -8.63197 | -37.8698 | 18.0657 | 24.5006 | 14.7246 | -117.006 |
| 82 | -28.1957 | 16 | -2.66431 | -8.02172 | -49.6141 | 18.705 | 20.5377 | 12.9249 | -49.2406 |
| 83 | -15.8289 | 10 | -3.3902 | -7.04524 | -32.321 | 7.17716 | 21.805 | 11.2664 | -83.5634 |
| 84 | -19.7025 | 12 | -2.59647 | -8.22251 | -42.2805 | 16.5282 | 21.4866 | 17.1835 | -93.9476 |
| 85 | -20.923 | 14 | -3.98554 | -7.93095 | -36.918 | 16.4319 | 18.2857 | 14.0575 | -101.058 |
| 86 | -12.4815 | 12 | -3.132 | -9.01644 | -33.9915 | 14.4039 | 21.6789 | 18.2058 | -77.89 |
| 87 | -27.6384 | 18 | -8.74043 | -8.2704 | -38.5381 | 15.2468 | 20.512 | 18.7426 | -99.3424 |
| 88 | -16.2798 | 18 | -6.2621 | -9.35777 | -32.5887 | 21.6442 | 22.8201 | 15.9191 | -66.013 |
| 89 | -15.0666 | 14 | -4.16065 | -8.43636 | -32.9923 | 14.9433 | 19.6391 | 16.6291 | -87.876 |
| 90 | -22.6985 | 12 | -5.10194 | -6.16523 | -34.2886 | 9.44384 | 18.586 | 11.3473 | -76.6988 |
| 91 | -28.2007 | 14 | -7.81508 | -7.22258 | -42.7092 | 20.07 | 24.941 | 18.203 | -108.767 |
| 92 | -25.6194 | 10 | -3.367 | -7.27839 | -42.0286 | 23.7366 | 18.429 | 15.125 | -113.378 |
| 93 | -22.9568 | 10 | -2.44091 | -6.99079 | -37.0474 | 13.4922 | 14.7261 | 11.9778 | -79.7059 |
| 94 | -32.166 | 10 | -3.70066 | -8.77156 | -46.6317 | 28.1353 | 16.944 | 16.6626 | -98.6969 |
| 95 | -36.0292 | 10 | -5.18817 | -7.95919 | -49.2927 | 28.0829 | 25.002 | 10.5617 | -102.793 |
| 96 | -31.4296 | 10 | -8.61535 | -6.42436 | -47.7474 | 20.129 | 30.7575 | 20.2286 | -95.9724 |
| 97 | -32.9329 | 10 | -5.35346 | -7.93329 | -45.2394 | 24.431 | 22.7811 | 12.2428 | -83.3941 |
| 98 | -34.5884 | 12 | -6.11009 | -7.98249 | -47.6734 | 25.3662 | 23.3403 | 13.9191 | -58.0347 |
| 99 | -35.8765 | 10 | -3.45883 | -7.86237 | -53.9532 | 28.7244 | 25.5917 | 10.4569 | -107.942 |
| 100 | -40.9654 | 10 | -5.62563 | -7.9502 | -50.8283 | 26.4849 | 21.0436 | 11.5753 | -108.011 |
| 101 | -28.1644 | 10 | -4.2306 | -7.3442 | -43.6516 | 18.295 | 20.0047 | 15.3148 | -106.286 |
| 102 | -31.2659 | 14 | -10.4332 | -7.03357 | -42.4466 | 21.1945 | 24.9159 | 23.0339 | -78.3639 |
| 103 | -28.2636 | 10 | -2.43324 | -8.53701 | -46.7782 | 17.1904 | 17.1025 | 17.5206 | -102.442 |
| 104 | -23.8835 | 8 | -2.93037 | -6.76193 | -36.8767 | 7.43273 | 15.8378 | 12.5139 | -116.515 |
| 105 | -30.0786 | 11 | -6.88078 | -6.77913 | -48.8862 | 21.9945 | 25.1628 | 23.1606 | -120.684 |
| 106 | -22.0383 | 10 | -4.18105 | -6.7364 | -37.9774 | 14.075 | 20.4955 | 14.6348 | -74.0969 |
| 107 | -30.9565 | 13 | -8.26279 | -7.38845 | -44.445 | 22.9588 | 28.4447 | 15.4698 | -128.48 |
| 108 | -26.9196 | 10 | -5.42799 | -6.4232 | -42.911 | 14.4478 | 22.4156 | 16.994 | -113.082 |
| 109 | -29.5014 | 10 | -3.59021 | -7.97361 | -44.699 | 19.3309 | 19.3414 | 13.8137 | -106.165 |
| 110 | -24.0952 | 12 | -2.80716 | -8.1029 | -51.161 | 23.9403 | 23.3401 | 22.5633 | -61.8288 |
| 111 | -26.548 | 14 | -4.30192 | -8.33022 | -49.954 | 25.3382 | 23.578 | 20.8903 | -33.5751 |
| 112 | -28.5778 | 10 | -3.86012 | -7.82279 | -45.2629 | 17.1998 | 21.8108 | 14.143 | -95.1136 |
| 113 | -32.7032 | 12 | -5.62461 | -8.50624 | -44.9864 | 22.0608 | 23.1576 | 11.5824 | -56.2593 |
| 114 | -23.6347 | 14 | -5.18378 | -7.80718 | -44.3773 | 22.4271 | 23.6538 | 19.6182 | -88.1686 |
| 115 | -25.7043 | 14 | -5.19269 | -8.19193 | -41.35 | 18.8235 | 23.0179 | 13.0429 | -106.167 |
| 116 | -25.1133 | 16 | -4.356 | -8.34765 | -46.7192 | 23.425 | 24.7964 | 15.1851 | -111.589 |
| 117 | -20.2214 | 18 | -3.52016 | -8.28162 | -48.1678 | 17.2585 | 25.2648 | 19.1704 | -110.521 |
| 118 | -22.9138 | 14 | -3.75781 | -8.6563 | -42.6149 | 25.6775 | 21.9514 | 15.3705 | -52.1665 |
| 119 | -29.971 | 10 | -5.71644 | -7.94978 | -46.6571 | 18.0451 | 21.9107 | 21.2757 | -73.3937 |
| 120 | -32.799 | 10 | -7.26378 | -7.87674 | -48.988 | 36.773 | 27.2158 | 20.3514 | -139.483 |
| 121 | -31.6708 | 10 | -6.64498 | -7.23183 | -43.419 | 21.9039 | 23.7989 | 14.479 | -51.3722 |
| 122 | -30.5434 | 10 | -6.57681 | -7.6548 | -52.5054 | 22.3747 | 23.6365 | 30.307 | -81.6456 |
| 123 | -33.6227 | 10 | -8.70004 | -6.19644 | -45.6333 | 25.1582 | 30.1063 | 14.5357 | -80.6874 |
| 124 | -20.3523 | 10 | -4.25549 | -7.25481 | -45.0388 | 23.6502 | 27.4753 | 20.7684 | -72.4882 |
| 125 | -24.9347 | 10 | -5.73902 | -6.45272 | -46.8226 | 19.5244 | 28.3057 | 20.4571 | -101.987 |
| 126 | -18.5871 | 6 | -2.48925 | -6.27396 | -29.6392 | 7.85135 | 13.8656 | 11.4458 | -74.5552 |
| 127 | -19.0499 | 14 | -4.77501 | -7.55045 | -35.2031 | 13.8721 | 18.9296 | 16.2054 | -88.0113 |
| 128 | -16.8309 | 18 | -3.80952 | -7.22412 | -38.8195 | 18.4244 | 21.1936 | 14.8055 | -88.7868 |
| 129 | -23.7638 | 10 | -2.16959 | -7.57722 | -37.4288 | 16.9827 | 14.0784 | 11.6241 | -72.3612 |
| 130 | -18.6452 | 8 | -2.19543 | -7.00083 | -31.3212 | 11.4939 | 13.8622 | 11.7036 | -66.4759 |
| 131 | -22.3632 | 10 | -2.49831 | -7.40352 | -38.0782 | 14.4893 | 15.7617 | 13.9012 | -98.7971 |
| 132 | -21.8194 | 10 | -3.25968 | -7.26769 | -41.2602 | 15.6094 | 20.834 | 16.563 | -114.426 |
| 133 | -22.2098 | 10 | -3.43929 | -7.55042 | -37.6916 | 13.5691 | 18.3769 | 14.3323 | -100.223 |
| 134 | -25.7998 | 10 | -3.45753 | -7.84148 | -41.0745 | 18.5588 | 19.6741 | 12.9098 | -105.114 |
| 135 | -23.4141 | 10 | -2.72652 | -8.07918 | -42.7456 | 22.0145 | 21.7986 | 14.0814 | -108.633 |
| 136 | -26.9896 | 12 | -5.53854 | -8.33924 | -43.0557 | 19.3484 | 23.1611 | 16.8106 | -102.257 |
| 137 | -30.1838 | 10 | -4.88743 | -8.05876 | -43.4976 | 16.452 | 20.9148 | 14.2308 | -117.505 |
| 138 | -24.2288 | 10 | -3.24302 | -8.17574 | -43.895 | 14.7159 | 20.4864 | 18.1576 | -102.501 |
| 139 | -22.667 | 10 | -2.71886 | -8.28449 | -42.7111 | 18.6951 | 20.0385 | 17.3403 | -82.3952 |
| 140 | -24.4138 | 10 | -2.53768 | -7.97228 | -41.0399 | 15.5655 | 15.6687 | 16.1156 | -106.392 |
| 141 | -23.8804 | 10 | -2.50637 | -7.85052 | -41.2241 | 16.2929 | 18.1522 | 14.1372 | -104.264 |
| 142 | -24.5058 | 10 | -2.52279 | -8.1131 | -40.608 | 14.4106 | 16.2561 | 14.7403 | -103.403 |
| 143 | -19.4381 | 10 | -2.72471 | -7.90777 | -39.0681 | 17.9196 | 18.0059 | 18.6626 | -110.993 |
| 144 | -20.3267 | 10 | -2.43139 | -8.26522 | -37.1406 | 17.4152 | 15.8694 | 16.0622 | -72.2583 |
| 145 | -23.158 | 10 | -2.51321 | -8.30952 | -41.136 | 15.3005 | 16.9131 | 16.9986 | -112.062 |
| 146 | -27.0626 | 10 | -2.42354 | -8.27042 | -42.716 | 16.5056 | 17.0507 | 12.9365 | -104.399 |
| 147 | -27.5136 | 10 | -2.63421 | -7.44073 | -45.482 | 19.8172 | 19.5703 | 13.5463 | -100.672 |
| 148 | -26.0132 | 10 | -2.05444 | -8.54031 | -43.5756 | 20.4696 | 17.0797 | 14.655 | -102.377 |
| 149 | -22.2497 | 10 | -2.53501 | -8.32469 | -40.4367 | 15.8502 | 17.272 | 17.0062 | -111.653 |
| 150 | -28.0478 | 11 | -1.93085 | -8.48297 | -45.4406 | 20.7664 | 17.6167 | 12.3468 | -91.248 |
| 151 | -22.855 | 11 | -2.37687 | -8.2447 | -43.9459 | 18.1019 | 17.3568 | 19.7132 | -30.8727 |
| 152 | -19.2741 | 10 | -2.47342 | -8.72879 | -39.4252 | 21.1209 | 21.0345 | 15.8808 | -88.6541 |
| 153 | -21.4236 | 10 | -1.8919 | -8.18037 | -40.8772 | 20.7262 | 19.3097 | 13.9979 | -99.7827 |
| 154 | -22.6154 | 10 | -2.54004 | -8.49094 | -43.2689 | 16.8684 | 18.7505 | 19.2514 | -105.93 |
| 155 | -20.2577 | 10 | -1.30901 | -8.8316 | -42.2076 | 16.663 | 15.9134 | 20.0665 | -99.1405 |
| 156 | -28.9757 | 14 | -5.94655 | -8.07715 | -42.3512 | 16.623 | 19.2606 | 16.6463 | -100.095 |
| 157 | -22.8269 | 10 | -2.38905 | -8.14603 | -40.3134 | 16.4531 | 17.9923 | 14.3855 | -106.566 |
| 158 | -21.0947 | 11 | -2.58502 | -8.32396 | -40.4965 | 16.9727 | 18.1058 | 17.1813 | -101.893 |
| 159 | -26.104 | 10 | -4.64493 | -7.31959 | -36.2392 | 20.8461 | 19.9717 | 8.8063 | -115.207 |
| 160 | -28.6303 | 10 | -4.17247 | -7.9855 | -39.2056 | 19.9412 | 14.8937 | 14.1086 | -109.532 |
| 161 | -21.2261 | 9 | -0.9408 | -8.00745 | -41.3118 | 9.47696 | 14.2719 | 17.7682 | -100.993 |
| 162 | -21.2573 | 10 | -2.67526 | -8.41929 | -41.2215 | 18.2778 | 19.4156 | 17.8967 | -113.841 |
| 163 | -24.999 | 10 | -3.3662 | -7.74474 | -42.3017 | 16.4817 | 18.4724 | 16.8998 | -108.441 |
| 164 | -28.1195 | 25 | -3.57815 | -9.86544 | -53.6564 | 26.6542 | 24.1623 | 12.239 | -32.7222 |
| 165 | -31.722 | 25 | -4.15537 | -9.09303 | -57.304 | 30.9091 | 24.7838 | 13.0298 | -64.3838 |
| 166 | -34.0822 | 25 | -5.4466 | -10.2801 | -52.3094 | 35.8347 | 23.3723 | 9.64463 | -54.5159 |
| 167 | -38.615 | 24 | -3.52948 | -9.27537 | -60.8063 | 27.7362 | 22.954 | 8.66663 | -85.7634 |
| 168 | -14.5641 | 16 | -3.96632 | -8.60765 | -33.4564 | 21.7896 | 19.8323 | 15.485 | -68.734 |
| 169 | -13.9033 | 14 | -4.19895 | -8.74774 | -27.0343 | 30.2386 | 17.5817 | 12.1652 | -47.0639 |
| 170 | -14.9206 | 19 | -4.06232 | -10.1929 | -35.8058 | 26.9752 | 20.7857 | 16.6709 | -40.484 |
| 171 | -22.3484 | 13 | -2.54547 | -8.69541 | -35.4138 | 27.6154 | 16.3868 | 7.94131 | -17.7618 |
| 172 | -14.6791 | 15 | -4.11655 | -8.07821 | -34.1761 | 16.8279 | 22.5858 | 14.2196 | -78.0058 |
| 173 | -17.6952 | 14 | -2.68788 | -8.32122 | -31.4419 | 15.8578 | 15.8093 | 8.88677 | -16.9284 |
| 174 | -20.8447 | 14 | -1.50787 | -9.16822 | -41.2819 | 22.3954 | 17.5553 | 13.3763 | -81.7 |
| 175 | -17.9526 | 13 | -3.3941 | -9.24718 | -32.914 | 29.2026 | 19.4165 | 11.1845 | -57.8997 |
| 176 | -12.8561 | 13 | -3.12164 | -9.26849 | -29.9284 | 18.8223 | 18.5487 | 14.346 | -34.6626 |
| 177 | -16.7653 | 13 | -4.04152 | -8.80957 | -35.0174 | 18.68 | 21.1577 | 16.2292 | -61.3828 |
| 178 | -17.1025 | 13 | -3.29065 | -8.19733 | -33.451 | 24.8007 | 18.9839 | 12.3153 | -17.6174 |
| 179 | -23.3753 | 13 | -4.08931 | -8.76071 | -37.0189 | 24.146 | 21.2801 | 9.24112 | -51.4614 |
| 180 | -19.9223 | 13 | -2.64304 | -9.1683 | -37.3959 | 28.4709 | 18.2892 | 13.3126 | -91.4928 |
| 181 | -16.0179 | 13 | -4.04886 | -8.59919 | -29.3127 | 33.5572 | 16.6488 | 13.6565 | -12.2089 |
| 182 | -18.2638 | 13 | -2.66846 | -8.11746 | -33.08 | 31.8206 | 16.9591 | 9.82838 | -21.0621 |
| 183 | -15.9959 | 13 | -3.49444 | -8.04293 | -34.4687 | 14.1709 | 20.5764 | 14.3487 | -54.0535 |
| 184 | -20.4246 | 13 | -2.5523 | -8.05285 | -36.1451 | 20.7937 | 17.2549 | 10.3516 | -87.768 |
| 185 | -14.0301 | 13 | -4.46929 | -9.45241 | -31.4222 | 19.1992 | 20.5091 | 17.9557 | -51.9359 |
| 186 | -20.0936 | 13 | -2.71672 | -9.30312 | -41.1299 | 31.2858 | 19.406 | 17.8609 | -105.001 |
| 187 | -23.5267 | 14 | -2.56808 | -9.41931 | -44.5395 | 23.8428 | 20.3029 | 15.4452 | -91.2328 |
| 188 | -20.4981 | 12 | -1.51544 | -8.1836 | -41.2517 | 14.4579 | 17.9185 | 14.2907 | -89.5514 |
| 189 | -17.1178 | 12 | -3.2082 | -7.75141 | -35.3444 | 19.2457 | 18.9363 | 15.3554 | -52.8672 |
| 190 | -4.72541 | 11 | -1.26628 | -8.62414 | -27.5833 | 20.9855 | 19.0244 | 16.6234 | -40.9186 |
| 191 | -21.9286 | 12 | -4.29207 | -7.01037 | -38.846 | 20.6951 | 21.3044 | 14.0833 | -50.4814 |
| 192 | -24.1042 | 12 | -4.50469 | -7.18437 | -40.7313 | 20.1466 | 21.4775 | 14.4384 | -46.1786 |
| 193 | -20.1705 | 12 | -2.76295 | -8.13595 | -40.8762 | 20.0214 | 20.8676 | 15.6006 | -93.4468 |
| 194 | -11.672 | 12 | -3.08066 | -8.70777 | -31.6448 | 11.883 | 20.457 | 16.755 | -51.1124 |
| 195 | -19.0711 | 12 | -3.49182 | -7.95713 | -38.6905 | 16.0438 | 20.1748 | 17.3543 | -91.2078 |
| 196 | -12.7898 | 12 | -2.37904 | -8.279 | -27.863 | 11.6946 | 14.9567 | 12.457 | -29.3276 |
| 197 | -21.5942 | 12 | -3.40497 | -8.48181 | -33.7248 | 16.985 | 16.523 | 10.3573 | -43.8671 |
| 198 | -15.7553 | 12 | -2.75452 | -8.82987 | -34.4207 | 19.7453 | 18.3963 | 15.9896 | -71.1562 |
| 199 | -20.1398 | 12 | -4.46695 | -8.1454 | -30.5365 | 20.8519 | 17.2169 | 10.671 | -40.1373 |
| 200 | -13.2718 | 13 | -2.6441 | -8.7057 | -29.647 | 15.2319 | 18.5708 | 10.8629 | -43.4513 |
| 201 | -14.6336 | 13 | -2.00626 | -8.29798 | -35.3499 | 17.7558 | 18.6357 | 14.5076 | -104.601 |
| 202 | -21.6568 | 8 | -1.75946 | -7.00566 | -35.9881 | 4.17634 | 12.6939 | 13.8729 | -80.5492 |
| 203 | -22.6298 | 12 | -4.07003 | -7.13133 | -39.337 | 15.8991 | 18.2804 | 16.4729 | -73.4759 |
| 204 | -31.911 | 12 | -5.02821 | -7.29514 | -49.5611 | 15.5087 | 24.5706 | 14.5928 | -54.6817 |
| 205 | -29.722 | 12 | -4.16315 | -8.85448 | -45.8329 | 13.0318 | 21.439 | 14.0862 | -86.3952 |
| 206 | -23.0052 | 12 | -1.28022 | -7.94195 | -45.264 | 12.5067 | 21.1498 | 11.7524 | -82.968 |
| 207 | -39.2547 | 12 | -4.17744 | -8.59803 | -54.3176 | 25.878 | 19.4511 | 14.5518 | -41.4508 |
| 208 | -26.2718 | 12 | -1.14953 | -9.4855 | -51.8781 | 15.599 | 17.6776 | 21.8359 | -67.6757 |
| 209 | -21.9444 | 10 | -1.73846 | -7.51768 | -39.8314 | 7.03025 | 15.0958 | 15.152 | -84.829 |
| 210 | -22.1828 | 12 | -3.08467 | -7.70197 | -41.6678 | 14.3014 | 16.8401 | 19.1203 | -90.9457 |
| 211 | -21.7924 | 12 | -2.89137 | -7.77333 | -46.4734 | 16.8008 | 23.7972 | 18.4189 | -82.9657 |
| 212 | -26.4536 | 12 | -2.0983 | -8.7945 | -47.1987 | 26.2672 | 18.0688 | 16.9613 | -101.264 |
| 213 | -34.1084 | 12 | -3.12378 | -9.02349 | -49.808 | 22.376 | 17.4469 | 14.1868 | -82.5616 |
| 214 | -32.1663 | 12 | -4.00638 | -8.02777 | -48.0127 | 26.1695 | 19.1521 | 14.8441 | -63.1074 |
| 215 | -24.1972 | 12 | -1.75712 | -7.71493 | -47.1468 | 30.4494 | 22.5258 | 12.833 | -89.5076 |
| 216 | -25.7956 | 12 | -4.81708 | -8.28396 | -47.9222 | 17.5681 | 21.3762 | 25.1998 | -87.6951 |
| 217 | -36.6957 | 12 | -5.5449 | -7.30025 | -49.0282 | 29.8822 | 21.4065 | 12.1172 | -60.9417 |
| 218 | -32.8842 | 12 | -2.30481 | -8.56163 | -53.7939 | 34.9348 | 19.3987 | 16.2548 | -54.414 |
| 219 | -26.2368 | 17 | -4.83995 | -8.36901 | -46.0986 | 27.4951 | 25.7514 | 12.4251 | -52.4845 |
| 220 | -21.7712 | 12 | -1.9565 | -8.15354 | -44.4016 | 16.8038 | 19.3417 | 17.1772 | -95.1744 |
| 221 | -25.3678 | 16 | -6.67895 | -8.04825 | -43.4051 | 20.2312 | 26.3795 | 16.58 | -109.27 |
| 222 | -28.0561 | 12 | -1.86056 | -7.83168 | -49.5034 | 15.1375 | 19.4215 | 14.5994 | -103.625 |
| 223 | -30.8884 | 18 | -5.197 | -8.1137 | -47.1061 | 23.2098 | 22.5167 | 10.78 | -97.6089 |
| 224 | -26.8981 | 20 | -2.3153 | -9.97249 | -49.7812 | 30.2237 | 18.0677 | 14.9569 | -88.0278 |
| 225 | -34.2629 | 16 | -6.89416 | -7.62214 | -42.3269 | 14.9787 | 20.4008 | 8.65529 | -41.9574 |
| 226 | -32.8717 | 19 | -3.32277 | -8.89386 | -51.4206 | 21.3971 | 17.3043 | 12.9336 | -88.601 |
| 227 | -34.1926 | 19 | -6.8661 | -8.493 | -47.1811 | 18.6763 | 21.7069 | 12.6816 | -88.942 |
| 228 | -29.227 | 19 | -8.0113 | -8.28285 | -39.4194 | 21.1726 | 21.5701 | 12.7771 | -38.3007 |
| 229 | -31.4073 | 19 | -3.78123 | -7.87565 | -51.0642 | 15.1251 | 18.0969 | 14.4355 | -117.254 |
| 230 | -31.3416 | 19 | -5.70352 | -8.52974 | -49.3279 | 18.1251 | 23.6366 | 13.642 | -139.49 |
| 231 | -34.0804 | 19 | -3.69127 | -8.75443 | -51.8473 | 18.1672 | 17.6012 | 12.6823 | -102.237 |
| 232 | -31.0739 | 19 | -5.05809 | -9.05516 | -46.3744 | 25.1891 | 19.7334 | 12.0672 | -77.1181 |
| 233 | -29.4388 | 19 | -5.83329 | -8.24586 | -42.1867 | 12.9437 | 20.0278 | 10.0151 | -106.783 |
| 234 | -36.4702 | 19 | -5.73078 | -9.07319 | -52.5683 | 25.4226 | 21.4162 | 13.9603 | -60.1182 |
| 235 | -30.5707 | 19 | -5.96474 | -8.58129 | -49.5964 | 18.3697 | 24.5732 | 15.206 | -121.589 |
| 236 | -35.7232 | 17 | -7.34482 | -7.45871 | -49.0547 | 29.2542 | 20.6085 | 17.0528 | -75.8464 |
| 237 | -43.826 | 17 | -6.54472 | -7.74992 | -50.4213 | 18.8895 | 17.7809 | 7.28823 | -95.3643 |
| 238 | -29.5264 | 17 | -6.31058 | -8.26483 | -39.7943 | 35.0278 | 19.8367 | 10.1417 | -56.2916 |
| 239 | -23.055 | 17 | -4.98163 | -7.78951 | -44.1209 | 17.9233 | 25.5235 | 14.4632 | -90.3161 |
| 240 | -34.0159 | 17 | -4.54662 | -8.56173 | -52.294 | 26.6429 | 18.7581 | 17.0437 | -59.1954 |
| 241 | -39.9061 | 17 | -5.99029 | -7.81668 | -51.4761 | 20.5324 | 20.2406 | 9.97303 | -91.7074 |
| 242 | -43.0022 | 17 | -8.08863 | -8.44783 | -48.1648 | 26.6923 | 16.6128 | 13.0717 | -81.1699 |
| 243 | -37.6511 | 17 | -4.1292 | -8.37869 | -52.9042 | 23.2168 | 17.4282 | 12.1857 | -110.167 |
| 244 | -28.8526 | 17 | -4.09612 | -8.17691 | -47.4148 | 20.6309 | 21.5725 | 12.1583 | -74.2121 |
| 245 | -34.2642 | 17 | -4.37483 | -8.7634 | -52.4393 | 32.3081 | 18.9769 | 16.1851 | -77.9197 |
| 246 | -19.5768 | 11 | -3.96816 | -8.61632 | -32.669 | 18.4709 | 21.2539 | 9.6427 | -32.7468 |
| 247 | -10.3215 | 11 | -2.29532 | -9.39747 | -26.9656 | 14.1435 | 19.2719 | 11.6571 | -48.7463 |
| 248 | -19.7255 | 11 | -3.55756 | -8.90556 | -31.5278 | 22.7506 | 17.94 | 10.1737 | -35.5296 |
| 249 | -14.5041 | 11 | -1.12343 | -7.87645 | -30.2568 | 13.0391 | 14.9104 | 9.22636 | -75.8886 |
| 250 | -14.3122 | 11 | -2.9687 | -8.74262 | -36.6075 | 22.5294 | 20.5091 | 20.7299 | -76.8369 |
| 251 | -9.59055 | 12 | -3.73063 | -8.37754 | -23.5544 | 17.2905 | 16.3043 | 14.5259 | -8.37093 |
| 252 | -21.9309 | 12 | -2.6464 | -8.76878 | -38.2232 | 20.2494 | 16.7711 | 13.7644 | -49.4261 |
| 253 | -13.5691 | 12 | -2.0995 | -8.94136 | -29.0016 | 19.5695 | 15.712 | 11.7438 | -59.7466 |
| 254 | -18.9117 | 12 | -4.08925 | -8.39766 | -31.785 | 22.043 | 17.8293 | 12.5356 | -87.2978 |
| 255 | -19.24 | 13 | -4.21627 | -8.63941 | -32.554 | 24.7844 | 17.5733 | 13.3175 | -83.7256 |
| 256 | -22.073 | 14 | -5.83007 | -7.60805 | -33.3802 | 19.0566 | 21.5768 | 9.95763 | -69.3429 |
| 257 | -25.6444 | 14 | -4.62718 | -7.59535 | -39.9384 | 8.26051 | 19.9806 | 11.6731 | -91.7431 |
| 258 | -28.9224 | 14 | -4.46553 | -8.14845 | -41.5053 | 17.9303 | 18.5194 | 10.6828 | -72.8488 |
| 259 | -37.844 | 14 | -6.07147 | -8.1002 | -39.5981 | 16.0776 | 14.1143 | 5.38843 | -32.4616 |
| 260 | -19.264 | 11 | -3.37083 | -8.08561 | -30.6426 | 13.6961 | 15.7735 | 10.4456 | -53.4295 |
| 261 | -22.7053 | 11 | -4.09234 | -8.58126 | -40.2061 | 20.9111 | 21.6613 | 16.2997 | -93.7468 |
| 262 | -17.9813 | 11 | -4.51092 | -8.21451 | -34.8664 | 23.0628 | 21.526 | 16.7529 | -92.1246 |
| 263 | -18.357 | 6 | -4.51989 | -6.15087 | -32.4345 | 6.75828 | 20.8986 | 15.6969 | -85.9642 |
| 264 | -20.8789 | 5 | -5.6292 | -6.85051 | -32.5156 | 20.2091 | 21.3377 | 17.3715 | -91.0347 |
| 265 | -17.5965 | 6 | -2.42689 | -6.88104 | -33.9278 | 6.37433 | 17.1302 | 16.1122 | -84.3419 |
| 266 | -21.4092 | 6 | -2.66741 | -6.95364 | -37.1775 | 17.3828 | 18.1131 | 15.1379 | -53.5926 |
| 267 | -23.8564 | 7 | -3.62648 | -7.88628 | -38.1034 | 27.2395 | 20.1535 | 14.2283 | -65.998 |
| 268 | -24.6169 | 8 | -3.67095 | -8.252 | -40.644 | 14.6504 | 21.6357 | 14.8736 | -91.1464 |
| 269 | -23.7128 | 8 | -3.38387 | -8.29491 | -40.2259 | 17.6079 | 22.0448 | 14.0882 | -95.6423 |
| 270 | -21.7648 | 8 | -2.95589 | -7.94502 | -36.7261 | 9.67046 | 19.3706 | 12.7801 | -69.4364 |
| 271 | -24.7675 | 8 | -3.66591 | -8.2319 | -40.5381 | 16.2283 | 22.2222 | 13.7789 | -89.362 |
| 272 | -21.6801 | 8 | -3.74663 | -7.83879 | -40.5912 | 17.2824 | 24.3423 | 16.0384 | -91.7982 |
| 273 | -23.7539 | 8 | -3.26517 | -8.60358 | -44.6949 | 29.9299 | 22.4801 | 20.1609 | -51.1246 |
| 274 | -24.4262 | 8 | -4.68543 | -7.95943 | -38.4878 | 17.0305 | 23.0751 | 13.8519 | -79.8578 |
| 275 | -23.1348 | 7 | -3.88193 | -7.24808 | -41.4763 | 10.6747 | 22.2621 | 18.3674 | -86.7481 |
| 276 | -24.7071 | 7 | -0.52439 | -6.93359 | -44.6711 | 38.3648 | 17.9379 | 12.5498 | -33.0575 |
| 277 | -21.7549 | 7 | -3.82814 | -7.04003 | -41.0517 | 10.2093 | 23.5952 | 17.8825 | -93.6518 |
| 278 | -22.1348 | 8 | -2.97725 | -7.222 | -40.9091 | 11.9968 | 21.9416 | 14.9838 | -75.475 |
| 279 | -22.383 | 8 | -1.83751 | -6.87238 | -42.4206 | 73.7755 | 22.0463 | 12.0549 | -42.3912 |
| 280 | -17.0366 | 8 | -2.38935 | -8.76061 | -40.6972 | 9.67281 | 24.4386 | 18.858 | -108.072 |
| 281 | -16.7724 | 8 | -3.62083 | -8.60044 | -36.2985 | 20.9896 | 24.2553 | 17.3601 | -98.0137 |
| 282 | -18.643 | 9 | -3.85622 | -7.63908 | -37.3873 | 21.3553 | 22.2902 | 17.4259 | -57.2421 |
| 283 | -35.1633 | 9 | -6.92039 | -7.80109 | -41.0972 | 18.1548 | 21.1256 | 11.2499 | -94.985 |
| 284 | -18.8783 | 6 | -3.71576 | -7.71997 | -32.5744 | 9.73273 | 18.0261 | 16.8936 | -58.2019 |
| 285 | -14.4893 | 8 | -1.08607 | -6.9443 | -35.2104 | 9.64495 | 18.5199 | 14.2903 | -54.7651 |
| 286 | -20.5821 | 8 | -0.34349 | -8.59742 | -44.0683 | 27.5471 | 18.1952 | 17.6804 | -58.323 |
| 287 | -23.174 | 8 | -2.09446 | -7.86338 | -38.3664 | 13.2522 | 15.0864 | 14.6138 | -56.6393 |
| 288 | -21.6121 | 8 | -3.09511 | -8.2896 | -40.1232 | 12.4925 | 22.0164 | 16.0355 | -97.6719 |
| 289 | -16.825 | 8 | -1.32002 | -6.22071 | -37.1603 | 14.4719 | 19.6228 | 12.6148 | -61.9857 |
| 290 | -11.8641 | 8 | -0.57798 | -8.27243 | -37.8638 | 16.8432 | 22.3533 | 17.3326 | -57.2426 |
| 291 | -28.8636 | 8 | -3.40538 | -8.25045 | -40.7243 | 16.5514 | 17.5026 | 12.2381 | -106.039 |
| 292 | -22.8719 | 9 | -5.64417 | -7.53636 | -31.6895 | 22.7456 | 20.187 | 11.5248 | -77.4263 |
| 293 | -30.371 | 10 | -4.28533 | -7.24175 | -37.8291 | 16.146 | 16.2764 | 7.49184 | -24.0191 |
| 294 | -13.3113 | 10 | -2.23014 | -7.46184 | -30.7299 | 19.9654 | 18.0567 | 12.9057 | -58.8156 |
| 295 | -18.8568 | 8 | -1.97622 | -6.89967 | -35.0929 | 24.1166 | 16.7794 | 12.8397 | -4.7588 |
| 296 | -22.6017 | 9 | -3.56806 | -7.88557 | -38.9572 | 14.1839 | 21.543 | 13.8011 | -78.0372 |
| 297 | -19.0491 | 8 | -3.70845 | -8.79882 | -35.9879 | 76.0609 | 20.1899 | 18.5969 | -22.6603 |
| 298 | -23.37 | 9 | -3.88924 | -7.76544 | -40.075 | 17.1922 | 22.14 | 14.7616 | -76.2106 |
| 299 | -20.7451 | 9 | -4.24125 | -8.73403 | -35.0081 | 37.0606 | 20.2525 | 15.5391 | -56.9198 |
| 300 | -24.5006 | 8 | -3.73597 | -7.47346 | -38.9727 | 15.0168 | 20.1914 | 13.6178 | -91.8599 |
| 301 | -20.5059 | 7 | -3.36246 | -8.00095 | -37.2978 | 16.3644 | 20.7318 | 16.5314 | -75.5772 |
| 302 | -28.5905 | 16 | -6.09849 | -5.838 | -37.1829 | 12.288 | 20.5416 | 4.4346 | -68.905 |
| 303 | -31.5904 | 19 | -6.67799 | -7.96489 | -41.7257 | 20.4112 | 17.497 | 11.8853 | -78.1436 |
| 304 | -33.7226 | 18 | -5.61215 | -7.38357 | -46.7908 | 22.7845 | 20.595 | 9.04194 | -70.1468 |
| 305 | -34.4714 | 21 | -7.40761 | -8.3257 | -46.3863 | 21.4495 | 19.6027 | 13.534 | -86.2126 |
| 306 | -35.9547 | 18 | -7.4894 | -8.05681 | -43.1589 | 19.1386 | 20.9211 | 7.66605 | -53.8578 |
| 307 | -33.5205 | 20 | -6.24453 | -7.57402 | -45.7841 | 20.9021 | 17.3986 | 12.2544 | -78.6322 |
| 308 | -25.1417 | 19 | -3.7581 | -8.36897 | -49.8351 | 16.5812 | 19.2157 | 21.2073 | -115.207 |
| 309 | -30.8685 | 19 | -5.06923 | -7.8023 | -45.866 | 24.1535 | 16.7522 | 13.7668 | -66.9297 |
| 310 | -35.0182 | 19 | -5.1021 | -8.38429 | -44.923 | 23.9263 | 15.834 | 7.81081 | -75.3059 |
| 311 | -40.0092 | 19 | -4.63684 | -9.15648 | -49.9805 | 31.8367 | 17.5478 | 4.96443 | -95.3107 |
| 312 | -23.8781 | 16 | -2.6788 | -8.30142 | -48.5047 | 11.7272 | 21.6501 | 16.8611 | -101.718 |
| 313 | -32.3911 | 16 | -6.18801 | -8.46603 | -47.8601 | 19.3871 | 23.8098 | 14.1508 | -103.514 |
| 314 | -18.2019 | 20 | -4.47121 | -9.02043 | -42.7044 | 21.5461 | 19.8997 | 22.6267 | -64.6273 |
| 315 | -29.1055 | 17 | -5.45116 | -8.39792 | -48.7615 | 13.0456 | 23.8645 | 16.6297 | -67.8437 |
| 316 | -18.431 | 19 | -4.76625 | -8.30414 | -39.847 | 21.9036 | 24.4796 | 14.0586 | -60.8469 |
| 317 | -24.8094 | 16 | -3.35102 | -8.71224 | -48.7212 | 19.1895 | 23.2821 | 16.9174 | -107.472 |
| 318 | -37.5185 | 19 | -4.83589 | -8.42643 | -46.3353 | 25.6952 | 15.3129 | 5.77677 | -94.1928 |
| 319 | -29.7651 | 16 | -3.91339 | -8.09748 | -50.0539 | 14.9037 | 21.823 | 14.6179 | -114.358 |
| 320 | -30.9952 | 19 | -5.68877 | -7.97987 | -50.9015 | 21.1884 | 25.4784 | 13.8433 | -97.027 |
| 321 | -30.2311 | 19 | -8.96702 | -8.18152 | -39.7354 | 18.0458 | 21.5116 | 15.3617 | -69.6988 |
| 322 | -29.7863 | 19 | -5.84216 | -7.17736 | -48.945 | 17.1786 | 24.7284 | 13.3363 | -107.231 |
| 323 | -24.5498 | 16 | -2.90469 | -7.80633 | -44.9126 | 13.7096 | 21.178 | 11.3007 | -95.192 |
| 324 | -29.8153 | 16 | -6.5435 | -6.95566 | -42.1821 | 14.6271 | 20.2997 | 13.2688 | -80.1194 |
| 325 | -34.8813 | 19 | -7.22169 | -7.78205 | -45.8611 | 14.4821 | 20.6578 | 11.4716 | -93.8523 |
| 326 | -36.5489 | 18 | -8.28938 | -7.84479 | -43.2536 | 17.4156 | 23.6052 | 6.71044 | -83.478 |
| 327 | -22.4552 | 21 | -4.80907 | -7.92915 | -42.7035 | 16.4218 | 25.5077 | 9.08643 | -84.7718 |
| 328 | -26.3754 | 2 | -4.55585 | -5.06502 | -30.6671 | 7.74695 | 16.7606 | 8.24403 | -63.941 |
| 329 | -27.6672 | 2 | -5.21151 | -4.6247 | -28.6945 | 5.13456 | 11.9711 | 10.8071 | -70.6478 |
| 330 | -38.2784 | 3 | -6.79765 | -6.32644 | -38.2518 | 12.1776 | 19.3906 | 7.67769 | -84.4646 |
| 331 | -48.3746 | 6 | -8.02981 | -8.75836 | -52.9618 | 28.5879 | 28.241 | 9.07582 | -138.46 |
| 332 | -11.7255 | 6 | -4.87823 | -5.44239 | -18.105 | 6.3886 | 19.0647 | 6.77161 | -67.1212 |
| 333 | -24.4577 | 2 | -1.5792 | -6.12951 | -35.4482 | 3.57467 | 12.8768 | 12.4773 | -84.686 |
| 334 | -19.2437 | 1 | -3.58473 | -4.36635 | -22.8069 | 5.4447 | 11.4432 | 9.6602 | -55.6491 |
| 335 | -22.521 | 11 | -3.78804 | -7.85688 | -29.982 | 19.772 | 15.2555 | 6.46541 | -72.0773 |
| 336 | -17.6579 | 11 | -2.67651 | -8.86688 | -32.0535 | 13.8232 | 16.3852 | 12.4532 | -57.1449 |
| 337 | -21.6522 | 11 | -4.21493 | -8.13069 | -33.4654 | 16.258 | 15.9375 | 14.1992 | -100.399 |
| 338 | -24.7691 | 11 | -3.53925 | -8.07232 | -31.277 | 17.4946 | 15.4845 | 4.02618 | -68.7815 |
| 339 | -18.4316 | 11 | -3.33893 | -8.53815 | -28.5507 | 15.4396 | 18.4896 | 5.78781 | -66.1607 |
| 340 | -21.4194 | 11 | -2.50613 | -7.76248 | -32.6003 | 17.5571 | 15.7281 | 6.55486 | -80.1532 |
| 341 | -21.8359 | 11 | -2.53818 | -7.88962 | -33.8314 | 20.0471 | 15.5597 | 8.23225 | -102.587 |
| 342 | -20.1957 | 11 | -3.25859 | -7.76368 | -29.3644 | 26.3282 | 15.7344 | 6.38462 | -75.9544 |
| 343 | -23.2979 | 11 | -4.88123 | -7.61749 | -31.255 | 18.3954 | 17.3793 | 8.75119 | -86.0391 |
| 344 | -24.1041 | 11 | -2.44714 | -7.95643 | -34.3578 | 19.7085 | 16.1284 | 4.6659 | -92.7144 |
| 345 | -19.23 | 11 | -2.47023 | -8.22861 | -32.9621 | 18.0574 | 16.9069 | 9.41637 | -100.443 |
| 346 | -21.7995 | 11 | -5.88019 | -8.66098 | -25.2992 | 16.644 | 15.3222 | 9.22391 | -14.5974 |
| 347 | -14.9746 | 13 | -3.50974 | -8.73297 | -32.8011 | 20.0065 | 19.4081 | 15.4562 | -79.264 |
| 348 | -7.18697 | 13 | -2.69753 | -8.71241 | -28.1691 | 15.8034 | 21.0644 | 15.221 | -82.0204 |
| 349 | -20.7854 | 12 | -2.50992 | -8.29758 | -34.7885 | 16.6495 | 17.1475 | 8.86665 | -109.98 |
| 350 | -22.631 | 12 | -5.40913 | -9.07477 | -30.8998 | 22.6929 | 16.982 | 12.2689 | -49.0113 |
| 351 | -18.8849 | 14 | -2.66175 | -8.59059 | -34.8837 | 21.6849 | 18.5969 | 9.30456 | -99.9759 |
| 352 | -19.2085 | 11 | -3.26036 | -7.27766 | -35.0213 | 14.113 | 15.9895 | 15.6749 | -112.838 |
| 353 | -22.6227 | 11 | -4.29456 | -6.71345 | -36.2831 | 15.8797 | 17.705 | 13.856 | -97.3545 |
| 354 | -18.8662 | 11 | -1.9993 | -7.52275 | -40.1536 | 15.2325 | 20.3655 | 14.4118 | -115.287 |
| 355 | -23.7835 | 11 | -1.88886 | -7.02155 | -43.8243 | 27.4271 | 18.7981 | 13.3721 | -103.621 |
| 356 | -25.238 | 11 | -1.4268 | -7.15024 | -46.7683 | 19.0668 | 16.5679 | 16.5225 | -119.23 |
| 357 | -17.0782 | 13 | -2.08353 | -7.91243 | -32.923 | 17.2761 | 16.9176 | 8.98379 | -31.5976 |
| 358 | -16.019 | 13 | -3.52126 | -8.91174 | -33.8484 | 19.7834 | 19.5803 | 15.4907 | -84.5847 |
| 359 | -17.8373 | 11 | -3.66829 | -8.1885 | -31.743 | 24.3949 | 18.372 | 12.5664 | -25.9725 |
| 360 | -15.3761 | 11 | -3.90224 | -7.38048 | -27.6269 | 21.2779 | 18.3364 | 10.1759 | -44.0593 |
| 361 | -9.75773 | 11 | -1.74219 | -8.87093 | -27.7839 | 16.4968 | 17.722 | 12.8575 | -28.8445 |
| 362 | -19.523 | 11 | -3.89595 | -8.69368 | -33.4063 | 17.5576 | 19.5113 | 12.6233 | -69.5654 |
| 363 | -13.3262 | 11 | -2.73023 | -8.58482 | -29.2297 | 19.3626 | 17.5453 | 13.3392 | -67.8653 |
| 364 | -22.1492 | 11 | -2.65725 | -8.24757 | -33.7848 | 20.2591 | 16.5209 | 7.41465 | -96.1696 |
| 365 | -9.12196 | 11 | -3.59183 | -8.00776 | -25.65 | 7.54057 | 20.3307 | 13.8373 | -61.1358 |
| 366 | -19.8916 | 11 | -2.77778 | -8.46029 | -35.1308 | 17.6376 | 19.0659 | 10.6606 | -87.1781 |
| 367 | -19.7477 | 11 | -2.67264 | -7.56371 | -33.2118 | 17.1356 | 17.0886 | 8.90473 | -79.9801 |
| 368 | -21.6879 | 11 | -3.99991 | -8.32134 | -31.8624 | 17.6536 | 19.7277 | 6.77852 | -76.196 |
| 369 | -22.9568 | 11 | -4.40042 | -8.18845 | -32.6394 | 17.5225 | 19.1963 | 8.03748 | -97.3948 |
| 370 | -9.71301 | 11 | -2.65523 | -7.3799 | -31.4969 | 19.3258 | 21.6241 | 16.1033 | -71.0421 |
| 371 | -30.2492 | 17 | -7.44436 | -7.70729 | -44.5553 | 17.8994 | 24.0595 | 15.2418 | -67.0799 |
| 372 | -28.1431 | 19 | -4.60016 | -8.60454 | -40.8269 | 17.3067 | 14.2155 | 12.1601 | -105.355 |
| 373 | -30.671 | 17 | -3.12772 | -7.93816 | -50.123 | 21.0581 | 19.2222 | 12.2228 | -67.7105 |
| 374 | -22.2639 | 17 | -1.85956 | -8.42462 | -48.8091 | 25.0156 | 21.0512 | 16.5242 | -91.8477 |
| 375 | -28.2882 | 19 | -5.69644 | -8.09106 | -44.4125 | 27.1184 | 20.2593 | 14.1861 | -59.3859 |
| 376 | -23.9021 | 19 | -5.18293 | -8.40717 | -39.7115 | 22.6495 | 19.88 | 12.4925 | -118.972 |
| 377 | -40.0116 | 15 | -6.71354 | -7.89461 | -45.5945 | 27.6561 | 19.339 | 6.59591 | -84.3421 |
| 378 | -36.7722 | 17 | -5.93479 | -8.71269 | -54.0921 | 29.8182 | 21.3625 | 18.1068 | -99.054 |
| 379 | -34.619 | 19 | -5.65546 | -10.5115 | -54.0279 | 29.2062 | 22.9443 | 18.4118 | -86.1455 |
| 380 | -31.7389 | 17 | -4.66803 | -9.28896 | -48.8917 | 18.0581 | 21.2368 | 13.7257 | -94.1905 |
| 381 | -41.0174 | 18 | -8.41472 | -9.57104 | -51.6845 | 24.1886 | 24.2383 | 14.2276 | -77.9924 |
| 382 | -35.7593 | 17 | -8.48378 | -9.58473 | -46.2261 | 33.0808 | 25.1117 | 14.1194 | -116.457 |
| 383 | -30.5929 | 19 | -3.81414 | -10.5657 | -53.0757 | 28.8346 | 20.162 | 19.2274 | -87.0947 |
| 384 | -31.6206 | 21 | -5.71799 | -9.61814 | -54.0839 | 37.4876 | 29.4182 | 13.1974 | -113.127 |
| 385 | -33.8956 | 19 | -1.39169 | -11.1856 | -65.551 | 29.4481 | 18.5811 | 26.2651 | -73.4481 |
| 386 | -29.1904 | 19 | -5.07741 | -10.0507 | -52.0086 | 31.5844 | 25.7916 | 17.658 | -114.188 |
| 387 | -35.1939 | 21 | -6.79613 | -9.71374 | -52.1617 | 17.9694 | 24.0311 | 15.2226 | -60.4502 |
| 388 | -25.8926 | 29 | -4.90906 | -9.64292 | -51.2053 | 27.2498 | 21.0957 | 16.6238 | -82.0238 |
| 389 | -25.5395 | 17 | -5.29206 | -9.13223 | -46.4625 | 29.9006 | 25.0079 | 17.3858 | -92.3281 |
| 390 | -30.325 | 13 | -4.81096 | -8.37854 | -46.0171 | 17.506 | 20.6444 | 15.4456 | -90.6421 |
| 391 | -37.6006 | 15 | -7.73436 | -9.51451 | -45.6771 | 35.2595 | 24.2578 | 10.3422 | -96.6512 |
| 392 | -24.6073 | 13 | -5.58763 | -6.80168 | -33.3885 | 16.5731 | 22.215 | 4.5664 | -15.4937 |
| 393 | -30.9951 | 15 | -3.7662 | -9.53589 | -48.8347 | 20.6873 | 23.5825 | 10.7125 | -88.4009 |
| 394 | -36.7138 | 19 | -5.90076 | -9.55039 | -50.7052 | 23.033 | 22.8182 | 10.3112 | -123.388 |
| 395 | -36.7364 | 17 | -4.98937 | -10.523 | -56.1871 | 25.4614 | 22.8617 | 17.8452 | -95.4276 |
| 396 | -33.4962 | 17 | -6.68287 | -8.41873 | -51.1979 | 15.1835 | 24.051 | 18.1996 | -81.3264 |
| 397 | -35.8605 | 17 | -7.00316 | -9.25505 | -48.6738 | 31.9167 | 21.2403 | 16.0635 | -50.317 |
| 398 | -27.314 | 19 | -1.19009 | -9.91409 | -49.3425 | 11.0687 | 14.0621 | 14.7504 | -124.845 |
| 399 | -39.114 | 17 | -9.89646 | -8.65427 | -50.4379 | 19.3151 | 23.8046 | 21.3741 | -101.227 |
| 400 | -34.1994 | 17 | -8.53468 | -8.36417 | -37.3196 | 23.6882 | 22.7727 | 4.60213 | -53.1529 |
| 401 | -28.4711 | 15 | -4.08674 | -9.77015 | -52.1481 | 30.177 | 22.323 | 22.4549 | -102.187 |
| 402 | -34.4519 | 17 | -4.78494 | -9.32474 | -53.5056 | 28.7107 | 20.9488 | 17.4174 | -104.855 |
| 403 | -36.4474 | 15 | -5.8416 | -9.88628 | -51.6107 | 40.3542 | 26.2729 | 11.9116 | -120.733 |
| 404 | -33.0217 | 15 | -4.27402 | -10.0474 | -51.6756 | 26.1535 | 24.9245 | 12.8873 | -146.311 |
| 405 | -32.4294 | 15 | -5.52243 | -8.94228 | -49.5273 | 31.6732 | 24.0206 | 15.2205 | -57.8751 |
| 406 | -46.1624 | 19 | -3.38087 | -9.34911 | -69.7036 | 34.9336 | 20.1566 | 17.9414 | -79.9191 |
| 407 | -45.2438 | 19 | -3.50571 | -11.8105 | -69.3548 | 19.4296 | 22.6716 | 18.9238 | -130.812 |
| 408 | -31.8089 | 19 | -2.45341 | -9.73674 | -63.4582 | 37.2677 | 26.1497 | 20.2541 | -107.55 |
| 409 | -32.5348 | 17 | -5.90518 | -9.80748 | -57.4265 | 34.7649 | 30.7895 | 19.8648 | -124.227 |
| 410 | -41.7718 | 17 | -6.96086 | -9.03811 | -58.2095 | 42.2494 | 25.2298 | 16.6392 | -33.8274 |
| 411 | -27.6328 | 13 | -7.82421 | -8.2663 | -41.054 | 21.1174 | 25.6333 | 17.7726 | -113.49 |
| 412 | -38.5956 | 19 | -3.79648 | -9.56378 | -59.3722 | 32.3458 | 22.5778 | 12.8123 | -98.4492 |
| 413 | -33.8021 | 20 | -3.28538 | -10.4349 | -65.3749 | 27.3404 | 25.2792 | 24.1163 | -111.843 |
| 414 | -31.7754 | 21 | -5.345 | -9.57575 | -61.804 | 25.0009 | 28.7653 | 23.929 | -110.12 |
| 415 | -33.6217 | 8 | -8.03685 | -6.82436 | -37.2047 | 12.4002 | 20.7448 | 12.3182 | -92.308 |
| 416 | -31.9641 | 11 | -5.93259 | -7.15355 | -39.855 | 15.1664 | 19.0152 | 10.3512 | -80.547 |
| 417 | -31.0445 | 13 | -4.22138 | -8.00162 | -48.8434 | 10.3865 | 20.6592 | 15.9782 | -67.1014 |
| 418 | -31.4877 | 13 | -3.81473 | -8.40515 | -51.351 | 12.7322 | 22.0673 | 16.357 | -64.8817 |
| 419 | -29.1185 | 14 | -3.99507 | -9.73146 | -50.3302 | 21.3947 | 19.2827 | 22.6929 | -89.3093 |
| 420 | -30.64 | 14 | -4.8594 | -9.10293 | -47.1197 | 15.0436 | 20.5003 | 16.8148 | -97.043 |
| 421 | -36.7109 | 13 | -10.1097 | -8.10788 | -38.1109 | 17.7418 | 19.4841 | 15.1467 | -90.256 |
| 422 | -23.7334 | 12 | -3.90926 | -8.27667 | -41.0827 | 7.54205 | 21.235 | 14.6345 | -63.0479 |
| 423 | -30.4308 | 14 | -4.3175 | -9.27929 | -50.1025 | 9.72299 | 20.484 | 19.7713 | -81.3048 |
| 424 | -25.6076 | 13 | -3.60105 | -7.96926 | -42.6815 | 12.3905 | 20.4419 | 12.7161 | -96.3127 |
| 425 | -36.5849 | 11 | -10.3129 | -7.31432 | -35.4784 | 15.6171 | 18.6941 | 14.0382 | -82.9656 |
| 426 | -34.076 | 11 | -4.14118 | -8.59267 | -46.1188 | 15.1904 | 19.2917 | 10.9215 | -111.166 |
| 427 | -31.576 | 11 | -2.80714 | -8.45522 | -50.7317 | 11.9105 | 18.1693 | 17.7209 | -57.6072 |
| 428 | -34.1245 | 15 | -5.86681 | -8.13203 | -52.8715 | 13.4664 | 23.4017 | 18.92 | -87.7616 |
| 429 | -36.8964 | 15 | -7.52257 | -7.12416 | -48.1013 | 13.8049 | 22.9294 | 13.3532 | -110.824 |
| 430 | -35.865 | 17 | -8.01686 | -9.62823 | -51.0362 | 36.2127 | 25.8819 | 18.6264 | -81.295 |
| 431 | -25.0913 | 17 | -3.78769 | -9.36154 | -49.5335 | 17.7655 | 25.8574 | 16.2185 | -98.3511 |
| 432 | -31.7559 | 17 | -4.39308 | -8.33038 | -52.656 | 23.2699 | 19.366 | 19.5022 | -117.523 |
| 433 | -38.9473 | 17 | -3.67377 | -8.52248 | -59.0996 | 24.6604 | 17.0103 | 18.4838 | -117.976 |
| 434 | -36.0108 | 15 | -7.128 | -8.10741 | -50.216 | 16.1128 | 25.4301 | 14.543 | -116.973 |
| 435 | -36.5078 | 15 | -4.34126 | -8.93283 | -55.5265 | 22.3325 | 20.9316 | 17.1037 | -76.9623 |
| 436 | -34.4971 | 19 | -5.9959 | -7.89167 | -46.8389 | 17.1169 | 22.1554 | 7.24585 | -68.4923 |
| 437 | -36.0885 | 19 | -6.43313 | -9.15162 | -57.3165 | 26.8922 | 25.4422 | 19.9299 | -102.032 |
| 438 | -37.2083 | 20 | -6.15554 | -8.64328 | -57.5906 | 25.623 | 24.4385 | 17.2998 | -74.3663 |
| 439 | -30.3482 | 20 | -7.93487 | -9.04947 | -52.9028 | 42.989 | 29.4789 | 22.0967 | -80.6099 |
| 440 | -32.1244 | 18 | -4.52253 | -8.97059 | -50.5154 | 22.3344 | 21.6104 | 13.3872 | -99.2837 |
| 441 | -29.0045 | 22 | -11.4468 | -8.19155 | -39.3649 | 37.9949 | 30.9899 | 12.677 | -42.3109 |
| 442 | -30.5702 | 15 | -3.36561 | -7.50027 | -49.3592 | 18.4348 | 21.9921 | 10.3564 | -78.3785 |
| 443 | -40.3772 | 17 | -3.56312 | -8.27169 | -52.8466 | 30.591 | 16.3526 | 6.9117 | -42.2984 |
| 444 | -32.5742 | 13 | -5.54669 | -7.35672 | -46.79 | 35.8005 | 23.8458 | 11.354 | -52.6845 |
| 445 | -30.7419 | 17 | -5.32566 | -8.26411 | -44.1946 | 31.0911 | 23.0281 | 7.56435 | -92.2417 |
| 446 | -34.4744 | 13 | -5.90207 | -7.07484 | -46.0531 | 34.8623 | 21.7068 | 10.8651 | -37.3795 |
| 447 | -33.5297 | 17 | -5.77819 | -7.08914 | -45.3494 | 16.8347 | 20.2895 | 8.74416 | -47.4985 |
| 448 | -38.4769 | 13 | -6.00705 | -7.25836 | -49.0888 | 18.46 | 23.9352 | 7.45675 | -47.4566 |
| 449 | -23.046 | 17 | -4.70783 | -7.59109 | -44.0134 | 24.4194 | 24.034 | 14.7557 | -88.4681 |
| 450 | -29.7542 | 15 | -3.67811 | -7.61088 | -48.8956 | 11.3527 | 24.2017 | 9.6894 | -60.7552 |
| 451 | -17.4777 | 15 | -3.5654 | -7.10532 | -42.7976 | 12.2399 | 28.5595 | 13.1536 | -51.8128 |
| 452 | -22.9749 | 12 | -3.67338 | -7.84949 | -36.9248 | 11.4681 | 17.9116 | 11.9228 | -102.726 |
| 453 | -24.5816 | 11 | -3.85791 | -8.84523 | -36.3288 | 20.281 | 19.3326 | 9.59775 | -65.5034 |
| 454 | -26.6832 | 11 | -4.99818 | -8.58133 | -35.5841 | 17.6432 | 18.8083 | 9.9842 | -62.4941 |
| 455 | -28.1572 | 11 | -2.7028 | -8.83748 | -38.385 | 13.5646 | 14.5031 | 8.34806 | -57.1873 |
| 456 | -24.4332 | 11 | -4.9921 | -8.55501 | -37.7456 | 13.4818 | 20.5931 | 14.59 | -89.6496 |
| 457 | -25.3433 | 12 | -5.37604 | -8.32585 | -35.1764 | 16.1725 | 21.4215 | 8.70704 | -71.6512 |
| 458 | -20.2232 | 13 | -3.78487 | -8.58149 | -37.5052 | 10.8227 | 20.8664 | 13.8751 | -65.0548 |
| 459 | -24.0367 | 13 | -4.72211 | -9.12774 | -36.8371 | 7.79637 | 20.5003 | 11.648 | -77.246 |
| 460 | -31.6398 | 11 | -5.61696 | -8.23406 | -36.9996 | 15.2754 | 18.1431 | 7.39425 | -75.6743 |
| 461 | -29.1569 | 9 | -7.86446 | -7.00857 | -31.718 | 8.88255 | 20.27 | 9.92647 | -90.2627 |
| 462 | -32.6659 | 9 | -7.91428 | -7.10496 | -31.4675 | 8.25561 | 19.9116 | 4.92632 | -94.4613 |
| 463 | -27.6053 | 10 | -2.52692 | -7.75758 | -39.7563 | 10.6956 | 17.6397 | 6.83631 | -63.4403 |
| 464 | -30.5128 | 10 | -5.21902 | -7.25101 | -40.8335 | 17.8603 | 18.4246 | 12.9765 | -30.5924 |
| 465 | -30.9676 | 10 | -4.31535 | -7.91468 | -42.9135 | 18.3998 | 18.1889 | 12.9165 | -93.431 |
| 466 | -32.2233 | 10 | -3.38119 | -7.64484 | -43.3611 | 13.1765 | 15.3509 | 11.0623 | -64.7387 |
| 467 | -25.137 | 11 | -1.25217 | -8.40117 | -44.3744 | 8.19836 | 15.7543 | 14.5734 | -101.494 |
| 468 | -42.7388 | 15 | -9.49545 | -9.02513 | -48.1781 | 26.7585 | 20.961 | 16.3467 | -112.114 |
| 469 | -39.3478 | 15 | -9.96803 | -8.43905 | -46.6036 | 19.1862 | 24.5599 | 16.2259 | -103.917 |
| 470 | -26.9337 | 18 | -4.74022 | -8.93417 | -49.2073 | 18.9428 | 25.1401 | 16.0564 | -111.511 |
| 471 | -18.5997 | 14 | -2.21925 | -7.8973 | -42.1443 | 15.3983 | 20.9853 | 15.6235 | -43.6735 |
| 472 | -25.1071 | 13 | -5.89175 | -7.63896 | -39.9867 | 17.9956 | 25.7963 | 11.7523 | -87.296 |
| 473 | -22.9793 | 13 | -4.39583 | -8.47836 | -44.7117 | 18.1361 | 27.9566 | 14.7983 | -100.915 |
| 474 | -28.1741 | 12 | -6.42436 | -7.79378 | -41.6669 | 17.7887 | 22.5057 | 16.4985 | -93.0628 |
| 475 | -22.3702 | 14 | -3.36781 | -9.29986 | -41.2173 | 14.1713 | 19.9663 | 15.4907 | -71.9603 |
| 476 | -29.9029 | 15 | -1.16864 | -10.531 | -56.9504 | 23.8593 | 19.4725 | 20.3873 | -108.754 |
| 477 | -24.3217 | 15 | -3.51866 | -9.06714 | -39.2372 | 15.8508 | 18.423 | 10.7204 | -98.2802 |
| 478 | -23.2279 | 15 | -3.77079 | -7.376 | -39.4322 | 18.5432 | 19.0934 | 11.1611 | -79.9099 |
| 479 | -25.5678 | 15 | -3.3288 | -8.13736 | -43.6697 | 12.6163 | 18.4462 | 13.8502 | -80.5934 |
| 480 | -28.6275 | 16 | -6.43496 | -8.2381 | -38.5746 | 14.9292 | 18.2104 | 12.8625 | -58.0625 |
| 481 | -28.9356 | 13 | -3.8199 | -8.91765 | -44.937 | 14.0302 | 18.9125 | 14.6306 | -72.5046 |
| 482 | -25.8263 | 15 | -3.58795 | -9.74939 | -45.5464 | 22.0212 | 20.4474 | 16.6579 | -103.841 |
| 483 | -26.0542 | 14 | -5.08068 | -9.41492 | -42.0245 | 17.6093 | 21.831 | 15.6957 | -90.3123 |
| 484 | -28.623 | 15 | -6.28326 | -9.37589 | -41.5334 | 29.1505 | 20.3104 | 16.4487 | -95.4576 |
| 485 | -24.5794 | 14 | -1.38956 | -9.46018 | -49.9022 | 18.0082 | 19.9386 | 17.9132 | -91.168 |
| 486 | -18.4906 | 14 | -2.49918 | -10.4214 | -41.9116 | 20.7644 | 21.4824 | 18.5067 | -90.5103 |
| 487 | -27.4509 | 12 | -3.92796 | -8.44203 | -46.4087 | 16.7737 | 22.532 | 15.8374 | -103.283 |
| 488 | -15.057 | 14 | -2.32257 | -9.28931 | -33.3143 | 14.0478 | 19.7966 | 10.7725 | -50.2591 |
| 489 | -23.9922 | 14 | -5.69822 | -8.76067 | -40.5788 | 20.6593 | 22.6555 | 17.3956 | -93.9377 |
| 490 | -24.0871 | 14 | -2.54471 | -9.17479 | -39.8988 | 24.1401 | 19.0384 | 8.66373 | -94.0522 |
| 491 | -22.7616 | 12 | -3.96077 | -8.81684 | -39.2537 | 15.9546 | 21.9734 | 13.2425 | -58.1944 |
| 492 | -29.289 | 12 | -3.82876 | -10.3729 | -46.6062 | 19.0379 | 22.5761 | 14.8814 | -86.2508 |
| 493 | -27.5387 | 13 | -5.04517 | -7.56948 | -43.328 | 27.5174 | 20.8317 | 15.4566 | -59.5062 |
| 494 | -22.3838 | 13 | -5.39645 | -8.0712 | -36.9567 | 17.9683 | 20.2461 | 16.1348 | -77.9647 |
| 495 | -27.0323 | 20 | -6.48492 | -9.728 | -42.6497 | 25.1861 | 24.722 | 12.1221 | -109.242 |
| 496 | -28.5046 | 14 | -4.69922 | -9.4762 | -43.0308 | 26.2019 | 20.0948 | 14.0805 | -65.8997 |
| 497 | -20.3731 | 6 | -2.72526 | -5.63819 | -29.4385 | 12.4567 | 14.0073 | 8.53397 | -26.9573 |
| 498 | -21.2434 | 14 | -5.03155 | -9.19649 | -36.1588 | 33.3899 | 20.5764 | 15.113 | -62.2158 |
| 499 | -21.4611 | 10 | -3.22426 | -7.84543 | -34.9267 | 5.86759 | 18.2629 | 10.8822 | -96.0968 |
| 500 | -30.9313 | 10 | -7.20419 | -7.79894 | -37.8801 | 17.8486 | 19.7594 | 14.5167 | -84.7918 |
| 501 | -32.9912 | 10 | -7.7184 | -6.77435 | -44.6868 | 19.9348 | 24.7817 | 16.9327 | -108.361 |
| 502 | -30.8013 | 10 | -6.47672 | -6.71932 | -44.6382 | 15.5503 | 22.1811 | 18.3168 | -108.002 |
| 503 | -29.3088 | 15 | -7.87758 | -7.68042 | -37.9182 | 14.294 | 19.3254 | 15.4417 | -114.424 |
| 504 | -22.805 | 14 | -7.28431 | -7.71177 | -32.1059 | 14.0344 | 19.0661 | 15.4508 | -78.4942 |
| 505 | -19.7125 | 11 | -2.45542 | -8.16283 | -34.9861 | 10.803 | 14.6523 | 14.1904 | -109.913 |
| 506 | -16.8239 | 18 | -5.99847 | -7.06397 | -34.0908 | 14.7894 | 26.3636 | 10.005 | -71.1888 |
| 507 | -24.2802 | 10 | -2.49562 | -8.01553 | -39.3956 | 13.0341 | 14.9966 | 14.4731 | -111.567 |
| 508 | -23.5552 | 11 | -2.50131 | -8.42715 | -38.6221 | 12.5272 | 14.9158 | 14.022 | -107.554 |
| 509 | -23.9375 | 20 | -8.60954 | -8.34064 | -37.2289 | 13.5823 | 25.9081 | 13.9859 | -98.273 |
| 510 | -20.2273 | 10 | -2.61026 | -7.52771 | -36.9284 | 14.8598 | 18.0737 | 13.3233 | -92.817 |
| 511 | -10.7571 | 18 | -3.64399 | -8.55906 | -38.4235 | 15.7698 | 21.7839 | 23.458 | -91.4619 |
| 512 | -19.2175 | 19 | -5.70333 | -7.89915 | -39.1765 | 12.2263 | 25.0636 | 14.3683 | -95.3644 |
| 513 | -23.3074 | 10 | -2.49122 | -7.83898 | -38.2381 | 12.6026 | 14.6808 | 14.3559 | -111.048 |
| 514 | -13.9816 | 18 | -4.21587 | -7.71505 | -37.7207 | 12.4569 | 25.3203 | 14.8245 | -78.7652 |
| 515 | -21.7728 | 18 | -7.80931 | -6.85161 | -36.7894 | 15.0189 | 26.1723 | 13.5388 | -64.4188 |
| 516 | -18.0132 | 10 | -2.47297 | -7.81952 | -34.7602 | 12.5433 | 16.4432 | 15.0156 | -110.816 |
| 517 | -20.1507 | 11 | -2.52983 | -7.93468 | -35.2006 | 11.3778 | 14.4765 | 14.1048 | -103.84 |
| 518 | -18.4226 | 14 | -2.47486 | -8.95263 | -38.1214 | 20.176 | 17.1538 | 16.2017 | -114.104 |
| 519 | -19.3801 | 11 | -2.5392 | -8.37704 | -34.895 | 13.2848 | 15.1353 | 14.5459 | -101.326 |
| 520 | -24.2116 | 10 | -2.36097 | -7.15294 | -37.6592 | 12.5767 | 14.2036 | 11.4555 | -75.0295 |
| 521 | -22.2597 | 11 | -2.6546 | -7.81871 | -41.4409 | 18.0014 | 18.5014 | 16.1512 | -77.4836 |
| 522 | -24.8439 | 12 | -2.68105 | -7.89735 | -44.2827 | 20.3036 | 19.7598 | 14.3837 | -86.197 |
| 523 | -25.7679 | 14 | -4.6893 | -7.93328 | -44.2951 | 27.1772 | 24.7792 | 13.2151 | -87.3508 |
| 524 | -18.5868 | 14 | -2.55776 | -9.05104 | -37.6319 | 12.0667 | 16.9429 | 15.8686 | -106.444 |
| 525 | -22.929 | 18 | -7.11164 | -7.25401 | -39.2734 | 14.2587 | 24.0587 | 15.6864 | -96.2938 |
| 526 | -18.8455 | 10 | -2.47971 | -7.84652 | -33.4539 | 10.638 | 14.3087 | 14.2541 | -106.978 |
| 527 | -17.654 | 12 | -2.5603 | -8.7403 | -37.6353 | 25.5225 | 17.9598 | 17.6439 | -107.002 |
| 528 | -18.4136 | 16 | -4.82561 | -7.20818 | -38.2527 | 16.0209 | 22.1871 | 16.1168 | -90.0879 |
| 529 | -22.5904 | 18 | -5.04237 | -8.39074 | -43.8395 | 20.6893 | 22.924 | 17.6289 | -79.9616 |
| 530 | -23.846 | 10 | -3.36438 | -8.38054 | -43.3519 | 20.8687 | 20.5176 | 18.5524 | -54.99 |
| 531 | -15.6736 | 18 | -3.66305 | -7.80905 | -39.9963 | 12.8124 | 23.1616 | 16.1379 | -90.3149 |
| 532 | -18.3551 | 16 | -2.67887 | -8.74784 | -38.7662 | 12.1562 | 19.0702 | 13.8592 | -86.628 |
| 533 | -23.7087 | 14 | -5.79128 | -8.10603 | -32.9432 | 16.9476 | 19.6475 | 9.36639 | -89.3456 |
| 534 | -30.4211 | 22 | -11.7492 | -8.83902 | -37.6593 | 22.6902 | 24.3451 | 17.3136 | -103.052 |
| 535 | -22.8046 | 14 | -2.52316 | -9.08719 | -43.2706 | 20.93 | 18.2177 | 16.4767 | -106.609 |
| 536 | -17.4158 | 20 | -3.8901 | -9.77172 | -45.9815 | 31.9398 | 25.9323 | 20.4628 | -1.91329 |
| 537 | -22.5543 | 16 | -5.953 | -7.62652 | -36.0811 | 11.8327 | 20.8031 | 12.8717 | -103.199 |
| 538 | -15.7069 | 18 | -6.0534 | -9.00909 | -37.3037 | 19.5451 | 26.9467 | 18.0877 | -82.8163 |
| 539 | -17.3166 | 10 | -4.19565 | -7.14409 | -32.6646 | 11.8068 | 19.2537 | 15.62 | -80.802 |
| 540 | -18.7482 | 14 | -5.49663 | -6.52212 | -32.4726 | 12.6058 | 20.8142 | 12.1061 | -70.2981 |
| 541 | -18.0146 | 16 | -2.58433 | -7.72796 | -40.6086 | 12.2876 | 20.0877 | 14.6127 | -101.169 |
| 542 | -22.5706 | 12 | -2.59645 | -7.87961 | -41.0072 | 15.3462 | 18.413 | 14.0535 | -113.006 |
| 543 | -12.6995 | 18 | -2.87165 | -9.12745 | -38.8102 | 13.5986 | 22.7807 | 17.5846 | -86.1433 |
| 544 | -19.5455 | 18 | -4.54767 | -10.2711 | -36.5081 | 19.1879 | 18.1383 | 16.5849 | -78.0284 |
| 545 | -20.8387 | 14 | -3.23572 | -8.7566 | -46.8169 | 21.375 | 27.3578 | 16.8381 | -124.747 |
| 546 | -28.49 | 12 | -3.60441 | -9.50515 | -43.5471 | 20.5503 | 17.6948 | 15.263 | -106.711 |
| 547 | -20.2151 | 24 | -5.15276 | -9.06301 | -41.5109 | 21.5739 | 20.4542 | 16.1572 | -94.1584 |
| 548 | -17.341 | 12 | -3.27834 | -7.31711 | -31.6586 | 12.0351 | 17.3464 | 11.0674 | -88.6829 |
| 549 | -25.8697 | 18 | -6.30571 | -7.9865 | -44.9403 | 28.4589 | 25.2359 | 16.1335 | -98.6633 |
| 550 | -16.7859 | 14 | -2.29404 | -8.03848 | -35.9559 | 12.3253 | 16.4954 | 14.5274 | -109.868 |
| 551 | -27.7294 | 16 | -2.58613 | -7.90113 | -44.5604 | 19.2018 | 15.6972 | 11.0527 | -73.749 |
| 552 | -18.84 | 18 | -4.07242 | -8.96991 | -43.5222 | 24.8784 | 23.6929 | 18.8267 | -101.121 |
| 553 | -33.066 | 10 | -1.70501 | -8.39413 | -45.2629 | 18.2663 | 14.8357 | 7.58766 | -56.9096 |
| 554 | -23.9336 | 18 | -6.87167 | -6.69782 | -39.5572 | 17.9248 | 24.3528 | 12.7754 | -97.9421 |
| 555 | -22.0723 | 18 | -5.4026 | -7.73914 | -40.8701 | 16.3164 | 26.1899 | 10.9228 | -104.734 |
| 556 | -25.2181 | 10 | -4.06823 | -8.41122 | -37.8641 | 10.5172 | 18.6932 | 12.9609 | -110.544 |
| 557 | -19.402 | 12 | -3.05528 | -7.99514 | -40.4976 | 18.3821 | 21.9409 | 15.9492 | -94.6099 |
| 558 | -22.0267 | 14 | -3.94156 | -8.06775 | -36.9442 | 12.5184 | 18.2428 | 12.4438 | -100.304 |
| 559 | -23.5719 | 12 | -4.09208 | -8.87386 | -41.8558 | 14.2328 | 21.3474 | 17.23 | -108.551 |
| 560 | -28.3491 | 18 | -7.96578 | -8.40628 | -39.0325 | 19.2813 | 20.6129 | 15.4687 | -91.4305 |
| 561 | -16.1257 | 18 | -5.6066 | -9.47051 | -34.423 | 30.6144 | 24.8363 | 14.2425 | -78.7493 |
| 562 | -11.6146 | 14 | -5.09849 | -8.13666 | -26.1857 | 11.3557 | 21.0666 | 13.2149 | -84.5513 |
| 563 | -22.3499 | 12 | -5.11556 | -6.12309 | -34.3475 | 9.31162 | 19.0381 | 11.4603 | -76.8851 |
| 564 | -30.9966 | 14 | -7.66882 | -8.31392 | -41.9556 | 31.1346 | 22.6892 | 15.931 | -60.7693 |
| 565 | -24.2755 | 10 | -2.36625 | -7.14862 | -37.6716 | 12.6827 | 14.214 | 11.3815 | -74.8404 |
| 566 | -23.9692 | 10 | -2.6561 | -8.7308 | -42.6758 | 25.9852 | 20.3189 | 15.2076 | -118.925 |
| 567 | -40.3046 | 10 | -4.88468 | -8.13299 | -52.8641 | 28.4588 | 24.2013 | 9.41792 | -120.061 |
| 568 | -30.0288 | 10 | -8.1384 | -6.54186 | -43.7937 | 21.0136 | 27.3456 | 18.5286 | -115.718 |
| 569 | -28.3493 | 10 | -5.51797 | -7.44615 | -43.744 | 30.444 | 24.1117 | 15.5459 | -130.147 |
| 570 | -36.1278 | 12 | -5.11363 | -8.55042 | -49.4533 | 27.4156 | 21.5983 | 13.0197 | -79.0773 |
| 571 | -27.2774 | 10 | -1.70621 | -9.13075 | -57.7142 | 34.934 | 28.1385 | 20.8595 | -116.024 |
| 572 | -40.2909 | 10 | -5.54494 | -8.21415 | -50.3404 | 27.3005 | 21.4255 | 11.3827 | -49.1761 |
| 573 | -24.6649 | 10 | -3.54944 | -7.12439 | -42.3445 | 21.7114 | 21.222 | 14.4189 | -95.2213 |
| 574 | -27.2246 | 14 | -6.33418 | -6.7336 | -42.3785 | 16.5762 | 24.2796 | 13.7593 | -88.0377 |
| 575 | -20.5524 | 8 | -4.50655 | -6.14447 | -37.37 | 10.7918 | 20.7241 | 18.1712 | -105.784 |
| 576 | -28.0708 | 11 | -6.88451 | -6.39203 | -44.0198 | 16.3442 | 25.0875 | 18.5361 | -94.4346 |
| 577 | -31.2809 | 10 | -5.08748 | -7.22699 | -43.3774 | 16.4186 | 20.903 | 12.3223 | -104.056 |
| 578 | -26.107 | 13 | -3.52828 | -8.35789 | -43.9453 | 19.1563 | 19.2668 | 15.3251 | -103.929 |
| 579 | -24.5477 | 10 | -4.75959 | -6.51824 | -43.864 | 18.178 | 25.5616 | 15.9909 | -102.886 |
| 580 | -26.3983 | 12 | -5.99856 | -7.66844 | -43.2944 | 25 | 24.9609 | 17.1055 | -103.621 |
| 581 | -28.1663 | 10 | -4.29685 | -7.71898 | -42.8237 | 25.8271 | 20.3112 | 14.3436 | -121.388 |
| 582 | -24.9166 | 14 | -4.60546 | -7.5021 | -43.5351 | 14.0529 | 19.9634 | 18.0669 | -89.9549 |
| 583 | -31.1046 | 14 | -4.50594 | -8.5097 | -46.3103 | 24.3567 | 21.9755 | 11.2508 | -93.9969 |
| 584 | -24.3591 | 16 | -6.4859 | -7.39521 | -44.1857 | 26.7356 | 28.785 | 15.1545 | -78.7218 |
| 585 | -23.5109 | 18 | -4.47984 | -8.33538 | -47.989 | 26.1136 | 26.0146 | 16.7968 | -116.523 |
| 586 | -27.5167 | 14 | -3.64077 | -8.5129 | -45.1737 | 18.4825 | 21.3661 | 12.3443 | -121.622 |
| 587 | -29.7072 | 10 | -3.07837 | -7.9986 | -48.142 | 18.4311 | 21.8805 | 13.8966 | -112.15 |
| 588 | -30.4483 | 10 | -7.46105 | -7.12528 | -43.3552 | 18.5713 | 26.8287 | 15.8118 | -99.5991 |
| 589 | -35.1081 | 10 | -5.50808 | -8.05672 | -47.7474 | 22.0223 | 22.7243 | 13.5295 | -81.9547 |
| 590 | -28.4259 | 10 | -4.32082 | -8.0882 | -48.3895 | 18.9326 | 21.4632 | 21.5409 | -70.4141 |
| 591 | -30.2998 | 10 | -7.33014 | -6.36866 | -43.2279 | 19.1494 | 28.052 | 13.184 | -96.1345 |
| 592 | -30.1918 | 10 | -3.51771 | -7.87577 | -44.9103 | 16.1536 | 19.24 | 12.826 | -125.323 |
| 593 | -32.4254 | 10 | -5.08177 | -7.1783 | -46.0302 | 21.8259 | 24.2493 | 10.7336 | -99.2273 |
| 594 | -18.5915 | 6 | -2.50537 | -6.22392 | -29.547 | 7.9532 | 13.7797 | 11.4081 | -74.6841 |
| 595 | -19.4696 | 18 | -5.18617 | -7.98269 | -41.4937 | 19.3628 | 23.8792 | 17.8547 | -83.397 |
| 596 | -23.1152 | 10 | -2.02158 | -7.11263 | -37.5413 | 16.5218 | 14.1659 | 11.639 | -72.0332 |
| 597 | -19.9755 | 8 | -2.01584 | -6.85363 | -32.5922 | 12.1269 | 13.913 | 10.7182 | -70.9409 |
| 598 | -22.0216 | 10 | -2.49503 | -7.93252 | -40.5546 | 13.0739 | 18.7365 | 15.3151 | -110.454 |
| 599 | -21.217 | 10 | -2.72465 | -7.91097 | -39.1372 | 15.0005 | 17.8823 | 16.2155 | -112.887 |
| 600 | -21.1491 | 10 | -2.49812 | -7.30937 | -38.375 | 18.0908 | 16.6947 | 15.0339 | -104.295 |
| 601 | -25.2007 | 10 | -3.46796 | -8.02879 | -40.9661 | 18.0569 | 19.9197 | 13.6045 | -105.242 |
| 602 | -21.8846 | 10 | -0.53362 | -7.87485 | -39.8431 | 11.0294 | 14.5087 | 11.6715 | -60.3323 |
| 603 | -23.6829 | 12 | -3.38923 | -7.78704 | -40.0246 | 15.8838 | 17.9831 | 14.3128 | -107.058 |
| 604 | -28.7148 | 10 | -3.41079 | -7.96463 | -43.9896 | 16.7191 | 17.5652 | 15.2524 | -113.562 |
| 605 | -28.4008 | 10 | -4.71425 | -8.45004 | -39.8528 | 19.4382 | 16.5984 | 16.0481 | -118.129 |
| 606 | -24.4807 | 10 | -2.4796 | -8.05805 | -42.0788 | 15.8479 | 18.6771 | 14.0341 | -111.041 |
| 607 | -27.0027 | 10 | -2.80789 | -7.70816 | -43.2089 | 19.0614 | 16.2868 | 15.5447 | -106.062 |
| 608 | -23.3121 | 11 | -2.46265 | -7.90198 | -44.1688 | 21.4405 | 20.2006 | 16.1078 | -60.6198 |
| 609 | -21.6801 | 11 | -0.9501 | -8.1048 | -41.4942 | 9.87375 | 14.7947 | 15.0789 | -99.6264 |
| 610 | -18.6398 | 10 | -3.7599 | -8.13795 | -34.0674 | 13.6763 | 20.4827 | 13.6827 | -73.8628 |
| 611 | -23.8212 | 10 | -2.48922 | -8.4509 | -42.2039 | 16.4927 | 19.6803 | 14.5185 | -111.382 |
| 612 | -27.3591 | 10 | -5.6436 | -7.51566 | -41.7525 | 16.8762 | 22.3173 | 16.62 | -80.913 |
| 613 | -27.7539 | 10 | -2.67651 | -8.07625 | -44.6423 | 13.1656 | 17.3529 | 15.237 | -92.5169 |
| 614 | -35.3182 | 14 | -9.26042 | -7.67304 | -41.3261 | 24.9867 | 23.1267 | 13.3829 | -112.816 |
| 615 | -24.2975 | 10 | -2.73209 | -7.87954 | -42.3106 | 20.7979 | 18.2512 | 15.9336 | -97.2131 |
| 616 | -22.6661 | 11 | -2.49175 | -8.44539 | -39.1884 | 14.8046 | 16.7943 | 14.0745 | -115.9 |
| 617 | -23.1036 | 10 | -4.52551 | -7.45074 | -35.8098 | 22.6231 | 19.8693 | 12.4935 | -115.858 |
| 618 | -21.8203 | 10 | -2.56821 | -8.08916 | -40.7922 | 15.798 | 17.9537 | 17.309 | -99.5221 |
| 619 | -21.3916 | 9 | -0.92692 | -8.07306 | -41.3115 | 9.37675 | 14.3618 | 17.4282 | -101.132 |
| 620 | -25.1003 | 10 | -3.40903 | -7.78958 | -42.3309 | 16.7841 | 18.5666 | 16.8932 | -108.005 |
| 621 | -21.646 | 6 | -7.21416 | -7.15645 | -30.944 | 16.6143 | 21.6623 | 18.9363 | -85.8616 |
| 622 | -17.1931 | 5 | -2.15593 | -6.51514 | -33.9884 | 9.45933 | 17.7846 | 15.5705 | -48.6481 |
| 623 | -18.1012 | 6 | -2.43252 | -6.85117 | -33.9532 | 6.4087 | 17.1128 | 15.397 | -84.8315 |
| 624 | -19.1077 | 6 | -3.75905 | -6.99838 | -35.8829 | 13.6582 | 21.9243 | 16.5624 | -64.6763 |
| 625 | -22.9831 | 7 | -3.56778 | -7.87076 | -36.4137 | 13.5861 | 20.1808 | 12.7195 | -58.1602 |
| 626 | -22.0159 | 8 | -2.33507 | -8.37735 | -42.7908 | 37.0365 | 20.5979 | 18.253 | -70.4395 |
| 627 | -24.9068 | 8 | -3.95013 | -8.33284 | -39.7674 | 14.1274 | 21.9241 | 13.9308 | -97.7833 |
| 628 | -24.8733 | 8 | -3.67522 | -8.33842 | -40.7249 | 14.3087 | 21.841 | 14.4786 | -91.5827 |
| 629 | -24.7563 | 8 | -3.66832 | -8.23866 | -40.5293 | 15.813 | 22.1142 | 13.9215 | -89.6463 |
| 630 | -21.1161 | 8 | -2.2327 | -7.78508 | -38.8871 | 31.331 | 20.1695 | 13.1948 | -59.0242 |
| 631 | -20.609 | 8 | -1.82326 | -7.95214 | -38.3076 | 32.2251 | 20.4418 | 11.3758 | -66.101 |
| 632 | -21.9177 | 8 | -0.43106 | -8.08498 | -44.6339 | 32.6774 | 19.2576 | 15.122 | -60.8134 |
| 633 | -23.946 | 7 | -4.11841 | -7.26351 | -41.4131 | 10.4168 | 22.313 | 17.9068 | -87.4733 |
| 634 | -25.9214 | 7 | -5.41231 | -7.26644 | -37.988 | 20.6418 | 22.594 | 14.3678 | -72.4819 |
| 635 | -21.8711 | 7 | -3.8241 | -7.02158 | -41.039 | 10.5585 | 23.6468 | 17.5943 | -93.9406 |
| 636 | -24.1279 | 8 | -4.86122 | -9.32111 | -39.0074 | 28.873 | 21.9603 | 18.4142 | -99.0627 |
| 637 | -25.3529 | 8 | -3.37658 | -8.72619 | -42.3016 | 14.1607 | 20.4828 | 16.9332 | -79.0207 |
| 638 | -15.5537 | 8 | -0.39076 | -7.16134 | -39.9629 | 22.868 | 21.1903 | 14.3988 | -95.3023 |
| 639 | -18.2506 | 8 | -1.81681 | -7.99354 | -38.9762 | 32.5676 | 22.4053 | 13.7482 | -57.1504 |
| 640 | -21.1703 | 9 | -3.3052 | -7.08401 | -42.2004 | 12.0571 | 23.2346 | 17.1404 | -84.514 |
| 641 | -22.4382 | 9 | -3.44502 | -8.8795 | -42.7097 | 16.8748 | 25.0247 | 16.3155 | -100.792 |
| 642 | -21.6352 | 6 | -3.40574 | -7.76851 | -35.3135 | 7.23047 | 18.0793 | 15.6669 | -58.0608 |
| 643 | -16.4509 | 8 | -0.31321 | -8.76866 | -41.4682 | 31.3068 | 18.1264 | 20.1362 | -61.8194 |
| 644 | -26.6004 | 8 | -5.30938 | -9.10929 | -40.8779 | 24.1424 | 20.8868 | 20.2249 | -35.7606 |
| 645 | -25.7486 | 8 | -3.66635 | -8.47219 | -39.0928 | 14.3365 | 19.6544 | 13.2662 | -91.3073 |
| 646 | -24.4789 | 8 | -3.66166 | -7.56947 | -42.3473 | 15.4463 | 21.9611 | 16.5653 | -94.5302 |
| 647 | -16.901 | 8 | -2.01331 | -6.20583 | -36.0888 | 42.4703 | 20.1569 | 12.9118 | -89.1954 |
| 648 | -24.0571 | 8 | -3.7878 | -7.87919 | -41.7903 | 16.368 | 22.9642 | 16.0185 | -93.1722 |
| 649 | -23.8283 | 8 | -1.07617 | -8.88981 | -45.4175 | 28.845 | 20.4003 | 15.4057 | -76.3306 |
| 650 | -21.7781 | 9 | -5.13014 | -7.53196 | -37.7196 | 37.4501 | 22.1645 | 18.0968 | -69.4689 |
| 651 | -14.6045 | 10 | -1.7199 | -7.20456 | -36.3722 | 12.2646 | 22.6126 | 12.1009 | -78.0719 |
| 652 | -14.6859 | 10 | -0.58329 | -8.4335 | -37.9363 | 35.4091 | 17.8188 | 16.6857 | -51.7472 |
| 653 | -18.6849 | 8 | -4.11217 | -8.01696 | -35.7733 | 37.8485 | 21.1971 | 18.4329 | -68.5899 |
| 654 | -20.0679 | 9 | -5.08792 | -7.56066 | -36.6787 | 27.1009 | 22.5637 | 18.5243 | -80.7656 |
| 655 | -16.5759 | 8 | -0.65425 | -7.74733 | -41.5593 | 59.5113 | 19.7463 | 18.5147 | -55.0565 |
| 656 | -18.9449 | 9 | -3.86411 | -8.21486 | -37.809 | 39.3988 | 22.5547 | 17.9216 | -65.4932 |
| 657 | -27.6661 | 9 | -3.17657 | -7.03341 | -35.4382 | 18.7989 | 15.0239 | 5.8383 | -45.6596 |
| 658 | -22.994 | 8 | -1.66641 | -7.23582 | -42.7413 | 24.0525 | 19.6769 | 14.0207 | -126.348 |
| 659 | -20.686 | 7 | -3.46731 | -7.86997 | -37.0986 | 13.5187 | 20.2332 | 16.7922 | -75.7683 |
| 660 | -31.4785 | 16 | -5.18608 | -6.9385 | -45.9209 | 21.2469 | 17.4745 | 14.4031 | -86.371 |
| 661 | -31.7659 | 19 | -8.22536 | -7.51057 | -44.6683 | 20.3124 | 25.0191 | 13.0005 | -115.664 |
| 662 | -30.1934 | 18 | -8.36575 | -7.24908 | -41.14 | 15.9918 | 25.3201 | 10.877 | -86.518 |
| 663 | -21.4126 | 21 | -4.94577 | -7.97761 | -44.3739 | 14.3869 | 22.8088 | 16.8371 | -79.8873 |
| 664 | -24.2255 | 20 | -5.91508 | -6.59365 | -40.7757 | 11.6976 | 24.1757 | 8.78995 | -78.6852 |
| 665 | -28.3145 | 19 | -6.51167 | -7.71392 | -45.0309 | 23.8438 | 24.16 | 13.3927 | -109.469 |
| 666 | -30.1055 | 19 | -6.85743 | -7.75011 | -43.1785 | 14.6909 | 24.0181 | 9.39588 | -101.69 |
| 667 | -22.7747 | 19 | -2.67373 | -8.14035 | -48.152 | 17.1773 | 21.0641 | 15.7543 | -102.346 |
| 668 | -27.1746 | 19 | -8.05339 | -8.603 | -41.283 | 23.3893 | 24.8327 | 15.4894 | -78.4078 |
| 669 | -25.4189 | 20 | -4.21884 | -8.594 | -46.2865 | 16.9936 | 23.3144 | 11.8385 | -99.2624 |
| 670 | -22.443 | 19 | -6.3727 | -8.03393 | -37.8153 | 27.8205 | 23.7906 | 11.5682 | -71.3216 |
| 671 | -20.6913 | 19 | -3.69827 | -8.25179 | -50.4018 | 22.1633 | 26.2363 | 20.4827 | -95.4566 |
| 672 | -23.605 | 19 | -3.68543 | -8.75292 | -52.1358 | 23.7091 | 25.3083 | 20.2094 | -105.771 |
| 673 | -26.9948 | 19 | -7.61439 | -7.60679 | -39.5618 | 17.1628 | 24.2678 | 11.1017 | -55.9088 |
| 674 | -29.7927 | 19 | -6.4431 | -8.16958 | -46.7357 | 19.7234 | 22.3464 | 15.9967 | -83.5224 |
| 675 | -33.6613 | 16 | -8.28961 | -7.25124 | -43.4348 | 19.9999 | 25.0094 | 10.981 | -105.502 |
| 676 | -31.1111 | 19 | -7.62648 | -7.32588 | -43.9917 | 9.25505 | 23.4003 | 12.3238 | -109.947 |
| 677 | -31.2973 | 18 | -8.49412 | -8.49991 | -43.8264 | 13.5467 | 23.3133 | 17.317 | -107.218 |
| 678 | -27.6589 | 21 | -3.7408 | -9.44373 | -50.3388 | 17.7471 | 20.6359 | 15.7493 | -85.4653 |
| 679 | -26.2871 | 2 | -4.4981 | -5.06968 | -30.7182 | 7.32323 | 16.7501 | 8.25065 | -63.5472 |
| 680 | -27.2218 | 2 | -5.39815 | -4.62651 | -28.5804 | 5.7292 | 12.4885 | 11.4383 | -71.0823 |
| 681 | -38.343 | 3 | -6.78199 | -6.32717 | -38.2547 | 12.1423 | 19.4045 | 7.50898 | -84.5364 |
| 682 | -46.963 | 6 | -7.96849 | -8.7275 | -52.9931 | 27.8601 | 29.7012 | 9.33614 | -137.783 |
| 683 | -24.4835 | 2 | -1.58017 | -6.06754 | -35.4537 | 3.57101 | 12.8157 | 12.4568 | -84.752 |
| 684 | -17.3143 | 1 | -2.58196 | -4.08815 | -22.2241 | 2.06834 | 11.5052 | 7.50351 | -36.8987 |
| 685 | -26.297 | 11 | -3.52191 | -6.3641 | -37.9794 | 19.6145 | 17.5449 | 7.72447 | -87.9342 |
| 686 | -20.6115 | 11 | -2.27905 | -6.67114 | -33.6622 | 8.47871 | 14.7482 | 8.52301 | -98.6501 |
| 687 | -26.3305 | 11 | -2.60858 | -7.26411 | -38.9075 | 22.4809 | 15.4834 | 8.83504 | -90.1544 |
| 688 | -25.9181 | 11 | -2.88894 | -6.98638 | -42.693 | 15.7548 | 18.1434 | 12.9653 | -84.1463 |
| 689 | -29.9391 | 11 | -5.79944 | -7.24369 | -40.1878 | 13.111 | 18.8681 | 13.672 | -85.8775 |
| 690 | -29.4151 | 22 | -8.53511 | -7.04952 | -42.9357 | 24.329 | 22.3753 | 14.9335 | -44.8602 |
| 691 | -33.7142 | 6 | -7.94387 | -6.38236 | -36.2287 | 14.4549 | 18.9009 | 13.8075 | -69.9082 |
| 692 | -16.8251 | 2 | -3.20719 | -4.97387 | -25.2026 | 2.94012 | 13.8646 | 12.4632 | -37.9273 |
| 693 | -21.8798 | 3 | -3.2925 | -5.07784 | -25.1535 | 5.72038 | 13.3072 | 4.88706 | -35.8741 |
| 694 | -20.3753 | 4 | -3.31524 | -5.6048 | -26.842 | 5.63462 | 13.9679 | 8.68727 | -46.2084 |
| 695 | -20.5883 | 3 | -3.44242 | -5.42646 | -25.1306 | 4.50432 | 13.6697 | 7.32588 | -44.7282 |
| 696 | -15.2017 | 3 | -1.04418 | -6.57212 | -26.1818 | 8.58744 | 12.6221 | 10.2404 | -67.8611 |
| 697 | -14.4882 | 5 | -2.15798 | -6.06902 | -25.9842 | 2.87747 | 14.7364 | 10.56 | -50.0714 |
| 698 | -12.1014 | 4 | -1.36288 | -6.18946 | -19.317 | 6.86658 | 10.8695 | 6.45226 | -28.02 |
| 699 | -18.7536 | 2 | -3.47776 | -4.7791 | -24.5299 | 4.13536 | 13.1042 | 10.2256 | -51.1351 |
| 700 | -23.296 | 3 | -2.72816 | -5.57571 | -27.8826 | 7.59201 | 9.80082 | 9.20295 | -44.7658 |
| 701 | -17.4854 | 4 | -1.57878 | -5.52735 | -25.6209 | 5.07241 | 11.0276 | 7.8218 | -49.3752 |
| 702 | -18.2865 | 13 | -5.15653 | -8.24276 | -37.3943 | 36.038 | 25.9632 | 15.7636 | -83.3669 |
| 703 | -24.2066 | 13 | -5.36576 | -9.34972 | -43.4535 | 31.5377 | 24.032 | 20.0949 | -78.0894 |
| 704 | -30.2783 | 13 | -4.57989 | -9.99364 | -51.439 | 25.0807 | 23.1984 | 21.5829 | -95.7698 |
| 705 | -23.7107 | 13 | -3.63124 | -9.25737 | -42.5916 | 29.351 | 24.3231 | 12.4667 | -72.9011 |
| 706 | -22.9074 | 9 | -6.81249 | -7.6183 | -28.9036 | 13.8396 | 20.9626 | 10.9304 | -88.8133 |
| 707 | -26.445 | 9 | -4.78204 | -9.38352 | -37.5072 | 21.4843 | 21.2458 | 12.293 | -87.8529 |
| 708 | -28.7919 | 9 | -6.05468 | -6.47818 | -39.2807 | 14.7741 | 22.5574 | 11.8628 | -36.5716 |
| 709 | -24.578 | 13 | -3.99658 | -8.16803 | -44.2443 | 23.3399 | 26.0002 | 12.0455 | -86.0575 |
| 710 | -40.6853 | 13 | -5.60823 | -10.2961 | -56.9635 | 44.3923 | 20.3063 | 21.7208 | -128.5 |
| 711 | -33.6607 | 13 | -4.64642 | -8.95375 | -46.1346 | 21.8126 | 21.0204 | 10.0956 | -124.266 |
| 712 | -18.1052 | 9 | -2.36859 | -8.88544 | -38.9169 | 16.7964 | 21.0816 | 17.4942 | -82.7811 |
| 713 | -17.3289 | 9 | -1.14974 | -7.61625 | -39.3771 | 13.2896 | 18.8917 | 15.8956 | -71.9399 |
| 714 | -24.1649 | 9 | -2.80158 | -8.55094 | -40.132 | 31.2616 | 19.9918 | 12.7126 | -70.2429 |
| 715 | -23.8136 | 13 | -2.19795 | -9.60695 | -52.3392 | 17.6422 | 21.6192 | 25.0132 | -106.476 |
| 716 | -33.3057 | 21 | -7.86629 | -7.92762 | -51.64 | 29.8052 | 30.6552 | 12.0548 | -72.657 |
| 717 | -33.2543 | 18 | -7.20251 | -8.52893 | -48.1108 | 33.2714 | 22.2381 | 17.1434 | -72.7102 |
| 718 | -17.9139 | 18 | -2.79595 | -9.41 | -45.5441 | 20.453 | 23.4347 | 19.1404 | -65.9331 |
| 719 | -29.3717 | 15 | -7.77821 | -7.2613 | -46.8768 | 16.6371 | 28.7088 | 17.4507 | -59.881 |
| 720 | -31.5342 | 15 | -7.86597 | -7.03234 | -49.3286 | 15.599 | 28.3086 | 18.4477 | -61.7315 |
| 721 | -27.6483 | 15 | -5.61344 | -7.82105 | -41.4669 | 14.1654 | 22.5762 | 11.1038 | -81.2092 |
| 722 | -41.7758 | 21 | -5.63603 | -9.85113 | -58.4343 | 25.045 | 21.2733 | 13.5802 | -152.759 |
| 723 | -27.5678 | 21 | -4.17284 | -9.95423 | -50.4903 | 28.2796 | 21.967 | 16.7786 | -89.721 |
| 724 | -31.6027 | 20 | -5.39535 | -9.97765 | -50.835 | 17.2074 | 26.3863 | 11.7845 | -101.782 |
| 725 | -41.1696 | 22 | -5.1714 | -10.4722 | -59.9253 | 44.9961 | 21.6041 | 14.3221 | -112.094 |
| 726 | -41.4139 | 22 | -7.55481 | -9.23167 | -51.9272 | 22.2944 | 22.3234 | 8.89325 | -91.7479 |
| 727 | -28.4456 | 20 | -6.0912 | -9.82658 | -43.0641 | 14.553 | 21.9852 | 12.3094 | -53.1911 |
| 728 | -36.4376 | 21 | -4.54056 | -9.76637 | -57.7152 | 26.9307 | 20.6221 | 17.0334 | -142.357 |
| 729 | -28.9706 | 20 | -2.84965 | -10.4556 | -59.153 | 39.1504 | 26.3545 | 19.1384 | -105.272 |
| 730 | -37.1976 | 22 | -5.08469 | -10.0212 | -58.2186 | 34.8673 | 23.1991 | 15.1516 | -79.7006 |
| 731 | -28.2383 | 22 | -3.99007 | -9.94123 | -50.508 | 25.692 | 19.5846 | 16.8737 | -84.1041 |
| 732 | -33.944 | 21 | -2.27361 | -10.4041 | -62.9289 | 30.8204 | 22.7635 | 18.2353 | -82.3124 |
| 733 | -31.0673 | 21 | -2.09434 | -9.41826 | -65.0954 | 38.1834 | 23.06 | 23.8516 | -65.8012 |
| 734 | -26.5641 | 21 | -2.94458 | -9.75795 | -59.4872 | 27.1742 | 21.8036 | 27.2091 | -124.285 |
| 735 | -43.0954 | 20 | -5.56793 | -8.93468 | -62.5885 | 33.9806 | 24.9416 | 13.4243 | -116.795 |
| 736 | -38.8739 | 22 | -4.68736 | -10.7743 | -64.9127 | 31.9473 | 25.6063 | 19.2604 | -109.321 |
| 737 | -40.4852 | 22 | -4.58328 | -10.5781 | -62.76 | 27.6067 | 22.4274 | 16.5717 | -110.552 |
| 738 | -38.4525 | 21 | -5.9709 | -10.8378 | -61.8361 | 26.0833 | 28.1512 | 18.2394 | -123.511 |
| 739 | -36.1686 | 21 | -5.44372 | -10.1182 | -56.1468 | 22.0762 | 23.562 | 15.5433 | -137.534 |
| 740 | -37.3408 | 22 | -4.96198 | -10.3866 | -60.6434 | 34.7585 | 27.3999 | 13.7357 | -158.347 |
| 741 | -47.8797 | 21 | -4.51652 | -10.3768 | -69.7449 | 33.0823 | 22.8703 | 15.8968 | -119.657 |
| 742 | -45.8567 | 22 | -6.27775 | -9.99517 | -58.7726 | 24.8632 | 18.2943 | 12.9918 | -159.284 |
| 743 | -45.871 | 22 | -5.90656 | -9.24186 | -64.0191 | 32.3165 | 22.7921 | 13.6208 | -94.9619 |
| 744 | -46.603 | 22 | -6.3432 | -9.75047 | -66.2931 | 39.5325 | 26.3905 | 14.0581 | -108.158 |
| 745 | -29.3625 | 22 | -4.59591 | -9.96954 | -58.0921 | 34.9451 | 23.3656 | 24.7161 | -117.367 |
| 746 | -20.8424 | 22 | -2.24714 | -10.1724 | -54.7668 | 26.8546 | 24.2247 | 22.8131 | -101.853 |
| 747 | -42.7634 | 21 | -6.57458 | -9.77774 | -56.3696 | 36.5568 | 20.9131 | 12.8882 | -119.96 |
| 748 | -33.7993 | 24 | -8.13322 | -8.9893 | -51.2523 | 29.0565 | 23.8618 | 17.813 | -54.0401 |
| 749 | -41.3828 | 21 | -5.78219 | -9.57904 | -57.9468 | 25.9473 | 20.8531 | 14.1984 | -154.948 |
| 750 | -22.6018 | 20 | -2.6132 | -8.77202 | -47.3171 | 17.5084 | 18.5428 | 17.1165 | -50.1246 |
| 751 | -24.7307 | 20 | -4.27814 | -9.82793 | -51.4987 | 27.0015 | 22.3619 | 23.341 | -97.7588 |
| 752 | -31.5138 | 21 | -6.92558 | -10.4228 | -54.3245 | 32.6112 | 26.3209 | 22.6895 | -72.8414 |
| 753 | -28.7036 | 21 | -6.99225 | -7.99984 | -48.0053 | 23.5688 | 26.8931 | 14.5184 | -75.2158 |
| 754 | -33.4166 | 22 | -5.24577 | -9.99845 | -57.6796 | 33.1301 | 25.543 | 17.9946 | -104.103 |
| 755 | -32.8947 | 21 | -7.66992 | -9.19716 | -51.3237 | 27.9767 | 24.5139 | 19.7131 | -93.6071 |
| 756 | -25.5171 | 21 | -3.61918 | -9.32016 | -60.3048 | 29.973 | 25.6319 | 27.8254 | -80.9694 |
| 757 | -28.0567 | 21 | -5.95756 | -9.80486 | -53.815 | 34.7436 | 25.2457 | 24.0393 | -114.042 |
| 758 | -30.0665 | 21 | -3.57765 | -10.8013 | -60.8736 | 29.9064 | 25.1044 | 23.738 | -106.829 |
| 759 | -29.5034 | 21 | -3.37886 | -10.241 | -48.2079 | 26.6377 | 16.0934 | 14.3098 | -87.661 |
| 760 | -28.1159 | 22 | -3.42284 | -9.92649 | -53.3217 | 32.885 | 22.2609 | 16.092 | -115.784 |
| 761 | -39.4119 | 20 | -5.56766 | -9.75765 | -56.2128 | 29.5018 | 22.7293 | 12.6937 | -149.236 |
| 762 | -24.8007 | 5 | -2.23924 | -5.33426 | -34.2628 | 5.721 | 13.0752 | 8.93178 | -70.6566 |
| 763 | -22.0819 | 5 | -2.25373 | -5.80855 | -31.8304 | 5.79454 | 12.5765 | 10.4694 | -59.6222 |
| 764 | -23.817 | 5 | -1.99143 | -5.38647 | -34.1297 | 4.60191 | 13.2356 | 9.14127 | -61.2341 |
| 765 | -27.9355 | 6 | -2.36424 | -6.94179 | -40.2093 | 14.4639 | 14.9154 | 12.3027 | -58.6375 |
| 766 | -30.1857 | 7 | -2.1963 | -6.74493 | -41.6748 | 13.5007 | 14.0269 | 10.3707 | -60.9799 |
| 767 | -21.0736 | 5 | -0.97052 | -4.99281 | -32.7792 | 5.57682 | 11.0908 | 9.37584 | -56.8871 |
| 768 | -29.2831 | 5 | -3.87766 | -6.27214 | -35.539 | 8.7527 | 13.6975 | 10.5993 | -57.6597 |
| 769 | -22.966 | 5 | -2.1925 | -5.86385 | -33.0169 | 9.12391 | 12.4923 | 10.8447 | -59.8856 |
| 770 | -21.1453 | 4 | -1.87151 | -5.48943 | -32.459 | 2.85944 | 12.1337 | 12.4641 | -53.5018 |
| 771 | -27.5442 | 6 | -3.85642 | -5.24373 | -32.2694 | 6.54288 | 12.5117 | 7.59308 | -50.9365 |
| 772 | -22.3562 | 6 | -1.81352 | -5.22028 | -34.7482 | 5.11995 | 13.1914 | 10.5821 | -58.7577 |
| 773 | -25.7262 | 4 | -2.12658 | -5.32575 | -36.1252 | 7.74176 | 12.8866 | 11.0342 | -53.6226 |
| 774 | -28.1113 | 5 | -2.9985 | -4.83578 | -36.2073 | 6.45458 | 12.4647 | 9.9602 | -50.9219 |
| 775 | -25.0518 | 4 | -3.45652 | -5.39808 | -34.1894 | 6.78673 | 15.0267 | 11.8626 | -44.5974 |
| 776 | -27.1837 | 5 | -6.7489 | -5.21882 | -34.3048 | 3.86242 | 18.1392 | 16.7999 | -82.2765 |
| 777 | -27.7304 | 6 | -6.32468 | -5.60081 | -32.4923 | 5.73604 | 15.3393 | 14.2597 | -69.2965 |
| 778 | -25.4614 | 4 | -1.99684 | -5.81422 | -34.7166 | 8.26277 | 13.062 | 9.10037 | -52.3318 |
| 779 | -26.3872 | 4 | -2.30435 | -5.53426 | -35.2406 | 6.19308 | 13.8298 | 8.51363 | -71.0233 |
| 780 | -29.481 | 4 | -5.77362 | -5.17099 | -34.9178 | 9.61738 | 17.0632 | 12.5929 | -53.6159 |
| 781 | -31.9142 | 5 | -5.96314 | -5.55509 | -35.0955 | 5.43715 | 15.1523 | 11.5541 | -72.4213 |
| 782 | -29.2815 | 5 | -4.73674 | -5.78695 | -34.5315 | 10.4053 | 14.9467 | 10.4563 | -70.9012 |
| 783 | -20.5422 | 5 | -1.6265 | -5.85366 | -30.9208 | 5.53309 | 11.7316 | 10.0256 | -27.9806 |
| 784 | -21.9396 | 4 | -7.14535 | -3.88771 | -18.7136 | 5.03232 | 13.1857 | 7.82904 | -51.8007 |
| 785 | -26.2482 | 4 | -3.15372 | -5.28654 | -33.3084 | 8.16103 | 13.0835 | 9.64908 | -78.4418 |
| 786 | -25.0524 | 5 | -4.31125 | -5.26507 | -31.5661 | 3.10913 | 14.977 | 10.1757 | -68.7304 |
| 787 | -18.4533 | 4 | -1.54352 | -5.46939 | -31.0136 | 3.94856 | 11.9472 | 13.287 | -61.2921 |
| 788 | -24.9181 | 5 | -2.25722 | -5.70261 | -35.1198 | 5.43862 | 13.094 | 10.4739 | -67.9227 |
| 789 | -20.5926 | 11 | -1.40372 | -7.44483 | -38.5514 | 13.7624 | 15.7653 | 12.2337 | -85.7321 |
| 790 | -26.5761 | 11 | -5.81301 | -7.08832 | -38.7489 | 22.6458 | 21.4303 | 13.57 | -79.3394 |
| 791 | -28.5759 | 11 | -1.85951 | -7.30964 | -44.8487 | 29.2239 | 16.9305 | 9.96499 | -95.8304 |
| 792 | -19.9657 | 11 | -2.5676 | -7.19408 | -40.4443 | 17.0141 | 20.5229 | 14.8522 | -92.8447 |
| 793 | -26.6616 | 11 | -3.35606 | -7.68214 | -45.4832 | 18.6865 | 21.3871 | 14.8775 | -103.936 |
| 794 | -30.7722 | 14 | -9.22321 | -8.39312 | -38.2952 | 21.5498 | 23.1987 | 16.1851 | -78.5134 |
| 795 | -21.1154 | 11 | -1.41006 | -8.11329 | -45.3103 | 18.5161 | 18.591 | 19.1741 | -97.6533 |
| 796 | -32.7195 | 10 | -4.49676 | -7.73873 | -47.0057 | 22.081 | 20.8937 | 13.9057 | -69.6262 |
| 797 | -33.5084 | 10 | -7.31003 | -6.90331 | -44.9986 | 19.2605 | 24.9032 | 15.0491 | -105.702 |
| 798 | -28.6551 | 14 | -7.06185 | -7.82471 | -44.0785 | 13.7325 | 24.0424 | 18.3342 | -91.7228 |
| 799 | -33.1876 | 14 | -8.84239 | -7.11694 | -42.5248 | 21.3816 | 24.8367 | 14.317 | -100.176 |
| 800 | -23.0083 | 12 | -1.70728 | -8.11159 | -44.1829 | 20.6646 | 18.2514 | 15.2125 | -113.747 |
| 801 | -18.9723 | 12 | -1.97484 | -8.43781 | -39.8372 | 17.5188 | 18.7497 | 15.5338 | -93.9332 |
| 802 | -34.7153 | 6 | -8.74758 | -6.01817 | -37.4857 | 15.0208 | 19.8031 | 15.8812 | -51.5776 |
| 803 | -20.1009 | 11 | -1.63081 | -7.63809 | -38.1249 | 19.0659 | 15.854 | 13.2985 | -56.6444 |
| 804 | -24.2662 | 10 | -1.08157 | -8.17664 | -41.9828 | 15.7293 | 15.1763 | 12.9532 | -93.9961 |
| 805 | -26.6193 | 10 | -6.1853 | -7.15032 | -37.7343 | 20.4619 | 20.1178 | 15.8579 | -31.2201 |
| 806 | -28.8778 | 21 | -4.61172 | -9.67106 | -62.3361 | 31.2655 | 28.3934 | 26.8341 | -102.702 |
| 807 | -28.967 | 14 | -4.10027 | -8.89318 | -42.2559 | 14.0136 | 14.7577 | 15.3829 | -106.793 |
| 808 | -37.4641 | 17 | -6.78186 | -9.84336 | -49.9478 | 28.7523 | 22.5481 | 13.8269 | -70.5345 |
| 809 | -18.8113 | 17 | -4.86873 | -9.33406 | -45.8584 | 23.6778 | 25.787 | 24.3613 | -68.4523 |
| 810 | -29.0301 | 17 | -6.4919 | -7.68887 | -42.9306 | 11 | 20.9048 | 14.5422 | -93.2264 |
| 811 | -26.4772 | 19 | -4.23553 | -7.51231 | -49.6322 | 16.8416 | 22.6934 | 15.8849 | -87.6139 |
| 812 | -23.4977 | 15 | -5.43228 | -8.60677 | -36.2285 | 20.3361 | 20.0625 | 12.4153 | -58.5135 |
| 813 | -17.9028 | 17 | -5.9856 | -7.63796 | -35.9452 | 14.1311 | 24.2076 | 15.0722 | -78.2184 |
| 814 | -21.5858 | 17 | -4.97281 | -9.6512 | -44.7117 | 26.1609 | 24.5794 | 20.5135 | -58.6793 |
| 815 | -24.8005 | 16 | -6.36287 | -8.47452 | -39.3209 | 21.359 | 21.9221 | 15.5374 | -27.313 |
| 816 | -28.042 | 19 | -10.0983 | -9.27661 | -35.9514 | 15.5484 | 24.5438 | 14.9479 | -93.6411 |
| 817 | -33.1574 | 19 | -5.23021 | -8.48621 | -49.0449 | 30.6975 | 18.3525 | 14.608 | -73.6776 |
| 818 | -28.1278 | 18 | -6.82556 | -9.74901 | -51.3305 | 24.3872 | 26.1538 | 25.133 | -106.808 |
| 819 | -47.5162 | 18 | -7.02239 | -10.16 | -59.088 | 31.2274 | 20.7456 | 14.8279 | -71.3342 |
| 820 | -32.0165 | 19 | -4.38901 | -9.39362 | -55.8675 | 32.0068 | 24.9302 | 16.8959 | -78.8358 |
| 821 | -38.3761 | 20 | -8.57315 | -7.05044 | -52.9001 | 19.8434 | 27.2898 | 12.8341 | -74.585 |
| 822 | -33.991 | 19 | -7.32551 | -8.67439 | -57.4834 | 23.2105 | 29.7651 | 21.3848 | -125.785 |
| 823 | -27.3771 | 17 | -5.00898 | -8.55798 | -49.1482 | 25.5177 | 25.6573 | 16.2648 | -91.3933 |
| 824 | -26.2715 | 19 | -5.44409 | -10.3278 | -46.3382 | 41.317 | 20.9656 | 20.6601 | -63.83 |
| 825 | -25.7576 | 21 | -6.04468 | -8.17837 | -53.0601 | 33.2891 | 31.2182 | 18.2749 | -118.073 |
| 826 | -14.0557 | 21 | -4.42987 | -9.89654 | -43.9648 | 23.8676 | 30.1362 | 19.0078 | -98.1143 |
| 827 | -37.894 | 20 | -7.31362 | -8.93502 | -56.4881 | 25.8867 | 25.9657 | 17.5906 | -115.201 |
| 828 | -34.1436 | 23 | -7.55958 | -9.39305 | -57.4916 | 32.4344 | 29.343 | 19.636 | -88.8157 |
| 829 | -30.912 | 23 | -3.96668 | -10.4895 | -53.4185 | 28.9592 | 22.0829 | 13.9514 | -97.2203 |
| 830 | -26.4905 | 23 | -6.59953 | -9.295 | -57.549 | 36.7149 | 32.9662 | 23.4217 | -69.272 |
| 831 | -33.2892 | 15 | -3.50675 | -8.72281 | -43.4906 | 26.814 | 13.3714 | 8.92313 | -84.9877 |
| 832 | -27.8145 | 16 | -6.4635 | -8.00336 | -43.2153 | 15.0008 | 22.5872 | 16.0232 | -110.421 |
| 833 | -25.4665 | 18 | -3.98967 | -9.1371 | -48.4018 | 18.0246 | 21.183 | 18.8846 | -105.285 |
| 834 | -28.2801 | 18 | -3.17037 | -8.26925 | -50.582 | 13.9425 | 19.8525 | 15.415 | -63.4471 |
| 835 | -33.8395 | 18 | -6.96938 | -7.58092 | -44.728 | 14.9812 | 23.4435 | 7.90545 | -88.4023 |
| 836 | -30.6787 | 18 | -4.82474 | -8.47311 | -52.391 | 16.2875 | 22.1046 | 18.5113 | -94.1733 |
| 837 | -31.777 | 17 | -6.33596 | -8.2688 | -40.9701 | 19.5611 | 17.3619 | 11.4269 | -73.0355 |
| 838 | -24.2874 | 18 | -5.17608 | -9.21632 | -41.2232 | 14.5536 | 20.9633 | 14.6723 | -110.59 |
| 839 | -26.9527 | 15 | -3.91922 | -8.43083 | -39.4376 | 21.062 | 15.5255 | 11.2198 | -48.3223 |
| 840 | -32.1705 | 15 | -4.27805 | -7.58193 | -42.2726 | 20.406 | 15.0634 | 8.64833 | -49.7591 |
| 841 | -22.8349 | 17 | -3.1836 | -8.27015 | -42.7652 | 15.6279 | 19.9452 | 12.6771 | -90.0505 |
| 842 | -24.3541 | 19 | -4.89456 | -7.63217 | -46.145 | 19.3213 | 24.0109 | 14.9689 | -91.5392 |
| 843 | -22.1032 | 15 | -3.85236 | -8.74741 | -35.1674 | 11.9809 | 16.8764 | 10.6276 | -57.6709 |
| 844 | -24.8705 | 17 | -2.59861 | -9.44076 | -43.8147 | 25.7443 | 16.9208 | 13.5658 | -67.2479 |
| 845 | -31.5938 | 15 | -5.14233 | -7.39174 | -46.8538 | 16.0379 | 19.3637 | 14.6966 | -83.9579 |
| 846 | -30.507 | 20 | -5.41818 | -8.89228 | -49.1328 | 15.3219 | 22.0161 | 14.8175 | -111.161 |
| 847 | -39.885 | 21 | -10.2655 | -8.83239 | -45.9384 | 25.3816 | 23.5209 | 11.6698 | -83.1619 |
| 848 | -24.095 | 21 | -5.98292 | -8.15034 | -36.9594 | 22.5876 | 21.0387 | 7.70132 | -89.4335 |
| 849 | -31.5429 | 6 | -4.85759 | -7.01704 | -39.8655 | 19.2539 | 15.696 | 15.0632 | -36.6361 |
| 850 | -32.3573 | 6 | -4.843 | -7.11204 | -40.4965 | 19.1149 | 16.4436 | 13.9764 | -38.6191 |
| 851 | -36.3982 | 7 | -8.03073 | -5.95254 | -42.6758 | 22.5292 | 21.7942 | 15.2089 | -67.4348 |
| 852 | -30.2986 | 6 | -6.43809 | -5.11897 | -35.271 | 14.124 | 19.0014 | 10.3628 | -79.2178 |
| 853 | -27.901 | 6 | -4.80417 | -5.73945 | -35.1752 | 11.796 | 18.4543 | 8.8386 | -44.2882 |
| 854 | -24.3073 | 6 | -2.87917 | -6.58657 | -38.3521 | 15.9551 | 15.8019 | 15.5919 | -66.7044 |
| 855 | -31.9016 | 6 | -5.72445 | -6.84553 | -37.204 | 17.9676 | 17.1359 | 11.9997 | -55.3311 |
| 856 | -30.125 | 6 | -5.23125 | -6.91566 | -36.6278 | 21.0378 | 16.3235 | 12.9223 | -36.1701 |
| 857 | -36.8699 | 7 | -6.33006 | -6.38271 | -44.644 | 24.8175 | 19.6063 | 13.88 | -36.6331 |
| 858 | -29.2144 | 6 | -8.72743 | -6.57942 | -32.1324 | 15.5669 | 21.4234 | 14.7561 | -62.885 |
| 859 | -23.8579 | 9 | -2.03495 | -8.26117 | -33.982 | 25.6367 | 14.6718 | 6.67207 | -53.7545 |
| 860 | -37.0053 | 6 | -6.24691 | -6.59327 | -41.561 | 21.723 | 17.3111 | 12.4161 | -44.6814 |
| 861 | -31.8329 | 6 | -5.24487 | -6.58631 | -41.1739 | 19.5499 | 18.4995 | 14.4678 | -49.7949 |
| 862 | -16.9741 | 6 | -1.77116 | -6.20998 | -33.4858 | 11.2403 | 18.3126 | 11.8468 | -96.7937 |
| 863 | -18.0462 | 6 | -3.24047 | -6.74848 | -30.0734 | 13.7358 | 16.5199 | 13.2683 | -86.6633 |
| 864 | -30.4618 | 7 | -5.37369 | -6.98567 | -39.4363 | 19.6436 | 17.0431 | 15.5576 | -37.9364 |
| 865 | -25.5689 | 6 | -2.58237 | -6.74907 | -40.1822 | 19.4908 | 15.6 | 15.7099 | -73.721 |
| 866 | -38.2854 | 6 | -8.28662 | -6.17096 | -37.9556 | 17.7771 | 18.7353 | 10.7854 | -53.0416 |
| 867 | -32.251 | 7 | -6.30101 | -6.63239 | -37.0433 | 19.2471 | 18.3188 | 10.9674 | -48.4054 |
| 868 | -38.5197 | 8 | -5.6605 | -7.1056 | -44.4287 | 25.0323 | 17.8494 | 10.3087 | -39.8954 |
| 869 | -25.2969 | 6 | -3.17078 | -6.01659 | -35.6764 | 26.0699 | 18.124 | 7.93897 | -35.2593 |
| 870 | -21.2097 | 7 | -3.03847 | -7.03495 | -30.6815 | 16.4832 | 15.3974 | 9.27952 | -53.8437 |
| 871 | -21.9553 | 10 | -1.38696 | -7.60477 | -40.7634 | 10.6028 | 15.7065 | 14.5946 | -104.181 |
| 872 | -22.16 | 10 | -2.36621 | -7.67953 | -39.114 | 20.5649 | 17.2234 | 13.8913 | -96.0736 |
| 873 | -24.4664 | 10 | -3.26086 | -8.5525 | -40.3541 | 13.8751 | 16.59 | 17.311 | -110.108 |
| 874 | -23.6638 | 10 | -2.87685 | -8.0992 | -38.2593 | 13.1786 | 15.3579 | 14.8212 | -110.846 |
| 875 | -20.273 | 10 | -2.44282 | -8.05898 | -38.951 | 20.3288 | 16.1948 | 18.3511 | -109.421 |
| 876 | -21.9978 | 11 | -4.92241 | -7.29413 | -39.3278 | 21.8499 | 21.9185 | 17.6241 | -105.178 |
| 877 | -17.314 | 6 | -3.02555 | -5.77039 | -25.7098 | 10.3462 | 14.6409 | 8.08476 | -41.003 |
| 878 | -26.4151 | 12 | -2.83573 | -6.95155 | -42.8461 | 14.6962 | 18.0622 | 11.3884 | -84.0246 |
| 879 | -23.4509 | 12 | -2.47224 | -6.64326 | -44.2339 | 16.8756 | 20.0237 | 14.0469 | -97.38 |
| 880 | -24.5856 | 11 | -2.45667 | -7.82324 | -41.7697 | 15.1193 | 18.547 | 12.3193 | -94.8153 |
| 881 | -22.0034 | 10 | -5.72065 | -7.27866 | -37.7233 | 12.1512 | 22.8865 | 18.0378 | -107.745 |
| 882 | -30.3032 | 10 | -4.98323 | -7.29666 | -41.5371 | 16.4243 | 20.083 | 11.6182 | -102.167 |
| 883 | -27.6244 | 14 | -7.48691 | -8.02901 | -37.6943 | 21.4468 | 21.5322 | 14.9124 | -86.9664 |
| 884 | -18.3921 | 4 | -1.01999 | -5.85794 | -28.9634 | 4.80601 | 10.7633 | 10.0064 | -58.0893 |
| 885 | -22.6663 | 5 | -1.5836 | -6.10662 | -30.4085 | 8.03342 | 10.2075 | 7.85527 | -49.57 |
| 886 | -21.739 | 6 | -2.43504 | -6.877 | -29.6642 | 8.00966 | 11.8598 | 9.39184 | -39.377 |
| 887 | -22.1219 | 7 | -1.62025 | -5.92802 | -32.2358 | 8.51294 | 10.4163 | 9.3525 | -45.9847 |
| 888 | -21.4025 | 7 | -2.44297 | -7.06981 | -30.0932 | 8.51708 | 11.4957 | 10.2824 | -45.6165 |
| 889 | -21.9209 | 8 | -1.69372 | -6.32052 | -32.9723 | 8.94707 | 10.8068 | 10.1002 | -45.8421 |
| 890 | -21.5215 | 8 | -1.25546 | -6.7969 | -32.2581 | 7.83413 | 11.0398 | 8.16214 | -36.2169 |
| 891 | -20.6239 | 9 | -2.75668 | -6.75114 | -30.0223 | 14.748 | 13.0793 | 8.61614 | -50.3999 |
| 892 | -19.6831 | 9 | -2.14803 | -7.54769 | -31.4767 | 11.1255 | 13.2034 | 10.5796 | -42.7162 |
| 893 | -22.0274 | 10 | -1.70991 | -6.88003 | -35.6408 | 9.72742 | 12.3591 | 11.0298 | -47.1327 |
| 894 | -21.326 | 6 | -2.46336 | -6.75773 | -29.666 | 10.7238 | 11.7344 | 10.1503 | -42.8296 |
| 895 | -22.9856 | 7 | -1.58911 | -6.10162 | -33.3449 | 8.2435 | 10.404 | 9.79517 | -44.68 |
| 896 | -15.1222 | 6 | -0.94139 | -6.03516 | -29.3664 | 8.7962 | 13.7456 | 10.2428 | -62.5682 |
| 897 | -22.7387 | 7 | -1.48803 | -6.79912 | -32.0681 | 6.31384 | 10.6755 | 8.24686 | -43.8334 |
| 898 | -22.4613 | 8 | -1.46094 | -6.5359 | -34.1606 | 8.12285 | 11.3304 | 9.81317 | -45.5221 |
| 899 | -21.4455 | 8 | -1.29531 | -7.53983 | -33.0231 | 12.3849 | 11.8636 | 9.40711 | -51.2247 |
| 900 | -16.9099 | 9 | -2.43317 | -7.37791 | -28.2755 | 15.2073 | 13.5084 | 10.5052 | -60.3758 |
| 901 | -23.702 | 6 | -1.71541 | -6.67393 | -32.782 | 6.97204 | 10.7636 | 9.42252 | -45.5852 |
| 902 | -24.2648 | 7 | -1.6681 | -6.47488 | -34.6852 | 8.59825 | 11.0665 | 9.81661 | -47.1159 |
| 903 | -24.7211 | 6 | -1.68899 | -6.93758 | -33.9924 | 6.65035 | 11.4672 | 9.07919 | -48.121 |
| 904 | -20.1942 | 4 | -0.98702 | -5.75261 | -32.202 | 9.30694 | 12.9418 | 9.47406 | -63.9104 |
| 905 | -25.5997 | 6 | -1.51791 | -6.2413 | -34.1617 | 9.57913 | 9.80253 | 8.53334 | -44.5341 |
| 906 | -19.676 | 6 | -1.36037 | -6.44953 | -33.171 | 8.3894 | 13.9031 | 10.9553 | -86.0924 |
| 907 | -20.0027 | 7 | -1.46966 | -6.59451 | -31.759 | 10.229 | 11.4508 | 10.7649 | -55.6565 |
| 908 | -19.427 | 8 | -2.15525 | -6.49681 | -36.7399 | 7.93572 | 13.5968 | 18.365 | -54.7097 |
| 909 | -25.3004 | 10 | -3.97616 | -5.54064 | -32.8889 | 11.8294 | 14.4285 | 6.87738 | -51.3525 |
| 910 | -23.8152 | 11 | -3.56008 | -6.41803 | -32.716 | 9.5294 | 14.3747 | 7.31354 | -60.8462 |
| 911 | -20.0622 | 9 | -5.00603 | -6.10965 | -28.1009 | 15.2404 | 16.704 | 10.4083 | -60.9538 |
| 912 | -28.9177 | 10 | -4.04948 | -6.40844 | -35.0053 | 11.1328 | 12.823 | 7.58963 | -59.8667 |
| 913 | -22.1024 | 6 | -3.76777 | -5.70069 | -29.0894 | 9.79316 | 13.9653 | 9.49231 | -34.486 |
| 914 | -19.1773 | 8 | -1.65416 | -6.81249 | -34.8905 | 10.258 | 12.9567 | 15.0682 | -71.8688 |
| 915 | -26.6784 | 7 | -1.82438 | -7.91403 | -37.5479 | 14.611 | 10.9161 | 12.7281 | -94.6937 |
| 916 | -19.2268 | 3 | -0.94546 | -5.85602 | -31.7645 | 6.94137 | 13.2297 | 10.8044 | -55.2457 |
| 917 | -17.3665 | 5 | -0.96039 | -6.53367 | -31.9071 | 6.55112 | 14.4136 | 11.4209 | -76.7956 |
| 918 | -16.0317 | 4 | -0.94889 | -6.19997 | -27.4652 | 8.76797 | 12.5904 | 9.31115 | -18.467 |
| 919 | -21.7508 | 6 | -1.49313 | -6.99878 | -35.0739 | 9.86245 | 12.6681 | 13.165 | -31.3457 |
| 920 | -15.5863 | 5 | -2.50274 | -7.02161 | -29.2886 | 18.6286 | 15.3748 | 15.466 | -31.0472 |
| 921 | -17.1545 | 7 | -0.85791 | -7.25182 | -35.8692 | 11.9067 | 15.4466 | 15.0893 | -97.9495 |
| 922 | -19.2676 | 4 | -0.83792 | -6.7615 | -31.1257 | 7.99971 | 11.5469 | 11.285 | -42.2561 |
| 923 | -16.9283 | 4 | -0.85508 | -6.61503 | -28.3233 | 10.3757 | 12.1407 | 9.82567 | -33.6867 |
| 924 | -18.2351 | 6 | -1.22861 | -7.2194 | -35.9476 | 9.79394 | 16.7431 | 14.3949 | -112.713 |
| 925 | -17.3134 | 4 | 0 | -7.12202 | -34.376 | 7.43881 | 13.5396 | 14.06 | -80.8257 |
| 926 | -17.4952 | 6 | 0 | -7.52062 | -37.4373 | 10.2649 | 14.6408 | 15.758 | -81.0633 |
| 927 | -21.1105 | 4 | -2.54765 | -6.7278 | -32.4049 | 12.747 | 14.8861 | 13.1561 | -49.8122 |
| 928 | -20.702 | 6 | -1.42273 | -7.1828 | -34.9515 | 10.905 | 15.8138 | 10.9107 | -84.6502 |
| 929 | -23.6372 | 5 | -1.72927 | -8.03348 | -33.2253 | 16.2878 | 11.5472 | 11.6442 | -14.0969 |
| 930 | -20.3247 | 7 | -1.41522 | -7.64511 | -35.9695 | 11.9728 | 17.2612 | 10.9116 | -86.1897 |
| 931 | -14.7694 | 4 | -1.30969 | -7.4936 | -25.0234 | 47.1777 | 13.5481 | 9.13204 | 32.2226 |
| 932 | -23.4074 | 6 | -2.65322 | -7.55778 | -37.1114 | 19.8436 | 16.2845 | 14.6469 | -79.119 |
| 933 | -24.4061 | 7 | -3.11551 | -8.57202 | -35.7463 | 11.6386 | 14.9574 | 14.4661 | -72.6869 |
| 934 | -27.8969 | 7 | -4.83476 | -7.31643 | -41.9422 | 17.3201 | 22.6379 | 15.1529 | -94.617 |
| 935 | -22.6319 | 8 | -3.22498 | -6.99101 | -39.1012 | 11.3745 | 19.1385 | 15.3918 | -66.3999 |
| 936 | -23.5686 | 9 | -3.99811 | -7.53947 | -39.7403 | 8.54537 | 20.8187 | 15.643 | -69.189 |
| 937 | -27.3305 | 6 | -2.8233 | -7.42446 | -43.0533 | 16.1859 | 17.4317 | 16.9187 | -37.0682 |
| 938 | -21.3207 | 3 | -1.30475 | -5.70521 | -31.567 | 6.5355 | 12.5474 | 9.33225 | -44.2973 |
| 939 | -22.9496 | 8 | -2.89738 | -6.67724 | -41.1752 | 11.4393 | 21.896 | 13.3449 | -89.8211 |
| 940 | -16.6434 | 3 | -2.52104 | -5.90728 | -26.9056 | 4.99419 | 12.5718 | 14.1961 | -84.2017 |
| 941 | -22.2003 | 3 | -1.34717 | -5.76506 | -31.6743 | 5.38163 | 12.3437 | 8.61695 | -47.3851 |
| 942 | -33.6348 | 5 | -5.49037 | -6.47948 | -40.5944 | 12.9514 | 16.695 | 14.6519 | -78.7392 |
| 943 | -25.7865 | 6 | -4.44508 | -7.09403 | -37.5998 | 12.3685 | 20.7152 | 13.1453 | -107.682 |
| 944 | -21.4254 | 7 | -2.22377 | -6.63782 | -37.7773 | 10.4884 | 18.5286 | 12.6196 | -61.7178 |
| 945 | -23.2864 | 8 | -2.44417 | -7.31581 | -38.59 | 16.2815 | 16.4325 | 14.0372 | -48.8298 |
| 946 | -20.8621 | 8 | -3.27502 | -7.2792 | -39.9 | 14.1666 | 23.5696 | 14.7328 | -52.7338 |
| 947 | -18.0841 | 6 | -1.79139 | -7.21874 | -37.1223 | 22.248 | 20.5621 | 14.2203 | -75.9515 |
| 948 | -32.5207 | 8 | -7.01144 | -7.37875 | -38.9386 | 11.3629 | 19.2432 | 14.952 | -77.7778 |
| 949 | -29.6037 | 8 | -7.93622 | -7.16188 | -32.4555 | 14.1431 | 18.7754 | 13.4051 | -36.8366 |
| 950 | -21.0201 | 6 | -3.35691 | -7.96694 | -35.1836 | 6.81256 | 18.0952 | 16.3984 | -60.495 |
| 951 | -25.5378 | 9 | -4.15254 | -7.36046 | -38.5503 | 17.157 | 20.4769 | 11.655 | -92.2568 |
| 952 | -25.0983 | 4 | -3.81379 | -5.33939 | -29.6908 | 2.53823 | 13.0771 | 8.50138 | -78.4265 |
| 953 | -22.4553 | 6 | -2.65341 | -7.51902 | -36.7798 | 19.033 | 16.6963 | 15.0806 | -78.9864 |
| 954 | -23.9405 | 6 | -2.39032 | -7.02094 | -38.4411 | 16.06 | 18.9514 | 11.2706 | -99.0338 |
| 955 | -25.6365 | 9 | -3.71633 | -8.50073 | -41.0488 | 19.8216 | 18.5219 | 16.998 | -68.2763 |
| 956 | -21.5378 | 8 | -2.8297 | -6.8142 | -37.5543 | 10.0238 | 15.4474 | 17.2056 | -79.3442 |
| 957 | -23.4437 | 6 | -2.82577 | -8.52406 | -38.8932 | 18.5901 | 18.3377 | 16.6008 | -74.605 |
| 958 | -21.3512 | 6 | -2.58928 | -7.15078 | -38.8749 | 21.7923 | 19.3416 | 16.3022 | -74.9595 |
| 959 | -15.9054 | 6 | -2.06814 | -6.74753 | -32.2235 | 20.7961 | 18.1343 | 13.4404 | -76.507 |
| 960 | -29.6317 | 9 | -5.19875 | -7.04715 | -44.8266 | 15.0808 | 22.0697 | 16.844 | -74.2783 |
| 961 | -23.1261 | 8 | -3.67212 | -7.67028 | -38.5837 | 18.9737 | 20.6364 | 14.5604 | -70.2681 |
| 962 | -28.4331 | 11 | -5.26055 | -7.10836 | -45.4079 | 17.0715 | 22.6181 | 17.3986 | -77.5074 |
| 963 | -26.4096 | 12 | -6.45725 | -7.40093 | -39.5907 | 20.1652 | 25.9299 | 11.8623 | -76.281 |
| 964 | -21.1295 | 9 | -4.667 | -7.1571 | -42.1502 | 44.8996 | 24.261 | 21.2541 | -91.3999 |
| 965 | -23.8292 | 11 | -5.57487 | -8.12875 | -41.4307 | 42.3147 | 23.9042 | 19.1273 | -82.7514 |
| 966 | -33.7516 | 8 | -10.1934 | -6.7626 | -35.2522 | 14.3424 | 22.6194 | 15.2369 | -37.6422 |
| 967 | -27.5126 | 8 | -7.1678 | -7.28533 | -38.4462 | 18.32 | 26.117 | 14.4872 | -97.8594 |
| 968 | -22.8148 | 8 | -3.34494 | -7.74312 | -40.027 | 19.2755 | 24.5013 | 11.6467 | -88.3412 |
| 969 | -25.1268 | 8 | -4.20451 | -7.03578 | -36.646 | 27.3521 | 17.2704 | 13.8148 | -84.4035 |
| 970 | -22.9034 | 7 | -4.30565 | -6.59035 | -35.6605 | 11.0283 | 21.1474 | 12.1293 | -83.6918 |
| 971 | -18.814 | 8 | -4.03188 | -6.55008 | -40.2048 | 21.3762 | 22.617 | 21.5414 | -50.6402 |
| 972 | -19.8138 | 9 | -2.72751 | -6.68793 | -35.7733 | 22.0749 | 19.6337 | 10.9331 | -43.6754 |
| 973 | -18.5123 | 7 | -1.95214 | -7.46775 | -39.4967 | 31.2831 | 20.2161 | 17.522 | -47.4845 |
| 974 | -16.6998 | 7 | -1.08347 | -6.72041 | -37.69 | 14.8636 | 19.3415 | 14.4364 | -64.7477 |
| 975 | -25.5811 | 8 | -4.68142 | -7.75519 | -40.7407 | 15.9487 | 20.6682 | 18.028 | -50.2448 |
| 976 | -23.7368 | 9 | -1.98607 | -6.8423 | -39.0261 | 14.7486 | 15.818 | 11.5681 | -40.9851 |
| 977 | -23.2566 | 10 | -4.10507 | -8.13345 | -40.295 | 13.0952 | 21.3632 | 16.4412 | -77.3119 |
| 978 | -14.6151 | 8 | -3.59157 | -8.25196 | -34.3055 | 21.9263 | 22.0176 | 19.6859 | -49.5558 |
| 979 | -27.0621 | 8 | -2.64939 | -7.26153 | -39.0072 | 19.2788 | 15.9099 | 10.2739 | -49.727 |
| 980 | -32.0501 | 8 | -3.77215 | -7.4676 | -40.2493 | 26.9292 | 14.4464 | 10.7746 | -25.7292 |
| 981 | -13.7503 | 9 | -1.45383 | -8.37012 | -35.952 | 10.4685 | 20.7439 | 15.9544 | -56.8867 |
| 982 | -18.8173 | 8 | -1.7234 | -7.22304 | -39.6163 | 17.759 | 22.3525 | 12.7792 | -88.5632 |
| 983 | -23.1592 | 8 | -4.29997 | -9.40563 | -37.6709 | 23.1568 | 20.996 | 16.8869 | -85.4381 |
| 984 | -16.7271 | 9 | -2.78978 | -7.71061 | -34.8652 | 88.258 | 21.7184 | 13.1417 | -11.4983 |
| 985 | -25.1464 | 9 | -4.16229 | -7.74841 | -42.5119 | 21.5728 | 23.0335 | 15.7765 | -72.9461 |
| 986 | -22.4821 | 8 | -3.67488 | -8.47157 | -40.5055 | 19.5448 | 22.6465 | 16.9877 | -81.526 |
| 987 | -22.9324 | 8 | -3.64581 | -8.12468 | -38.7618 | 38.0583 | 19.1056 | 17.2236 | -70.3944 |
| 988 | -14.1891 | 9 | -2.18986 | -8.72803 | -30.9796 | 77.6539 | 16.8102 | 15.4105 | 3.98305 |
| 989 | -27.7149 | 8 | -2.07549 | -8.72782 | -38.3831 | 21.4257 | 14.9237 | 8.74845 | -64.2386 |
| 990 | -25.0144 | 5 | -2.26364 | -5.35122 | -34.3355 | 5.6955 | 13.1031 | 8.79707 | -70.9439 |
| 991 | -24.1625 | 2 | -2.74564 | -4.89206 | -27.8393 | 2.81345 | 9.47718 | 8.47438 | -72.3558 |
| 992 | -25.5686 | 2 | -1.19187 | -5.49309 | -33.1951 | 6.07997 | 10.8006 | 7.60869 | -38.3827 |
| 993 | -25.24 | 3 | -2.32982 | -5.60728 | -34.3243 | 12.3148 | 12.9089 | 10.9907 | -57.729 |
| 994 | -23.9519 | 4 | -1.38005 | -5.11235 | -33.4785 | 7.99225 | 12.4482 | 7.13234 | -43.426 |
| 995 | -28.0287 | 3 | -4.66652 | -4.51743 | -33.7192 | 9.24639 | 16.1788 | 9.98384 | -62.5937 |
| 996 | -26.903 | 2 | -4.29055 | -6.07054 | -29.2048 | 8.08562 | 13.0391 | 9.46184 | -82.1634 |
| 997 | -24.7295 | 1 | -1.4547 | -5.66386 | -31.3883 | 4.71503 | 11.3254 | 7.63636 | -64.2414 |
| 998 | -26.9449 | 2 | -4.30172 | -6.07307 | -29.1661 | 8.04525 | 13.1052 | 9.31006 | -82.1545 |
| 999 | -22.7429 | 2 | -1.37457 | -6.02643 | -30.6643 | 14.3003 | 12.7871 | 7.04015 | -61.6788 |
| 1000 | -21.3056 | 1 | -2.31492 | -6.47846 | -30.3931 | 5.01171 | 14.0512 | 12.3431 | -68.2405 |

**Nflex:-** Number of rotatable torsions

**Hbond:-** hydrogen bond energy

**Hphob:-** hydrophobic energy in exposing a surface to water

**Vwint:-** The van der Waals interaction energy (sum of gc and gh van der Waals)

**Eintl:-** Internal conformational energy of the ligand

**Dsolv:-** The desolvation of exposed H-bond donors and acceptors

**SolEl:-** The solvation electrostatics energy change upon binding

**mfScore:**- mean force score

**REF-IN:-** Reference Inhibitor

Supplementary table 3: Structure and IUPAC Name of Designed Novel Inhibitors

| **Compound Index No.** | **Structure** | **IUPAC Name** |
| --- | --- | --- |
| 46 | 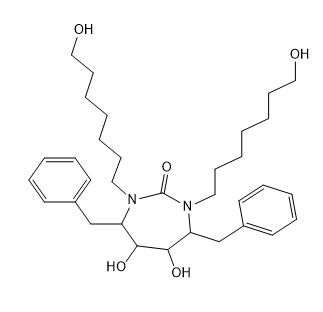 | 4,7-dibenzyl-5,6-dihydroxy-1,3-bis(7-hydroxyheptyl)-1,3-diazepan-2-one |
| 46a | 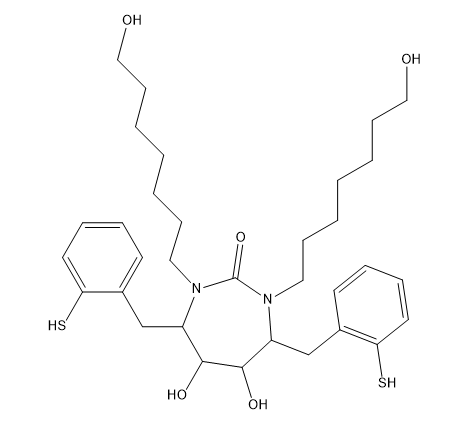 | 5,6-dihydroxy-1,3-bis(7-hydroxyheptyl)-4,7-bis(2-mercaptobenzyl)-1,3-diazepan-2-one |
| 46b | 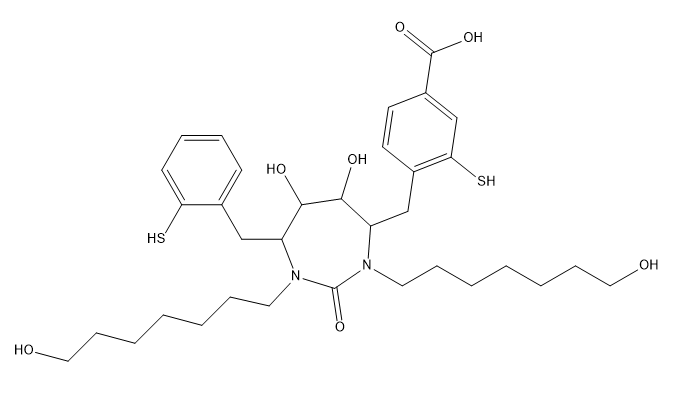 | 4-((5,6-dihydroxy-1,3-bis(7-hydroxyheptyl)-7-(2-mercaptobenzyl)-2-oxo-1,3-diazepan-4-yl)methyl)-3-mercaptobenzoic acid |
| 46c | 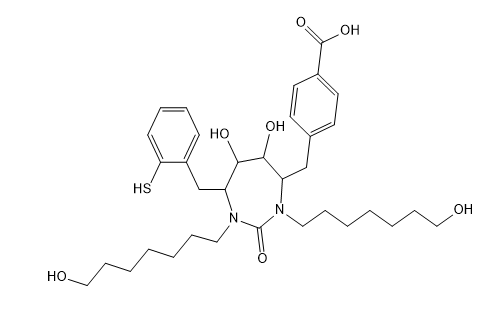 | 4-((5,6-dihydroxy-1,3-bis(7-hydroxyheptyl)-7-(2-mercaptobenzyl)-2-oxo-1,3-diazepan-4-yl)methyl)benzoic acid |
| 46d | 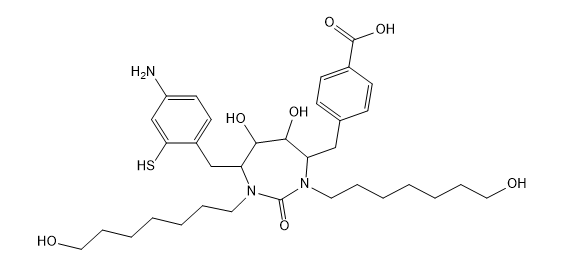 | 4-((7-(4-amino-2-mercaptobenzyl)-5,6-dihydroxy-1,3-bis(7-hydroxyheptyl)-2-oxo-1,3-diazepan-4-yl)methyl)benzoic acid |
| 46e | 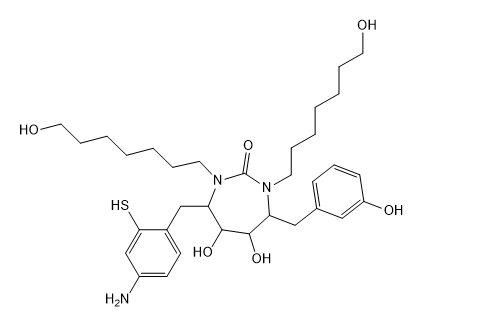 | 4-(4-amino-2-mercaptobenzyl)-5,6-dihydroxy-7-(3-hydroxybenzyl)-1,3-bis(7-hydroxyheptyl)-1,3-diazepan-2-one |
| 331 | 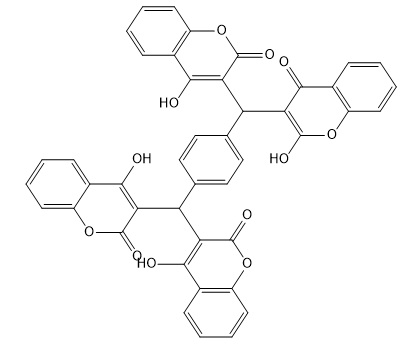 | 3,3'-((4-((4-hydroxy-2-oxo-2H-chromen-3-yl)(2-hydroxy-4-oxo-4H-chromen-3-yl)methyl)phenyl)methylene)bis(4-hydroxy-2H-chromen-2-one) |
| 331a | 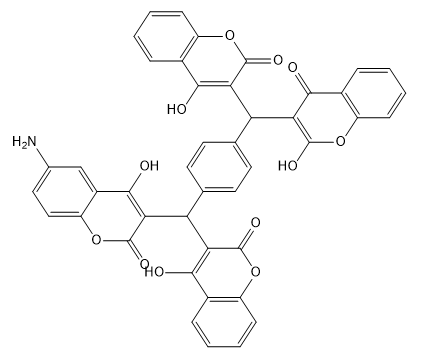 | 6-amino-4-hydroxy-3-((4-hydroxy-2-oxo-2H-chromen-3-yl)(4-((4-hydroxy-2-oxo-2H-chromen-3-yl)(2-hydroxy-4-oxo-4H-chromen-3-yl)methyl)phenyl)methyl)-2H-chromen-2-one |
| 331b | 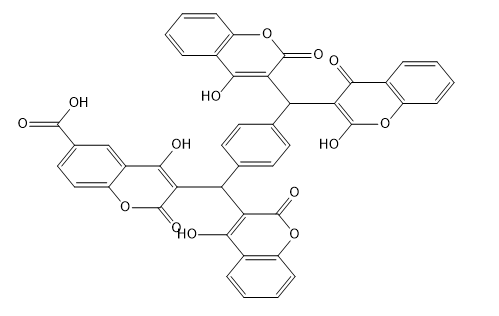 | 4-hydroxy-3-((4-hydroxy-2-oxo-2H-chromen-3-yl)(4-((4-hydroxy-2-oxo-2H-chromen-3-yl)(2-hydroxy-4-oxo-4H-chromen-3-yl)methyl)phenyl)methyl)-2-oxo-2H-chromene-6-carboxylic acid |
| 331c | 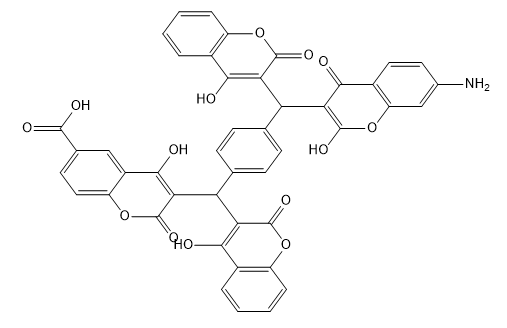 | 3-((4-((7-amino-2-hydroxy-4-oxo-4H-chromen-3-yl)(4-hydroxy-2-oxo-2H-chromen-3-yl)methyl)phenyl)(4-hydroxy-2-oxo-2H-chromen-3-yl)methyl)-4-hydroxy-2-oxo-2H-chromene-6-carboxylic acid |
| 331d | 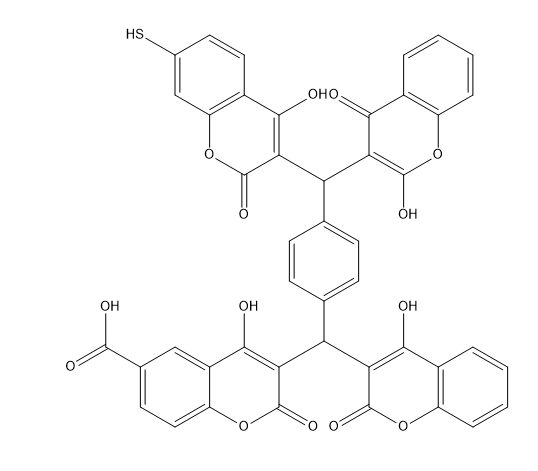 | 4-hydroxy-3-((4-hydroxy-2-oxo-2H-chromen-3-yl)(4-((2-hydroxy-4-oxo-4H-chromen-3-yl)(4-hydroxy-7-mercapto-2-oxo-2H-chromen-3-yl)methyl)phenyl)methyl)-2-oxo-2H-chromene-6-carboxylic acid |
| 331e | 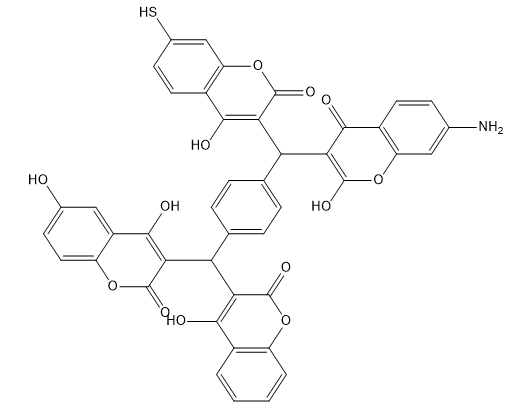 | 3-((7-amino-2-hydroxy-4-oxo-4H-chromen-3-yl)(4-((4,6-dihydroxy-2-oxo-2H-chromen-3-yl)(4-hydroxy-2-oxo-2H-chromen-3-yl)methyl)phenyl)methyl)-4-hydroxy-7-mercapto-2H-chromen-2-one |
| 441 | 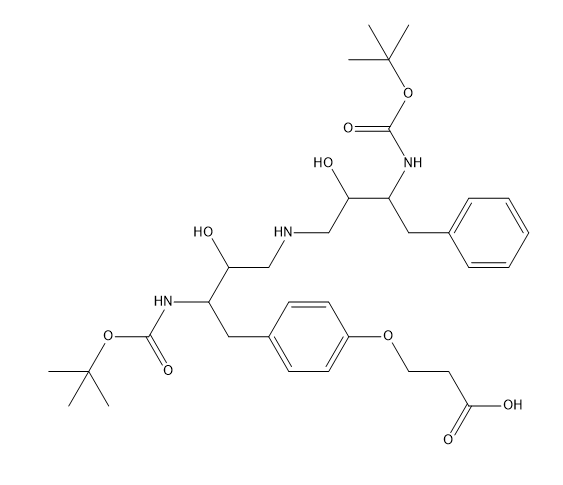 | 3-(4-(2-((tert-butoxycarbonyl)amino)-4-((3-((tert-butoxycarbonyl)amino)-2-hydroxy-4-phenylbutyl)amino)-3-hydroxybutyl)phenoxy)propanoic acid |
| 441a | 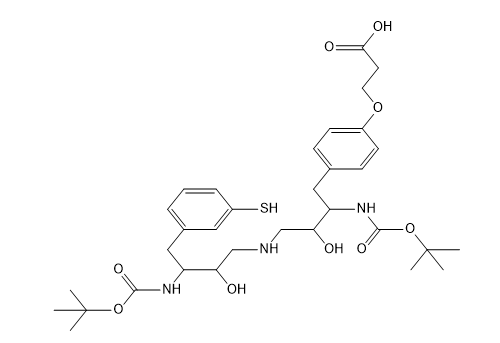 | 3-(4-(2-((tert-butoxycarbonyl)amino)-4-((3-((tert-butoxycarbonyl)amino)-2-hydroxy-4-(3-mercaptophenyl)butyl)amino)-3-hydroxybutyl)phenoxy)propanoic acid |
| 441b | 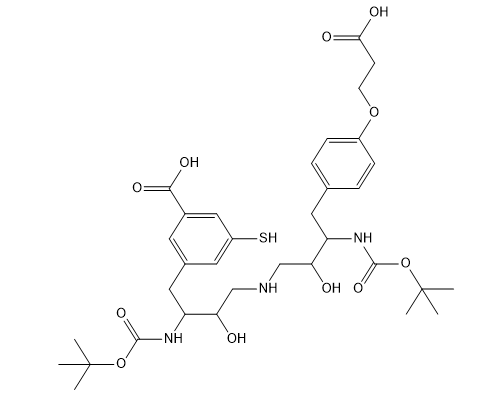 | 3-(2-((tert-butoxycarbonyl)amino)-4-((3-((tert-butoxycarbonyl)amino)-4-(4-(2-carboxyethoxy)phenyl)-2-hydroxybutyl)amino)-3-hydroxybutyl)-5-mercaptobenzoic acid |
| 441c | 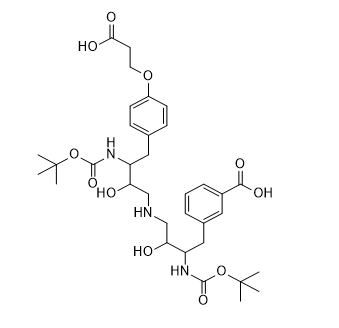 | 3-(2-((tert-butoxycarbonyl)amino)-4-((3-((tert-butoxycarbonyl)amino)-4-(4-(2-carboxyethoxy)phenyl)-2-hydroxybutyl)amino)-3-hydroxybutyl)benzoic acid |
| 441d | 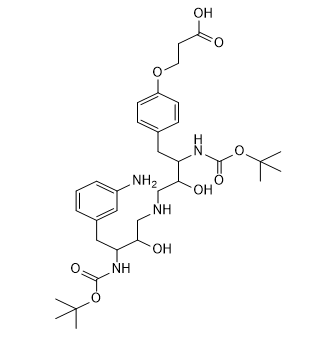 | 3-(4-(4-((4-(3-aminophenyl)-3-((tert-butoxycarbonyl)amino)-2-hydroxybutyl)amino)-2-((tert-butoxycarbonyl)amino)-3-hydroxybutyl)phenoxy)propanoic acid |
| 441e | 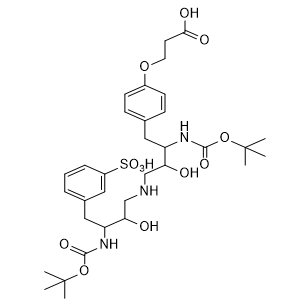 | 3-(4-(2-((tert-butoxycarbonyl)amino)-4-((3-((tert-butoxycarbonyl)amino)-2-hydroxy-4-(3-sulfophenyl)butyl)amino)-3-hydroxybutyl)phenoxy)propanoic acid |
| 741 | 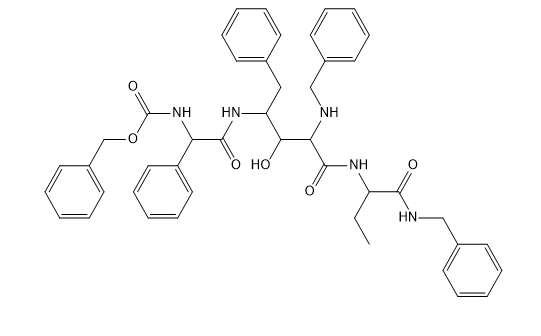 | benzyl (2-((4-(benzylamino)-5-((1-(benzylamino)-1-oxobutan-2-yl)amino)-3-hydroxy-5-oxo-1-phenylpentan-2-yl)amino)-2-oxo-1-phenylethyl) carbamate |
| 741a | 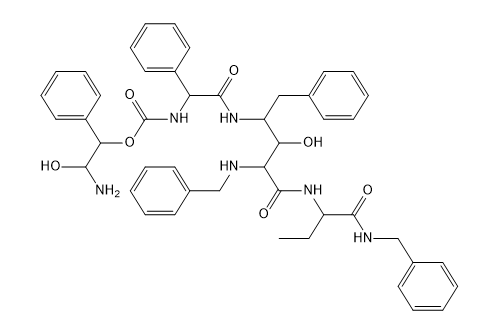 | 2-amino-2-hydroxy-1-phenylethyl (2-((4-(benzylamino)-5-((1-(benzylamino)-1-oxobutan-2-yl)amino)-3-hydroxy-5-oxo-1-phenylpentan-2-yl)amino)-2-oxo-1-phenylethyl) carbamate |
| 741b | 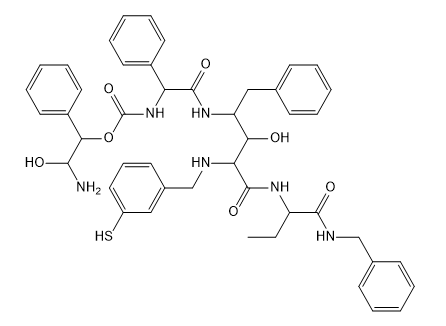 | 2-amino-2-hydroxy-1-phenylethyl (2-((5-((1-(benzylamino)-1-oxobutan-2-yl)amino)-3-hydroxy-4-((3-mercaptobenzyl)amino)-5-oxo-1-phenylpentan-2-yl)amino)-2-oxo-1-phenylethyl)carbamate |
| 741c | 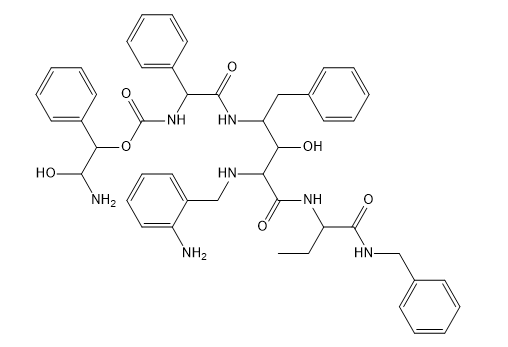 | 2-amino-2-hydroxy-1-phenylethyl (2-((4-((2-aminobenzyl)amino)-5-((1-(benzylamino)-1-oxobutan-2-yl)amino)-3-hydroxy-5-oxo-1-phenylpentan-2-yl)amino)-2-oxo-1-phenylethyl)carbamate |
| 741d | 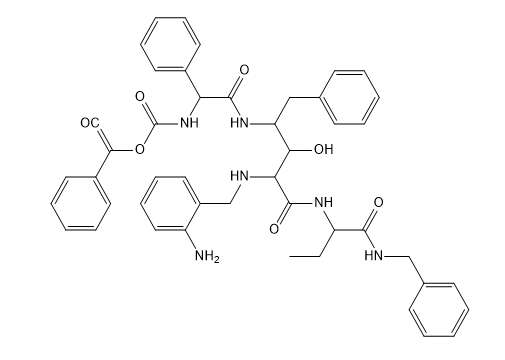 | 2-oxo-1-phenylvinyl (2-((4-((2-aminobenzyl)amino)-5-((1-(benzylamino)-1-oxobutan-2-yl)amino)-3-hydroxy-5-oxo-1-phenylpentan-2-yl)amino)-2-oxo-1-phenylethyl)carbamate |
| 741e | 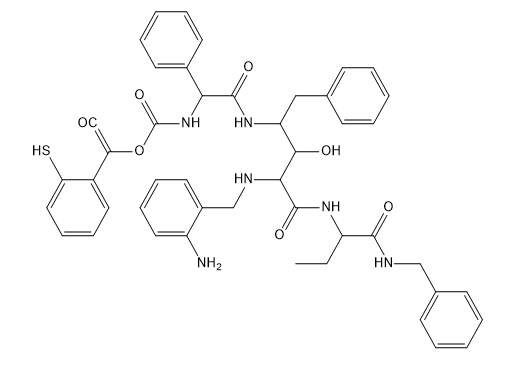 | 1-(2-mercaptophenyl)-2-oxovinyl (2-((4-((2-aminobenzyl)amino)-5-((1-(benzylamino)-1-oxobutan-2-yl)amino)-3-hydroxy-5-oxo-1-phenylpentan-2-yl)amino)-2-oxo-1-phenylethyl)carbamate |
| 819 | 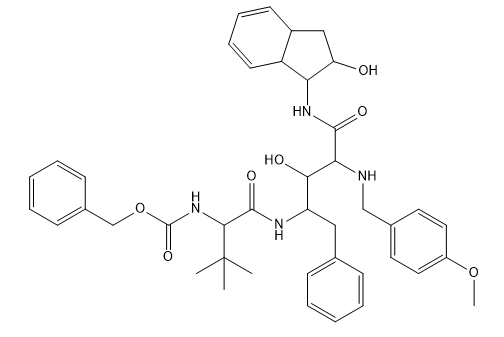 | benzyl (1-((3-hydroxy-5-((2-hydroxy-2,3,3a,7a-tetrahydro-1H-inden-1-yl)amino)-4-((4-methoxybenzyl)amino)-5-oxo-1-phenylpentan-2-yl)amino)-3,3-dimethyl-1-oxobutan-2-yl)carbamate |
| 819a | 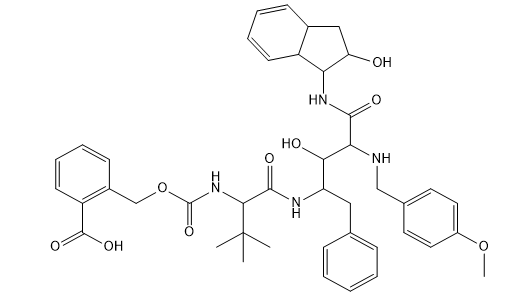 | 2-(8-benzyl-5-(tert-butyl)-9-hydroxy-10-((2-hydroxy-2,3,3a,7a-tetrahydro-1H-inden-1-yl)carbamoyl)-12-(4-methoxyphenyl)-3,6-dioxo-2-oxa-4,7,11-triazadodecyl)benzoic acid |
| 819b | 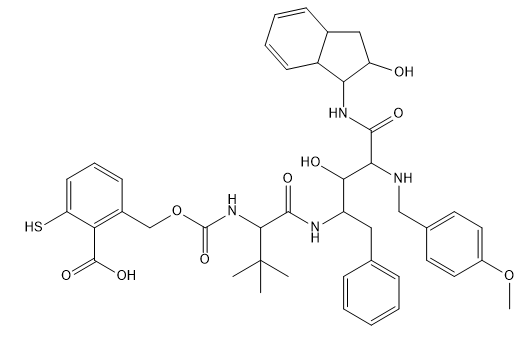 | 2-(8-benzyl-5-(tert-butyl)-9-hydroxy-10-((2-hydroxy-2,3,3a,7a-tetrahydro-1H-inden-1-yl)carbamoyl)-12-(4-methoxyphenyl)-3,6-dioxo-2-oxa-4,7,11-triazadodecyl)-6-mercaptobenzoic acid |
| 819c | 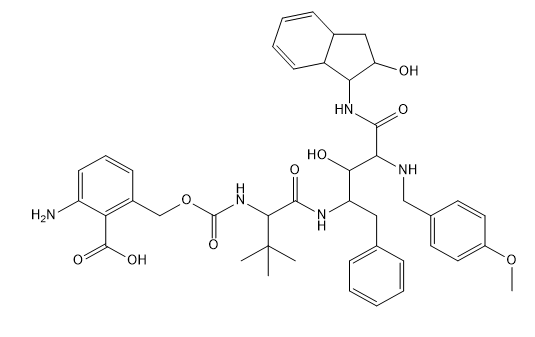 | 2-amino-6-(8-benzyl-5-(tert-butyl)-9-hydroxy-10-((2-hydroxy-2,3,3a,7a-tetrahydro-1H-inden-1-yl)carbamoyl)-12-(4-methoxyphenyl)-3,6-dioxo-2-oxa-4,7,11-triazadodecyl)benzoic acid |
| 819d | 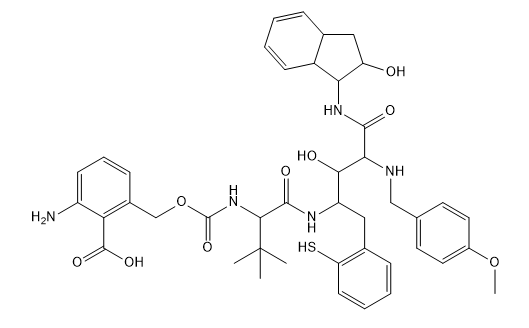 | 2-amino-6-(5-(tert-butyl)-9-hydroxy-10-((2-hydroxy-2,3,3a,7a-tetrahydro-1H-inden-1-yl)carbamoyl)-8-(2-mercaptobenzyl)-12-(4-methoxyphenyl)-3,6-dioxo-2-oxa-4,7,11-triazadodecyl)benzoic acid |
| 819e | 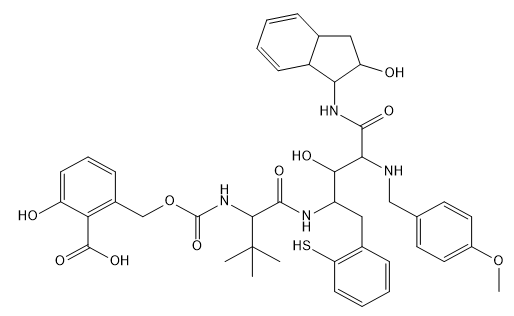 | 2-(5-(tert-butyl)-9-hydroxy-10-((2-hydroxy-2,3,3a,7a-tetrahydro-1H-inden-1-yl)carbamoyl)-8-(2-mercaptobenzyl)-12-(4-methoxyphenyl)-3,6-dioxo-2-oxa-4,7,11-triazadodecyl)-6-hydroxybenzoic acid |
| 847 | 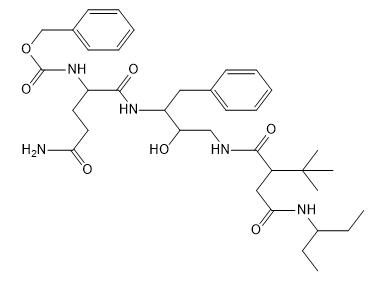 | benzyl (5-amino-1-((4-(2-(tert-butyl)-4-oxo-4-(pentan-3-ylamino)butanamido)-3-hydroxy-1-phenylbutan-2-yl)amino)-1,5-dioxopentan-2-yl)carbamate |
| 847a | 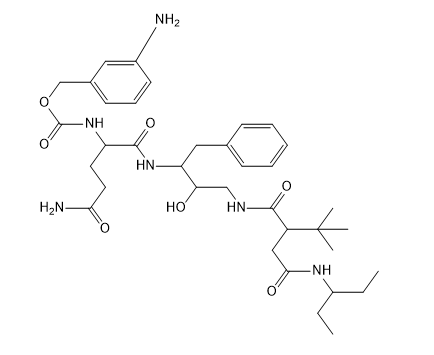 | 3-aminobenzyl (5-amino-1-((4-(2-(tert-butyl)-4-oxo-4-(pentan-3-ylamino)butanamido)-3-hydroxy-1-phenylbutan-2-yl)amino)-1,5-dioxopentan-2-yl)carbamate |
| 847b | 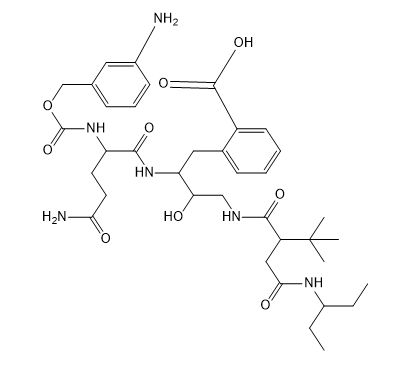 | 2-(2-(5-amino-2-((((3-aminobenzyl)oxy)carbonyl)amino)-5-oxopentanamido)-4-(2-(tert-butyl)-4-oxo-4-(pentan-3-ylamino)butanamido)-3-hydroxybutyl)benzoic acid |
| 847c | 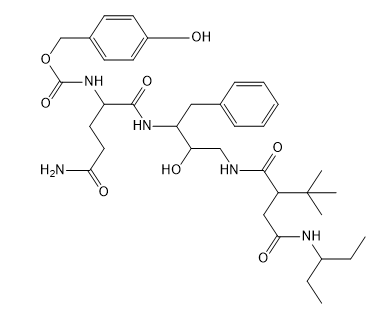 | 4-hydroxybenzyl (5-amino-1-((4-(2-(tert-butyl)-4-oxo-4-(pentan-3-ylamino)butanamido)-3-hydroxy-1-phenylbutan-2-yl)amino)-1,5-dioxopentan-2-yl)carbamate |
| 847d | 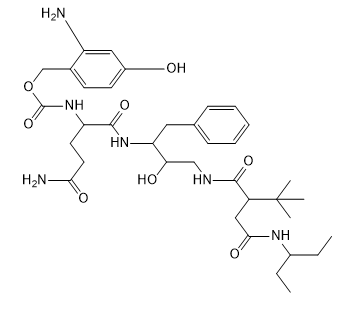 | 2-amino-4-hydroxybenzyl (5-amino-1-((4-(2-(tert-butyl)-4-oxo-4-(pentan-3-ylamino)butanamido)-3-hydroxy-1-phenylbutan-2-yl)amino)-1,5-dioxopentan-2-yl)carbamate |
| 847e | 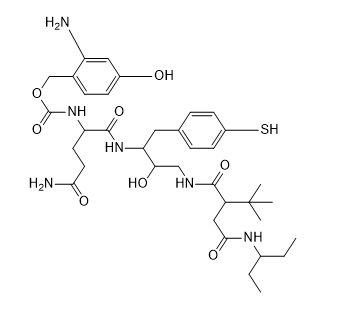 | 2-amino-4-hydroxybenzyl (5-amino-1-((4-(2-(tert-butyl)-4-oxo-4-(pentan-3-ylamino)butanamido)-3-hydroxy-1-(4-mercaptophenyl)butan-2-yl)amino)-1,5-dioxopentan-2-yl)carbamate |
